# Biogenic Amine-Containing 1,4-Naphthoquinones Mediate Extracellular Electron Transfer in *Lactiplantibacillus plantarum*

**DOI:** 10.1021/acs.orglett.5c03438

**Published:** 2025-09-08

**Authors:** Benjamin T. Blackburn, Joseph Barton, Micah Hoernig, Anne M. Brown, Emily Mevers

**Affiliations:** † Department of Chemistry, 1757 Virginia Tech, Blacksburg, Virginia 24061, United States; ‡ Department of Biochemistry, Virginia Tech, Blacksburg, Virginia 24061, United States; ⊥ University Libraries, Virginia Tech, Blacksburg, Virginia 24061, United States

## Abstract

*Lactiplantibacillus plantarum*, a lactic acid gut bacterium, uses exogenous quinones to facilitate extracellular electron transfer (EET) via type II NADH dehydrogenase (Ndh2). To probe Ndh2 specificity, we designed and evaluated a library of biogenic amine-substituted 1,4-naphthoquinones in an Ndh2-dependent EET assay. Analysis of mediator Ndh2 binding interactions revealed that activity correlates with key binding interactions. Specifically, mediators containing aromatic substitutions elicit favorable Ndh2 interactions, promoting EET.

## Graphical abstract

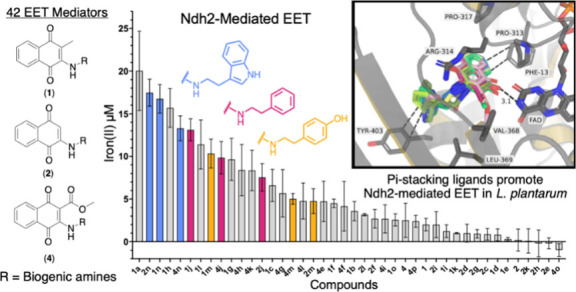

Quinones are naturally occurring small molecules that serve essential biological functions, including acting as cofactors, vitamins and in coloration.^1^ Much of this utilization is due to their favorable reduction potential under biological conditions, switching between the hydroquinone (reduced form) and quinone (oxidized form). Of increasing interest is the ability of quinones to shuttle electrons through membranes to terminal electron acceptors (TEAs), termed extracellular electron transfer (EET).^2–4^ Diverse bacterial strains use EET to respire under anaerobic conditions, and this mechanism has recently been investigated for use in selective biosensing applications.^2,5,6^ For example, *Lactiplantibacillus plantarum*, a lactic acid gut bacterium, uses exogenous analogues of vitamin K, such as menadione (**1**), to facilitate EET through a type II NADH dehydrogenase (Ndh2).^7,8^ *L. plantarum* can utilize a range of TEAs, including iron­(III) oxide nanoparticles and a carbon felt electrode within a bioelectronic system (BES).^9,10^ Our recent study of EET mediator physicochemical properties revealed that Ndh2-dependent EET is driven by the mediators’ lipophilicity (Log*D* at pH 7.4) and predicted free energy of binding (*ΔG*_comp_).^9^ Evaluation of one mediator, 3-amine-menadione (**1a**), in a BES showed that it could produce an incredibly stable current (up to 5 days).

As part of our continued effort to develop quinone mediators capable of Ndh2-dependent EET in *L. plantarum*, we synthesized a diverse library of 1,4-naphthoquinone mediators (Scheme 1). Three scaffolds—menadione (**1**), 1,4-naphthoquinone (**2**), and 1,4-dihydroxy-2-naphthoic acid (DHNA) methyl ester (**4**)—were conjugated to a variety of biogenic amines (**a**–**o**), yielding 42 synthetic compounds exhibiting diverse physicochemical properties that are predicted to elicit a range of biochemical interactions within the Ndh2 binding pocket. This includes biogenic amines that support hydrogen bonding (e.g., aminoethanol and aminopropanol), van der Waals (e.g., ethylamine, methylamine, pyrrolidine, and isobutylamine), ionic (e.g., taurine and cadaverine), and π-stacking (e.g., phenethylamine, tryptamine, tyramine, and histamine) interactions. Evaluation of the EET properties using an iron­(III) oxide nanoparticle reduction assay revealed key active site interactions that promote Ndh2-dependent EET. In addition, we developed a predictive, quantitative metric or composite score for the biological process. The composite score is based on the mediator’s *ΔG*_comp_ and ADME properties (Schrodinger Maestro version 14.1 QikProp).

**Scheme 1:**
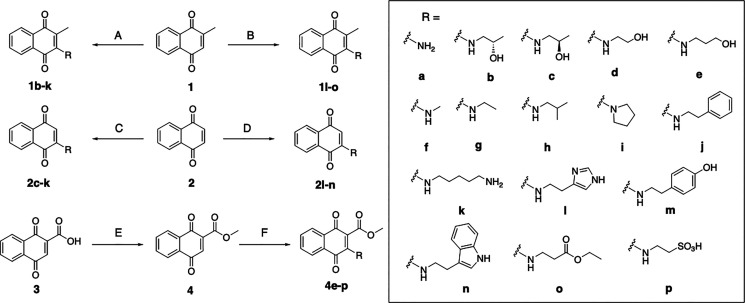
Synthetic Procedures for the Formation of Biogenic Amine-Containing 1,4-Naphthoquinones^a^

a Conditions: (A) amine (neat), HClO_4_/SiO_2_ (0.1 equiv), sonicated for 20 min and then heated to 60 °C, 2 min, 20–44%; (B) amine (10 equiv), MeCN, 70 °C, 3–5 h, 7–23%; (C) amine (neat), HClO_4_/SiO_2_ (0.1 equiv), sonicated for 20 min and then heated to 60 °C, 2 min, 23–98%; (D) amine (10 equiv), MeCN, 70 °C, 3–5 h, 73–93%; (E) MeI (2.0 equiv), NaHCO_3_ (2.0 equiv), DMF, N_2_, rt, 22 h, 77%; (F) amine (4.0 equiv), MeCN, 50 °C, 5 h, 16–71%.

We began our efforts by conjugating **1** to various amines using a perchloric acid catalyst (Scheme 1). First, the liquid amines (**b**–**k**) were reacted neat with **1** in the presence of catalytic perchloric acid.^11^ The reaction mixture was sonicated for 20 min and then heated to 60 °C for 2 min, yielding **1b**–**1k** (20–44%). To prevent double quinone addition to **k**, **1** was dissolved in MeCN, and the solution was added dropwise to a reaction vessel containing excess amine. Generating mediators using solid amines (**l–o**) followed a similar method, where the amine and **1** were first dissolved in acetonitrile (MeCN) and then the mixture was heated to 70 °C for 3–5 h, yielding **1l**–**1o** (7–23%). Solid amines posed more difficult additions due to poor solubility. For example, amine **m** was relatively insoluble in MeCN, so a 1:1 ddH_2_O/MeCN mixture was employed.

To increase the polarity of mediators in comparison to **1** (Log*D* = 2.17), two more polar scaffolds, **2** (Log*D* = 1.65) and **4** (Log*D* = 1.68), were derivatized with the same biogenic amines. First, amines **c**–**n** were reacted with **2** under the same perchloric acid conditions to yield **2c**–**2n** (23–100%). As for the derivatization of scaffold **4**, DHNA (**3**) was methylated by treatment with methyl iodide and sodium bicarbonate in dimethylformamide (DMF) at room temperature for 22 h, yielding **4** (77%).^12^ Amines **d**–**o** were then conjugated to **4** using similar methods, yielding **4d**–**4o** (16–71%). Reactions to generate **4e** and **4f** were conducted at room temperature due to low liquid amine boiling points. Drops of ddH_2_O were added to reaction mixtures of **4k**–**4o** to dissolve the solid amine fully.

The Ndh2-dependent EET activity of all mediators was quantified using an established iron­(III) nanoparticle assay.^9^ All mediators were evaluated for EET properties against two *L. plantarum* strains, an Ndh2 competent strain (*ΔdmkAΔndh1*) and an Ndh2 deficient strain (*ΔdmkAΔndh1Δndh2*), at 5 μM (Figure 1A and Figure S1). Ndh2-dependent EET activity was calculated by the difference in iron­(II) produced by the Ndh2 competent and deficient strains (Figure 1B and Table 1). Overall, the biogenic amine-containing mediators exhibited robust Ndh2-dependent EET activity, as there was little iron­(II) produced by the Ndh2 deficient strain and significant iron­(II) production by the Ndh2 competent strain (Tables S1). No significant trends were observed between Ndh2 EET activity and the physicochemical properties of the mediators (Figures S2–S4), indicating factors such as size, aromaticity, and positioning in the binding cavity relative to flavin adenine dinucleotide (FAD) should be considered in ranking and score generation.

**Figure 1:**
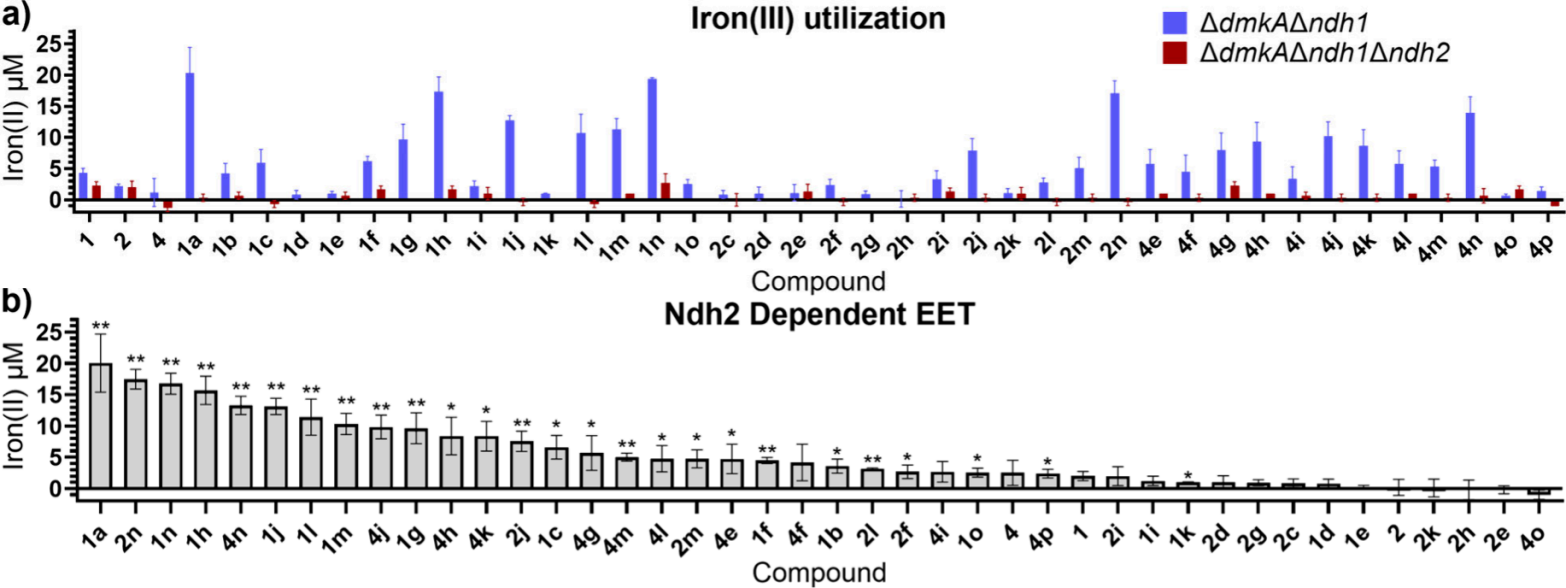
Iron­(III) oxide nanoparticle reduction assay data (a) across both strains of *L. plantarum*, Ndh2 competent (blue) and Ndh2 deficient (red), and (b) for Ndh2-mediated EET, calculated by taking the difference in iron­(II) production by Ndh2 competent vs deficient strains. Error bars represent the standard deviation across biological triplicates (*n* = 3). **p < 0.01 and *p < 0.05, relative to the solvent control using a two-tailed *t* test. Negative values represent variability in the assay rather than consumption of iron­(II).

**Table 1:** Mediators of Ndh2-Dependent EET

mediator	Ndh2-dependent EET (μM Fe^2+^)^a^	*P* value^b^	composite score
**1a**	20.0 ± 4.6	0.0038	10.2
**2n**	17.5 ± 1.6	0.0017	8.16
**1n**	16.8 ± 1.7	0.0020	8.94
**1h**	15.7 ± 2.3	0.0031	7.53
**4n**	13.3 ± 1.5	0.0027	8.86
**1j**	13.1 ± 1.3	0.0025	6.38
**1l**	11.4 ± 2.9	0.0073	4.36
**1m**	10.3 ± 1.7	0.0052	6.67
**4j**	9.85 ± 1.9	0.0065	4.56
**1g**	9.65 ± 2.5	0.0087	2.08
**4h**	8.39 ± 3.0	0.014	–1.32
**4k**	8.35 ± 2.4	0.011	0.01
**2j**	7.55 ± 1.6	0.0093	3.42
**1c**	6.60 ± 1.9	0.014	–0.78
**4g**	5.68 ± 2.8	0.027	2.45
**4m**	5.02 ± 0.6	0.0083	1.83
**4l**	4.77 ± 2.1	0.030	1.21
**2m**	4.76 ± 1.4	0.021	0.34
**4e**	4.72 ± 2.4	0.034	–4.53
**1f**	4.50 ± 0.5	0.0079	–0.04
**1b**	3.59 ± 1.1	0.028	0.8
**2l**	3.17 ± 0.1	0.0043	–1.37
**2f**	2.65 ± 1.1	0.047	–1.12
**1o**	2.56 ± 0.7	0.035	–3.13
**4p**	2.40 ± 0.7	0.023	–2.18
**1k**	1.00 ± 0.1	0.023	–2.29

a The iron­(II) concentration was determined using a ferrous sulfate calibration curve.

b *p* value calculated relative to the solvent blank.

Molecular docking studies using a refined homology model of *L. plantarum* Ndh2 revealed binding modes that organize into chemically and structurally coherent clusters with mechanistic implications for EET (Figure S5). Structural analysis of the model showed a Phe-Pro-Pro aromatic cleft and an Arg-Glu electrostatic clamp^13^ comprising portions of the binding site, which proved instrumental in defining the binding profile of top-performing compounds (Figure 2A). *ΔG*_comp_ and ADME values for each of the output poses across all compounds were calculated. Composite scores were obtained as outputs of a RidgeCV linear regression model trained using on ADME data and *ΔG*_comp_ values against measured EET. The composite score for each mediator generated by the model strongly correlates positively (*p* < 0.0001) with Ndh2-dependent EET activity (Figure 2B), and this model could be used in future studies to predict EET properties of novel mediators.

**Figure 2:**
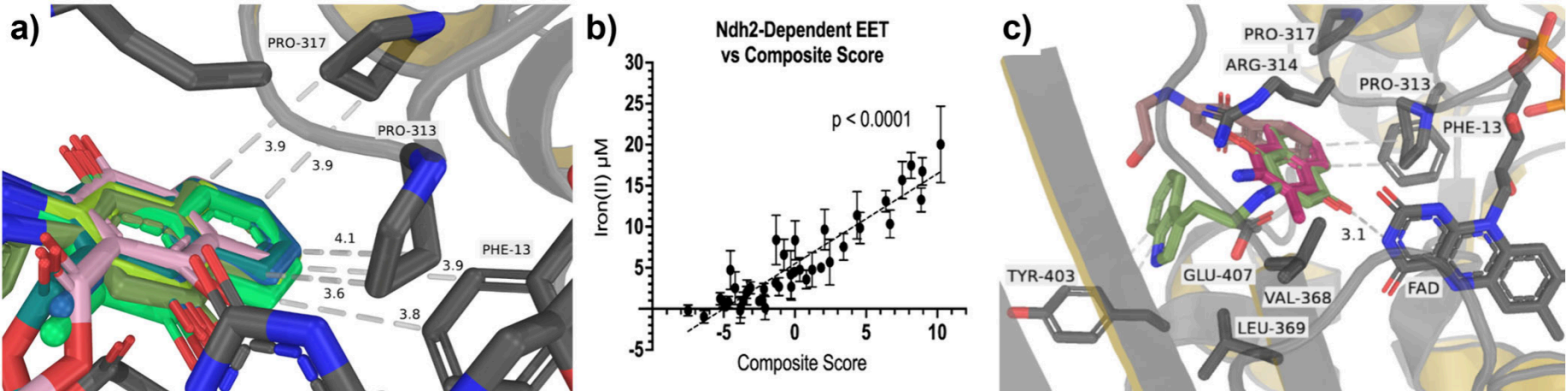
Docked poses in the *L. plantarum* Ndh2 binding site. (a) Compounds in the populated cluster of docked poses displaying the proper distance and orientation from the aromatic cleft (Phe-13, Pro-313, and Pro-317) conducive to T-stack π interactions. (b) Correlation between Ndh2-depenedent EET and composite score (*p* < 0.0001). (c) Compounds **1a**, **2n**, and **2d**, with highlighted interactions between the fused ring system and the aromatic cleft, the carbonyl oxygen and FAD, and the aromatic tail and Tyr-40.

Analysis of the molecular docking poses for each mediator led to clustering of mediators exhibiting similar binding interactions. The first compound cluster is comprised of 11 mediators (**1a**, **1g**, **1j**, **1l**, **1n**, **2j**, **2n**, **4h**, **4j**, **4k**, and **4n**), which contain many of the aromatic-containing side chains. Interestingly, all of these mediators are within the top 13 EET performing mediators, six within the 10 lowest *ΔG*_comp_. In addition, eight in the top 10 highest composite scores (Table 1). Mediators in this cluster are positioned within the Arg-Glu electrostatic clamp (Figure S6A), with the fused aromatic ring system oriented toward the amine tail extending out of the canonical binding pocket toward Leu-369 (Figure S6B). This orientation of aromatic-containing compounds is conducive for EET, as the aromatic cleft, polar clamp, and hydrophobic environment provided by Leu-369 collectively stabilize the naphthoquinones and facilitate productive positioning of the carbonyl group within the binding site in the proximity of FAD.^13^ The second, less populated compound cluster includes **1m** and **1h** (Figure S6C). In this orientation, the fused ring system occupies a similar region of the binding pocket as observed in the first cluster but adopts a flipped alignment, with the unsubstituted aromatic positioned toward Leu-369 and the amine tail extending toward Pro-317. Despite this flipped configuration, the carbonyl group remains positioned within an appropriate distance of FAD, maintaining the potential for electron transfer.

Surprisingly, the most active mediator was the only primary amine, **1a**, which is relatively polar (Log*D* = 0.02) and possesses the top composite score. The molecular docking experiment suggests it has favorable interactions with Ndh2, with its binding stabilized by primary amine H-bonding interactions with a key Glu-407 residue and T-stack π interactions with the aromatic cleft in the Ndh2 active site (Figure S6D). To probe this further, we evaluated the activity profile of three additional primary amines, **2a**, **3a**, and **4a**, which were synthesized using similar methods as described above. Compounds **2a**, **3a**, and **4a** show a similar binding pattern with the polar amine group situated between the Arg-Glu clamp and the carbonyl oriented toward the canonical binding site. A slight rotation was seen toward Tyr-403 for **2a**–**4a** compared to compound **1a** (Figure S7). All three compounds could perform EET at the same or improved magnitudes when compared to **1a** (Figure S8).

Docked poses across all 45 compounds suggest a conserved pattern of binding. Top-performing candidates are situated with the polar amine between the arginine-glutamic acid clamp, the fused ring system located in the canonical binding site, suggestive of T stack, π orbital interactions with the aromatic cleft, and a flexible, nonpolar, or aromatic amine tail with positioning suggestive of π stacking with Tyr-403 (Figure 2C) and nonpolar interaction with Leu-369 and Val-368 (Figure S6E). Mediators with polar, nonaromatic amines, such as **1d**, **2d**, **1e**, **2e**, and **4e**, are less likely to assume this orientation due to a lack of energetic favorability associated with placing a polar terminal hydroxyl group within the hydrophobic region of the binding pocket (Figure S6F). Mediators with pyrrolidine amine tails (**1i**, **2i**, and **4i**) lack the necessary size and conformational flexibility to effectively engage in π stacking interactions with Tyr-403 or the aromatic cleft with energetic favorability, as indicated in *ΔG*_comp_ values and the composite score. As a result, they likely cannot adopt a binding orientation that sufficiently anchors the ligand within the pocket or supports efficient electron transfer (Figure S6G).

In summary, we assembled a library of 45 naphthoquinones, 42 of which were conjugated to diverse biogenic amines, and showed that many amine-substituted mediators perform selective Ndh2-dependent EET in *L. plantarum*. Mediators possessing aromatic-containing amines (e.g., tryptamine and phenethylamine) are top-performing mediators as they elicit favorable π stacking interactions with Tyr-403 within the Ndh2 active site. Conversely, mediators possessing polar amines (e.g., aminoethanol and pyrrolidine) are much less active, likely due to unfavorable binding. Although physicochemical properties, such as Log*D*, did not strongly inversely correlate with Ndh2-dependent EET as seen in our previous study,^9^ the expansion of the chemical diversity of the mediators allowed us to highlight features of the quinones that are more related to the EET process. Gaining a better understanding of the mechanism of Ndh2-dependent EET is essential for designing small-molecule mediators and developing multichannel sensing applications.

## Supplementary Material



## Data Availability

The data underlying this study are available in the published article and its Supporting Information.

## Notes

Experimental details, NMR data, NMR spectra, biological data, and *XYZ* coordinates (PDF)
