# Supplementary material for: Biogenic Amine-Containing 1,4-Naphthoquinones Mediate Extracellular Electron Transfer in Lactiplantibacillus plantarum
Source: Org Lett. 2025 Sep 8;27(37):10548–52. doi: 10.1021/acs.orglett.5c03438 (PMC12455635; doi:10.1021/acs.orglett.5c03438)
Supplement: Supplementary file 1 [file ol5c03438_si_001.pdf]

Supporting Information

Biogenic Amine-Containing 1,4-Naphthoquinones Mediate Extracellular Electron Transfer in *Lactiplantibacillus plantarum*

Benjamin T. Blackburn,[†,#] Joseph Barton,[†,#] Micah Hoernig,[‡,^] Anne M. Brown,[‡,^] Emily Mevers[†,]*

[†]Department of Chemistry, Virginia Tech, Blacksburg, VA, 24061, USA

[‡]Department of Biochemistry, Virginia Tech, Blacksburg, VA, 24061, USA

[^]University Libraries, Virginia Tech, Blacksburg, VA, 24061, USA

KEYWORDS: extracellular electron transfer, naphthoquinone, biogenic amine, mediator

[#]Authors contributed equally to this work

Table of Contents

1. Experimental Methods
General experimental procedures ....pg. S4
Safety statement ....pg. S4
Synthetic procedures ....pg. S4
Computational methods ....pg. S20
Biological experimental methods. ....pg. S21
References ....pg. S22
Supplemental table
Table S1: Ndh-2 dependent activity for mediators ....pg. S23
Table S2: Reduction potential of all mediators ....pg. S25
Supplemental Figures
Figure S1: Iron(II)-ferrozine standard curve ....pg. S27
Figure S2: Ndh2 Dependent EET vs predicted free energy of binding (ΔG_{comp}) ....pg. S27
Figure S3: Ndh2 Dependent EET vs 1e$^-$ reduction potential ....pg. S28
Figure S4: Ndh2 Dependent EET vs Ndh2-dependent EET vs Log*D* ....pg. S28
Figure S5: Homology Model ....pg. S29
Figure S6: Docked poses in Homology Model ....pg. S30
Figure S7: Comparison of activity profile of mediators with a primary amine (**1a**, **2a**, **3a**, and **4a**) ....pg. S31
Figure S8: EET activity for primary amine analogs ....pg. S31
Figure S9: ^{1}H NMR Spectra (500 MHz) for 3-[(*S*)-amino-2-propanol]-menadione (**1b**) in $CDCl_3$ ....pg. S32
Figure S10: ^{13}C NMR Spectra (125 MHz) for 3-[(*S*)-amino-2-propanol]-menadione (**1b**) in $CDCl_3$ ....pg. S32
Figure S11: ^{1}H NMR Spectra (500 MHz) for 3-[(*R*)-amino-2-propanol]-menadione (**1c**) in $CDCl_3$ ....pg. S33
Figure S12: ^{13}C NMR Spectra (125 MHz) for 3-[(*R*)-amino-2-propanol]-menadione (**1c**) in $CDCl_3$ ....pg. S33
Figure S13: ^{1}H NMR Spectra (500 MHz) for 3-Aminoethanol-menadione (**1d**) in $CDCl_3$ ....pg. S34
Figure S14: ^{13}C NMR Spectra (125 MHz) for 3-Aminoethanol-menadione (**1d**) in $CDCl_3$ ....pg. S34
Figure S15: ^{1}H NMR Spectra (500 MHz) for 3-Aminopropanol menadione (**1e**) in $CDCl_3$ ....pg. S35
Figure S16: ^{13}C NMR Spectra (125 MHz) for 3-Aminopropanol menadione (**1e**) in $CDCl_3$ ....pg. S35
Figure S17: ^{1}H NMR Spectra (500 MHz) for 3-Methylamine-menadione (**1f**) in $CDCl_3$ ....pg. S36
Figure S18: ^{13}C NMR Spectra (125 MHz) for 3-Methylamine-menadione (**1f**) in $CDCl_3$ ....pg. S36
Figure S19: ^{1}H NMR Spectra (500 MHz) for 3-Ethylamine-menadione (**1g**) in $CDCl_3$ ....pg. S37
Figure S20: ^{13}C NMR Spectra (125 MHz) for 3-Ethylamine-menadione (**1g**) in $CDCl_3$ ....pg. S37
Figure S21: ^{1}H NMR Spectra (500 MHz) for 3-Isobutylamine-menadione (**1h**) in $CDCl_3$ ....pg. S38
Figure S22: ^{13}C NMR Spectra (125 MHz) for 3-Isobutylamine-menadione (**1h**) in $CDCl_3$ ....pg. S38
Figure S23: ^{1}H NMR Spectra (500 MHz) for 3-Pyrrolidine-menadione (**1i**) in $(CD_3)_2SO$ ....pg. S39
Figure S24: ^{13}C NMR Spectra (125 MHz) for 3-Pyrrolidine-menadione (**1i**) in $(CD_3)_2SO$ ....pg. S39
Figure S25: ^{1}H NMR Spectra (500 MHz) for 3-Phenethylamine-menadione (**1j**) in $CDCl_3$ ....pg. S40
Figure S26: ^{13}C NMR Spectra (125 MHz) for 3-Phenethylamine-menadione (**1j**) in $CDCl_3$ ....pg. S40
Figure S27: ^{1}H NMR Spectra (500 MHz) for 3-Cadaverine-menadione (**1k**) in $CDCl_3$ ....pg. S41
Figure S28: ^{13}C NMR Spectra (125 MHz) for 3-Cadaverine-menadione (**1k**) in $CDCl_3$ ....pg. S41
Figure S29: ^{1}H NMR Spectra (500 MHz) for 3-Histamine-menadione (**1l**) in CD_3OD ....pg. S42
Figure S30: ^{13}C NMR Spectra (125 MHz) for 3-Histamine-menadione (**1l**) in CD_3OD ....pg. S42
Figure S31: ^{1}H NMR Spectra (500 MHz) for 3-Tyramine-menadione (**1m**) in $CDCl_3$ ....pg. S43
Figure S32: ^{13}C NMR Spectra (125 MHz) for 3-Tyramine-menadione (**1m**) in $CDCl_3$ ....pg. S43
Figure S33: ^{1}H NMR Spectra (500 MHz) for 3-Tryptamine-menadione (**1n**) in $CDCl_3$ ....pg. S44
Figure S34: ^{13}C NMR Spectra (125 MHz) for 3-Tryptamine-menadione (**1n**) in $CDCl_3$ ....pg. S44
Figure S35: ^{1}H NMR Spectra (500 MHz) for 3-*β*-alanine-ethyl ester-menadione (**1o**) in $CDCl_3$ ....pg. S45
Figure S36: ^{13}C NMR Spectra (125 MHz) for 3-*β*-alanine-ethyl ester-menadione (**1o**) in $CDCl_3$ ....pg. S45
Figure S37: ^{1}H NMR Spectra (500 MHz) for 2-amine-naphthoquinone (**2a**) in $CDCl_3$ ....pg. S46
Figure S38: ^{13}C NMR Spectra (125 MHz) for 2-amine-naphthoquinone (**2a**) in $CDCl_3$ ....pg. S46
Figure S39: ^{1}H NMR Spectra (500 MHz) for 2-[(*R*)-amino-2-propanol]-naphthoquinone (**2c**)

in $CDCl_3$....pg. S47
Figure S40: ^{13}C NMR Spectra (125 MHz) for 2-[(*R*)-amino-2-propanol]-naphthoquinone (**2c**) in $CDCl_3$....pg. S47
Figure S41: ^{1}H NMR Spectra (500 MHz) for 2-Aminoethanol-naphthoquinone (**2d**) in $CDCl_3$....pg. S48
Figure S42: ^{13}C NMR Spectra (125 MHz) for 2-Aminoethanol-naphthoquinone (**2d**) in $CDCl_3$....pg. S48
Figure S43: ^{1}H NMR Spectra (500 MHz) for 2-Aminopropanol-naphthoquinone (**2e**) in $CDCl_3$....pg. S49
Figure S44: ^{13}C NMR Spectra (125 MHz) for 2-Aminopropanol-naphthoquinone (**2e**) in $CDCl_3$....pg. S49
Figure S45: ^{1}H NMR Spectra (500 MHz) for 2-Methylamine-naphthoquinone (**2f**) in CD_3OD....pg. S50
Figure S46: ^{13}C NMR Spectra (125 MHz) for 2-Methylamine-naphthoquinone (**2f**) in CD_3OD....pg. S50
Figure S47: ^{1}H NMR Spectra (500 MHz) for 2-Ethylamine-naphthoquinone (**2g**) in $CDCl_3$....pg. S51
Figure S48: ^{13}C NMR Spectra (125 MHz) for 2-Ethylamine-naphthoquinone (**2g**) in $CDCl_3$....pg. S51
Figure S49: ^{1}H NMR Spectra (500 MHz) for 2-Isobutylamine-naphthoquinone (**2h**) in $CDCl_3$....pg. S52
Figure S50: ^{13}C NMR Spectra (125 MHz) for 2-Isobutylamine-naphthoquinone (**2h**) in $CDCl_3$....pg. S52
Figure S51: ^{1}H NMR Spectra (500 MHz) for 2-Pyrrolidine-naphthoquinone (**2i**) in $CDCl_3$....pg. S53
Figure S52: ^{13}C NMR Spectra (125 MHz) for 2-Pyrrolidine-naphthoquinone (**2i**) in $CDCl_3$....pg. S53
Figure S53: ^{1}H NMR Spectra (500 MHz) for 2-Phenethylamine-naphthoquinone (**2j**) in $CDCl_3$....pg. S54
Figure S54: ^{13}C NMR Spectra (125 MHz) for 2-Phenethylamine-naphthoquinone (**2j**) in $CDCl_3$....pg. S54
Figure S55: ^{1}H NMR Spectra (500 MHz) for 2-Cadaverine-naphthoquinone (**2k**) in CD_3OD....pg. S55
Figure S56: ^{13}C NMR Spectra (125 MHz) for 2-Cadaverine-naphthoquinone (**2k**) in CD_3OD....pg. S55
Figure S57: ^{1}H NMR Spectra (500 MHz) for 2-Histamine-naphthoquinone (**2l**) in CD_3OD.....pg. S56
Figure S58: ^{13}C NMR Spectra (125 MHz) for 2-Histamine-naphthoquinone (**2l**) in CD_3OD....pg. S56
Figure S59: ^{1}H NMR Spectra (500 MHz) for 2-Tyramine-naphthoquinone (**2m**) in CD_3OD....pg. S57
Figure S60: ^{13}C NMR Spectra (125 MHz) for 2-Tyramine-naphthoquinone (**2m**) in CD_3OD....pg. S57
Figure S61: ^{1}H NMR Spectra (500 MHz) for 2-Tryptamine-naphthoquinone (**2n**) in $CDCl_3$....pg. S58
Figure S62: ^{13}C NMR Spectra (125 MHz) for 2-Tryptamine-naphthoquinone (**2n**) in $CDCl_3$....pg. S58
Figure S63: ^{1}H NMR Spectra (500 MHz) for 1,4-Dihydroxy-2-naphthoic acid methyl ester (**4**) in CD_3OD....pg. S59
Figure S64: ^{13}C NMR Spectra (125 MHz) for 1,4-Dihydroxy-2-naphthoic acid- methyl ester (**4**) in CD_3OD....pg. S59
Figure S65: ^{1}H NMR Spectra (500 MHz) for 3-amine-DHNA methyl ester (**4a**) in CD_3OD....pg. S60
Figure S66: ^{13}C NMR Spectra (125 MHz) for 3-amine-DHNA methyl ester (**4a**) in CD_3OD....pg. S60
Figure S67: ^{1}H NMR Spectra (500 MHz) for 3-aminopropanol-DHNA methyl ester (**4e**) in CD_3CN....pg. S61
Figure S68: ^{13}C NMR Spectra (125 MHz) for 3-aminopropanol-DHNA methyl ester (**4e**) in CD_3CN....pg. S61
Figure S69: ^{1}H NMR Spectra (500 MHz) for 3-Methylamine-DHNA methyl ester (**4f**) in CD_3CN....pg. S62
Figure S70: ^{13}C NMR Spectra (125 MHz) for 3-Methylamine-DHNA methyl ester (**4f**) in CD_3CN....pg. S62
Figure S71: ^{1}H NMR Spectra (500 MHz) for 3-Ethylamine-DHNA methyl ester (**4g**) in $CDCl_3$....pg. S63
Figure S72: ^{13}C NMR Spectra (125 MHz) for 3-Ethylamine-DHNA methyl ester (**4g**) in $CDCl_3$....pg. S63
Figure S73: ^{1}H NMR Spectra (500 MHz) for 3-Isobutylamine-DHNA methyl ester (**4h**) in $CDCl_3$....pg. S64
Figure S74: ^{13}C NMR Spectra (125 MHz) for 3-Isobutylamine-DHNA methyl ester (**4h**) in $CDCl_3$....pg. S64
Figure S75: ^{1}H NMR Spectra (500 MHz) for 3-Pyrrolidine-DHNA methyl ester (**4i**) in $CDCl_3$....pg. S65
Figure S76: ^{13}C NMR Spectra (125 MHz) for 3-Pyrrolidine-DHNA methyl ester (**4i**) in $CDCl_3$....pg. S65
Figure S77: ^{1}H NMR Spectra (500 MHz) for 3-Phenethylamine-DHNA methyl ester (**4j**) in $CDCl_3$....pg. S66
Figure S78: ^{13}C NMR Spectra (125 MHz) for 3-Phenethylamine-DHNA methyl ester (**4j**) in $CDCl_3$....pg. S66

Figure S79: ^{1}H NMR Spectra (500 MHz) for for 3-cadaverine-DHNA methyl ester (**4k**) in CD_3OD....pg. S67
Figure S80: ^{13}C NMR Spectra (125 MHz) for for 3-cadaverine-DHNA methyl ester (**4k**) in CD_3OD....pg. S67
Figure S81: ^{1}H NMR Spectra (500 MHz)for 3-Histamine-DHNA methyl ester (**4l**) in CD_3OD....pg. S68
Figure S82: ^{13}C NMR Spectra (125 MHz) for 3-Histamine-DHNA methyl ester (**4l**) in CD_3OD....pg. S68
Figure S83: ^{1}H NMR Spectra (500 MHz) for 3-Tyramine-DHNA methyl ester (**4m**) in CD_3CN....pg. S69
Figure S84: ^{13}C NMR Spectra (125 MHz) for 3-Tyramine-DHNA methyl ester (**4m**) in CD_3CN....pg. S69
Figure S85: ^{1}H NMR Spectra (500 MHz) for 3-Tryptamine-DHNA methyl ester (**4n**) in CD_3OD....pg. S70
Figure S86: ^{13}C NMR Spectra (125 MHz) for 3-Tryptamine-DHNA methyl ester (**4n**) in CD_3OD....pg. S70
Figure S87: ^{1}H NMR Spectra (500 MHz) for 3-β-alanine-ethyl ester-DHNA methyl ester (**4o**) in CD_3OD....pg. S71
Figure S88: ^{13}C NMR Spectra (125 MHz) for 3-β-alanine-ethyl ester-DHNA methyl ester (**4o**) in CD_3OD....pg. S71
Figure S89: ^{1}H NMR Spectra (500 MHz) for 3-Taurine-DHNA methyl ester (**4p**) in CD_3OD....pg. S72
Figure S90: ^{13}C NMR Spectra (125 MHz) for 3-Taurine-DHNA methyl ester (**4p**) in CD_3OD....pg. S72
XYZ coordinates for all compounds....pg. S73

1. Experimental Methods

General Experimental Procedures: Optical rotation data was recorded on a JASCO P-2000 polarimeter. NMR spectra were recorded with deuterated methanol, chloroform, acetonitrile, and DMSO with the residual solvent peak as internal standards (δ_C 49.2, δ_H 3.31 for CHD_2OD; δ_C 77.0, δ_H 7.26 for $CHCl_3$; δ_C 118.2, δ_H 1.93 for CHD_2CN; δ_C 39.5, δ_H 2.50 for CHD_2SOCD_3) on Bruker Neo 400 MHz equipped with an inverse Probe and Bruker Avance II 500 MHz instrument equipped with a broadband CBPPO Prodigy Probe (500 and 125 MHz). LR-LCMS data were obtained using an Agilent 1200 series HPLC system equipped with a photo-diode array detector and a Thermo LTQ mass spectrometer. HR-ESI-MS was carried out using a Shimadzu LC-q-TOF Mass Spectrometer equipped with an uHPLC system. All solvents were of HPLC quality. Flash chromatography purification was carried out with the Biotage® Selekt automated flash chromatography system with a Silicycle SiliSep™ 25g cartridge (Part no. FLH-R10030B-ISO25). Prep HPLC purification done with a Waters SymmetryPrep™ C18 7 µm (19 x 150 mm) column (Part no. WAT066240). All reactions were carried out with standard Schlenk line techniques under N_2 (*g*). Glassware, stir bars, and hygroscopic salt reagents were flame dried or placed under high vacuum for 24 h prior to reactions and ACS grade anhydrous solvents were used.

Safety statement: No unexpected or unusually high safety hazards were encountered. All chemicals were used in accordance with their corresponding safety data sheets. Always read chemical safety data sheets when replicating the reported synthetic protocols.

a. Synthetic Methods

Use of 3-amine-menadione (1a) and 2-amino-3-carboxy-1,4-naphthoquinone (ACNQ; 3a): Compounds **1a** and **3a** were used from a previous study, where it was purified and characterized.[1]

Procedure for $HClO_4$ on SiO_2 for Amine Addition Reactions: To a round bottom flask, 23.7 g silica gel (230-400 mesh) was suspended in 70 mL of ethyl ether, and stirred until fully immersed. Then 1.8 g (1.1 mL) perchloric acid was added and stirred for an additional 2 min. The mixture was concentrated under vacuum. The dried material was then placed under vacuum (1.08 ATM) and heated to 60 ℃ using an oil bath for 72 h. The reaction mixture was stored under N_2 (g) in glass vials for later use in amine addition reactions without purification.

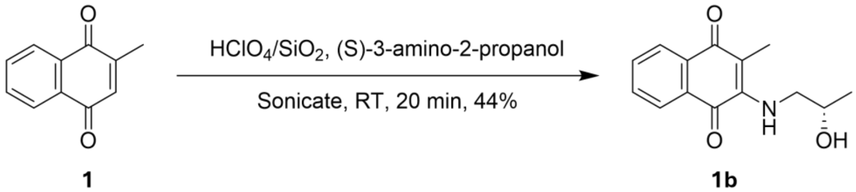

Preparation of 3-[(*S*)-amino-2-propanol]-menadione (1b): Menadione (**1**) (0.25 mmol, 43 mg) and $HClO_4$ on SiO_2 (0.025 mmol, 5.0 mg), was dissolved in (*S*)-3-amino-2-propanol (2.5 mmol, 0.19 mL). The mixture was sonicated for 20 min, then heated to 60 °C in an oil bath for 2 min. The reaction was vacuum filtered and extracted biphasically with EtOAc (3 x 20 mL) and ddH_2O. The organic layers were collected, combined, and concentrated under vacuum. Purification of the crude material was carried out via reverse phase prep HPLC using a gradient of MeCN + 0.1% formic acid (FA)/H_2O + 0.1% FA, starting at 35% MeCN + 0.1% FA and ramping to 65% MeCN + 0.1% FA over 24 min at a flow rate of 10 mL/min, which yielded a dark red oil (27 mg, 44%); **Optical Rotation** $[\alpha]_D^{20}$ +20.9 (c 0.0184, MeOH); ^{1}**H NMR** (500 MHz, $CDCl_3$) δ_H 8.06 (d, 1H, J = 7.6), 7.98 (d, 1H, J = 7.6) 7.66 (t, 1H, J = 7.1), 7.57 (t, 1H, J = 7.1), 6.01 (bs, 1H), 4.03 (m, 1H), 3.67 (m, 1H), 3.43 (m, 1H), 2.20 (s, 3H), 1.92 (bs, 1H), 1.29 (d, 3H, J = 6.4); ^{13}**C NMR** (125 MHz, $CDCl_3$) δ_C 183.8, 182.6, 146.6, 134.4, 133.4, 132.1, 130.5, 126.3, 126.2, 113.2, 67.6, 52.3, 21.2, 11.5; **HRMS (ESI):** calc for $C_{14}H_{16}NO_3^+$ 246.1125, found $[M+H]^+$ 246.1129, Δ1.6.

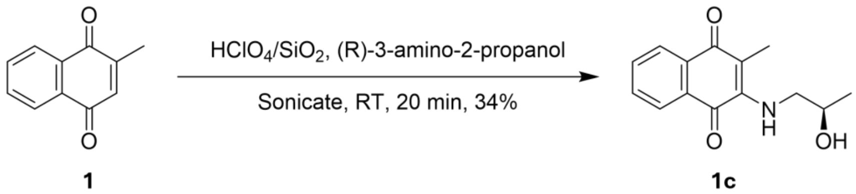

Preparation of 3-[(*R*)-amino-2-propanol)-menadione (1c): Menadione (**1**) (0.25 mmol, 43 mg) and $HClO_4$ on SiO_2 (0.025 mmol, 5.0 mg), was dissolved in (*R*)-3-amino-2-propanol (2.5 mmol, 0.19 mL). The mixture was sonicated for 20 min, then heated to 60 °C in an oil bath for 2 min. The reaction was vacuum filtered and extracted biphasically with EtOAc (3 x 20 mL) and ddH_2O. The organic layers were collected, combined, and concentrated under vacuum. Purification of the crude material was carried out via reverse phase prep HPLC using a gradient of MeCN + 0.1% FA/H_2O + 0.1% FA, starting at 35% MeCN + 0.1% FA and ramping to 65% MeCN + 0.1% FA over 24 min at a flow rate of 10 mL/min, which yielded a dark red oil (27 mg, 34%); **Optical Rotation** $[\alpha]_D^{20}$ -16.8 (c 0.0189, MeOH); ^{1}**H NMR** (500 MHz, $CDCl_3$) δ_H 8.05 (dd, 1H, J = 7.7, 1.1), 7.96 (dd, 1H, J = 7.7, 1.2) 7.66 (td, 1H, J = 7.6, 1.3), 7.56 (td, 1H, J = 7.6, 1.3), 6.02 (bs, 1H), 4.03 (m, 1H), 3.66 (m, 1H), 3.43 (m, 1H) 2.19 (s, 3H), 1.89 (bs, 1H), 1.29 (d, 3H, J = 4.07); ^{13}**C NMR** (125 MHz, $CDCl_3$) δ_C 183.8, 182.6, 146.6, 134.4, 133.4, 132.1, 130.5, 126.3, 126.1, 113.1, 67.5, 52.3, 21.2, 11.5; **HRMS (ESI):** calc for $C_{14}H_{16}NO_3^+$ 246.1125, found $[M+H]^+$ 246.1126, Δ0.4.

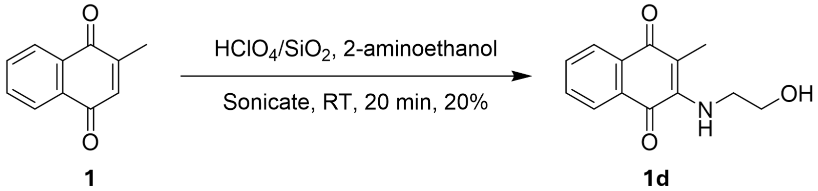

Preparation of 2-aminoethanol-menadione (1d): Menadione (**1**) (0.25 mmol, 43 mg) and $HClO_4$ on SiO_2 (0.025 mmol, 5 mg), was dissolved in 3-aminoethanol (2.5 mmol, 0.15 mL). The mixture was sonicated for 20 min, then heated to 60 °C in an oil bath for 2 min. The reaction was vacuum filtered and extracted biphasically with EtOAc (3 x 20 mL) and ddH_2O. The organic layers were collected, combined, and concentrated under vacuum. Purification of the crude material was carried out via reverse phase prep HPLC using a gradient of MeCN + 0.1% FA/H_2O + 0.1% FA, starting at 35% MeCN + 0.1% FA and ramping to 65% MeCN + 0.1% FA over 24 min at a flow rate of 10 mL/min, which yielded an amorphous dark red solid (11 mg, 20%); ^{1}**H NMR** (500 MHz, $CDCl_3$) δ_H 8.09 (dd,

1H, J = 7.7, 1.1), 8.00 (dd, 1H, J = 7.7, 1.2), 7.68 (td, 1H, J = 7.6, 1.3), 7.58 (td, 1H, J = 7.6, 1.3), 5.96 (bs, 1H), 3.86 (t, 2H, J = 5.4), 3.74 (q, 2H, J = 5.4), 2.22 (s, 3H); ^{13}**C NMR** (125 MHz, $CDCl_3$) δ_C 183.6, 182.4, 146.3, 134.2, 133.2, 131.9, 130.3, 126.1, 126.0, 113.2, 62.0, 47.1, 11.3; **HRMS (ESI):** calc for $C_{13}H_{14}NO_3^+$ 232.0968, found $[M+H]^+$ 232.0978, Δ4.3.

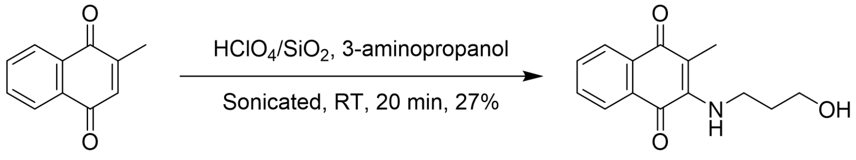

1 **1e**

Preparation of 3-aminopropanol-menadione (1e): Menadione (**1**) (0.25 mmol, 43 mg) and $HClO_4$ on SiO_2 (0.025 mmol, 5.0 mg) was dissolved in 3-aminopropanol (2.5 mmol, 0.19 mL). The mixture was sonicated for 20 min, then heated to 60 ℃ in an oil bath for 2 min. The reaction was vacuum filtered and extracted biphasically with EtOAc (3 x 20 mL) and ddH_2O. The organic layers were collected, combined, and concentrated under vacuum. Purification of the crude material was carried out via reverse phase prep HPLC using a gradient of MeCN + 0.1% FA/H_2O + 0.1% FA, starting at 35% MeCN + 0.1% FA and ramping to 65% MeCN + 0.1% FA over 24 min at a flow rate of 10 mL/min, which yielded an dark red oil (17 mg, 27%); ^{1}**H NMR** (500 MHz, $CDCl_3$) δ_H 8.07 (dt, 1H, J = 7.7, 0.60), 7.98 (dt, 1H, J = 7.6, 0.60), 7.67 (td, 1H, J = 7.6, 1.2), 7.57 (td, 1H, J = 7.7, 1.2), 5.95 (bs, 1H), 3.83 (t, 2H, J = 5.9), 3.74 (q, 2H, J = 6.2), 2.24 (s, 3H), 1.90 (quin, 2H, J = 6.2), 1.62 (bs, 1H); ^{13}**C NMR** (125 MHz, $CDCl_3$) δ_C 183.7, 182.8, 146.5, 134.4, 132.0, 130.5, 126.3, 126.1, 121.5, 112.7, 60.7, 43.2, 33.2, 11.3; **HRMS (ESI):** calc for $C_{14}H_{16}NO_3^+$ 246.1125, found $[M+H]^+$ 246.1124, Δ0.4.

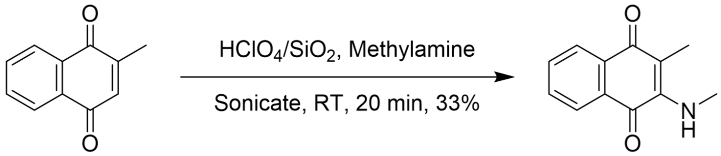

1 **1f**

Preparation of 3-Methylamine-menadione (1f): Menadione (**1**) (2.5 mmol, 0.43 g) and $HClO_4$ on SiO_2 (0.3 mmol, 5.0 mg) was dissolved in methylamine (25 mmol, 0.78 mL). The reaction mixture was sonicated for 20 min, then heated to 60 ℃ in an oil bath for 2 min. The mixture was vacuum filtered, rinsed with EtOAc, and concentrated under vacuum. The crude material was resuspended in 1:1 MeCN:H_2O and purified by reverse phase prep HPLC using the following gradient: holding 35% MeCN + 0.1% FA/65% H_2O + 0.1% FA for 1 min followed by a linear gradient to 65% MeCN + 0.1% FA/35% H_2O + 0.1% FA over 24 min all at a flow rate 10 mL/min to yield an amorphous dark red solid (170 mg, 33%); ^{1}**H NMR** (400 MHz, $CDCl_3$) δ_H 8.08 (d, 1H, J = 7.5), 7.98 (d, 1H, J = 7.5), 7.67 (d, 1H, J = 7.9), 7.57 (t, 1H, J = 7.9) 3.25 (d, 3H, J = 6.3), 2.29 (s, 3H); ^{13}**C NMR** (125 MHz, $CDCl_3$) δ_C 184.0, 182.8, 147.3, 133.9, 133.6, 132.1, 130.6, 126.5, 126.3, 112.3, 33.3, 11.3; **HRMS (ESI):** calc for $C_{12}H_{12}O_2N^+$ 202.0863, found $[M+H]^+$ 202.0860, Δ1.5.

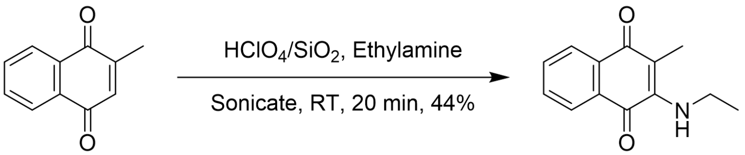

1 **1g**

Preparation of 3-Ethylamine-menadione (1g): Menadione (**1**) (2.5 mmol, 0.430 g) and $HClO_4$ on SiO_2 was dissolved in ethylamine (25 mmol, 1.63 mL). The reaction mixture was sonicated for 20 min, then heated to 60 ℃ in an oil bath for 2 min. The mixture was quenched with the addition of 100 mL DI H_2O, and extracted with EtOAc (3x100 mL). The organic layers were combined and washed with brine (3x75mL) dried with Na_2SO_4 and then

concentrated under vacuum. The crude material was resuspended in 1:1 MeCN:H_2O and purified by reverse phase prep HPLC using the following gradient: holding 35% MeCN + 0.1% formic acid (FA)/65% H_2O + 0.1% FA for 1 min followed by a linear gradient to 65% MeCN + 0.1% FA/35% H_2O + 0.1% FA over 24 min all at a flow rate 10 mL/min to yield an amorphous bright orange solid (0.213 g, 44%); **^{1}H NMR** (400 MHz, $CDCl_3$) δ_H 8.09 (d, 1H, J = 8.3), 7.98 (d, 1H, J = 8.3), 7.67 (td, 1H, J = 7.8, 1.3), 7.57 (td, 1H, J = 7.8, 1.3) 3.61 (qu, 2H, J = 13.6, 7.1), 2.24 (s, 3H), 1.29 (t, 3H, J = 7.1); **^{13}C NMR** (125 MHz, $CDCl_3$) δ_C 183.7, 182.6, 146.1, 134.3, 133.5, 131.8, 130.3, 126.2, 126.0, 112.0, 40.3, 16.2, 11.1; **HRMS (ESI):** calc for $C_{13}H_{14}O_2N^+$ 216.1019, found $[M+H]^+$ 216.1016, Δ1.4.

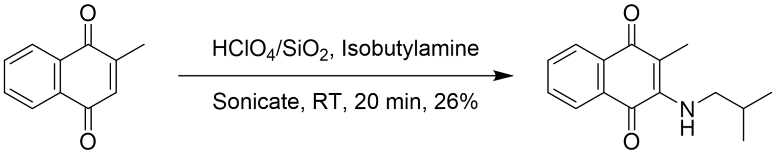

1 **1h**

Preparation of 3-Isobutylamine-menadione (1h): Menadione (**1**) (0.25 mmol, 43 mg) and $HClO_4$ on SiO_2 (0.025 mmol, 5.0 mg), was dissolved in isobutylamine (2.5 mmol, 0.19 mL). The mixture was sonicated for 20 min, then heated to 60 ℃ in an oil bath for 2 min. The reaction was vacuum filtered and extracted biphasically with EtOAc (3 x 20 mL) and ddH_2O. The organic layers were collected, combined, and concentrated under vacuum. Purification of the crude material was carried out via reverse phase prep HPLC using a gradient of MeCN + 0.1% FA/H_2O + 0.1% FA, starting at 35% MeCN + 0.1% FA and ramping to 100% MeCN + 0.1% FA over 26 min at a flow rate of 10 mL/min, which yielded an amorphous dark red solid (16 mg, 26%); **^{1}H NMR** (500 MHz, $CDCl_3$) δ_H 8.09 (dd, 1H, J = 7.8, 1.3), 7.99 (dd, 1H, J = 7.8, 1.2), 7.68 (td, 1H, J = 7.6, 1.2), 7.57 (td, 1H, J = 7.6, 1.3), 5.83 (bs, 1H), 3.35 (t, 2H, J = 6.4), 2.23 (s, 3H), 1.86 (sept, 1H, J = 6.8), 0.99 (d, 6H, J = 6.8); **^{13}C NMR** (125 MHz, $CDCl_3$) δ_C 183.8, 182.7, 146.4, 134.5, 133.7, 131.9, 130.4, 126.3, 126.1, 112.2, 53.1, 29.9, 20.2, 11.5; **HRMS (ESI):** calc for $C_{15}H_{18}NO_2^+$ 244.1332, found $[M+H]^+$ 244.1339, Δ2.9.

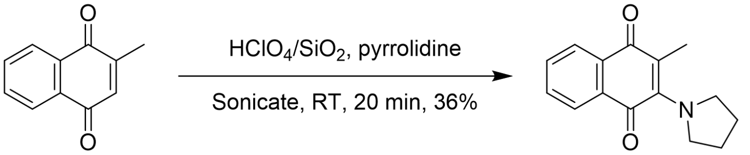

1 **1i**

Preparation of 3-Pyrrolidine-menadione (1i): Menadione (**1**) (0.25 mmol, 43 mg) and $HClO_4$ on SiO_2 (0.025 mmol, 5.0 mg) was dissolved in pyrrolidine (2.5 mmol, 0.21 mL). The mixture was sonicated for 20 min, then heated to 60 ℃ in an oil bath for 2 min. The reaction was vacuum filtered and extracted biphasically with EtOAc (3 x 20 mL) and ddH_2O. The organic layers were collected, combined, and concentrated under vacuum. Purification of the crude material was carried out via reverse phase prep HPLC using a gradient of MeCN + 0.1% FA/H_2O + 0.1% FA, starting at 35% MeCN + 0.1% FA and ramping to 100% MeCN + 0.1% FA over 24 min at a flow rate of 10 mL/min, which yielded an amorphous red solid (25 mg, 36%); **^{1}H NMR** [500 MHz,$(CD_3)_2SO$] δ_H 7.87 (dd, 1H, J = 7.8, 1.0), 7.82 (dd, 1H, J = 7.5, 1.1), 7.73 (td, 1H, J = 7.5, 1.3), 7.65 (td, 1H, J = 7.4, 1.3), 3.71 (t, 4H, J = 6.6), 2.08 (s, 3H), 1.87-1.81 (m, 4H); **^{13}C NMR** (125 MHz, $CDCl_3$) δ 183.8, 182.6, 141.8, 135.0, 133.1, 132.1, 127.4, 126.4, 126.2, 111.6, 47.6, 26.2, 11.3; **HRMS (ESI):** calc for $C_{15}H_{16}NO_2^+$ 242.1176, found $[M+H]^+$ 242.1174, Δ0.8.

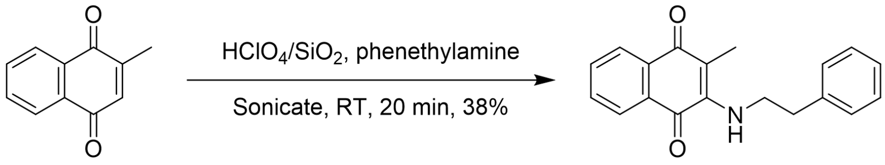

1 → 1j

Preparation of 3-Phenethylamine-menadione (1j): Menadione (**1**) (0.25 mmol, 43 mg) and $HClO_4$ on SiO_2 (0.025 mmol, 5.0 mg) was dissolved in phenethylamine (2.5 mmol, 0.19 mL). The mixture was sonicated for 20 min, then heated to 60 °C in an oil bath for 2 min. The reaction was vacuum filtered and extracted biphasically with EtOAc (3 x 20 mL) and ddH_2O. The organic layers were collected, combined, and concentrated under vacuum. Purification of the crude material was carried out via reverse phase prep HPLC using a gradient of MeCN + 0.1% FA/H_2O + 0.1% FA, starting at 35% MeCN + 0.1% FA and ramping to 100% MeCN + 0.1% FA over 24 min at a flow rate of 10 mL/min, which yielded an amorphous red solid (65 mg, 38%); ^{1}**H NMR** (500 MHz, $CDCl_3$) δ_H 8.09 (dd, 1H, J = 7.7, 0.95), 7.98 (dd, 1H, J = 7.7, 1.1), 7.67 (td, 1H, J = 7.6, 1.3), 7.57 (td, 1H, J = 7.6, 1.3), 7.33 (t, 2H, J = 7.6), 7.23 (t, 2H, J = 7.6), 7.23 (s, 1H), 5.75 (bs, 1H), 3.83 (q, 2H, J = 6.9), 2.93 (t, 2H, J = 7.1), 2.22 (s, 3H); ^{13}**C NMR** (125 MHz, $CDCl_3$) δ_C 183.7, 182.6, 146.2, 138.1, 134.4, 133.6, 132.0, 130.5, 129.0, 128.9, 127.0, 126.3, 126.1, 112.9, 46.8, 37.4, 11.4; **HRMS (ESI):** calc for $C_{19}H_{18}NO_2^+$ 292.1332, found $[M+H]^+$ 292.1335, Δ1.0.

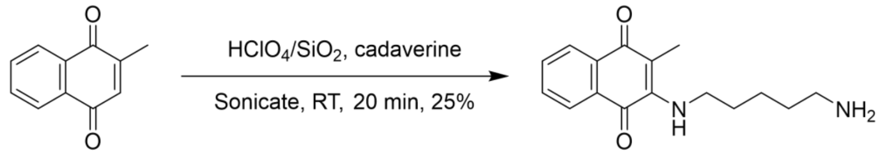

1 → 1k

Preparation of 3-Cadaverine-menadione (1k): Menadione (**1**) (0.25 mmol, 43 mg) and $HClO_4$ on SiO_2 (0.025 mmol, 5.0 mg) was dissolved in cadaverine (2.5 mmol, 0.29 mL). The mixture was sonicated for 20 min, then heated to 60 °C in an oil bath for 2 min. The reaction was vacuum filtered and extracted biphasically with EtOAc (3 x 20 mL) and ddH_2O. The organic layers were collected, combined, and concentrated under vacuum. Purification of the crude material was carried out via reverse phase prep HPLC using a gradient of MeCN + 0.1% FA/H_2O + 0.1% FA, starting at 35% MeCN + 0.1% FA and ramping to 100% MeCN + 0.1% FA over 24 min at a flow rate of 10 mL/min, which yielded an amorphous red solid (17 mg, 25%); ^{1}**H NMR** (500 MHz, $CDCl_3$) δ_H 8.10 (dd, 1H, J = 7.8, 0.95), 7.98 (dd, 1H, J = 7.7, 0.84), 7.68 (td, 1H, J = 7.7, 1.3), 7.58 (td, 1H, J = 7.6, 1.3), 5.68 (m, 1H), 5.43 (m, 1H), 3.56 (q, 2H, J = 6.5), 3.27 (q, 2H, J = 6.5), 2.23 (s, 3H), 1.98 (s, 2H), 1.25 (s, 2H); ^{13}**C NMR** (125 MHz, CD_3OD) δ_C 184.7, 183.5, 148.4, 135.4, 133.2, 129.1, 127.4, 127.0, 126.8, 112.3, 45.9, 40.1, 31.6, 29.9, 24.8, 22.6; **HRMS (ESI):** calc for $C_{16}H_{21}N_2O_2^+$ 273.1598, found $[M+H]^+$ 273.1608, Δ3.7.

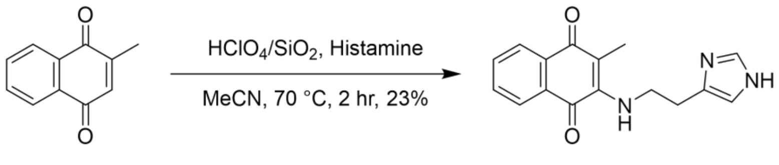

1 → 1l

Preparation of 3-Histamine-menadione (1l): Menadione (**1**) (0.25 mmol, 43 mg) and $HClO_4$ on SiO_2 (0.025 mmol, 5.0 mg), and histamine (2.5 mmol, 280 mg) was dissolved in MeCN (3 mL). The mixture was heated to 70 °C in an oil bath for 2 h with magnetic stirring. The reaction was vacuum filtered and extracted biphasically with EtOAc (3 x 20 mL) and ddH_2O. The organic layers were collected, combined, and concentrated under vacuum. Purification of the crude material was carried out via reverse phase prep HPLC using a gradient of MeCN + 0.1% FA/H_2O + 0.1% FA, starting at 35% MeCN + 0.1% FA and ramping to 65% MeCN + 0.1% FA over 26 min at a flow rate of 10 mL/min, which yielded an amorphous dark red solid (16 mg, 23%); ^{1}**H NMR** (500 MHz, CD_3OD) δ_H 8.30 (bs, 1H), 8.11 (s, 1H), 7.88 (d, 1H, J = 7.7), 7.84 (d, 1H, J = 7.1), 7.62 (t, 1H, J = 7.7), 7.54 (t, 1H, J = 7.1), 7.05 (s, 1H) 3.81 (t, 2H, J = 6.7), 2.89 (t, 2H, J = 6.7), 2.03 (s, 3H); ^{13}**C NMR** (125 MHz, CD_3OD) δ_C 184.7, 183.4,

148.5, 135.4, 134.4, 133.3, 132.0, 127.0, 126.8, 117.9, 113.4, 62.3, 45.4, 32.2, 28.3, 10.9; **HRMS (ESI):** calc for $C_{16}H_{16}N_3O_2^+$ 282.1237, found $[M+H]^+$ 282.1246, Δ3.2.

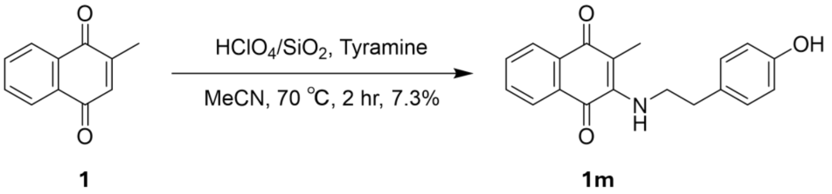

Preparation of 3-Tyramine-menadione (1m): Menadione (**1**) (0.25 mmol, 43 mg) and $HClO_4$ on SiO_2 (0.025 mmol, 5.0 mg), and tyramine (2.5 mmol, 342.7 mg) was dissolved in MeCN (3 mL). The mixture was heated to 70 ℃ in an oil bath for 2 h with magnetic stirring. The reaction was vacuum filtered and extracted biphasically with EtOAc (3 x 20 mL) and ddH_2O. The organic layers were collected, combined, and concentrated under vacuum. Purification of the crude material was carried out via reverse phase prep HPLC using a gradient of MeCN/water (modified with 0.1% FA), starting at 45% MeCN and ramping to 100% MeCN over 26 min at a flow rate of 10 mL/min, which yielded a dark red oil (5.6 mg, 7.3%); **^{1}H NMR** (500 MHz, $CDCl_3$) δ_H 8.09 (dd, 1H, J = 7.7, 1.3), 7.99 (dd, 1H, J = 7.6, 1.3), 7.67 (td, 1H, J = 7.7, 1.3), 7.57 (td, 1H, J = 7.6, 1.3), 7.10 (dt, 2H, J = 8.6, 3.1), 6.79 (dt, 2H, J = 8.6, 3.0), 5.72 (bs, 1H), 4.66 (bs, 1H), 3.77 (q, 2H, J = 7.0), 2.86 (t, 2H, J = 7.2), 2.22 (s, 3H); **^{13}C NMR** (125 MHz, $CDCl_3$) δ_C 183.8, 182.6, 154.6, 146.3, 134.5, 133.6, 132.0, 130.5, 130.2, 130.1, 126.4, 126.1, 123.9, 122.6, 115.8, 112.8, 47.0, 36.5, 11.4; **HRMS (ESI):** calc for $C_{19}H_{18}NO_3^+$ 308.1281, found $[M+H]^+$ 308.1283, Δ0.6.

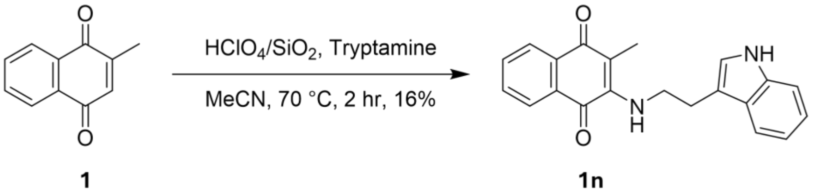

Preparation of 3-Tryptamine-menadione (1n): Menadione (**1**) (0.25 mmol, 43 mg) and $HClO_4$ on SiO_2 (0.025 mmol, 5.0 mg), and tryptamine (2.5 mmol, 401 mg) was dissolved in MeCN (3 mL). The mixture was heated to 70 ℃ in an oil bath for 2 h with magnetic stirring. The reaction was vacuum filtered and extracted biphasically with EtOAc (3 x 20 mL) and ddH_2O. The organic layers were collected, combined, and concentrated under vacuum. Purification of the crude material was carried out via reverse phase prep HPLC using a gradient of MeCN + 0.1% FA/H_2O + 0.1% FA, starting at 35% MeCN + 0.1% FA and ramping to 65% MeCN 0.1% FA over 26 min at a flow rate of 10 mL/min, which yielded an amorphous dark red solid (13 mg, 16%); **^{1}H NMR** (500 MHz, $CDCl_3$) δ_H 8.07 (d, 1H, J = 7.5), 7.96 (d, 1H, J = 7.5), 7.65 (t, 1H, J = 7.7), 7.60 (d, 1H, J = 8.1), 7.56 (t, 1H, J = 7.7), 7.38 (d, 1H, J = 8.1), 7.22 (t, 1H, J = 7.9), 7.14 (t, 1H, J = 7.42), 7.10 (d, 1H, J = 2.25), 5.83 (bs, 1H), 3.90 (q, 2H, J = 6.62), 3.12 (t, 2H, J = 6.83) 2.23 (s, 3H); **^{13}C NMR** (125 MHz, $CDCl_3$) δ_C 183.7, 182.7, 146.3, 136.6, 134.4, 133.6, 131.9, 130.5, 127.2, 126.3, 126.1, 122.6, 122.5, 119.8, 118.7, 112.5, 112.3, 111.5, 45.5, 26.9, 11.4; **HRMS (ESI):** calc for $C_{21}H_{19}N_2O_2^+$ 331.1441, found $[M+H]^+$ 331.1445, Δ1.2.

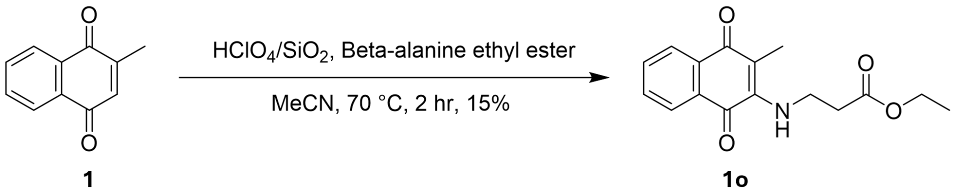

Preparation of 3-*β*-alanine-ethyl ester-menadione (1o): Menadione (**1**) (0.25 mmol, 43 mg) and $HClO_4$ on SiO_2 (0.025 mmol, 5.0 mg), and *β*-alanine-ethyl ester (2.5 mmol, 290 mg) was dissolved in MeCN (3 mL). Et_3N was added dropwise until the solution turned from a bright yellow to an orange color, indicating a relatively basic solution. The mixture was heated to 70 ℃ in an oil bath for 2 h with magnetic stirring. The reaction was vacuum

filtered and extracted biphasically with EtOAc (3 x 20 mL) and ddH_2O. The organic layers were collected, combined, and concentrated under vacuum. Purification of the crude material was carried out via reverse phase prep HPLC using a gradient of MeCN 0.1% FA/H_2O + 0.1% FA, starting at 35% MeCN + 0.1% FA and ramping to 65% MeCN + 0.1% over 26 min at a flow rate of 10 mL/min, which yielded a dark red oil (13 mg, 15%); ^{1}**H NMR** (500 MHz, $CDCl_3$) δ_H 8.07 (dd, 1H, J = 7.7, 1.2), 7.99 (dd, 1H, J = 7.7, 1.3), 7.67 (td, 1H, J = 7.6, 1.3), 7.57 (td, 1H, J = 7.6, 1.2), 5.92 (bs, 1H), 4.18 (q, 2H, J = 7.2), 3.85 (t, 2H, J = 6.5), 2.63 (t, 2H, J = 6.5), 2.21 (s, 3H), 1.27 (t, 3H, J = 7.2); ^{13}**C NMR** (125 MHz, $CDCl_3$) δ_C 183.7, 182.5, 171.6 146.1, 134.4, 133.4, 132.1, 130.5, 126.3, 126.2, 116.5, 61.2, 41.0, 35.6, 14.3, 11.3; **HRMS (ESI):** calc for $C_{16}H_{18}NO_4^+$ 288.1231, found $[M+H]^+$ 288.1232, Δ0.3.

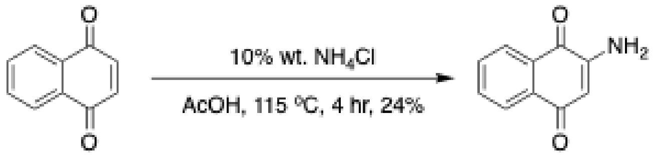

2 **2a**

Preparation of 3-Amino-naphthoquinone (2a): Naphthoquinone (0.19 g, 1.2 mmol) was added to a round bottom flask and dissolved in a 10% w/v solution of ammonium acetate in glacial acetic acid (1.05 g NH_4OAc in 10.5 mL HOAc). The solution was heated under reflux (115 ℃) for six hours. The reaction mixture was let cool to room temperature, quenched with cold DI H_2O, 50 mL, and extracted with $CHCl_3$ (3x50 mL). The organic layers were combined, washed with saturated $NaHCO_3$ solution (3x 50mL) and dried with Na_2SO_4, then concentrated via rotary evaporation. Purification of the crude material was carried out via reverse phase preparative HPLC using a gradient of MeCN/water at a flow rate of 10 mL/min, starting at 25% MeCN/75% water then ramping to 100% MeCN over 24 minutes, which yielded an amorphous orange solid (0.05 g, 24%). ^{1}**H NMR** (500 MHz, $CDCl_3$) δ_H 8.09 (d, 1H, J = 7.42), 8.05 (d, 1H, J = 7.42) 7.73 (t, 1H, J = 7.42), 7.64 (t, 1H, J = 7.42) 6.00 (s, 1H), 5.14 (bs, 2H); ^{13}**C NMR** (125 MHz, $CDCl_3$) δ_C 183.9, 182.0, 148.4, 134.8, 132.4, 131.8, 127.8, 126.4, 126.3, 105.3; **HRMS (ESI)** calc for $C_{10}H_8O_2N^+$ 174.0555, found: $[M+H]^+$ 174.0550 *m/z*, Δ2.9.

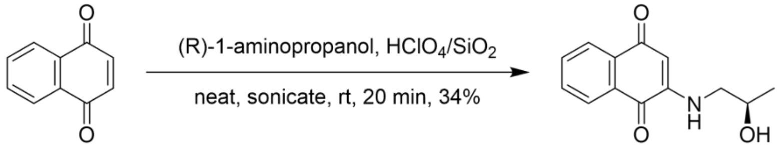

2 **2c**

Preparation of 2-[(*R*)-aminopropan-2-ol]-naphthoquinone (2c): Naphthoquinone (**2**) (0.6 mmol, 100 mg) and $HClO_4$ on SiO_2 (0.060 mmol, 9.6 mg) was dissolved in (*R*)-1-aminopropanol (2 mL). The mixture was sonicated for 20 min, then heated to 60 ℃ in an oil bath for 2 min. The reaction was vacuum filtered and extracted with EtOAc (3 x 20 mL) and ddH_2O. The organic layers were combined and concentrated under vacuum. Purification of the crude material was carried out via normal phase flash chromatography using a gradient of methanol (MeOH) + 0.1% FA/dichloromethane (DCM) + 0.1% FA at a flow rate of 25 mL/min, starting at 10% MeOH + 0.1% FA/90% DCM + 0.1% FA for 1 column volumes then ramping to 15% MeOH + 0.1% FA/85% DCM + 0.1% FA over 8 column volumes, which yielded an amorphous red solid (47 mg, 34%); **Optical Rotation** $[\alpha]_D^{20}$ +2.16 (c 0.013, MeOH); ^{1}**H NMR** (500 MHz, $CDCl_3$) δ_H 8.09 (dd, 1H, J = 7.7, 1.3), 8.05 (dd, 1H, J = 7.7, 1.4), 7.73 (td, 1H, J = 7.6, 1.3), 7.62 (td, 1H, J = 7.6, 1.4), 6.24 (bs, 1H), 5.75 (s, 1H), 4.15 (m, 1H), 3.26 (m, 1H), 3.13 (m, 1H), 1.32 (d, 3H, J = 6.3); ^{13}**C NMR** (125 MHz, $CDCl_3$) δ_C 184.4, 183.2, 148.5, 134.9, 132.2, 130.7, 126.5, 126.3, 125.4, 101.3, 65.9, 49.6, 21.5; **HRMS (ESI):** calc for $C_{13}H_{14}NO_3^+$ 232.0968, found $[M+H]^+$ 232.0970, Δ0.9.

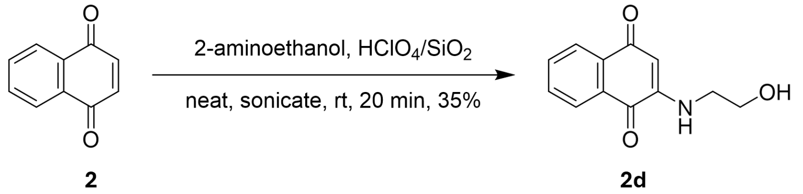

Preparation of 2-aminoethanol-naphthoquinone (2d) is in bold below.

Preparation of 2-aminoethanol-naphthoquinone (2d): Naphthoquinone (**2**) (0.6 mmol, 100 mg) and $HClO_4$ on SiO_2 (0.060 mmol, 9.6 mg) was dissolved in 2-aminoethanol (2 mL). The mixture was sonicated for 20 min, then heated to 60 ℃ in an oil bath for 2 min. The reaction was vacuum filtered and extracted biphasically with EtOAc (3 x 20 mL) and ddH_2O. The organic layers were collected, combined, and concentrated under vacuum. Purification of the crude material was carried out via normal phase flash chromatography using a gradient of methanol (MeOH) + 0.1% FA/dichloromethane (DCM) + 0.1% FA at a flow rate of 25 mL/min, starting at 10% MeOH + 0.1% FA/90% DCM + 0.1% FA for 1 column volumes then ramping to 20% MeOH + 0.1% FA/80% DCM + 0.1% FA over 8 column volumes, which yielded an amorphous red solid (45 mg, 35%); ^{1}**H NMR** (500 MHz, $CDCl_3$) δ_H 8.09 (dd, 1H, J = 7.6, 1.2), 8.05 (dd, 1H, J = 7.7, 1.1), 7.73 (td, 1H, J = 7.6, 1.2), 7.62 (td, 1H, J = 7.6, 1.1), 6.21 (bs, 1H), 5.78 (s, 1H), 3.93 (t, 2H, J = 5.0), 3.38 (q, 2H, J = 5.0), 1.25 (s, 1H); ^{13}**C NMR** (125 MHz, $CDCl_3$) δ_C 185.4, 181.9, 148.3, 134.9, 132.2, 130.7, 127.0, 126.5, 126.4, 101.3, 60.2, 44.6; **HRMS (ESI):** calc for $C_{12}H_{12}NO_3^+$ 218.0812, found $[M+H]^+$ 218.0815, Δ1.4.

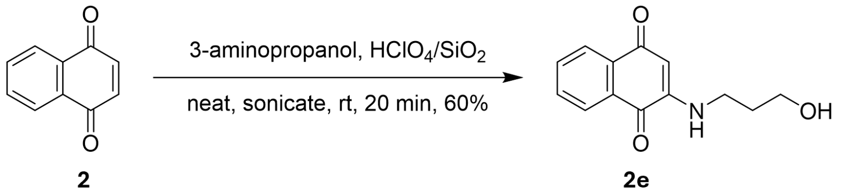

Preparation of 2-aminopropanol-naphthoquinone (2e): Naphthoquinone (**2**) (0.6 mmol, 100 mg) and $HClO_4$ on SiO_2 (0.06 mmol, 9.6 mg) was dissolved in 3-aminopropanol (2 mL). The mixture was sonicated for 20 min, then heated to 60 ℃ in an oil bath for 2 min. The reaction was vacuum filtered and extracted biphasically with EtOAc (3 x 20 mL) and ddH_2O. The organic layers were combined, and concentrated under vacuum. Purification of the crude material was carried out via normal phase flash chromatography using a gradient of methanol (MeOH) + 0.1% FA/dichloromethane (DCM) + 0.1% FA at a flow rate of 25 mL/min, starting at 10% MeOH + 0.1% FA/90% DCM + 0.1% FA for 1 column volumes then ramping to 15% MeOH + 0.1% FA/85% DCM + 0.1% FA over 8 column volumes, which yielded an amorphous red solid (84 mg, 60%); ^{1}**H NMR** (500 MHz, $CDCl_3$) δ_H 8.09 (dd, 1H, J = 7.7, 1.0), 8.02 (dd, 1H, J = 7.7, 1.1), 7.71 (td, 1H, J = 7.6, 1.3), 7.60 (td, 1H, J = 7.5, 1.3), 6.32 (bs, 1H), 5.75 (s, 1H), 3.84 (t, 2H, J = 5.8), 3.36 (q, 2H, J = 6.4), 1.95 (quin, 2H, J = 6.4); ^{13}**C NMR** (125 MHz, $CDCl_3$) δ_C 183.1, 182.1, 148.3, 134.9, 133.9, 132.1, 130.7, 126.4, 126.3, 100.8, 60.9, 40.5, 30.6; **HRMS (ESI):** calc for $C_{13}H_{14}NO_3^+$ 232.0968, found $[M+H]^+$ 232.0972, Δ1.7.

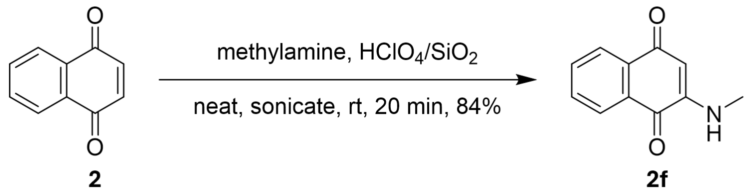

Preparation of 2-methylamine-naphthoquinone (2f): Naphthoquinone (**2**) (0.6 mmol, 100 mg) and $HClO_4$ on SiO_2 (0.060 mmol, 9.6 mg) was dissolved in methylamine (2 mL). The mixture was sonicated for 20 min, then heated to 60 ℃ in an oil bath for 2 min. The reaction was vacuum filtered and extracted biphasically with EtOAc (3 x 20 mL) and ddH_2O. The organic layers were combined, and concentrated under vacuum, yielding an amorphous dark red solid (94 mg, 84%);^{1}**H NMR** (500 MHz, CD_3OD) δ_H 8.08 (d, 1H, J = 6.4), 8.05 (d, 1H, J = 6.8), 7.80 (t, 1H, J = 7.5), 7.70 (t, 1H, J = 7.5), 5.68 (s, 1H), 2.93 (s, 3H); ^{13}**C NMR** (125 MHz, CD_3OD) δ_C 186.2, 185.6, 152.1,

136.0, 135.3, 133.5, 132.3, 127.5, 127.1, 99.9, 29.4; **HRMS (ESI):** calc for $C_{11}H_{10}NO_2^+$ 188.0706, found $[M+H]^+$ 188.0706, Δ0.0.

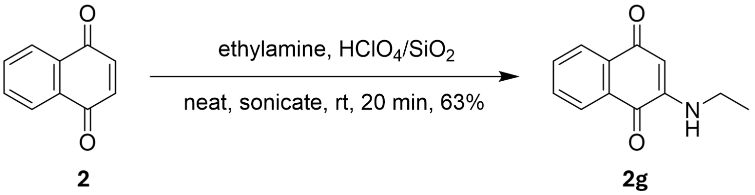

Preparation of 2-ethylamine-naphthoquinone (2g): Naphthoquinone (**2**) (0.6 mmol, 100 mg) and $HClO_4$ on SiO_2 (0.060 mmol, 9.6 mg) was dissolved in ethylamine (2 mL). The mixture was sonicated for 20 min, then heated to 60 °C in an oil bath for 2 min. The reaction was vacuum filtered and extracted biphasically with EtOAc (3 x 20 mL) and ddH_2O. The organic layers were combined, and concentrated under vacuum. The product was purified via normal phase flash chromatography using a gradient of methanol (MeOH) + 0.1% FA/dichloromethane (DCM) + 0.1% FA at a flow rate of 25 mL/min, starting at 10% MeOH + 0.1% FA/90% DCM + 0.1% FA for 1 column volumes then ramping to 15% MeOH + 0.1% FA/85% DCM + 0.1% FA over 8 column volumes, yielding an amorphous dark red solid (76 mg, 63%); ^{1}**H NMR** (500 MHz, $CDCl_3$) δ_H 8.11 (dd, 1H, J = 7.7, 0.98), 8.05 (dd, 1H, J = 7.7, 1.3), 7.71 (td, 1H, J = 7.6, 1.3), 7.61 (td, 1H, J = 7.6, 1.2), 5.83 (bs, 1H), 5.73 (s, 1H), 3.23 (m, 2H), 1.34 (t, 3H, J = 7.3); ^{13}**C NMR** (125 MHz, $CDCl_3$) δ_C 183.1, 182.1, 148.0, 134.9, 133.9, 132.1, 130.7, 126.4, 126.4, 100.9, 37.4, 13.7; **HRMS (ESI):** calc for $C_{12}H_{12}NO_2^+$ 202.0863, found $[M+H]^+$ 202.0867, Δ2.0.

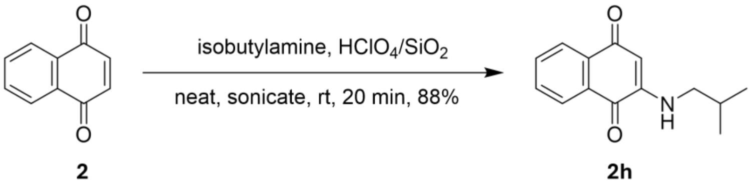

Preparation of 2-isobutylamine-naphthoquinone (2h): Naphthoquinone (**2**) (1.5 mmol, 240 mg) and $HClO_4$ on SiO_2 (0.15 mmol, 24 mg) was dissolved in isobutylamine (2 mL). The mixture was sonicated for 20 min, then heated to 60 °C an oil bath for 2 min. The reaction was vacuum filtered and extracted biphasically with EtOAc (3 x 20 mL) and ddH_2O. The organic layers were combined, and concentrated under vacuum, yielding an amorphous red solid (301 mg, 88%); ^{1}**H NMR** (500 MHz, $CDCl_3$) δ_H 8.09 (dd, 1H, J = 7.7, 1.2), 8.05 (dd, 1H, J = 7.7, 1.2) 7.73 (td, 1H, J = 7.7, 1.3), 7.61 (td, 1H, J = 7.6, 1.3), 5.98 (bs, 1H), 5.74 (s, 1H), 3.01 (t, 2H, J = 6.3), 2.01 (hept, 1H, J = 6.6), 1.01 (d, 6H, J = 6.7); ^{13}**C NMR** (125 MHz, $CDCl_3$) δ_C 182.9, 181.9, 148.0, 134.7, 133.6, 131.8, 130.4, 126.2, 126.1, 100.7, 50.0, 27.5, 20.2; **HRMS (ESI):** calc for $C_{14}H_{16}NO_2^+$ 230.1176, found $[M+H]^+$ 230.1179, Δ1.3.

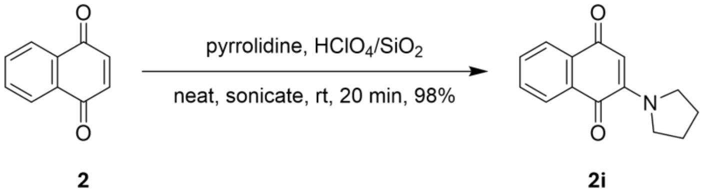

Preparation of 2-pyrrolidine-naphthoquinone (2i): Naphthoquinone (**2**) (0.6 mmol, 100 mg) and $HClO_4$ on SiO_2 (0.060 mmol, 9.6 mg) was dissolved in pyrrolidine (2 mL). The mixture was sonicated for 20 min, then heated to 60 °C in an oil bath for 2 min. The reaction was vacuum filtered and extracted biphasically with EtOAc (3 x 20 mL) and ddH_2O. The organic layers were combined, and concentrated under vacuum, yielding an amorphous bright red solid (130 mg, 98%); ^{1}**H NMR** (500 MHz, $CDCl_3$) δ_H 8.05 (dd, 1H, J = 7.7, 1.0), 7.99 (dd, 1H, J = 7.7, 1.0), 7.69 (td, 1H, J = 7.6, 1.2), 7.59 (td, 1H, J = 7.6, 1.2), 5.74 (s, 1H), 3.11-4.22 (m, 4H), 2.00 (m, 4H); ^{13}**C NMR** (125 MHz,

CDCl3) δC 183.5, 182.5, 149.2, 134.1, 133.4, 132.0, 131.8, 126.5, 125.6, 105.1, 99.4, 51.1; **HRMS (ESI)** calc for $C_{14}H_{14}NO_2^+$ 228.1019, found $[M+H]^+$ 228.1019, Δ0.0.

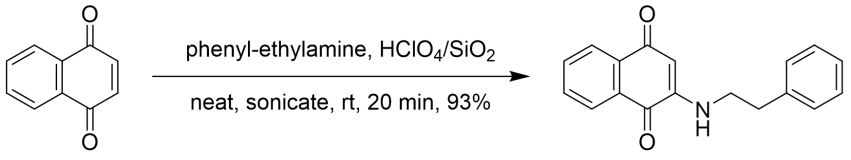

2 **2j**

Preparation of 2-phenyl-ethylamine-naphthoquinone (2j): Naphthoquinone (**2**) (0.6 mmol, 100 mg) and $HClO_4$ on SiO_2 (0.060 mmol, 9.6 mg) was dissolved in phenyl-ethylamine (2 mL). The mixture was sonicated for 20 min, then heated to 60 ℃ in an oil bath for 2 min. The reaction was vacuum filtered and extracted biphasically with EtOAc (3 x 20 mL) and ddH_2O. The organic layers were combined, and concentrated under vacuum. Purification of the crude material was carried out via normal phase flash chromatography using a gradient of methanol (MeOH) + 0.1% FA/dichloromethane (DCM) + 0.1% FA at a flow rate of 25 mL/min, starting at 10% MeOH + 0.1% FA/90% DCM + 0.1% FA for 1 column volumes then ramping to 15% MeOH + 0.1% FA/85% DCM + 0.1% FA over 8 column volumes, yielding an amorphous red solid (160 mg, 93%); ^{1}**H NMR** (500 MHz, $CDCl_3$) δ_H 8.10 (dd, 1H, J = 7.7, 1.0), 8.02 (dd, 1H, J = 7.7, 1.0), 7.72 (td, 1H, J = 7.6, 1.2), 7.61 (td, 1H, J = 7.6, 1.2), 7.34 (t, 2H, J = 7.5), 7.27 (t, 1H, J = 7.3), 7.23 (d, 2H, J = 7.1), 5.94 (bs, 1H), 5.79 (s, 1H), 3.47 (q, 2H, J = 6.3), 2.98 (t, 2H, J = 7.0); ^{13}**C NMR** (125 MHz, $CDCl_3$) δ_C 183.2, 181.9, 147.9, 138.0, 134.9, 133.8, 132.1, 130.6, 129.1, 128.9, 128.8, 128.6, 127.1, 126.4, 126.4, 101.2, 43.8, 34.5; **HRMS (ESI):** calc for $C_{18}H_{16}NO_2^+$ 278.1176, found $[M+H]^+$ 278.1182, Δ2.2.

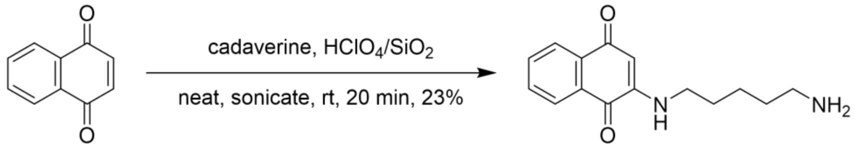

2 **2k**

Preparation of 2-cadaverine-naphthoquinone (2k): naphthoquinone (**2**) (0.6 mmol, 100 mg) and $HClO_4$ on SiO_2 (0.060 mmol, 9.6 mg) was dissolved in cadaverine (2 mL). The mixture was sonicated for 20 min, then heated to 60 ℃ in an oil bath for 2 min. The reaction was vacuum filtered and extracted biphasically with EtOAc (3 x 20 mL) and ddH_2O. The organic layers were combined, and concentrated under vacuum. Purification of the crude material was carried out via normal phase flash chromatography using a gradient of DCM/MeOH (modified with 0.1% FA), starting at 10% MeOH and ramping to 15% MeOH over 8 column volumes. The fraction containing the product was then further purified with reverse phase HPLC using a gradient of MeCN and water, starting at 25% MeCN and ramping to 100% MeCN over 26 min, yielding an amorphous bright red solid (35 mg, 23%); ^{1}**H NMR** (500 MHz, CD_3OD) δ_H 8.06 (dd, 1H, J = 7.7, 1.2), 8.03 (dd, 1H, J = 7.7, 1.3), 7.78 (td, 1H, J = 7.6, 1.4), 7.70 (td, 1H, J = 7.6, 1.3), 5.71 (s, 1H), 3.29 (t, 2H, J = 7.1), 2.94 (t, 2H, J = 7.6), 1.68-1.78 (m, 4H), 1.50 (m, 2H); ^{13}**C NMR** (125 MHz, CD_3OD) δ_C 184.6, 182.4, 150.6, 135.7, 134.8, 133.3, 131.9, 127.2, 126.7, 99.9, 42.9, 40.4, 28.3, 28.1, 24.8; **HRMS (ESI):** calc for $C_{15}H_{19}N_2O_2^+$ 259.1446, found $[M+H]^+$ 259.1448, Δ0.8.

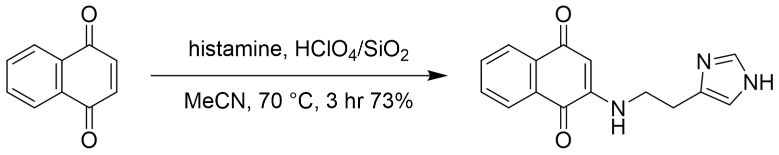

2 **2l**

Preparation of 2-histamine-naphthoquinone (2l): Naphthoquinone (**2**) (0.3 mmol, 50 mg), $HClO_4$ on SiO_2 (0.030 mmol, 4.8 mg), and histamine (3.0 mmol, 330 mg) were dissolved in MeCN (3 mL). The mixture was heated to 70

°C in an oil bath for 3 h with magnetic stirring. The reaction was vacuum filtered and extracted biphasically with EtOAc (3 x 20 mL) and ddH_2O. The organic layers were combined and concentrated under vacuum. Purification of the crude material was carried out via normal phase flash chromatography using a gradient of methanol (MeOH) + 0.1% FA/dichloromethane (DCM) + 0.1% FA at a flow rate of 25 mL/min, starting at 10% MeOH + 0.1% FA/90% DCM + 0.1% FA for 1 column volumes then ramping to 15% MeOH + 0.1% FA/85% DCM + 0.1% FA over 8 column volumes, which yielded an amorphous reddish-brown solid (59 mg, 73%); ^{1}**H NMR** (500 MHz, CD_3OD) δ_H 8.04 (dd, 1H, J = 7.7, 1.2), 8.00 (dd, 1H, J = 7.8, 1.1), 7.77 (td, 1H, J = 7.6, 1.2), 7.67 (td, 1H, J = 7.6, 1.3), 7.62 (d, 1H, J = 1.2), 6.92 (s, 1H), 5.72 (s, 1H), 3.50 (t, 2H, J = 7.0), 2.94 (t, 2H, J = 7.0); ^{13}**C NMR** (125 MHz, CD_3OD) δ_C 184.8, 182.5, 150.6, 136.4, 135.8, 135.0, 133.4, 132.1, 127.3, 126.8, 124.4, 117.5, 100.3, 43.4, 26.7; **HRMS (ESI):** calc for $C_{15}H_{14}N_3O_2^+$ 268.1081, found $[M+H]^+$ 268.1078, Δ1.1.

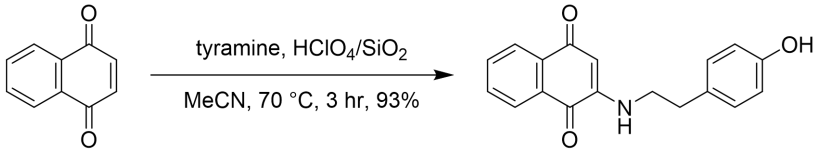

2 **2m**

Preparation of 2-tyramine-naphthoquinone (2m): Naphthoquinone (**2**) (0.6 mmol, 100 mg), $HClO_4$ on SiO_2 (0.060 mmol, 9.6 mg), and tyramine (6.0 mmol, 820 mg) were dissolved in MeCN (3 mL). The mixture was heated to 70 °C in an oil bath for 3 h with magnetic stirring. The reaction was vacuum filtered and extracted biphasically with EtOAc (3 x 20 mL) and ddH_2O. The organic layers were combined, and concentrated under vacuum. Purification of the crude material was carried out via normal phase flash chromatography using a gradient of methanol (MeOH) + 0.1% FA/dichloromethane (DCM) + 0.1% FA at a flow rate of 25 mL/min, starting at 10% MeOH + 0.1% FA/90% DCM + 0.1% FA for 1 column volumes then ramping to 15% MeOH + 0.1% FA/85% DCM + 0.1% FA over 8 column volumes, which yielded an amorphous red solid (160 mg, 93%); ^{1}**H NMR** (500 MHz, CD_3OD) δ_H 8.03 (dd, 1H, J = 7.8, 1.2), 8.01 (dd, 1H, J = 7.8, 1.2) 7.77 (td, 1H, J = 7.6, 1.3), 7.67 (td, 1H, J = 7.5, 1.3), 7.06-7.11 (m, 2H), 6.71-6.75 (m, 2H), 5.71 (s, 1H), 3.43 (t, 2H, J = 7.2), 2.86 (t, 2H, J = 7.4); ^{13}**C NMR** (125 MHz, CD_3OD) δ_C 184.8, 182.5, 157.2, 150.6, 146.1, 145.7, 135.8, 135.0, 133.4, 132.1, 130.8, 130.6, 127.3, 126.8, 116.4, 100.2, 45.2, 34.3; **HRMS (ESI):** calc for $C_{18}H_{16}NO_3^+$ 294.1125, found: $[M+H]^+$ 294.1126, Δ0.3.

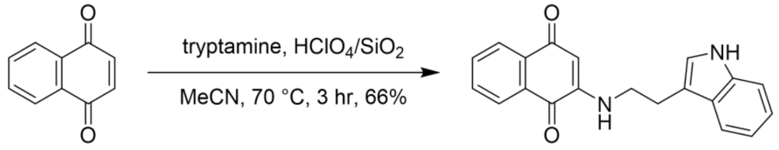

2 **2n**

Preparation of 2-tryptamine-naphthoquinone (2n): Naphthoquinone (**2**) (0.6 mmol, 100 mg), $HClO_4$ on SiO_2 (0.060 mmol, 9.6 mg), and tryptamine (6.0 mmol, 960 mg) were dissolved in MeCN (3 mL). The mixture was heated to 70 °C in an oil bath for 3 h with magnetic stirring. The reaction was vacuum filtered and extracted biphasically with EtOAc (3 x 20 mL) and ddH_2O. The organic layers were combined and concentrated under vacuum. Purification of the crude material was carried out via normal phase flash chromatography using a gradient of methanol (MeOH) + 0.1% FA/dichloromethane (DCM) + 0.1% FA at a flow rate of 25 mL/min, starting at 10% MeOH + 0.1% FA/90% DCM + 0.1% FA for 1 column volumes then ramping to 15% MeOH + 0.1% FA/85% DCM + 0.1% FA over 8 column volumes, which yielded an amorphous red solid (130 mg, 66%); ^{1}**H NMR** (500 MHz, $CDCl_3$) δ_H 8.10 (dd, 1H, J = 7.7, 1.1), 8.01 (dd, 1H, J = 7.7, 1.1), 7.72 (td, 1H, J = 7.5, 1.3), 7.61 (d, 1H, J = 7.7), 7.60 (td, 1H, J = 7.5, 1.3), 7.40 (d, 1H, J = 8.10), 7.23 (t, 1H, J = 7.5), 7.16 (t, 1H, J = 7.3), 7.10 (d, 1H, J = 2.3), 6.01 (bs, 1H), 5.80 (s, 1H), 3.52 (q, 2H, J = 7.0), 3.16 (t, 2H, J = 7.0); ^{13}**C NMR** (125 MHz, $CDCl_3$) δ_C 190.2, 183.4, 148.2, 136.9, 135.1, 134.1, 132.3, 130.9, 127.4, 126.6, 126.6, 122.9, 122.6, 120.2, 118.9, 112.5, 111.8, 101.3, 42.9, 31.3; **HRMS (ESI):** calc for $C_{20}H_{17}N_2O_2^+$ 317.1285, found $[M+H]^+$ 317.1292, Δ2.2.

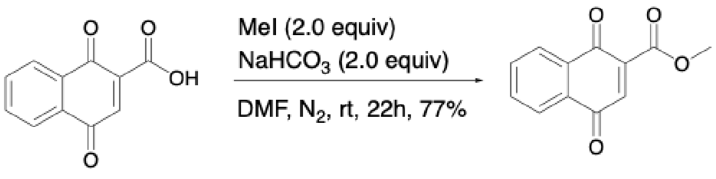

3 **4**

Preparation of 1,4-dihydroxy-2-naphthoic acid (DHNA) methyl ester (4): DHNA (**3**) (24.7 mmol, 5.00 g) and $NaHCO_3$ (23.0 mmol, 2.70 g) was added to a round bottom flask with 100 mL of anhydrous dimethylformamide (DMF). The solution was brought to 0 atm, purged with N_2 (g) and left to stir for 30 min with magnetic stirring. Methyl iodide (38.1 mmol, 2.44 mL) was added dropwise to the stirring mixture. The reaction was left to stir at rt, under N_2 (g), overnight. The reaction was quenched with the addition of brine (10 mL) followed by 1M HCl (10 mL). The mixture was extracted with EtOAc (3 x 100 mL). The organic layers were combined, washed with 1 M K_2CO_3 (5 x 50 mL; to remove DHNA starting material), and brine (3 x 50 mL). The organic layer was dried with Na_2SO_4 and concentrated under vacuum. The resulting solid was resuspended in DCM in a warm water bath until fully dissolved. The mixture was placed in the freezer overnight to form golden colored crystals. The crystals were collected via vacuum filtration yielding a golden yellow solid (4.11 g, 77%); **^{1}H NMR** (400 MHz, CD_3OD) δ_H 8.31 (dt, 1H, J = 8.4, 1.5), 8.14 (dt, 1H, J = 8.4, 1.5), 7.60 (td, 1H, J = 1.29), 7.52 (td, 1H, J = 1.29) 7.07 (s, 1H), 3.96 (s, 3H); **^{13}C NMR** (125 MHz, CD_3OD) δ_C 172.9, 155.7, 146.7, 131.3, 129.8, 127.4, 127.0, 124.8, 123.6, 106.2, 105.4, 53.1; **Melting Point:** 192.9-194.4°C; **HRMS (ESI):** calc for $C_{12}H_9O_4^+$ 217.0501, found $[M+H]^+$ 217.0494, Δ3.2.

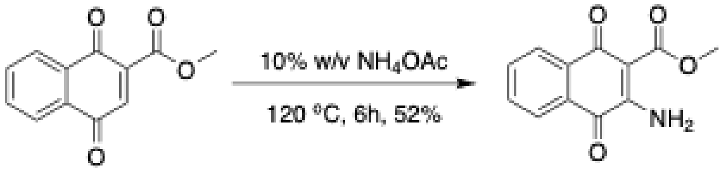

4 **4a**

Preparation of 3 Amino-DHNA-methyl-ester (4a): DHNA-methyl-ester (0.25 g, 1.2 mmol) was added to a round bottom flask and dissolved in a 10% w/v solution of ammonium acetate in glacial acetic acid (1.05 g NH_4OAc in 10.5 mL HOAc). The solution was heated under reflux (120 ℃) for six hours. The reaction mixture was let cool to room temperature, quenched with cold DI H_2O, 50 mL, and extracted with $CHCl_3$ (3x50 mL). The organic layers were combined, washed with saturated $NaHCO_3$ solution (3x 50mL) and dried with Na_2SO_4.. The dried organic sample was concentrated under vacuum with a rotovap. Purification of the crude material was carried out via normal phase flash chromatography using a gradient of ethyl acetate (EtOAc)/hexanes at a flow rate of 25 mL/min, starting at 20% EtOAc/80% hexanes + for 2 column volumes then ramping to 35% EtOAc/65% hexanes over 6 column volumes, which yielded an amorphous red/yellow amorphous solid (0.15 g, 52%). **^{1}H NMR** (500 MHz, CD_3OD) δ_H 8.29 (d, 1H, J = 8.05), 8.23 (d, 1H, J = 8.05) 8.00 (td, 1H, J = 7.40, 1.24), 7.88 (td, 1H, J = 7.40, 1.24) 4.02 (s. 3H); **^{13}C NMR** (125 MHz, CD_3OD) δ_C 180.4, 180.3, 169.4, 154.7, 136.1, 134.8, 133.1, 130.7, 127.2, 126.6, 101.0, 51.4; **HRMS (ESI)** calc for $C_{12}H_{10}O_4N^+$ 232.0610, found: $[M+H]^+$ 232.0611 *m/z*, Δ0.4.

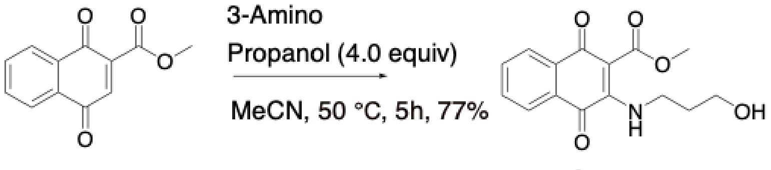

4 **4e**

Preparation of 3 Amino-Propanol-DHNA-methyl-ester (4e): DHNA-methyl-ester (**4**) (0.93 mmol, 0.20 g) and 3-amino-propanol (3.72 mmol, 0.29 mL) were dissolved in MeCN (10.0 mL). The mixture was stirred at 50 ℃ in an oil bath for 5 h with magnetic stirring. The reaction was quenched with ddH_2O (25 mL) and was extracted with EtOAc (3 x 25 mL). The organic layer was dried with Na_2SO_4, and concentrated under vacuum. The resulting solid

was resuspended in 1:1 MeCN/H_2O in a warm water bath until completely dissolved. The mixture was then dried down on rotovap until crystals formed in the liquid (about half of the original liquid was dried). The crystals were removed through vacuum filtration. A bright orange crystalline solid was obtained (0.25 g, 77%); ^{1}**H NMR** (500 MHz, CD_3OD) δ_H 8.19 (t, 2H, J = 7.09), 7.95 (dt, 1H, J = 7.66, 1.38), 7.85 (dt, 1H, J = 7.66, 1.38), 4.02 (s, 3H), 3.83 (t, 2H, J = 5.53), 3.46 (m, 2H), 2.03 (p, 2H, J = 6.20); ^{13}**C NMR** (125 MHz, CD_3OD) 182.2, 181.5, 169.3, 139.3, 136.1, 134.1, 133.6, 131.2, 127.4, 126.9, 119.1, 60.7, 52.6, 42.8; **Melting Point:** 81.1-83.0℃; **HRMS (ESI):** calc for $C_{15}H_{16}NO_5^+$ 290.1077, found $[M+H]^+$ 290.1082, Δ1.7.

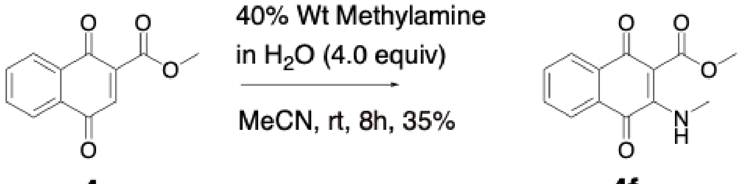

4 **4f**

Preparation of Methylamine-DHNA-methyl-ester (4f): DHNA-methyl-ester (**4**) (0.93 mmol, 0.20 g) and 40% wt methylamine solution in H_2O (3.7 mmol, 0.41 mL solution) were dissolved in MeCN (10.0 mL). The mixture was stirred at room temperature for 8 h with magnetic stirring. The reaction was quenched with ddH_2O (25 mL) and was extracted with EtOAc (3 x 25 mL). The organic layer was dried with Na_2SO_4, and concentrated under vacuum. The resulting solid was resuspended in 1:1 MeCN/H_2O until completely dissolved. The mixture was dried down on rotovap until crystals formed in the liquid (about half of the original liquid was dried). The crystals were removed through vacuum filtration. A bright pink crystalline solid was obtained (0.08 g, 36%); ^{1}**H NMR** (500 MHz, CD_3CN) δ_H 8.06 (d, 2H, J = 8.2), 7.83 (td, 1H, J = 7.4, 1.1), 7.73 (td, 1H, J = 7.4, 1.1), 3.86 (s, 3H), 2.93(m, 3H); ^{13}**C NMR** (125 MHz, CD_3CN) δ_C 182.4, 180.7, 168.3, 136.1, 133.8, 131.1, 128.5, 127.1, 126.7, 125.1, 52.6, 30.4; **Melting Point:** 198.0-201.3℃; **HRMS (ESI):** calc for $C_{13}H_{12}NO_4^+$ 246.0766, found $[M+H]^+$ 246.0761, Δ2.0.

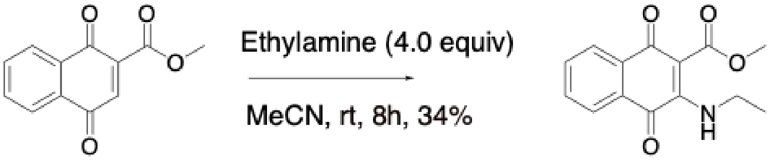

4 **4g**

Preparation of Ethylamine-DHNA-methyl-ester (4g): DHNA-methyl-ester (**4**) (0.93 mmol, 0.20 g) and ethylamine (3.7 mmol, 0.24 mL) were dissolved in MeCN (10.0 mL). The mixture was stirred at room temperature for 8 h with magnetic stirring. The reaction was quenched with ddH_2O (25 mL) and was extracted with EtOAc (3 x 25 mL). The organic layer was dried with Na_2SO_4, and concentrated under vacuum. The resulting solid was resuspended in 1:1 MeCN/H_2O until completely dissolved. The mixture was dried down on rotovap until crystals formed in the liquid (about half of the original liquid was dried). The crystals were removed through vacuum filtration. A bright orange crystalline solid was obtained (0.09 g, 36%); ^{1}**H NMR** (500 MHz, $CDCl_3$) δ_H 8.15 (d, 1H, J = 8.0), 8.03 (d, 1H, J = 8.0), 7.76 (td, 1H, J = 7.7, 1.8), 7.64 (td, 1H, J = 7.7, 1.8), 3.93 (s, 3H), 3.20-3.45 (m, 2H), 1.35 (t, 3H, J = 7.4); ^{13}**C NMR** (125 MHz, $CDCl_3$) δ_C 181.5, 179.9, 167.5, 143.4, 135.2, 133.0, 132.2, 129.6, 126.5, 126.3, 119.6, 52.3, 38.3, 14.7; **Melting Point:** 132.7-133.4℃; **HRMS (ESI):** calc for $C_{14}H_{14}NO_4^+$ 260.0923, found $[M+H]^+$ 260.0916, Δ2.7.

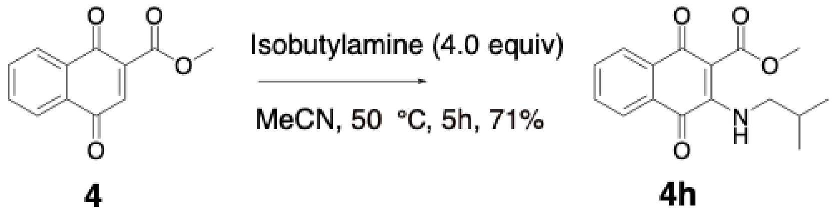

Preparation of Isobutylamine-DHNA-methyl-ester (4h): DHNA-methyl-ester (**4**) (0.93 mmol, 0.20 g) and isobutylamine (3.7 mmol, 0.37 mL) were dissolved in MeCN (10.0 mL). The mixture was stirred at 50 ℃ in an oil bath for 5 h with magnetic stirring. The reaction was quenched with ddH_2O (25 mL) and was extracted with EtOAc (3 x 25 mL). The organic layer was dried with Na_2SO_4, and concentrated under vacuum. The resulting solid was resuspended in 1:1 MeCN/H_2O until completely dissolved. The mixture was dried down on rotovap until crystals formed in the liquid (about half of the original liquid was dried). The crystals were removed through vacuum filtration. A bright orange crystalline solid was obtained (0.19 g, 71%); **^{1}H NMR** (500 MHz, $CDCl_3$) δ_H 8.12 (d, 1H, J = 7.0), 8.01 (d, 1H, J = 7.0), 7.75 (t, 1H, J = 7.8), 7.63 (t, 1H, J = 7.8), 3.91 (s, 3H), 1.95 (sep, 1H, J = 6.7), 1.02 (s, 3H), 1.00 (s, 3H), 0.93 (d, 2H, J = 5.6); **^{13}C NMR** (125 MHz, $CDCl_3$) δ_C 181.9, 173.5, 160.2, 154.5, 143.8, 136.2, 135.6, 132.3, 126.5, 125.7, 99.1, 52.6, 46.2, 32.2, 29.9, 28.7; **Melting Point:** 99.1-102.2℃; **HRMS (ESI):** calc for $C_{16}H_{18}NO_4^+$ 288.1236**,** found $[M+H]^+$ 288.1230, Δ2.1.

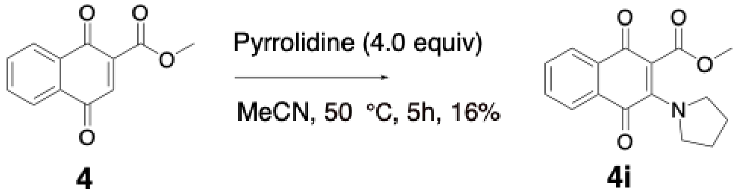

Preparation of Pyrrolidine-DHNA-methyl-ester (4i): DHNA-methyl-ester (**4**) (0.93 mmol, 0.20 g) and pyrrolidine (3.7 mmol, 0.31 mL) were dissolved in MeCN (10.0 mL). The mixture was stirred at 50 ℃ in an oil bath for 5 h with magnetic stirring. The reaction was quenched with ddH_2O (25 mL) and was extracted with EtOAc (3 x 25 mL). The organic layer was dried with Na_2SO_4, and concentrated under vacuum. The resulting solid was resuspended in 1:1 MeCN/H_2O until completely dissolved. The mixture was dried down on rotovap until crystals formed in the liquid (about half of the original liquid was dried). The crystals were removed through vacuum filtration. A dark red crystalline solid was obtained (0.04 g, 16%); **^{1}H NMR** (500 MHz, $CDCl_3$) δ_H 8.07 (dd, 1H, J = 8.0, 0.86), 7.95 (dd, 1H, J = 8.0, 0.86), 7.70 (td, 1H, J = 7.44, 1.0), 7.60 (td, 1H, J = 7.4, 1.0), 3.92 (s, 3H), 3.68-3.82 (m, 4H), 1.93 (pent, 4H, J = 3.2); **^{13}C NMR** (125 MHz, $CDCl_3$) δ_C 184.1, 180.3, 168.8, 148.5, 134.5, 132.8, 132.2, 131.5, 126.3, 125.9, 110.4, 103.5, 52.81, 52.76, 25.6 (two overlapping peaks); **Melting Point:**136.1-139.8℃; **HRMS (ESI):** calc for $C_{16}H_{16}NO_4^+$ 286.0179, found $[M+H]^+$ 286.0173, Δ2.1.

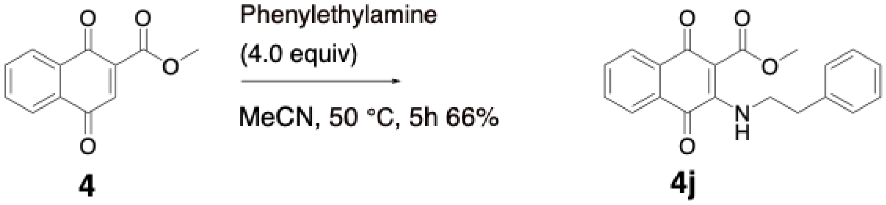

Preparation of Phenyl-ethylamine-DHNA-methyl-ester (4j): DHNA-methyl-ester (**4**) (0.93 mmol, 0.20 g) and phenylethylamine (3.7 mmol, 0.47 mL) were dissolved in MeCN (10.0 mL). The mixture was stirred at 50 ℃ in an oil bath for 5 h with magnetic stirring. The reaction was quenched with ddH_2O (25 mL) and was extracted with EtOAc (3 x 25 mL). The organic layer was dried with Na_2SO_4, and concentrated under vacuum. The resulting solid

was resuspended in 1:1 MeCN/H_2O until completely dissolved. The mixture was dried down on rotovap until crystals formed in the liquid (about half of the original liquid was dried). The crystals were removed through vacuum filtration. A bright red/orange crystalline solid was obtained (0.21 g, 66%); **^{1}H NMR** (500 MHz, $CDCl_3$) δ_H 8.12 (d, 1H, J = 7.8), 7.99 (d, 2H, 7.8), 7.75 (t, 1H, J = 7.8), 7.63 (t, 1H, J = 7.8), 7.34 (t, 2H, 7.3), 7.24-7.34 (m, 3H), 3.91 (s, 3H), 3.67-3.43 (m, 2H), 2.99 (t, 2H, J = 7.3); **^{13}C NMR** (125 MHz, $CDCl_3$) δ_C 182.2, 180.4, 167.8, 144.0, 137.7, 135.7, 133.5, 132.8, 130.2, 129.3, 129.1, 127.5, 126.8, 123.9, 118.1, 107.3, 52.8. 44.9, 35.9; **Melting Point:** 129.7-135.2°C; **HRMS (ESI):** calc for $C_{20}H_{18}NO_4^+$ 336.1236, found $[M+H]^+$ 336.1231, Δ1.5.

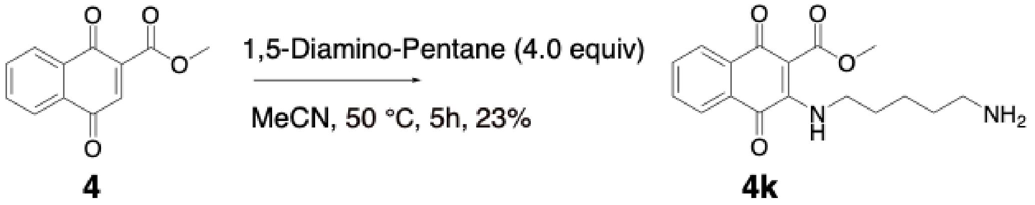

Preparation of cadaverine-DHNA-methyl-ester (4k): DHNA-methyl-ester (**4**) (0.93 mmol, 0.20 g) and cadaverine (3.7 mmol, 0.436 mL) were dissolved in MeCN (10.0 mL). The mixture was stirred at 50 ℃ in an oil bath for 5 h with magnetic stirring. The reaction was quenched with ddH_2O (25 mL) and was extracted with EtOAc (3 x 25 mL). The organic layer was dried with Na_2SO_4, and concentrated under vacuum. The crude material was resuspended in 1:1 MeCN/H_2O and purified by reverse phase prep HPLC using the following gradient: holding 35% MeCN + 0.1% formic acid (FA)/65% H_2O + 0.1% FA for 1 min followed by a linear gradient to 65% MeCN + 0.1% FA/35% H_2O + 0.1% FA over 24 min all at a flow rate 10 mL/min to yield an amorphous yellow solid (0.06 g, 23%); **^{1}H NMR** (500 MHz, CD_3OD) δ_H 8.20 (t, 2H, J = 7.1), 7.96 (td, 1H, J = 6.7, 1.1), 7.87 (td, 1H, J = 6.7, 1.1), 4.02 (s, 3H), 3.33-3.52 (m, 2H), 3.08 (t, 2H, J = 7.4), 1.76-1.93 (m, 4H), 1.51-1.66 (m, 2H); **^{13}C NMR** (125 MHz, CD_3OD) δ_C 181.9, 170.8, 168.3, 136.6, 134.4, 134.1, 131.5, 127.7, 127.3, 104.6, 94.4, 93.2, 53.0, 40.7, 29.9, 28.4, 25.0; **HRMS (ESI):** calc for $C_{17}H_{21}N_2O_4^+$ 317.1501, found $[M+H]^+$ 317.1491, Δ3.2.

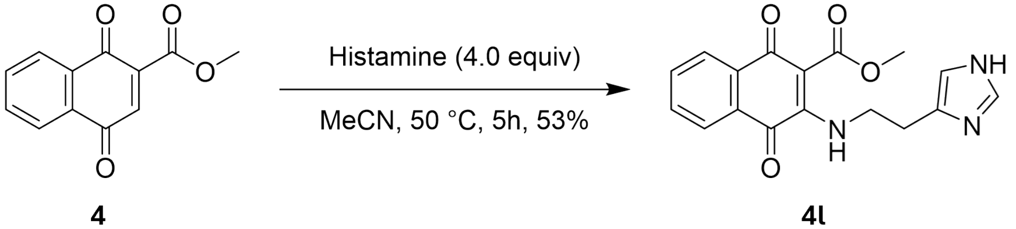

Preparation of Histamine-DHNA-methyl-ester (4l) DHNA-methyl-ester (**4**) (0.93 mmol, 0.20 g) and histamine (3.7 mmol, 0.41 g) were dissolved in MeCN (10.0 mL). Drops of water were added to the mixture until histidine was fully dissolved. The mixture was stirred at 50 ℃ in an oil bath for 5 h with magnetic stirring. The reaction was quenched with ddH_2O (25 mL) and was extracted with EtOAc (3 x 25 mL). The organic layer was dried with Na_2SO_4, and concentrated under vacuum. The crude material was resuspended in 1:1 MeCN/H_2O and purified by reverse phase prep HPLC using the following gradient: holding 35% MeCN + 0.1% formic acid (FA)/65% H_2O + 0.1% FA for 1 min followed by a linear gradient to 65% MeCN + 0.1% FA/35% H_2O + 0.1% FA over 24 min all at a flow rate 10 mL/min to yield an amorphous yellow solid (0.16, 53%); **^{1}H NMR** (500 MHz, CD_3OD) δ_H 8.37 (s, 1H), 8.27 (s, 1H), 8.15 (dd, 2H, J = 7.6, 0.96), 7.92 (td, 1H, J = 7.5, 1.4), 7.82 (td, 1H, J = 7.5, 1.4), 7.27 (s, 1H), 3.96 (s, 3H), 3.61-3.82 (m, 2H), 3.13 (t, 2H, J = 6.7); **^{13}C NMR** (125 MHz, CD_3OD) δ_C 181.7, 169.5, 166.7, 136.3, 136.0, 134.3, 134.1, 133.9, 131.5, 127.5, 127.1, 124.2, 117.5, 52.8, 44.1, 26.8; **HRMS (ESI):** calc for $C_{17}H_{16}N_3O_4^+$ 326.1141, found $[M+H]^+$ 326.1139, Δ0.6.

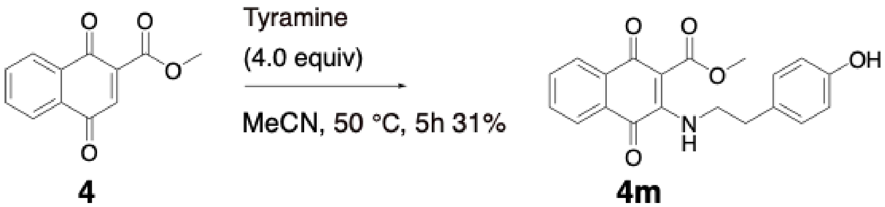

Preparation of Tyramine-DHNA-methyl-ester (4m): DHNA-methyl-ester (**4**) (0.93 mmol, 0.20 g) and tyramine (3.7 mmol, 0.51 g) were dissolved in MeCN (10.0 mL). Drops of water were added to the mixture until tyramine was fully dissolved. The mixture was stirred at 50 ℃ in an oil bath for 5 h with magnetic stirring. The reaction was quenched with ddH_2O (25 mL) and was extracted with EtOAc (3 x 25 mL). The organic layer was dried with Na_2SO_4, and concentrated under vacuum. The resulting solid was resuspended in 1:1 MeCN/H_2O until completely dissolved. The mixture was dried down on rotovap until crystals formed in the liquid (about half of the original liquid was dried). The crystals were removed through vacuum filtration. A bright yellow crystalline solid was obtained (0.10 g, 31%); ^{1}**H NMR** (400 MHz, CD_3CN) δ_H 7.81 (td, 1H, J = 7.9, 1.6), 7.70 (td, 1H, J = 8.1, 1.6), 7.09 (d, 2H, J = 8.7), 6.77 (d, 2H, J = 8.7), 3.82 (s, 3H), 3.32-3.48 (m, 2H), 2.85 (t, 2H, J = 7.6); ^{13}**C NMR** (125 MHz, CD_3CN) δ_C 182.0, 180.2, 168.1, 156.1, 136.1 (2x), 135.6, 133.2, 131.7, 130.5, 130.2, 129.7, 126.7, 126.2, 125.1, 115.8, 103.4, 52.2, 45.0, 34.3; **Melting Point**: 179.9-182.1℃ **HRMS (ESI):** calc for $C_{20}H_{18}NO_5^+$ 352.1185, found $[M+H]^+$ 352.1184, Δ0.3.

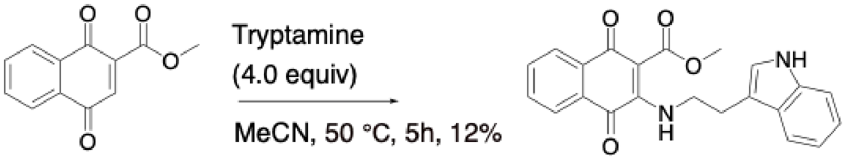

Preparation of Tryptamine-DHNA-methyl-ester (4n): DHNA-methyl-ester (**4**) (0.93 mmol, 0.20 g) and tryptamine (3.7 mmol, 0.51g) were dissolved in MeCN (10.0 mL). Drops of water were added to the mixture until tryptamine was fully dissolved. The mixture was stirred at 50 ℃ in an oil bath for 5 h with magnetic stirring. The reaction was quenched with ddH_2O (25 mL) and was extracted with EtOAc (3 x 25 mL). The organic layer was dried with Na_2SO_4, and concentrated under vacuum. The crude material was resuspended in 1:1 MeCN/H_2O and purified by reverse phase prep HPLC using the following gradient: holding 35% MeCN + 0.1% formic acid (FA)/65% H_2O + 0.1% FA for 1 min followed by a linear gradient to 65% MeCN + 0.1% FA/35% H_2O + 0.1% FA over 24 min all at a flow rate 10 mL/min to yield an amorphous orange solid (50 mg, 12%); ^{1}**H NMR** (400 MHz, CD_3OD) δ_H 8.04 (d, 2H, J = 7.2), 7.80 (m, 1H), 7.69 (m, 1H), 7.59 (d, 1H, J = 5.5), 7.34 (m, 1H), 7.15 (m, 1H), 7.10 (t, 1H, 7.4), 7.03 (t, 1H, J = 6.9), 3.83 (s, 3H), 3.39-3.56 (m, 2H), 3.14 (t, 2H, J = 7.3); ^{13}**C NMR** (125 MHz, CD_3OD) δ_C 181.3, 172.3, 162.9, 137.9, 135.9, 133.9, 133.4, 131.0, 128.1, 127.2, 126.7, 123.7, 122.2, 119.52, 118.9, 112.0, 111.6, 108.5, 107.7, 52.4, 44.8, 25.7; **HRMS (ESI):** calc for $C_{22}H_{19}N_2O_4^+$ 375.1345, found $[M+H]^+$ 375.1348, Δ0.8.

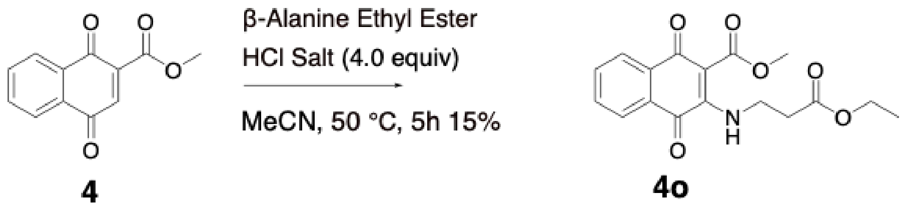

Preparation of β-Alanine-ethyl-ester-DHNA-methyl-ester (4o): DHNA-methyl-ester (**4**) (0.93 mmol, 0.20 g) and β-Alanine-ethyl-ester HCl salt (3.7 mmol, 0.57 g) were dissolved in MeCN (10.0 mL). Drops of water were added to the mixture until β-Alanine-ethyl-ester HCl salt was fully dissolved. The mixture was stirred at 50 ℃ in an oil bath for 5 h with magnetic stirring. The reaction was quenched with ddH$_2$O (25 mL) and was extracted with EtOAc (3 x 25 mL). The organic layer was dried with Na$_2$SO$_4$, and concentrated under vacuum. The resulting solid was resuspended in 1:1 MeCN/H$_2$O until completely dissolved. The mixture was dried down on rotovap until crystals formed in the liquid (about half of the original liquid was dried). The crystals were removed through vacuum filtration. A pale yellow/brown crystalline solid was obtained (50 mg, 15%); ^{1}**H NMR** (500 MHz, CD$_3$OD) δ_H 8.04 (td, 2H, J = 6.9, 0.96), 7.81 (dt, 1H, J = 7.7, 1.6), 7.71 (dt, 1H, J = 7.7, 1.6), 4.15 (q, 2H, J = 7.3), 3.87 (s, 3H), 3.44-3.65 (m, 2H), 2.72 (t, 2H, 6.9), 1.26 (t, 3H, J = 7.1); ^{13}**C NMR** (125 MHz, CD$_3$OD) δ_C 182.4, 181.7, 172.9, 169.4, 136.3, 134.12, 133.9, 131.5, 127.6, 127.1, 62.0, 52.9, 49.6, 49.5, 40.5, 34.6, 12.5; **HRMS (ESI):** calc for $C_{17}H_{18}NO_6^+$ 332.1134, found $[M+H]^+$ 332.1124, Δ3.0.

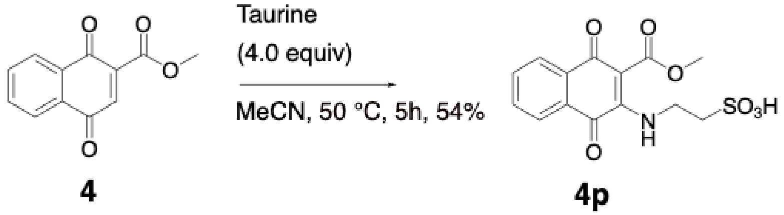

Preparation of Taurine-DHNA-methyl-ester (4p): DHNA-methyl-ester (**4**) (0.93 mmol, 0.20 g) and taurine (3.7 mmol, 0.47 g) were dissolved in MeCN (10.0 mL). Drops of water were added to the mixture until taurine was fully dissolved. The mixture was stirred at 50 ℃ in an oil bath for 5 h with magnetic stirring. The reaction was quenched with ddH$_2$O (25 mL) and was extracted with 1-butanol (3 x 25 mL). The organic layer was dried with Na$_2$SO$_4$, and concentrated under vacuum. The crude material was resuspended in 1:1 MeCN/H$_2$O and purified by reverse phase prep HPLC using the following gradient: holding 35% MeCN + 0.1% formic acid (FA)/65% H$_2$O + 0.1% FA for 1 min followed by a linear gradient to 65% MeCN + 0.1% FA/35% H$_2$O + 0.1% FA over 24 min all at a flow rate 10 mL/min to yield an amorphous yellow solid (0.17 g, 54%); ^{1}**H NMR** (400 MHz, CD$_3$OD) δ_H 7.95 (t, 2H, J = 7.3), 7.71 (td, 1H, J = 7.7, 1.6), 7.62 (td, 1H, J = 7.7, 1.6), 3.81 (s, 3H), 3.47-3.63 (m, 2H), 3.03 (t, 2H, J = 6.1); ^{13}**C NMR** (100 MHz, CD$_3$OD) δ_C 182.4, 192.0, 169.4, 146.3, 136.5, 134.3, 134.0, 131.6, 127.8, 127.3, 125.0, 53.1, 41.4, 37.4; **HRMS (ESI):** calc for $C_{14}H_{14}NO_7S^+$ 340.0491, found $[M+H]^+$ 340.0494, Δ0.9.

b. Other Experimental Methods

Molecular Energy Calculations and Reduction Potential Predictions Methods: All calculations were done using Gaussian 03 on WebMO server, accessed through Virginia Tech and followed previously published methods.[2] Geometry optimizations for the ground state (S_o), and radical anionic state (D_o) (charge = -1, Multiplicity: doublet) of each compound were taken using B3LYP level theory, a 6-311+G(d,p) basis set, and simulated in acetonitrile. The molecular energies were taken of the compounds and optimized using the same level theory, basis set, and solvent. The molecular energy difference was taken between the radical anionic and the ground state (D_o-S_o) of the compounds. The molecular energy differences were converted from Hartrees to V. The calculated molecular energy differences in V (y-axis) were taken and graphed against the experimentally determined one electron reduction potentials (x-axis) of the compounds. The values correlated to a linear regression. The equation of the line of best

fit was used to predict the experimental one electron reduction potential of new compounds after computing the molecular energy difference between ground and radical anionic states.

Determining Mediators Ndh2 Binding Affinity through Molecular Docking: A homology model of *L. plantarum* Ndh2 was constructed using Schrödinger Maestro v14.1[3], with *S. cerevisiae* NDH2 (PDB ID: 4G73)[4] serving as the only structural template. Structural alignment between the *L. plantarum* model and the *S. cerevisiae* template was performed in PyMOL v3.0[5] to facilitate comparison of binding site geometry. Residues Arg-314 and Tyr-403 in the *L. plantarum* model were optimized for rotameric favorability within the binding site using the PyMOL v3.0 rotamer selection tools. These modifications were made to better preserve key conserved features and steric volume between the two binding sites, as quantified using CavitOmiX.[6]

Molecular Docking, Binding Site Analysis, and Compound Ranking: Redocking of the native quinone ligand into *S. cerevisiae* Ndh2 was performed using GNINA[7] within the *S. cerevisiae* Ndh2 (PDB ID: 4G73) crystal structure.[4] GNINA extends the AutoDock Vina framework and previous work on *L. plantarum* Ndh2[1,8,9] by incorporating convolutional neural networks to improve pose prediction accuracy and scoring function performance, particularly for diverse and flexible ligand sets, which showed more consistent low energy and chemically feasible poses than that of AutoDock Vina. A docking box centered at coordinates (x = 38.5, y = 46.0, z = 4.0) with dimensions 16 × 24 × 12 $Å^3$ was used to define the search space. Docking accuracy was validated by calculating the root-mean-square deviation (RMSD) between the crystallographic ligand pose and the top-ranked docked pose, yielding an RMSD of 1.43 Å, indicative of successful protocol to replicate crystal structure quinone positioning relevant to FAD. The homology model of *L. plantarum* NDH2 was structurally aligned to the *S. cerevisiae* Ndh2 reference structure, and the same docking box parameters were retained to ensure consistency in pose comparison. A total of 42 compounds (listed in **Table 1**) were subsequently docked into both the *L. plantarum* homology model and *S. cerevisiae* Ndh2 using the GNINA parameters listed above.

Binding free energies were estimated using Molecular Mechanics/Generalized Born Surface Area (MM/GBSA, kcal/mol) calculations, with ligand efficiency (LE, kcal/mol per heavy atom) included to normalize for ligand size. All calculations were performed in Schrödinger Maestro v14.1[3] for each docked pose across both the *L. plantarum* and *S. cerevisiae* Ndh2 structures. The most energetically favorable poses, defined by the lowest MM/GBSA and LE values, were selected for further analysis. Protein–ligand interaction fingerprints were computed for all poses, and representative top-ranked poses were visualized in PyMOL v3.0.

RidgeCV, a cross-validated Ridge regression linear model stemming from the python package SKLearn, was used to train, test, and cross-validate the composite score and ranking generated computationally. MM/GBSA (Put deltaGComp) and compound feature analysis from Schrodinger Maestro v 14.1 QikProp was used as input factors for the model, with the EET experimental metric positioned as the dependent factor that the model was built against. The mean MM-GBSA (ΔG_{comp}) was averaged for each pose of the 42 compounds independently and merged with its associated compound QikProp dataset. The data was then preprocessed with the StandardScaler from SKLearn, standardizing features to improve the accuracy of the model by centering features means and unit variance.

The processed data was loaded, tested, and trained on a RidgeCV model with alphas of 0.1, 1, and 10. While the total amount of data inputted into the model is relatively small, the Spearman's rank correlation reported a positive monotonic relationship of 0.87, indicating a positive and relatively strong correlation between EET and our composite score. The composite score was then ranked (with higher, positive scores indicating better EET performance), providing us with our computationally generated ranking. Feature importance, and their weighted directionality, was evaluated for biological relevance.

Quantifying Iron Reduction of Quinones: Iron reduction assays were conducted following a previously published procedure.[10] Briefly, both *L. plantarum* NCIMB8826 strains (*ΔdmkAΔndh1* and *ΔdmkAΔndh1Δndh2*) were cultured in 3 mL MRS broth (Sigma Aldrich) in a static incubator at 37℃ overnight. The cultures were sub-inoculated in modified MRS (mMRS) broth [mMRS: combining the following components - 1000 mL of ddH_2O, 10 g protease peptone, 1 g tween 80, 10 g mannitol, 5 g yeast extract, 2 g K_2HPO_4, 8.3 g sodium acetate trihydrate, 2.15 g

ammonium citrate tribasic, 0.1 g $MgSO_4$, and 0.05 g $MnSO_4 \cdot H_2O$; pH adjusted to 6.5 with NaOH and then sterile filtered (0.22 µm)] to initial OD of 0.1 and left to grow overnight in a static incubator at 37℃. The cells were pelleted (4500 g, 4℃, 10 min), washed (x2) with PBS (pH 7.4), then samples were adjusted to a final OD of 4 in PBS. The iron reduction mix was prepared by combining equal amounts of 5x PBS, mannitol (100 mg/mL in water), iron(III) oxide nanoparticles (20 mM in water, <50 nm), ferrozine (20 mM in water), and ddH_2O. Under anaerobic conditions, 100 µL of the iron reduction mixture, 100 µL of the PBS cell mixture, and 2 µL of each mediator (500 µM in DMSO) was added to individual wells of a 96-well plate (Millipore MSSCNFX00). DMSO served as the vehicle control. The 96-well plates were incubated at room temperature inside a box for 3 h. At which time, the plates were removed from the anaerobic chamber, centrifuged (4000 rpm, 10 min), and approximately 100 µL from each well was removed via multi-channel pipette and placed in a new 96-well plate. Absorbances of all assay plates were measured at 562 nm and recorded. Assays were conducted with six technical replicates for all *L. plantarum* strains (*ΔdmkAΔndh1*, *ΔdmkAΔndh1Δndh2,* *ΔdmkAΔndh1Δndh2* + pSL93*,* *ΔdmkAΔndh1Δndh2* + pRA01) and as well as 3 biological replicates. Data in graphs represents the biological replicates.

An iron(II) standard curve was made through making serial dilutions of ferrous sulfate heptahydrate (0.8, 0.4, 0.2, 0.1, 0.05, and 0 mM) in 0.5 M HCl (*aq*). A 2x ferrozine solution was made by a 1:3:1 ratio mixture of pH 7.4 PBS, ferrozine (20 mM in H_2O), and H_2O. In a 96-well plate, 50 µL of each standard concentration sample was mixed with 50 µL of 2x ferrozine in PBS in triplicate, yielding final Fe^{2+} of 0.4, 0.2, 0.1, 0.05, 0.025, and 0 mM. The plate was left to sit in the dark for 5 min and absorbance recorded at 562 nm on the Byonoy Absorbance 96 plate reader was used to generate the calibration curve. The line of best fit was adjusted to have a y-intercept of 0 ($R^2 = 0.99$).

c. References

(1) Blackburn, B. T.; Alba, R. A. C.; Porokhin, V. O.; Hatch, A.; Hassoun, S.; Ajo-Franklin, C. M.; Mevers, E. Identifying Key Properties That Drive Redox Mediator Activity in Lactiplantibacillus Plantarum. *Angew. Chem. Int. Ed Engl.* **2025**, *64* (19). https://doi.org/10.26434/chemrxiv-2024-njrjh.
(2) Speelman, A. L.; Gillmore, J. G. Efficient Computational Methods for Accurately Predicting Reduction Potentials of Organic Molecules. *J. Phys. Chem. A* **2008**, *112* (25), 5684–5690.
(3) Maestro, Schrödinger, LLC, New York, NY. *Schrödinger Release 2025-2:* . 2025.
(4) Feng, Y.; Li, W.; Li, J.; Wang, J.; Ge, J.; Xu, D.; Liu, Y.; Wu, K.; Zeng, Q.; Wu, J.-W.; Tian, C.; Zhou, B.; Yang, M. Structural Insight into the Type-II Mitochondrial NADH Dehydrogenases. *Nature* **2012**, *491* (7424), 478–482.
(5) *The PyMOL Molecular Graphics System, LLC., Version 3 0;*
(6) Parigger, L.; Krassnigg, A.; Hetmann, M.; Hofmann, A.; Gruber, K.; Steinkellner, G.; Gruber, C. C. CavitOmiX Drug Discovery: Engineering Antivirals with Enhanced Spectrum and Reduced Side Effects for Arboviral Diseases. *Viruses* **2024**, *16* (8), 1186.
(7) McNutt, A. T.; Francoeur, P.; Aggarwal, R.; Masuda, T.; Meli, R.; Ragoza, M.; Sunseri, J.; Koes, D. R. GNINA 1.0: Molecular Docking with Deep Learning. *J. Cheminform.* **2021**, *13* (1). https://doi.org/10.1186/s13321-021-00522-2.
(8) Ragoza, M.; Hochuli, J.; Idrobo, E.; Sunseri, J.; Koes, D. R. Protein-Ligand Scoring with Convolutional Neural Networks. *J. Chem. Inf. Model.* **2017**, *57* (4), 942–957.
(9) Porokhin, V.; Brown, A. M.; Hassoun, S. Protein-Ligand Co-Design: A Case for Improving Binding Affinity between Type II NADH:quinone Oxidoreductase and Quinones. *J. Comput. Aided Mol. Des.* **2025**, *39* (1). https://doi.org/10.1007/s10822-025-00613-4.
(10) Pan, X.; Wang, H.; Li, C.; Zhang, J. Z. H.; Ji, C. MolGpka: A Web Server for Small Molecule pKa Prediction Using a Graph-Convolutional Neural Network. *J. Chem. Inf. Model.* **2021**, *61* (7), 3159–3165.

d. Supplementary Tables

Table S1. Compounds comprising the mediator library. Ndh2-dependent EET activity, physicochemical properties, and biochemical interactions for mediator library.

**Mediator**	**Ndh2 Dependent EET (µM of Fe^{2+})**	**cLog*P***	**pKa[11]**	**Log*D*[at pH 7.4**	**ΔG_{comp} (kcal/mol)**
**1**	2.00 ± 0.7	2.17	1.8	2.17	-28.30
**2**	0.19 ± 1.3	1.65	NA	1.65	-21.27
**4**	2.51 ± 2.0	1.68	44.0	1.68	-36.99
**1a**	20.0 ± 4.6	2.22	9.2	0.02	-28.81
**1b**	3.59 ± 1.1	1.75	10.6	1.75	-30.85
**1c**	6.60 ± 1.9	1.75	10.6	1.75	-33.97
**1d**	0.81 ± 0.7	1.44	10.2	1.44	-33.35
**1e**	0.30 ± 0.2	1.82	10.7	1.82	-32.18
**1f**	4.50 ± 0.5	2.26	11.7	2.26	-29.56
**1g**	9.65 ± 2.5	2.80	11.9	2.79	-29.68
**1h**	15.7 ± 2.3	3.72	11.1	3.72	-31.97
**1i**	1.23 ± 0.7	3.14	1.8	3.14	-30.73
**1j**	13.1 ± 1.3	4.36	10.6	4.36	-39.98
**1k**	1.00 ± 0.1	2.49	10.9	2.49	-28.39
**1l**	11.4 ± 2.9	1.24	10.5	1.24	-36.69
**1m**	10.3 ± 1.7	3.69	9.9	3.69	-41.14
**1n**	16.8 ± 1.7	4.35	10.7	4.35	-36.86
**1o**	2.56 ± 0.7	1.86	10.2	1.86	-32.88
**2c**	0.87 ± 0.7	1.23	11.7	1.23	-28.61
**2d**	1.00 ± 1.1	0.92	11.3	0.92	-30.44
**2e**	-0.19 ± 0.6	1.30	11.6	1.30	-35.64
**2f**	2.65 ± 1.1	1.09	11.7	1.09	-27.75
**2g**	0.93 ± 0.5	2.27	11.9	2.27	-30.50
**2h**	-0.19 ± 1.6	3.20	12	3.20	-30.12
**2i**	2.00 ± 1.5	2.62	1.6	2.62	-32.98
**2j**	7.55 ± 1.6	3.84	11.6	3.84	-39.85
**2k**	0.09 ± 1.4	1.97	11.9	1.97	-31.29
**2l**	3.17 ± 0.1	0.717	11.5	0.72	-34.73
**2m**	4.76 ± 1.4	3.17	10.3	3.17	-44.17

**2n**	17.5 ± 1.6	3.83	11.8	3.83	-42.83
**4e**	4.72 ± 2.4	0.487	7.4	0.19	-33.33
**4f**	4.16 ± 2.9	0.924	6.7	0.12	-32.03
**4g**	5.68 ± 2.8	1.90	10.5	1.90	-32.68
**4h**	8.39 ± 3.0	2.38	8.2	2.38	-34.99
**4i**	2.68 ± 1.6	1.80	1.6	1.80	-34.20
**4j**	9.85 ± 1.9	3.02	7.1	2.52	-42.42
**4k**	8.35 ± 2.4	-1.12	7.9	-1.21	-37.84
**4l**	4.77 ± 2.1	-0.100	7.0	-0.60	-35.56
**4m**	5.02 ± 0.6	2.35	6.8	1.75	-47.68
**4n**	13.3 ± 1.5	3.01	7.4	2.71	-37.88
**4o**	-0.97 ± 0.8	0.527	6.7	-0.27	-35.18
**4p**	2.40 ± 0.7	-0.677	8.8	-0.68	-33.65

**Table S2**: Reduction potential of mediators.			
**Compound**	**RB3LYP Energy of Geometry Optimization in Hartrees (Ground State)**	**RB3LYP Energy of Geometry Optimization in Hartrees (Radical Anion)**	**E° (V)**
Menadione (**1**)	-1509430.271	-1509800.368	-1.09
Naphthoquinone (**2**)	-1406100.801	-1406479.906	-1.03
1,4-Dihydroxy-2-naphthoic acid methyl ester (**4**)	-2004893.537	-2005312.253	-0.76
3-Amine-menadione (**1a**)	-1654945.358	-1655293.258	-1.24
3-[(*S*)-amino-2-propanol]-menadione (**1b**)	-2162523.786	-2162877.948	-1.19
3-[(*R*)-amino-2-propanol]-menadione (**1c**)	-2162527.001	-2162890.529	-1.13
3-Aminoethanol-menadione (**1d**)	-2059191.869	-2059548.803	-1.17
3-Aminopropanol-menadione (**1e**)	-2162507.952	-2162860.641	-1.20
3-Methylamine-menadione (**1f**)	-1758196.462	-1758551.542	-1.19
3-Ethylamine-menadione (**1g**)	-1861515.787	-1861867.671	-1.21
3-Isobutylamine-menadione (**1h**)	-2068115.873	-2068497.828	-1.01
3-Pyrrolidine-menadione (**1i**)	-2064900.422	-2065260.217	-1.16
3-Phenethylamine-menadione (**1j**)	-2468645.935	-2469001.713	-1.18
3-Cadaverine-menadione (**1k**)	-2316923.540	-2317277.679	-1.19
3-Histamine-menadione (**1l**)	-2452793.420	-2453147.056	-1.20
3-Tyramine-menadione (**1m**)	-2666347.815	-2666700.568	-1.20
3-Tryptamine-menadione (**1n**)	-2814245.815	-2814600.536	-1.19
3-β-alanine-ethyl ester-menadione (**1o**)	-2563703.952	-2564060.442	-1.18
2-[(*R*)-amino-2-propanol]-naphthoquinone (**2c**)	-2059235.307	-2059585.532	-1.22
2-Aminoethanol-naphthoquinone (**2d**)	-1955906.901	-1956260.011	-1.20
2-Aminopropanol-naphthoquinone (**2e**)	-2059225.308	-2059576.766	-1.21
2-Methylamine-naphthoquinone (**2f**)	-1654915.043	-1655265.870	-1.22
2-Ethylamine-naphthoquinone (**2g**)	-1758235.027	-1758585.599	-1.22

2-Isobutylamine-naphthoquinone (**2h**)	-1964863.475	-1965212.79	-1.23
2-Pyrrolidine-naphthoquinone (**2i**)	-1961580.197	-1961954.33	-1.06
2-Phenehtylamine-naphthoquinone (**2j**)	-2365364.924	-2365715.271	-1.22
2-Cadaverine-naphthoquinone (**2k**)	-2213641.074	-2213990.897	-1.22
2-Histamine-naphthoquinone (**2l**)	-2349511.494	-2349863.225	-1.21
2-Tyramine-naphthoquinone (**2m**)	-2563065.161	-2563414.751	-1.22
2-Tryptamine-naphthoquinone (**2n**)	-2711013.783	-2711363.998	-1.22
3-Aminopropanol-DHNA methyl ester (**4e**)	-2661129.465	-2661498.015	-1.10
3-Methylamine-DHNA methyl ester (**4f**)	-2253661.779	-2254032.94	-1.08
3-Ethylamine-DHNA methyl ester (**4g**)	-2356954.835	-2357344.83	-0.95
3-Isobutylamine-DHNA methyl ester (**4h**)	-2563584.265	-2563970.818	-0.98
3-Pyrrolidine-DHNA methyl ester (**4i**)	-2560327.178	-2560736.245	-0.83
3-Phenehtylamine-DHNA methyl ester (**4j**)	-2964089.835	-2964481.381	-0.94
3-Cadaverine-DHNA methyl ester (**4k**)	-2812345.366	-2812741.045	-0.92
3-Histamine-DHNA methyl ester (**4l**)	-2948235.185	-2948625.998	-0.95
3-Tyramine-DHNA methyl ester (**4m**)	-3161773.515	-3162168.243	-0.92
3-Tryptamine-DHNA methyl ester (**4n**)	-3206468.239	-3206863.332	-0.92
3-β-alanine-ethyl ester-DHNA methyl ester (**4o**)	-3059148.76	-3059545.687	-0.91
3-Taurine-DHNA methyl ester ground (**4p**)	-3995934.091	-3996332.673	-0.90

e. Supplementary Figures

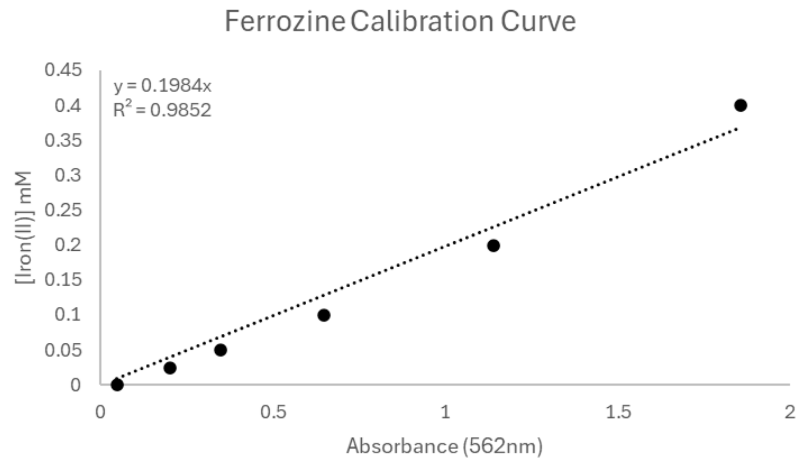

Figure S1: The average absorbance at 562 nM in triplicate vs concentration of ferrous sulfate heptahydrate serial dilutions with ferrozine (y = 0.1984x; R^2: 0.99) was used to find concentration of iron(II) present at the completion of the iron(III) oxide nanoparticle reduction assays.

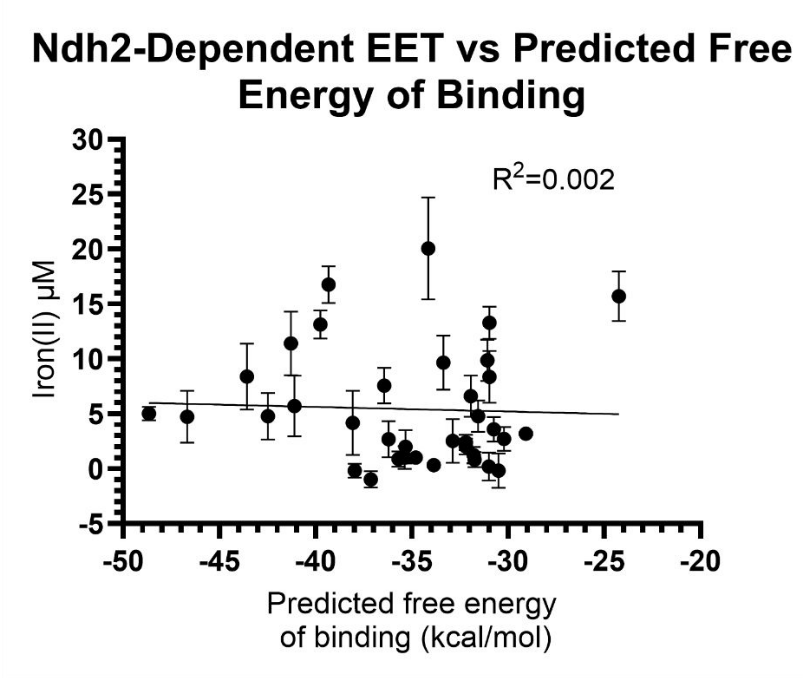

Figure S2: *L. plantarum* Ndh2-dependent EET vs free energy of binding. Error bars represent the standard deviation of the biological triplicates (n = 3). The linear best-fit lines were calculated using GraphPad's simple linear regression model (Version 10.1.0).

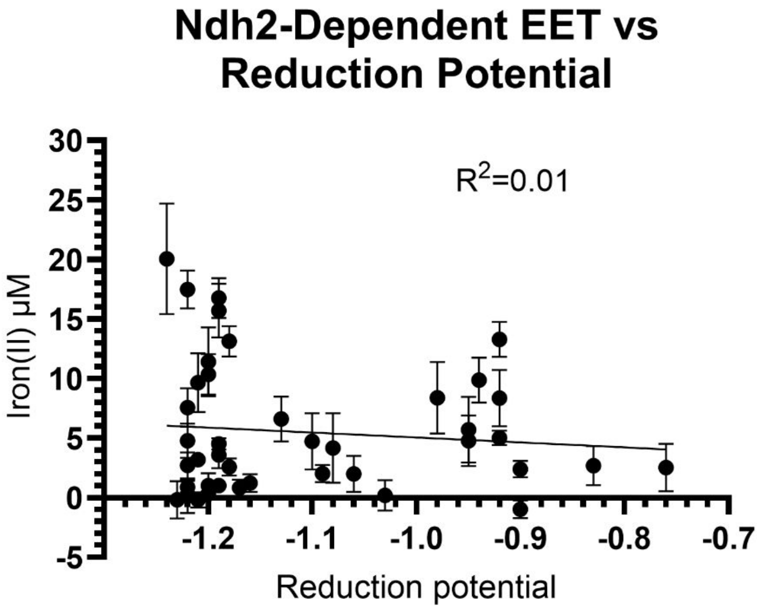

Figure S3: *L. plantarum* Ndh2-dependent EET vs predicted 1e$^-$ reduction potential. Error bars represent the standard deviation of the biological triplicates (n = 3). The linear best-fit lines were calculated using GraphPad's simple linear regression model (Version 10.1.0).

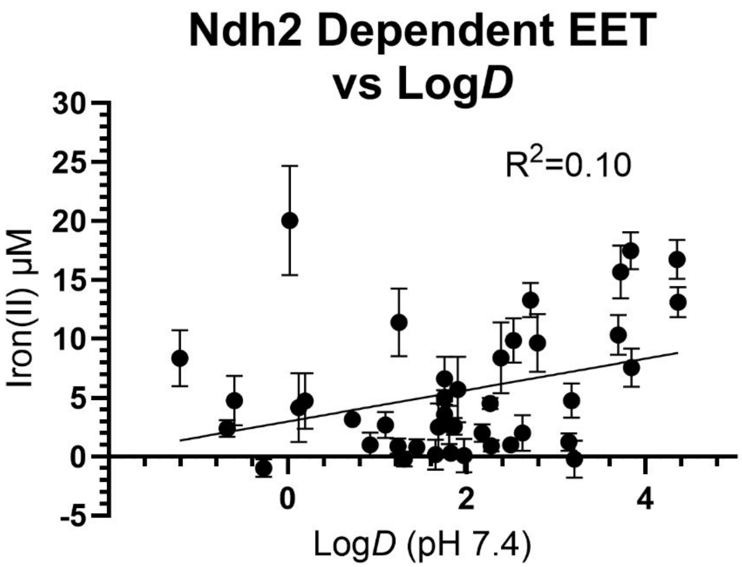

Figure S4: *L. plantarum* Ndh2-dependent EET vs Log*D*. Error bars represent the standard deviation of the biological triplicates (n = 3). The linear best-fit lines were calculated using GraphPad's simple linear regression model (Version 10.1.0).

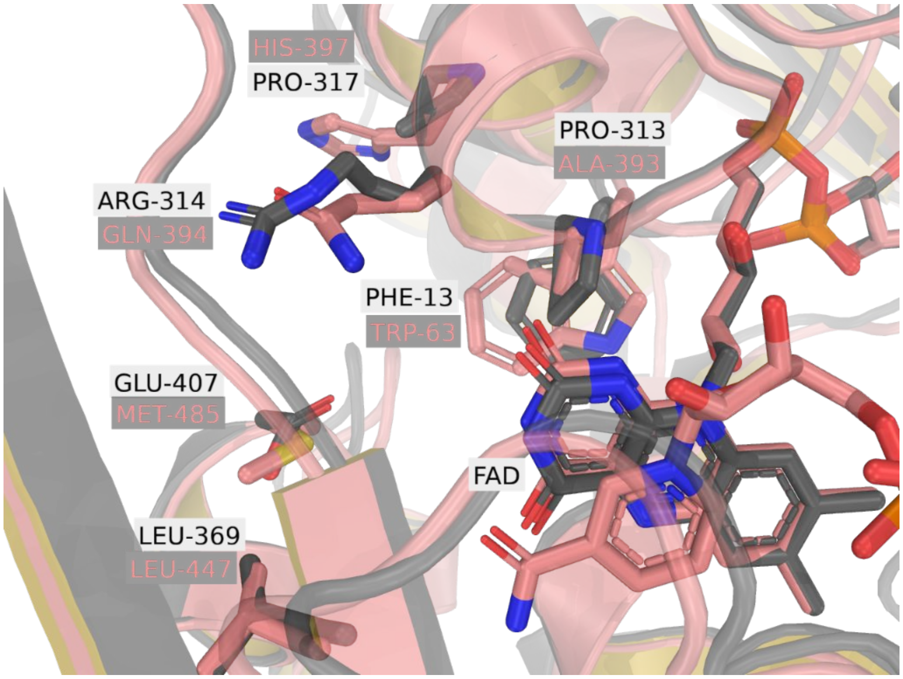

Figure S5: Homology model (grey) of *L. plantarum* Ndh2 overlaid with the S. cerevisiae Ndh2 (salmon, PDB ID: 4G73). Key binding site residues and FAD are highlighted and labeled.

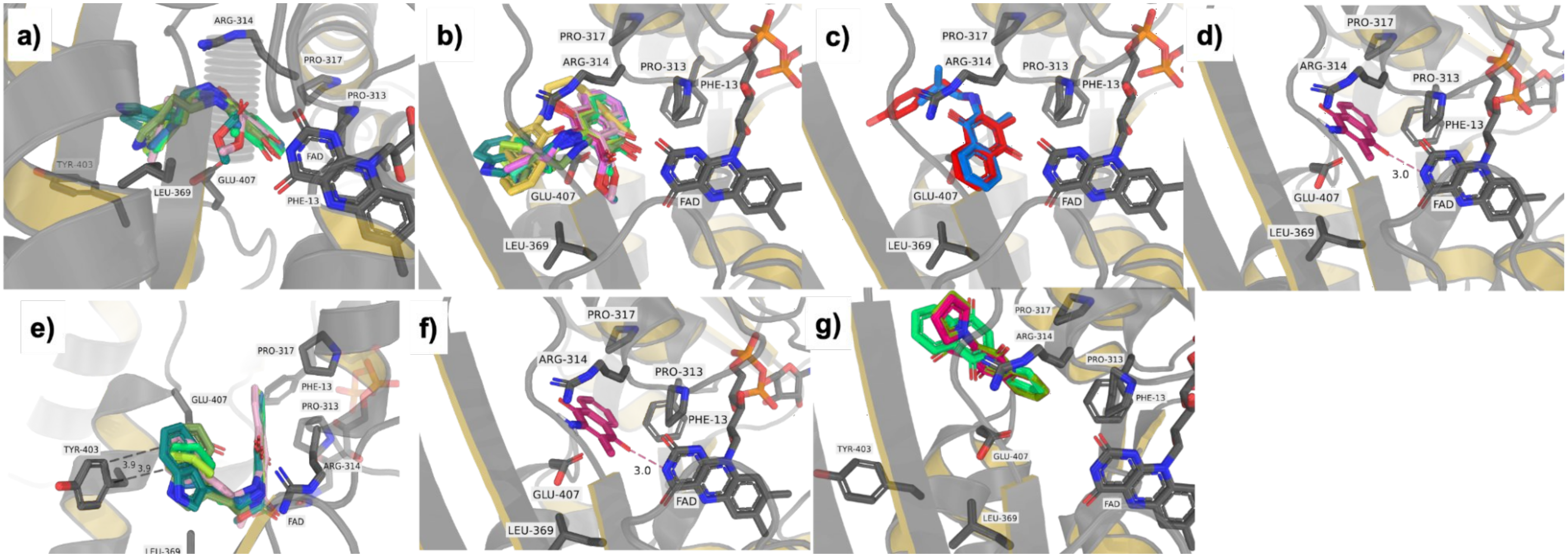

Figure S6: Docked poses of biogenic amine compounds in the homology model of Ndh2 from *L. plantarum*. **a)** Docked poses situated in the binding site between an arginine-glutamate clamp, highlighted with discs; **b)** Docked pose cluster including several aromatic amines (**1a**, **1g**, **1j**, **1l**, **1n**, **2j**, **2n**, **4h**, **4j**, **4k**, and **4n**); **c)** Docked pose cluster of top-performing compounds assuming orientations differing from the populated cluster in **A** (**1m**, **1h**); **d)** The top *in vivo*-performing compound **1a** docked in the binding site with proximity of the carbonyl oxygen and FAD highlighted with dashes; **e)** Aromatic amine tails proximal to TYR-403 with potential π-π stacking interaction highlighted with dashes; **f)** Docked poses of polar amine compounds, **1e** and **4e**, displaced from the canonical binding site; **g)** Docked poses of rigid amine compounds **1i**, **2i**, and **4i** displaced from the canonical binding site

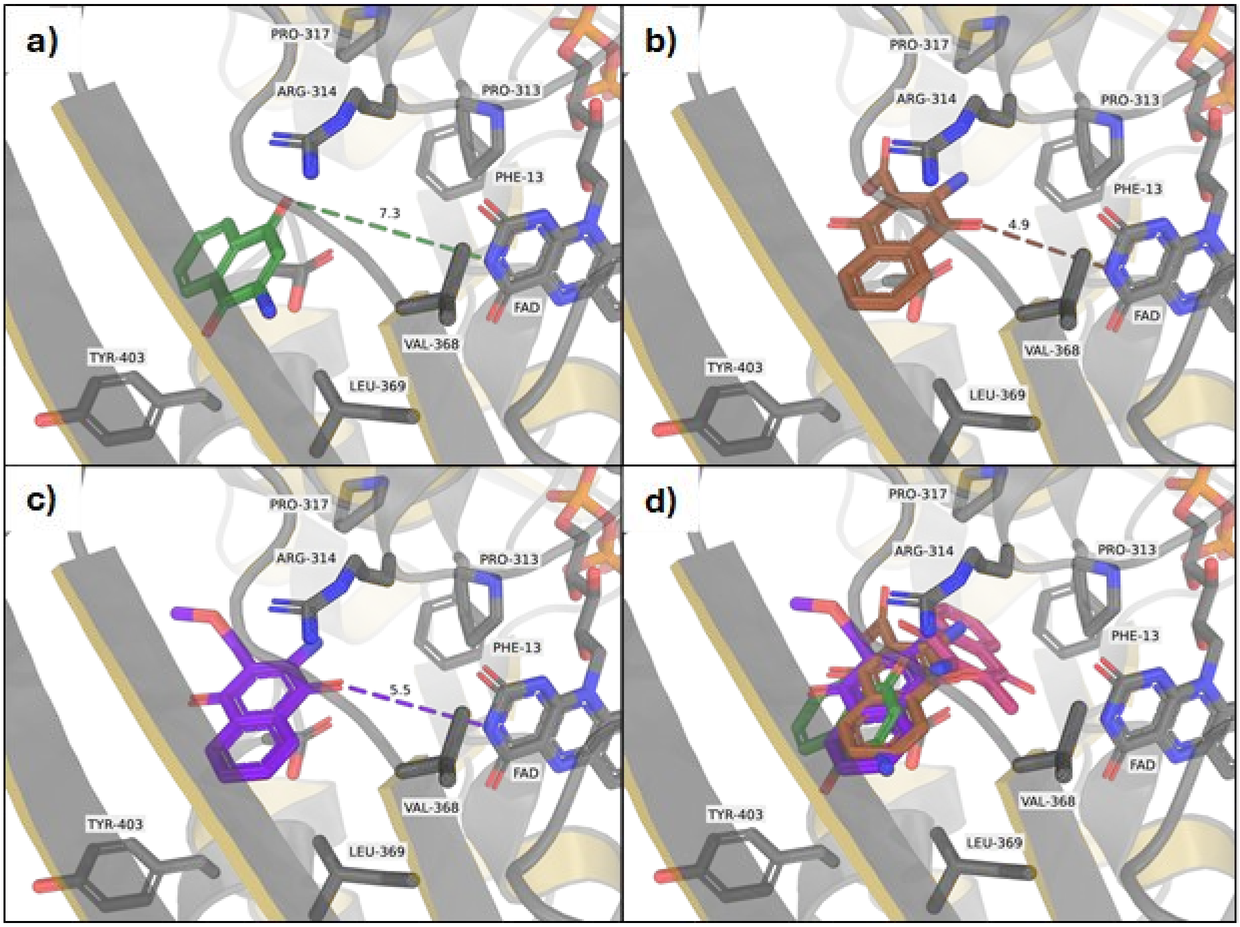

Figure S7: Comparison of activity profile of mediators with a primary amine (**1a**, **2a**, **3a**, and **4a**). **a-c)** docked poses of compounds **2a** (**a**), **3a** (**b**), and **4a** (**c**) situated in the binding site between the Arg-Glu clamp with the carbonyl group oriented towards FAD and the ring system toward Tyr-403.

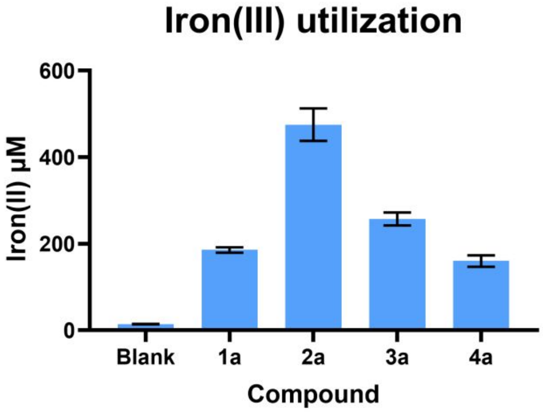

Figure S8: Comparison of activity profile of mediators with a primary amine (**1a**, **2a**, **3a**, and **4a**)

f. NMR Spectra

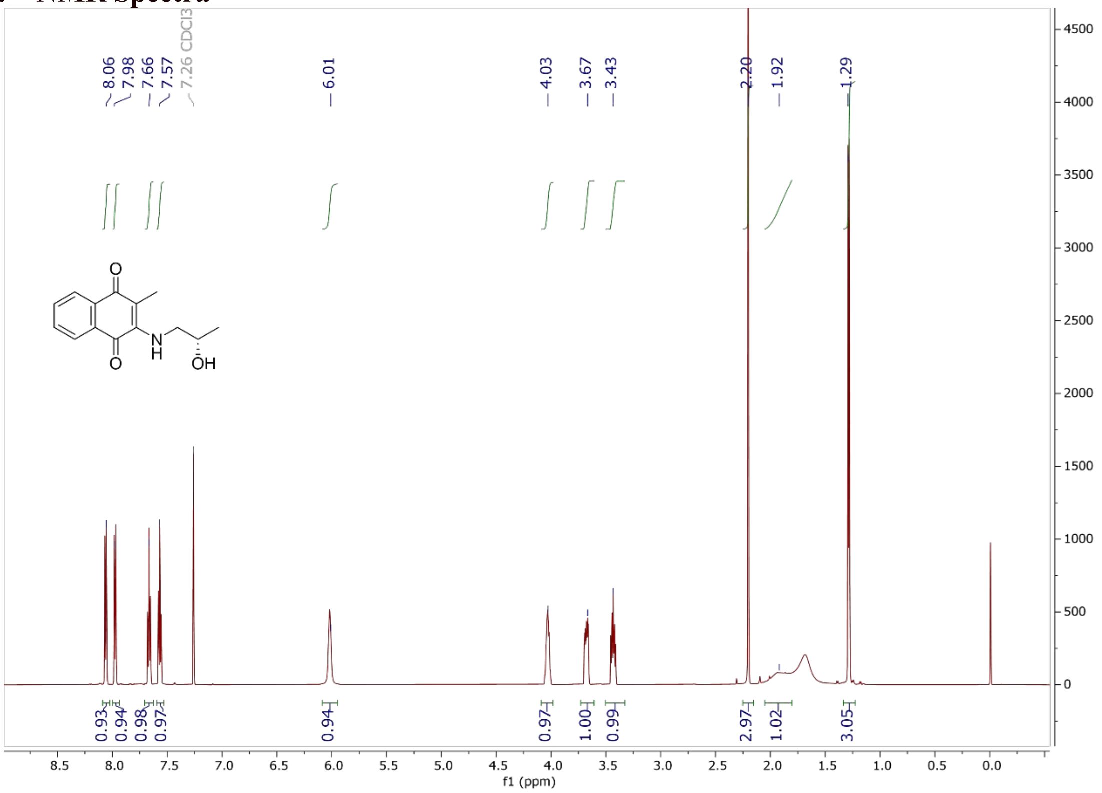

Figure S9: ^{1}H NMR Spectra (500 MHz) for 3-[(*S*)-amino-2-propanol]-menadione (**1b**) in $CDCl_3$

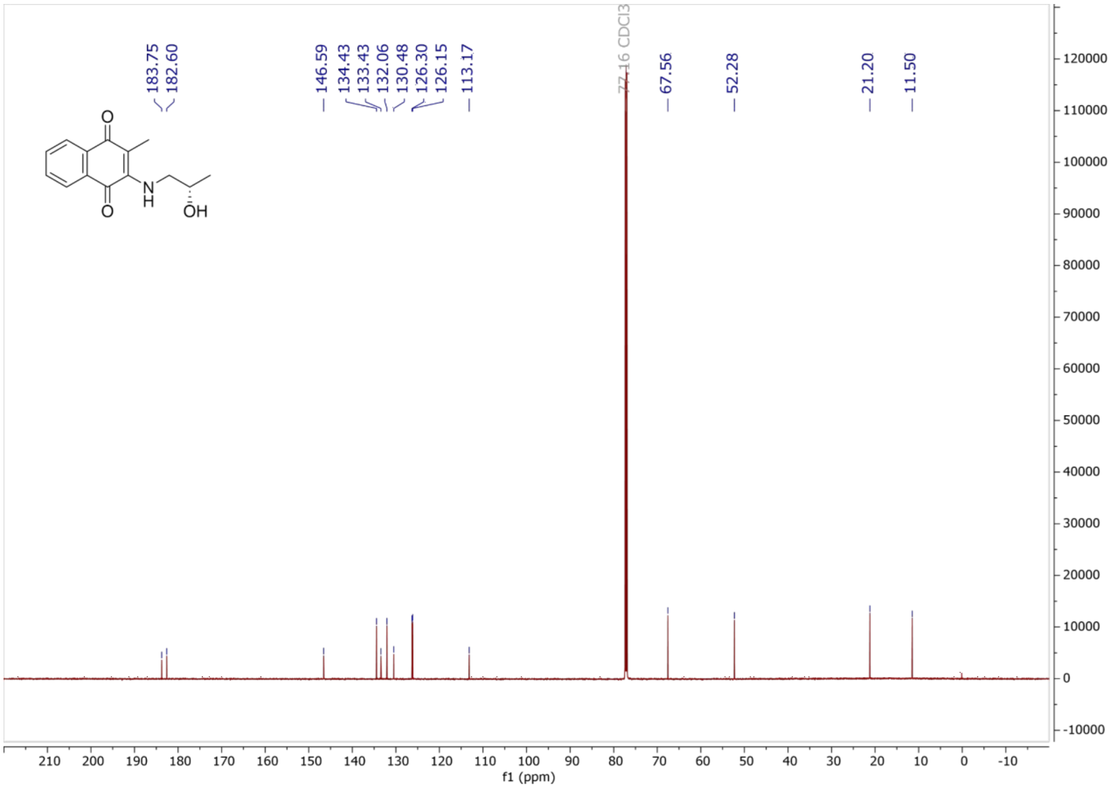

Figure S10: ^{13}C NMR Spectra (125 MHz) for 3-[(*S*)-amino-2-propanol]-menadione (**1b**) in $CDCl_3$

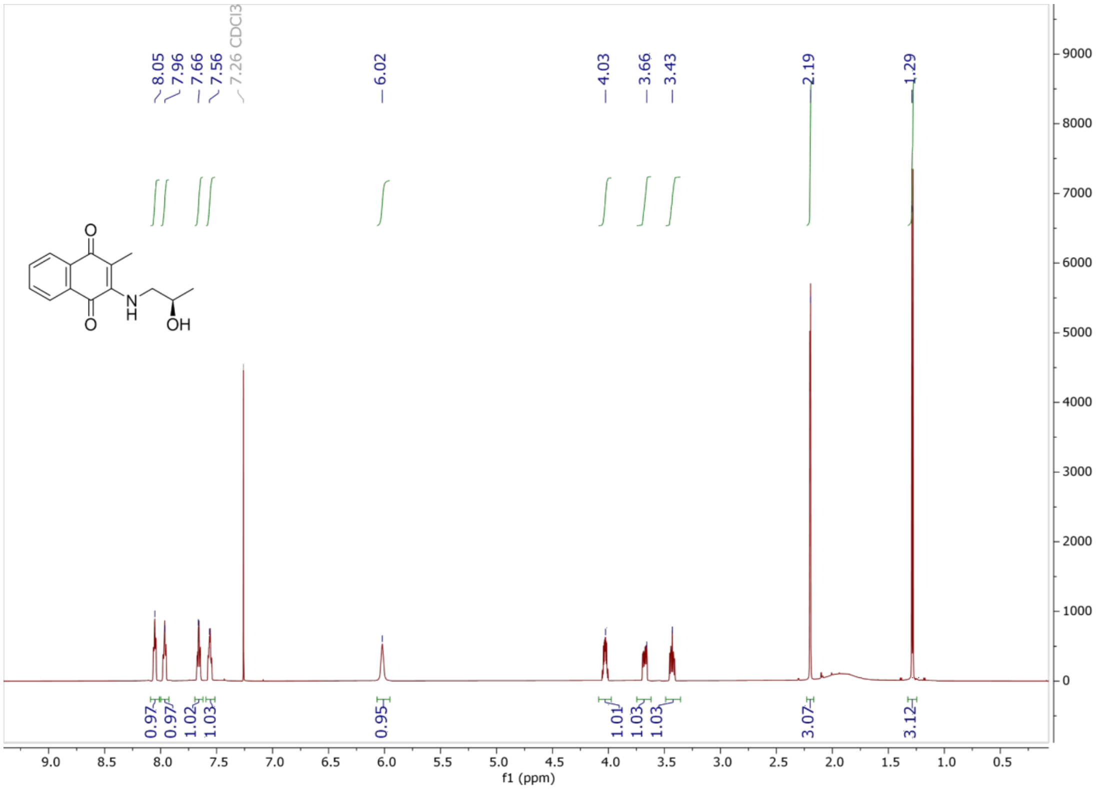

Figure S11: ^{1}H NMR Spectra (500 MHz) for 3-[(*R*)-amino-2-propanol]-menadione (**1c**) in $CDCl_3$

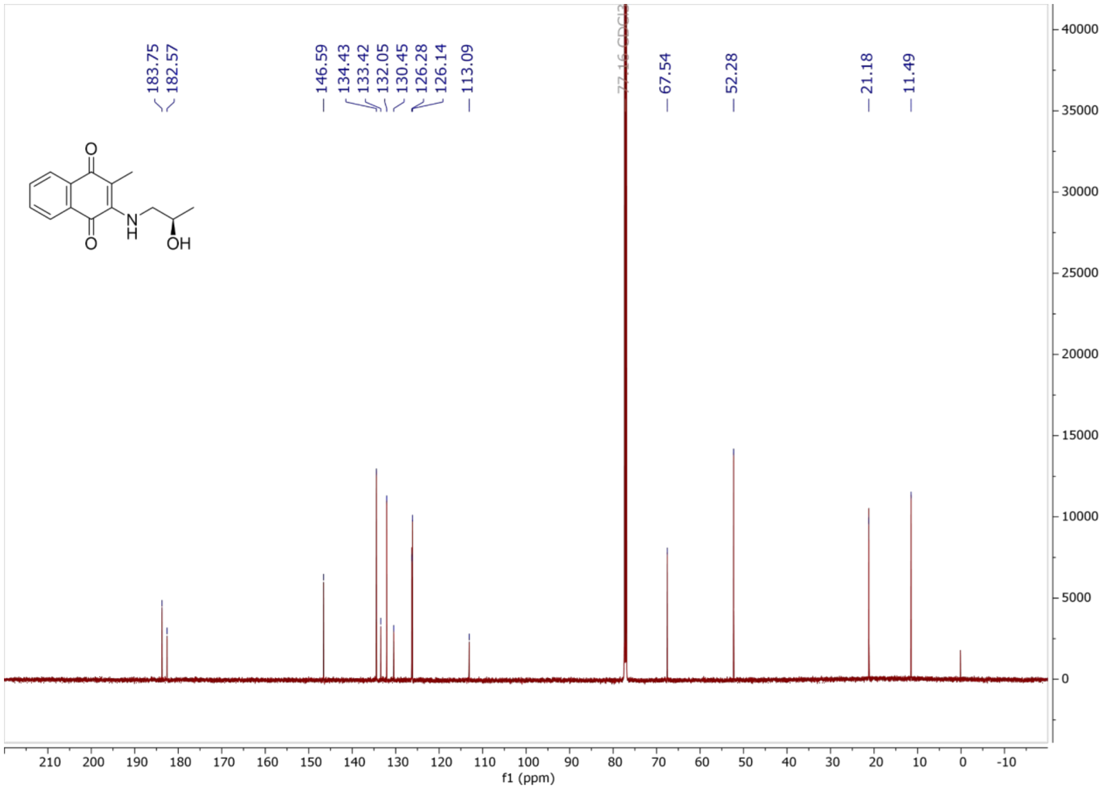

Figure S12: ^{13}C NMR Spectra (125 MHz) for 3-[(*R*)-amino-2-propanol]-menadione (**1c**) in $CDCl_3$

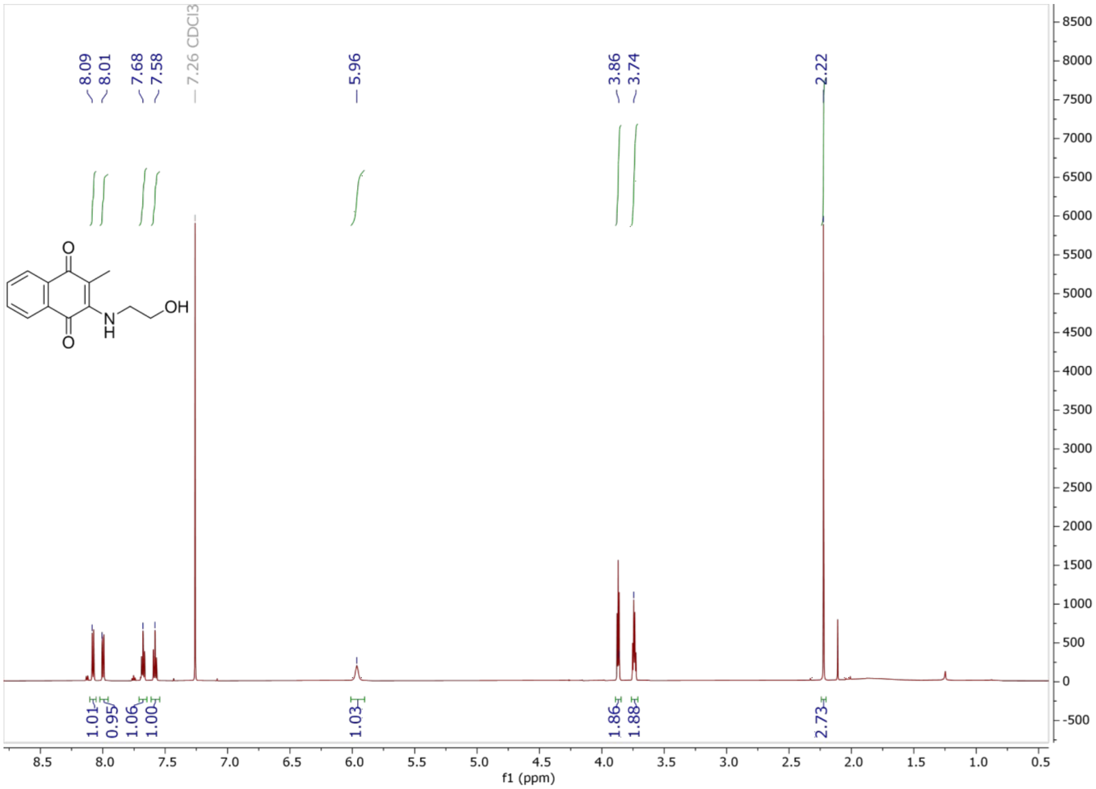

Figure S13: ^{1}H NMR Spectra (500 MHz) for 3-Aminoethanol-menadione (**1d**) in $CDCl_3$

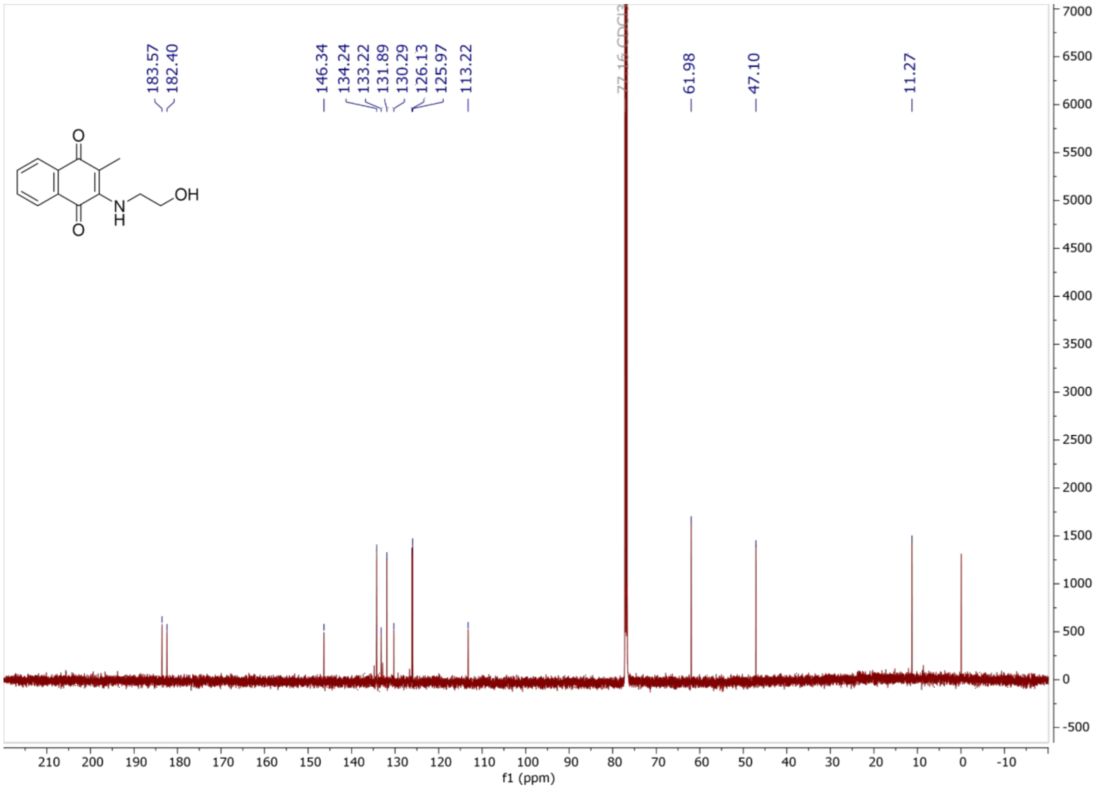

Figure S14: ^{13}C NMR Spectra (125 MHz) for 3-Aminoethanol-menadione (**1d**) in $CDCl_3$

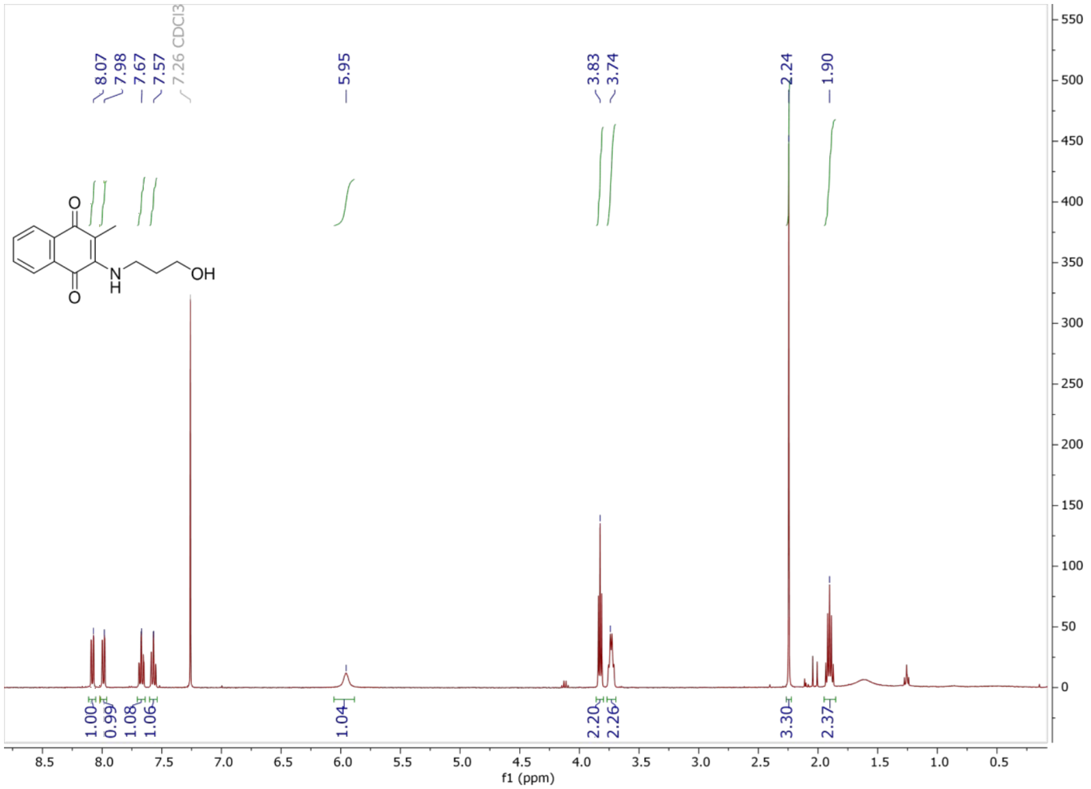

Figure S15: ^{1}H NMR Spectra (500 MHz) for 3-Aminopropanol menadione (**1e**) in $CDCl_3$

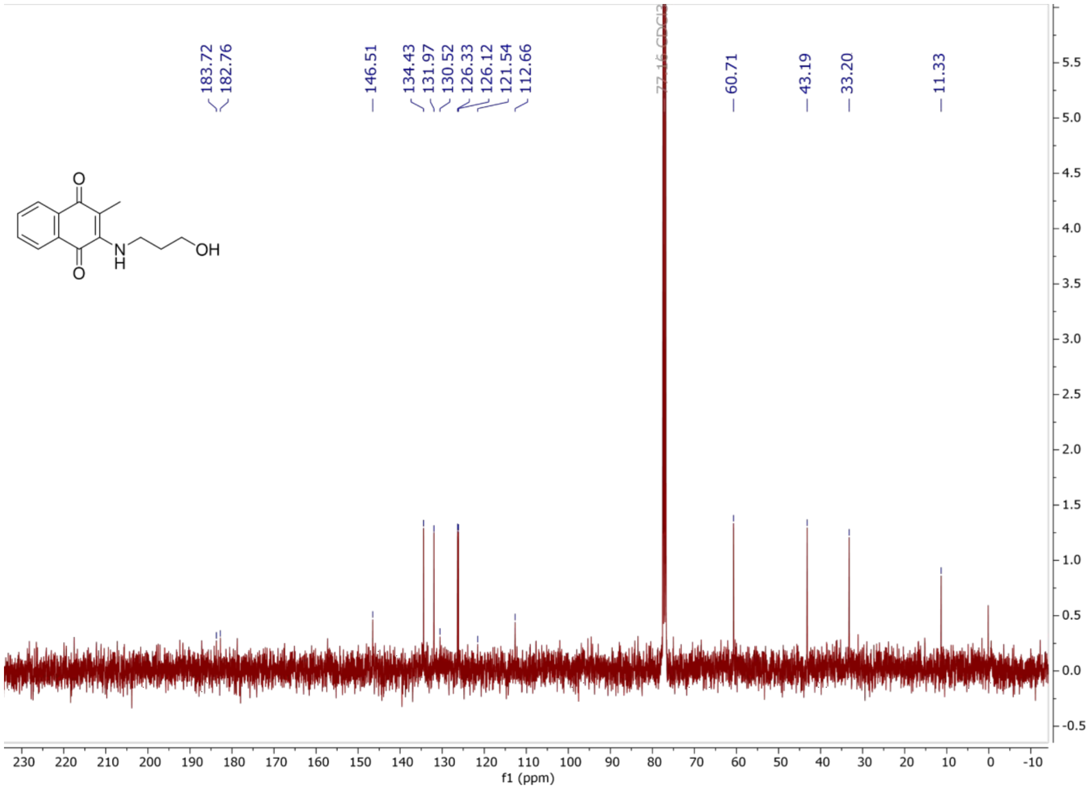

Figure S16: ^{13}C NMR Spectra (125 MHz) for 3-Aminopropanol menadione (**1e**) in $CDCl_3$

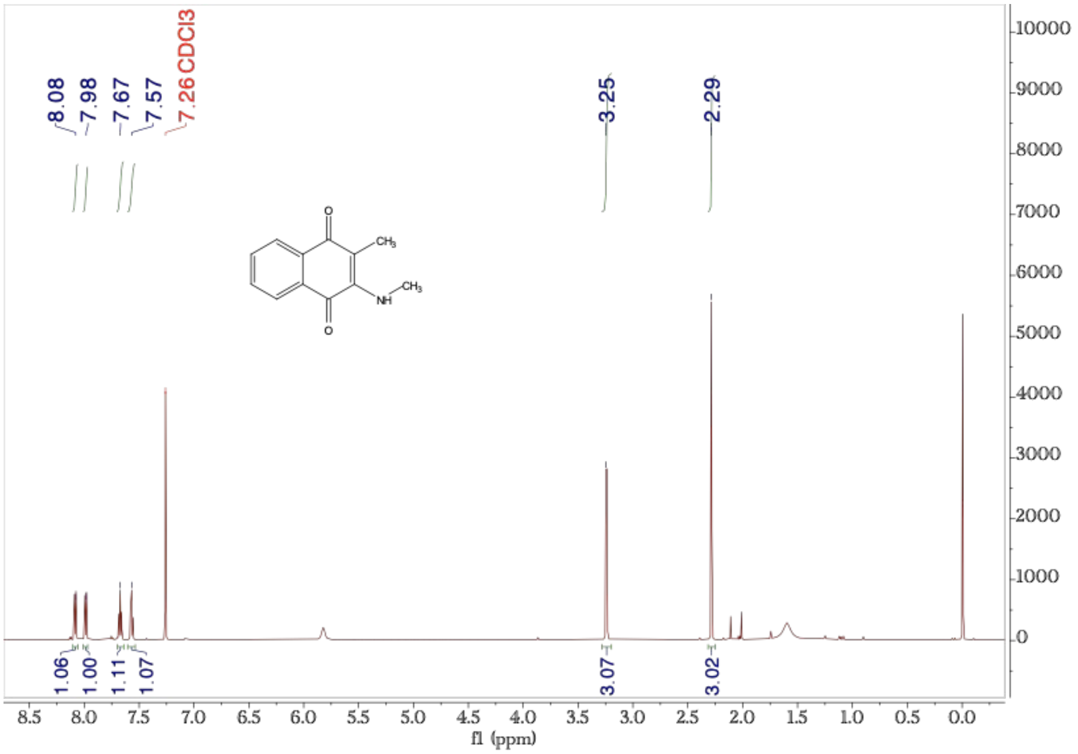

Figure S17: ^{1}H NMR Spectra (400 MHz) for 3-Methylamine-menadione (**1f**) in $CDCl_3$

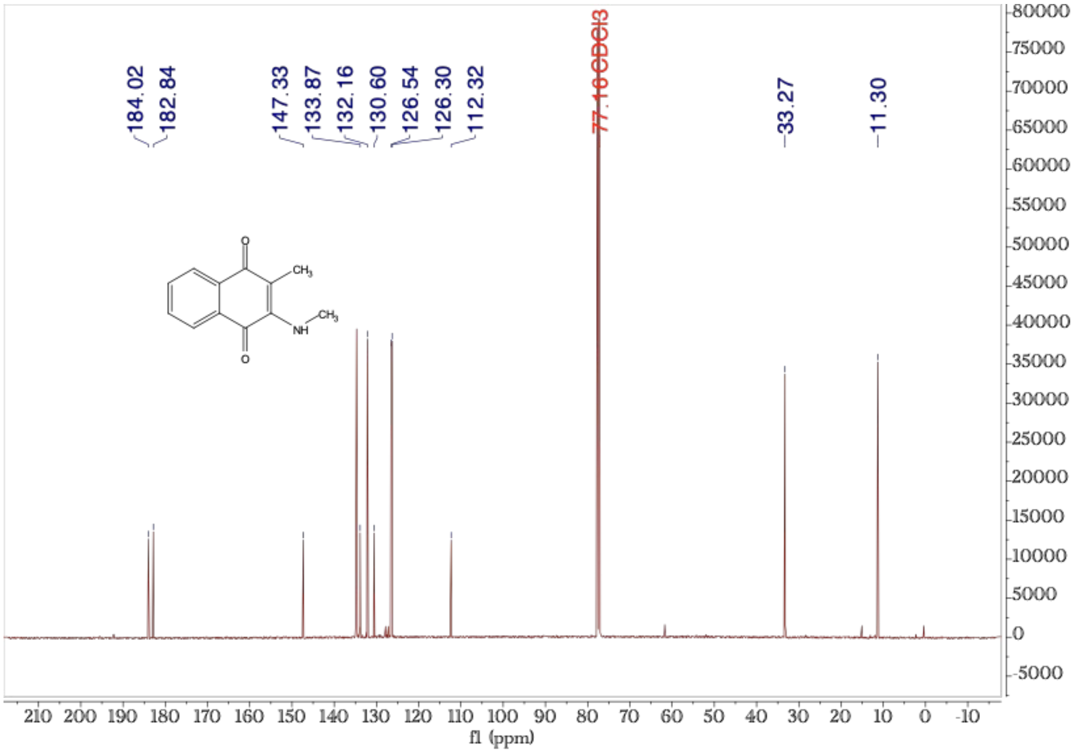

Figure S18: ^{13}C NMR Spectra (125 MHz) for 3-Methylamine-menadione (**1f**) in $CDCl_3$

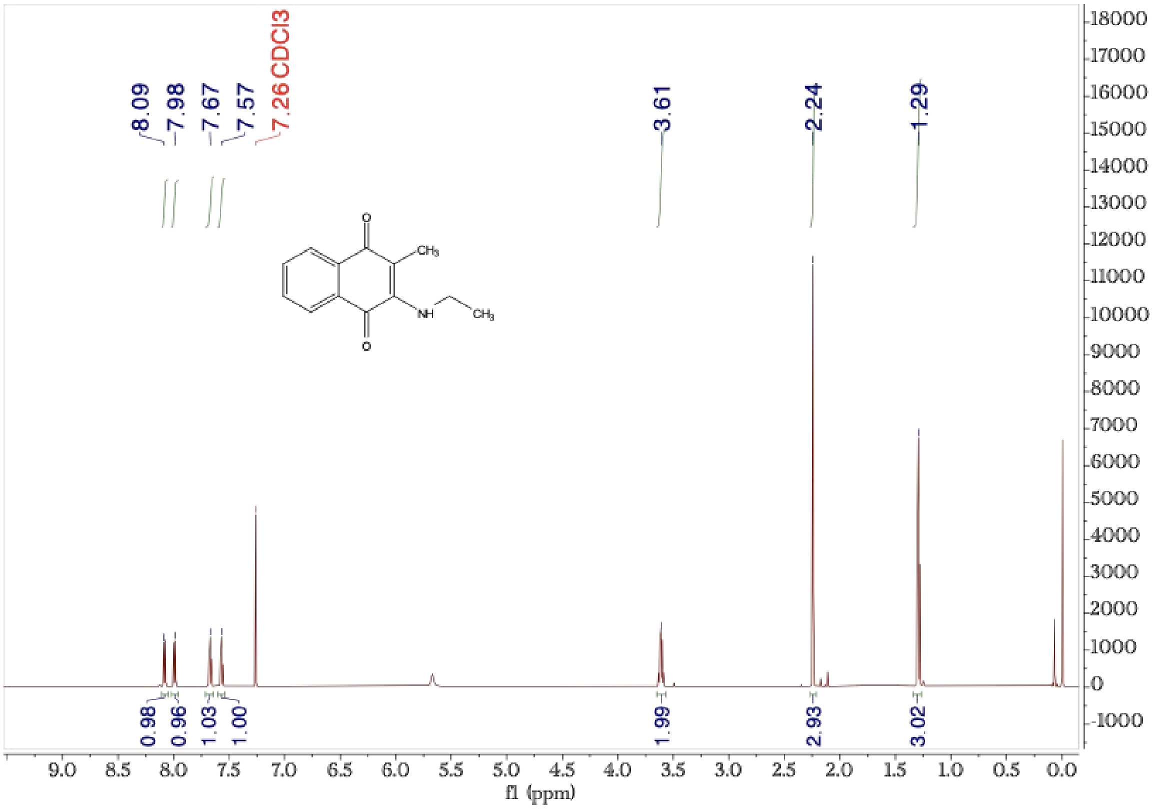

Figure S19: ^{1}H NMR Spectra (400 MHz) for 3-Ethylamine-menadione (**1g**) in $CDCl_3$

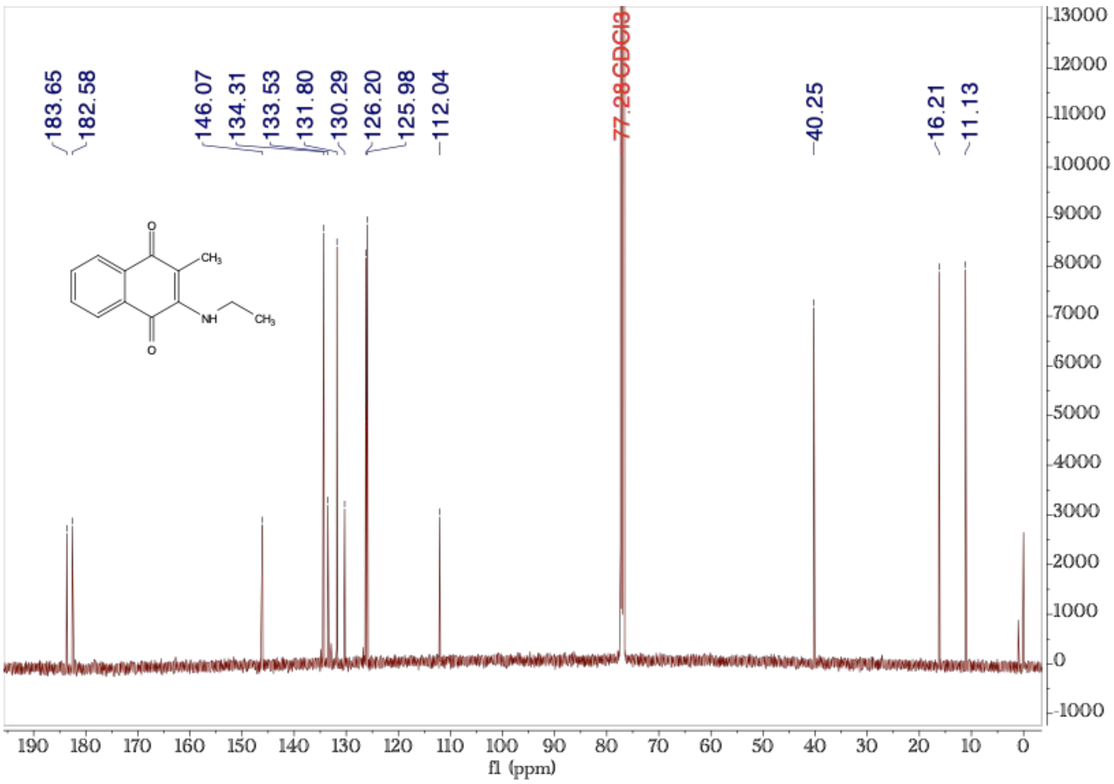

Figure S20: ^{13}C NMR Spectra (125 MHz) for 3-Ethylamine-menadione (**1g**) in $CDCl_3$

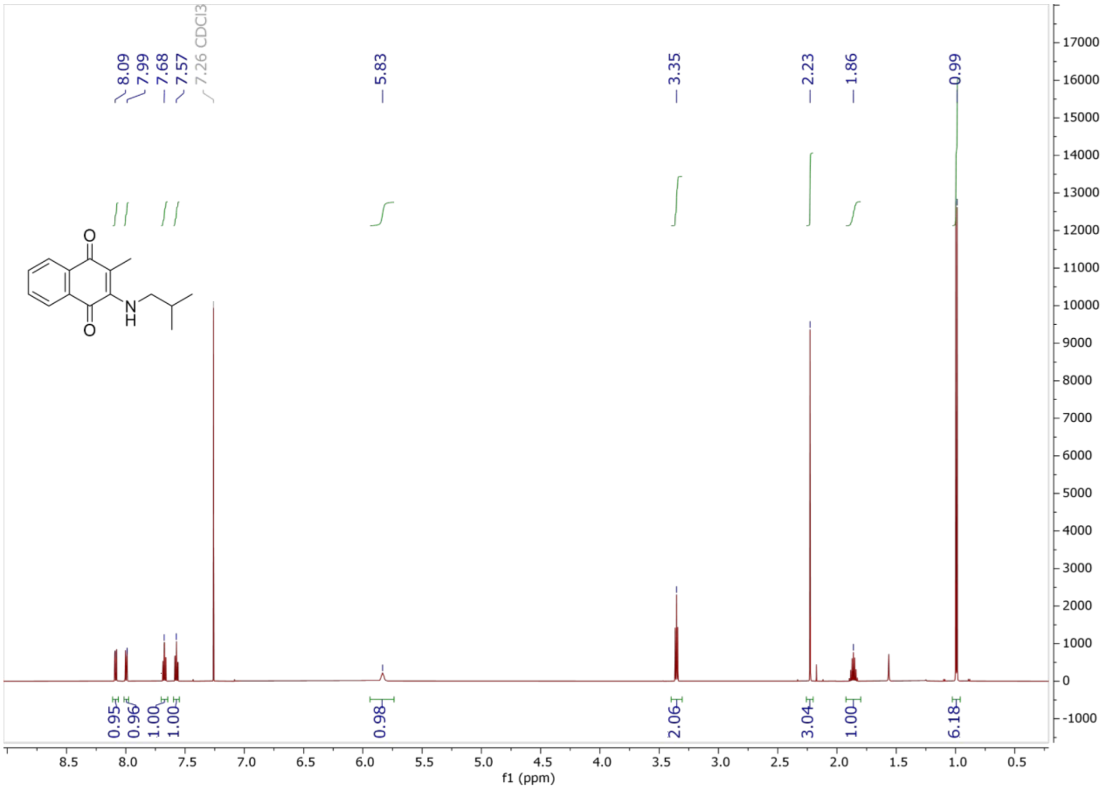

Figure S21: ^{1}H NMR Spectra (500 MHz) for 3-Isobutylamine-menadione (**1h**) in $CDCl_3$

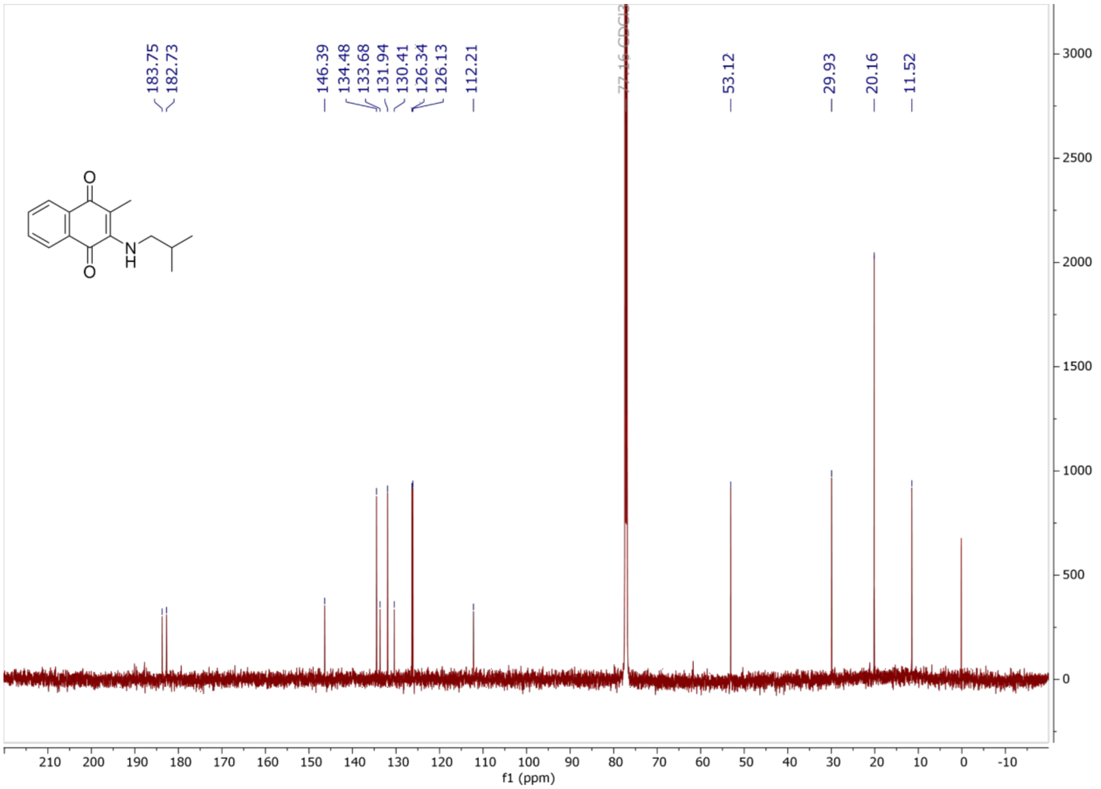

Figure S22: ^{13}C NMR Spectra (125 MHz) for 3-Isobutylamine-menadione (**1h**) in $CDCl_3$

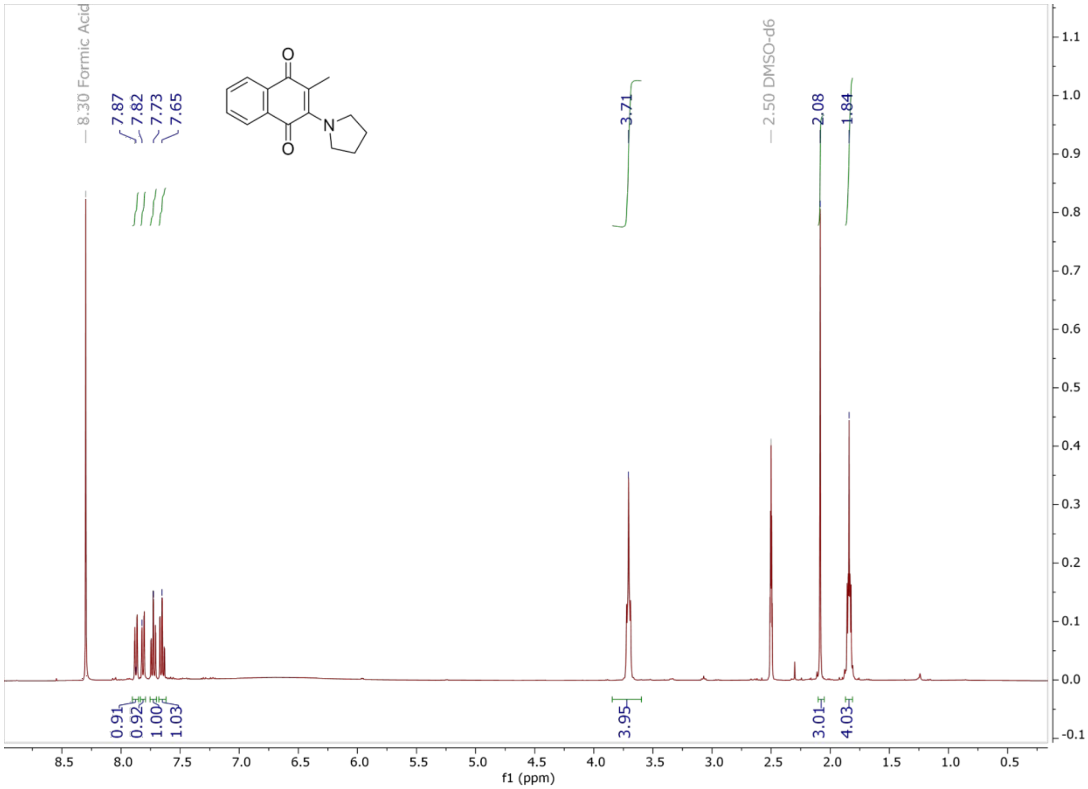

Figure S23: ^{1}H NMR Spectra (500 MHz) for 3-Pyrrolidine-menadione (**1i**) in $(CD_3)_2SO$

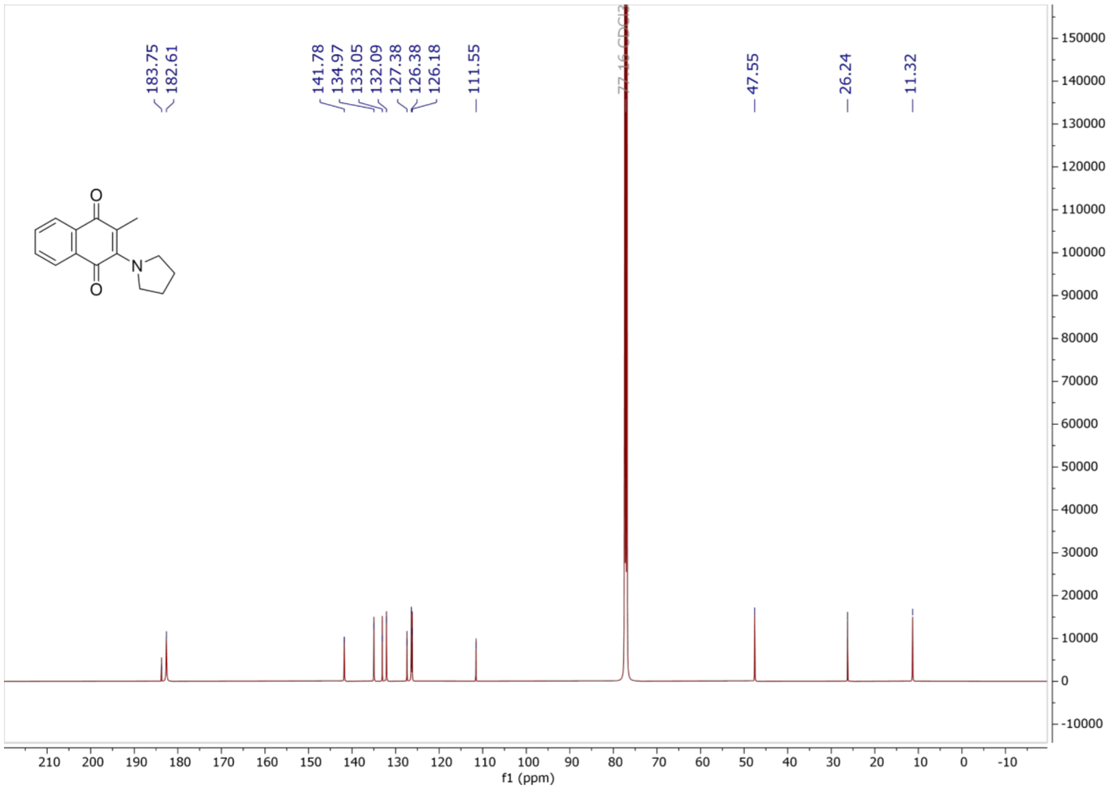

Figure S24: ^{13}C NMR Spectra (125 MHz) for 3-Pyrrolidine-menadione (**1i**) in $CDCl_3$

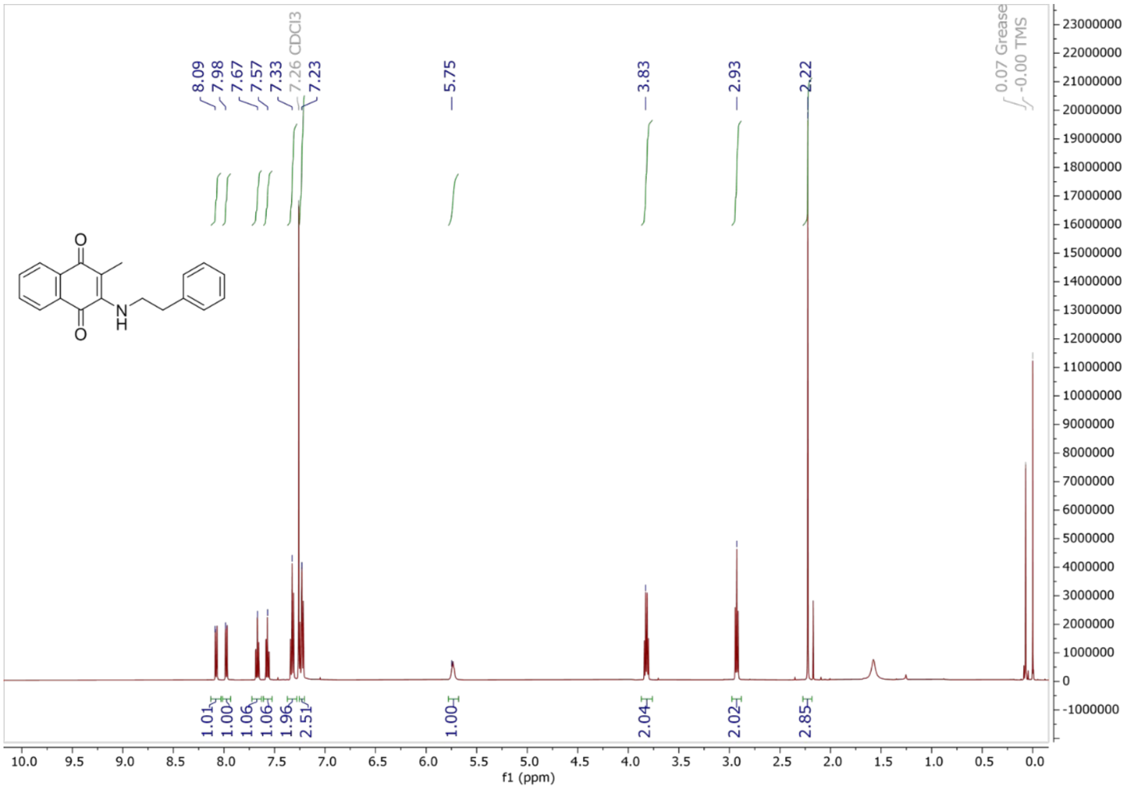

Figure S25: ^{1}H NMR Spectra (500 MHz) for 3-Phenethylamine-menadione (**1j**) in $CDCl_3$

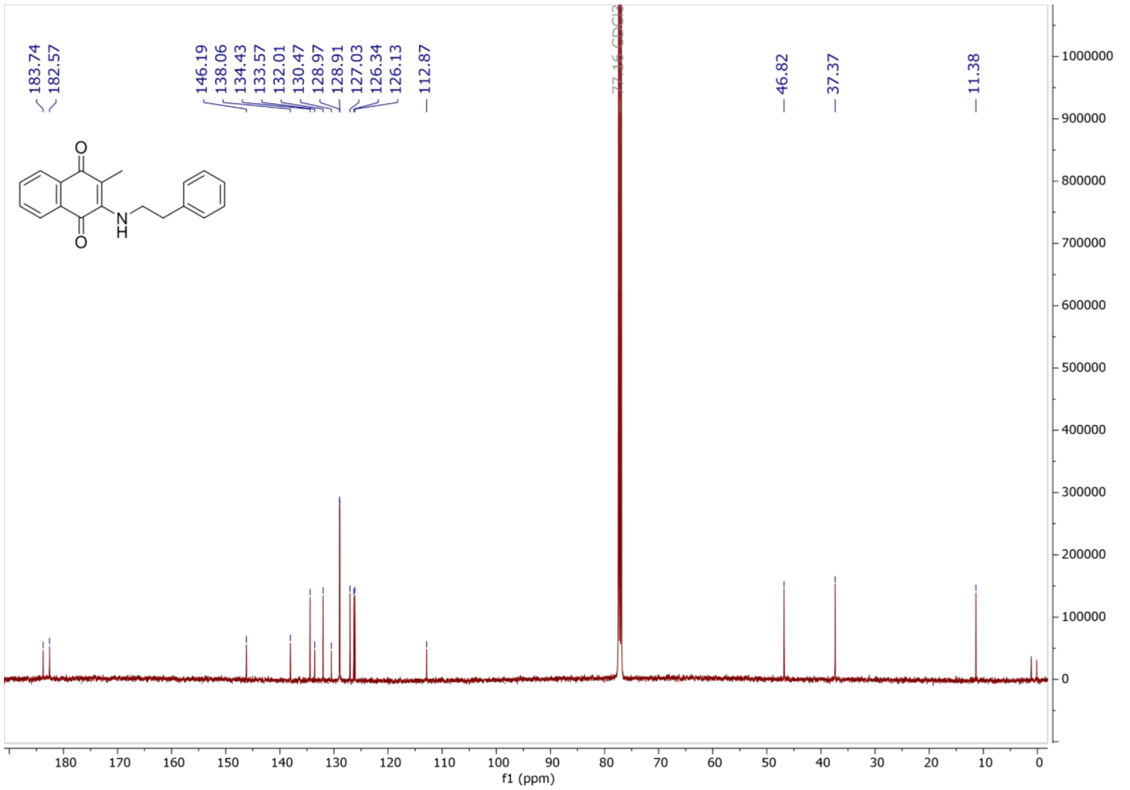

Figure S26: ^{13}C NMR Spectra (125 MHz) for 3-Phenethylamine-menadione (**1j**) in $CDCl_3$

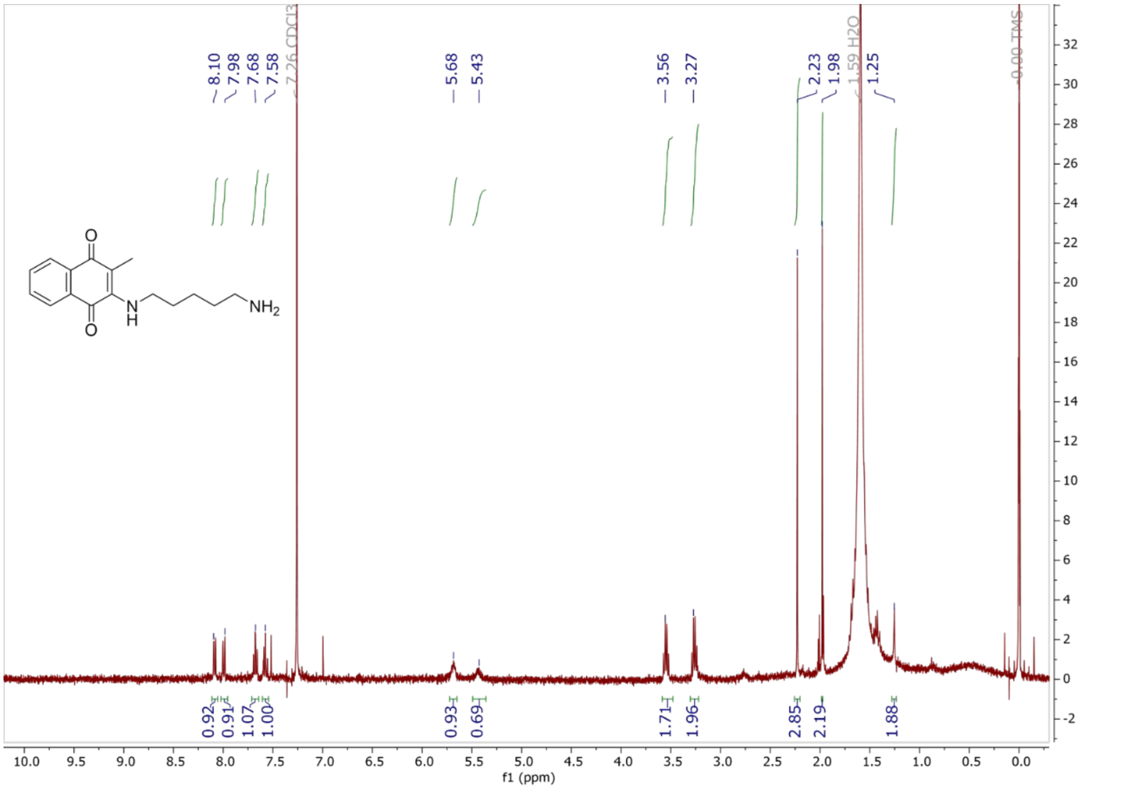

Figure S27: ^{1}H NMR Spectra (500 MHz) for 3-Cadaverine-menadione (**1k**) in $CDCl_3$

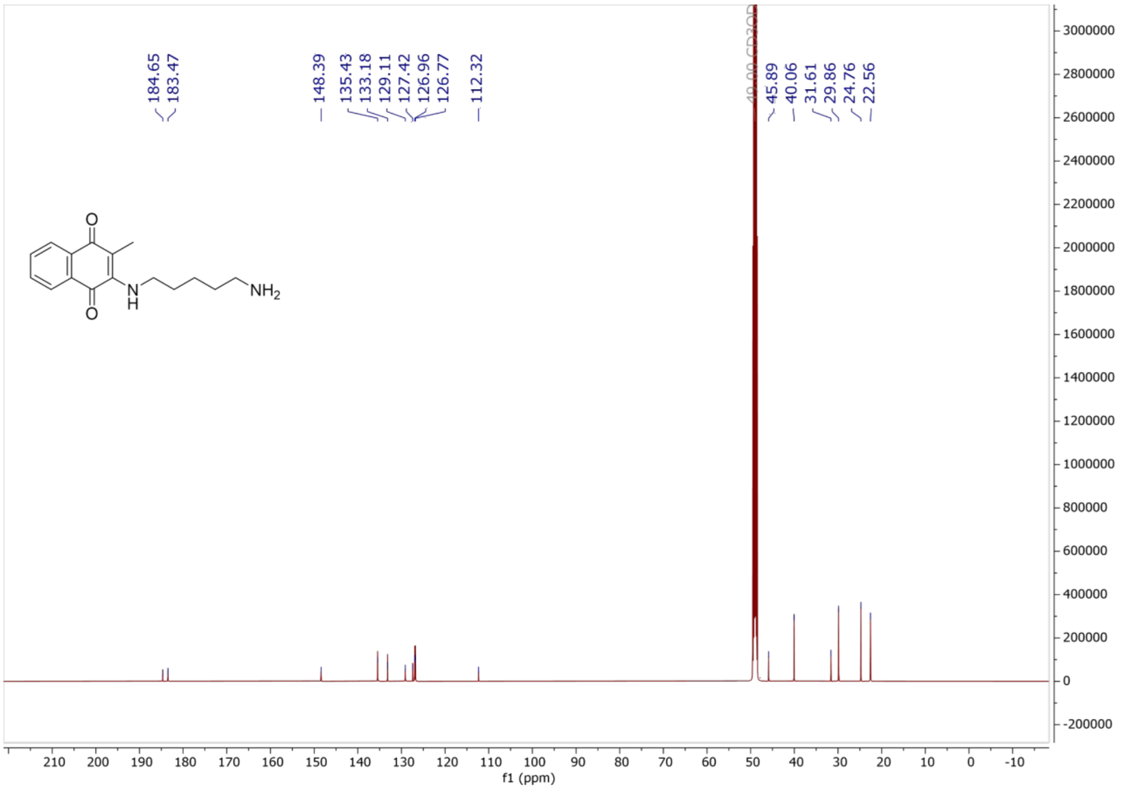

Figure S28: ^{13}C NMR Spectra (125 MHz) for 3-Cadaverine-menadione (**1k**) in CD_3OD

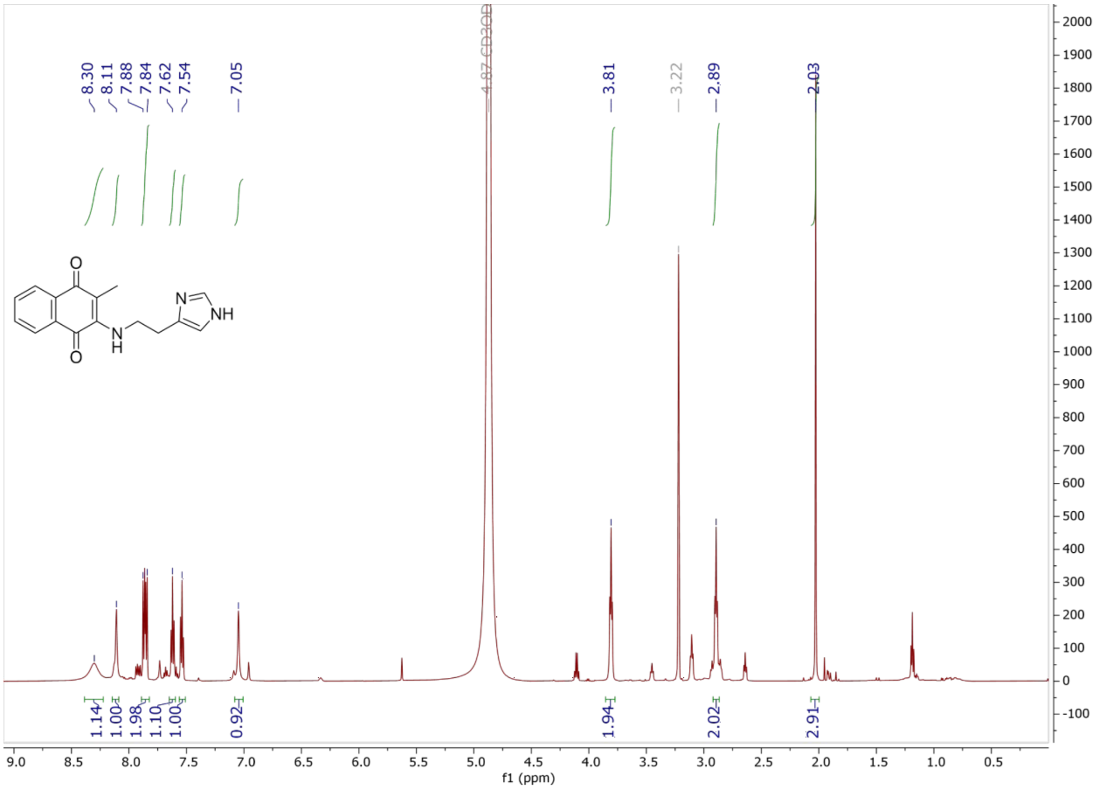

Figure S29: ^{1}H NMR Spectra (500 MHz) for 3-Histamine-menadione (**1l**) in CD_3OD

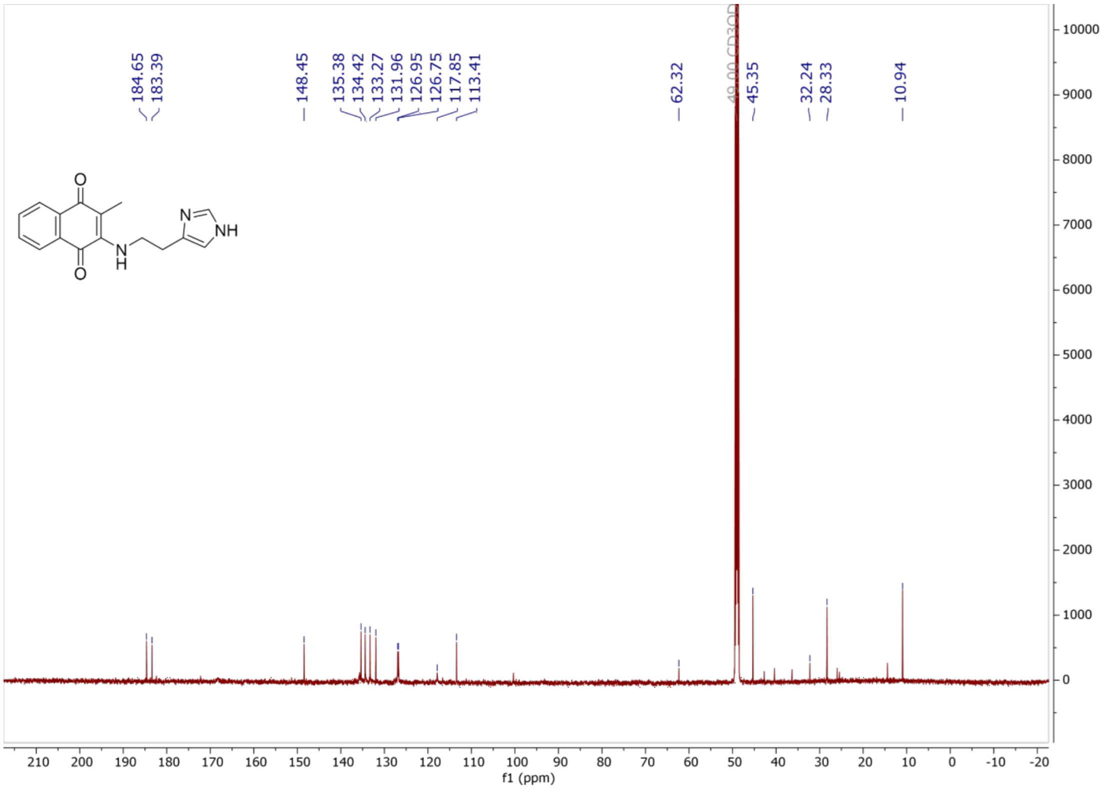

Figure S30: ^{13}C NMR Spectra (125 MHz) for 3-Histamine-menadione (**1l**) in CD_3OD

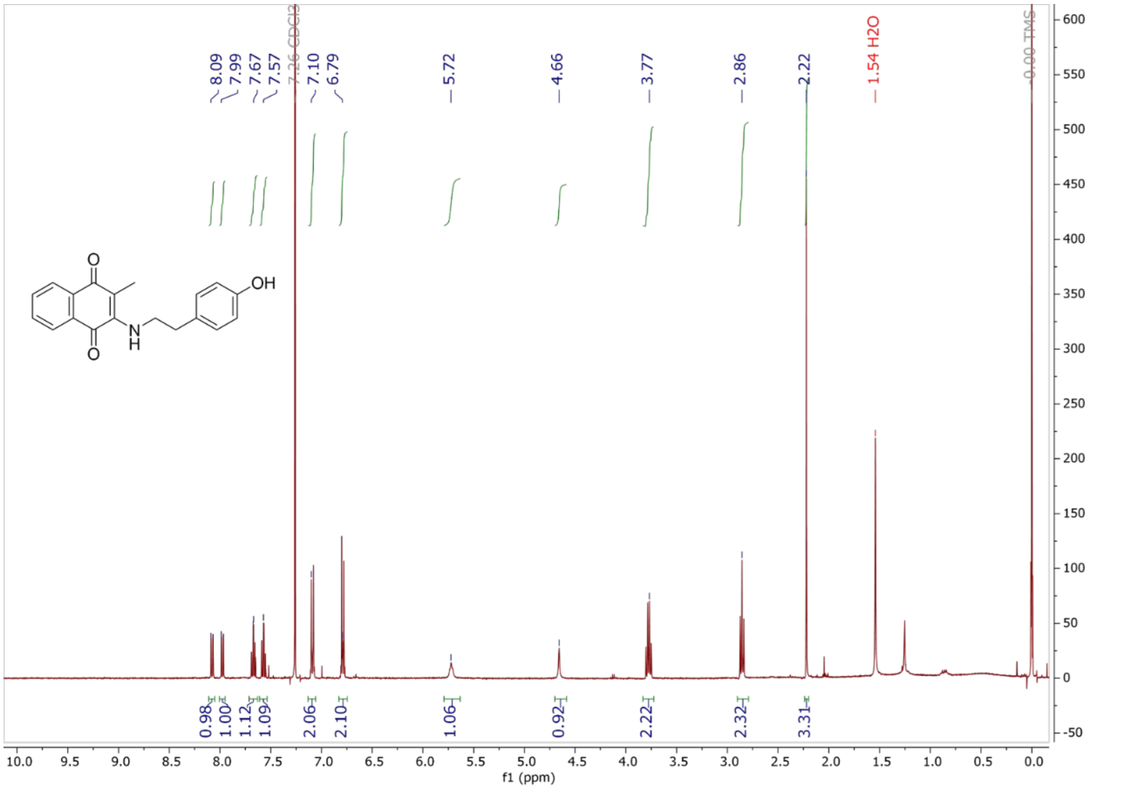

Figure S31: ^{1}H NMR Spectra (500 MHz) for 3-Tyramine-menadione (**1m**) in $CDCl_3$

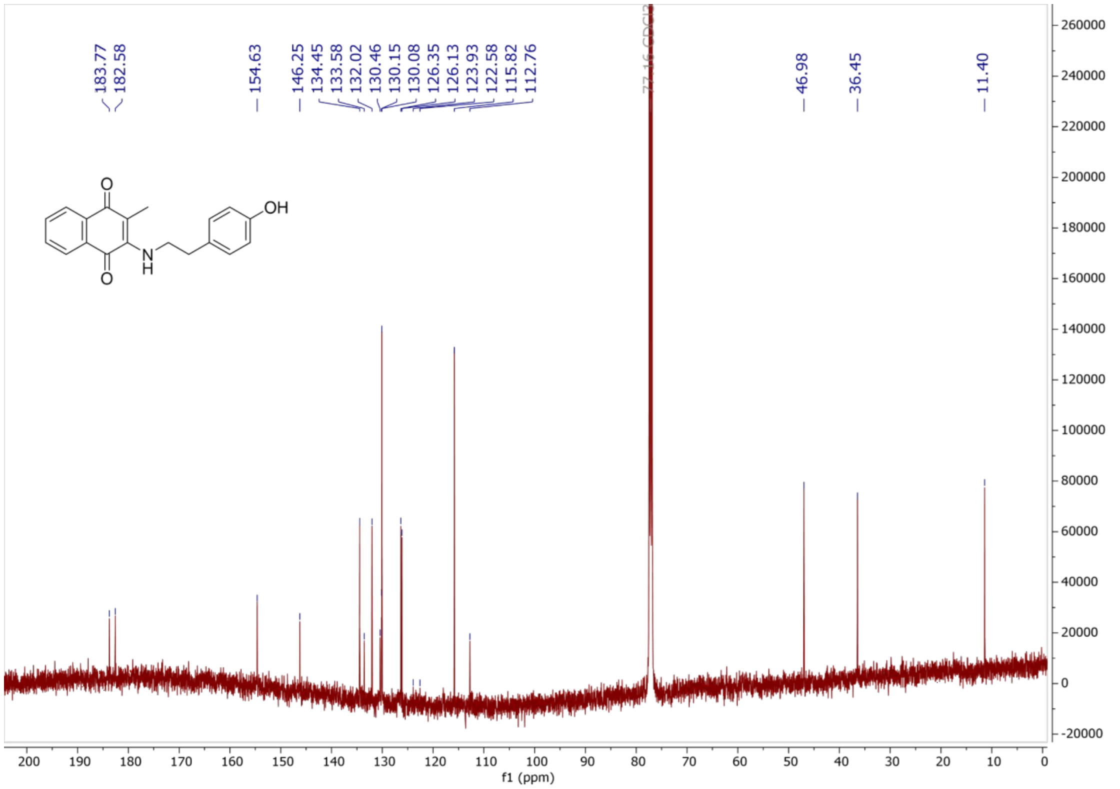

Figure S32: ^{13}C NMR Spectra (125 MHz) for 3-Tyramine-menadione (**1m**) in $CDCl_3$

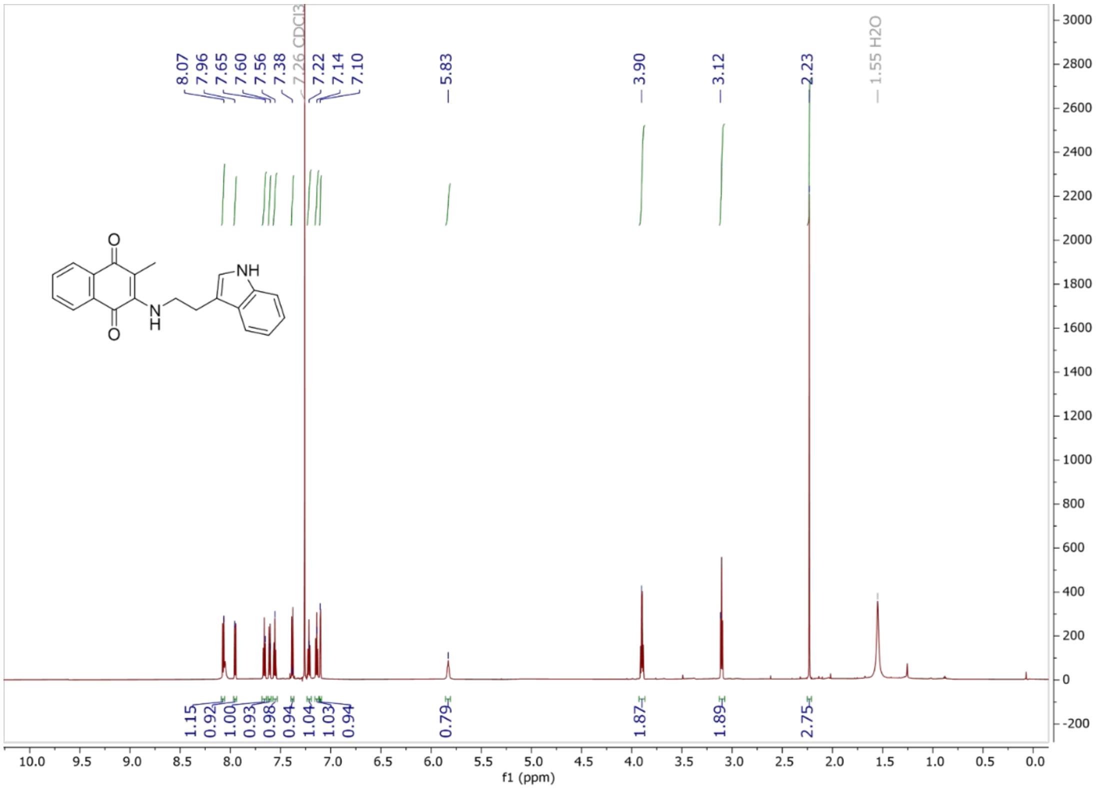

Figure S33: ^{1}H NMR Spectra (500 MHz) for 3-Tryptamine-menadione (**1n**) in $CDCl_3$

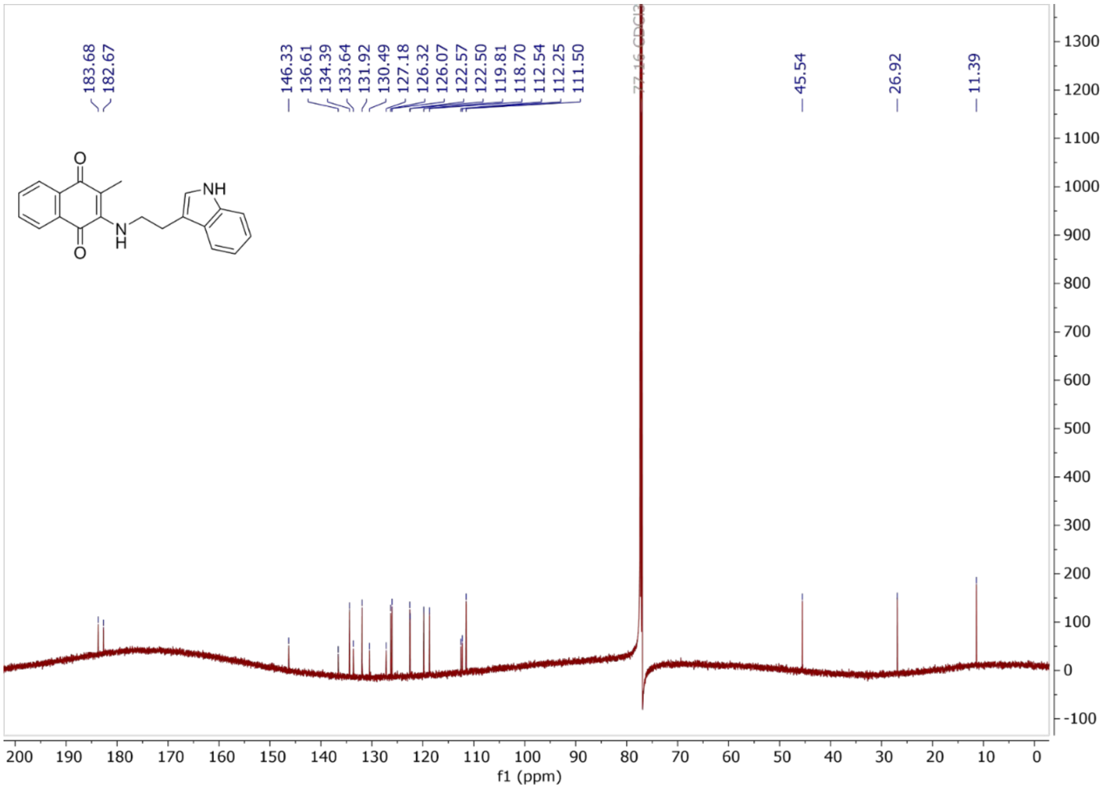

Figure S34: ^{13}C NMR Spectra (125 MHz) for 3-Tryptamine-menadione (**1n**) in $CDCl_3$

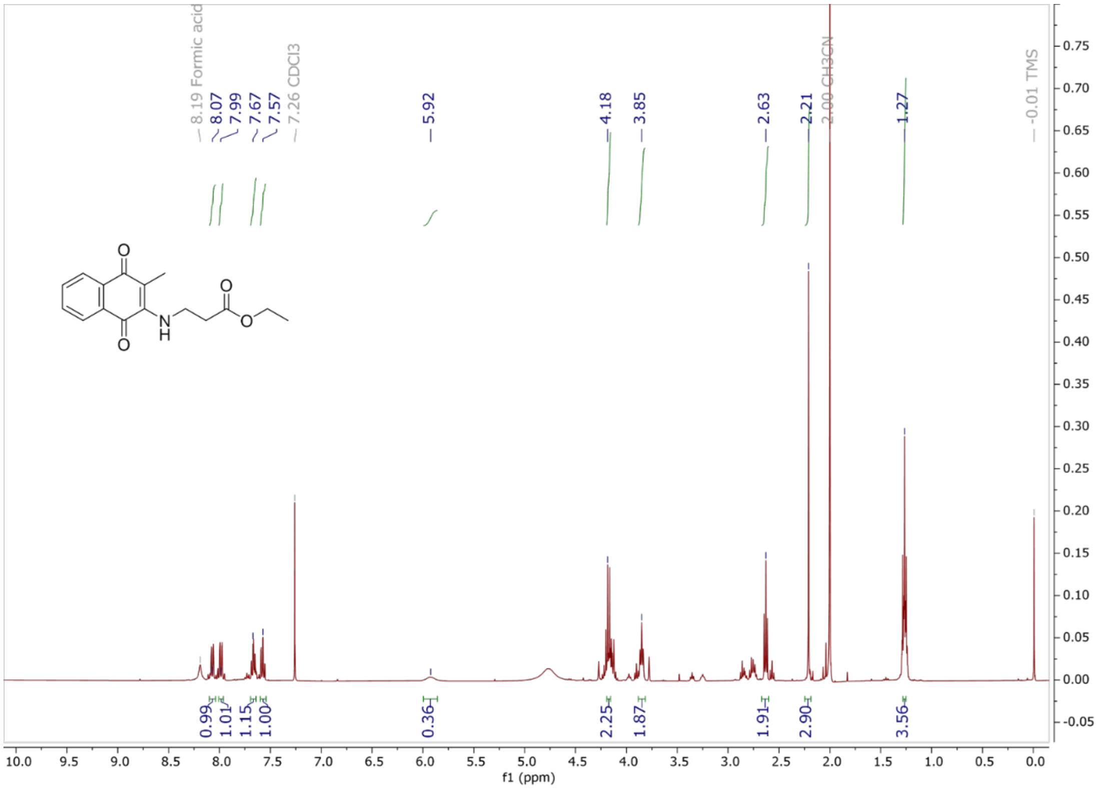

Figure S35: ^{1}H NMR Spectra (500 MHz) for 3-β-alanine-ethyl ester-menadione (**1o**) in $CDCl_3$

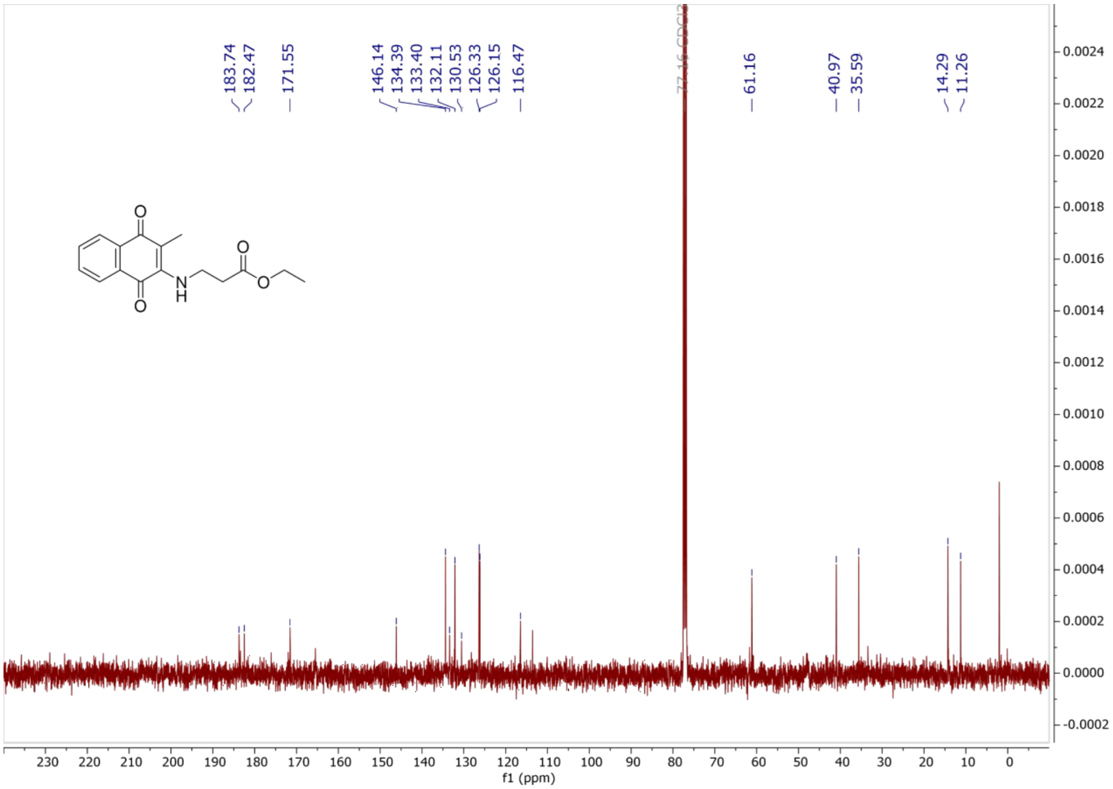

Figure S36: ^{13}C NMR Spectra (125 MHz) for 3-β-alanine-ethyl ester-menadione (**1o**) in $CDCl_3$

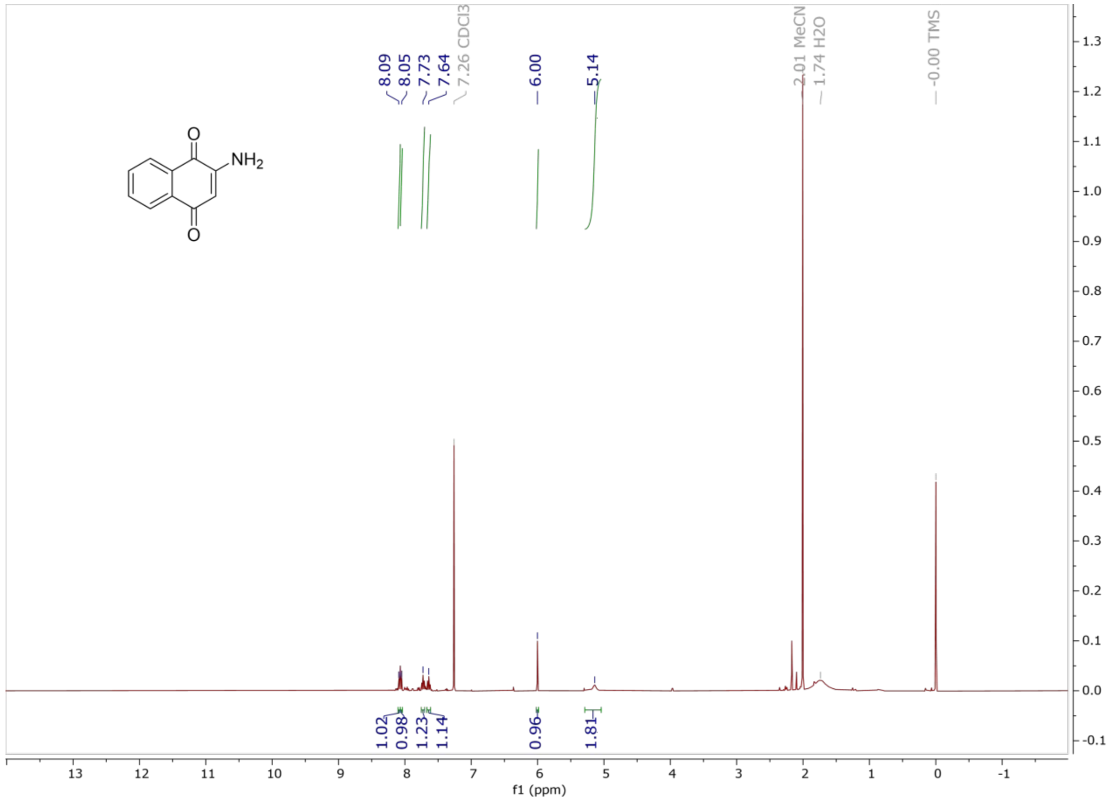

Figure S37: ^{1}H NMR Spectra (500 MHz) for 2-amine-naphthoquinone (**2a**) in $CDCl_3$

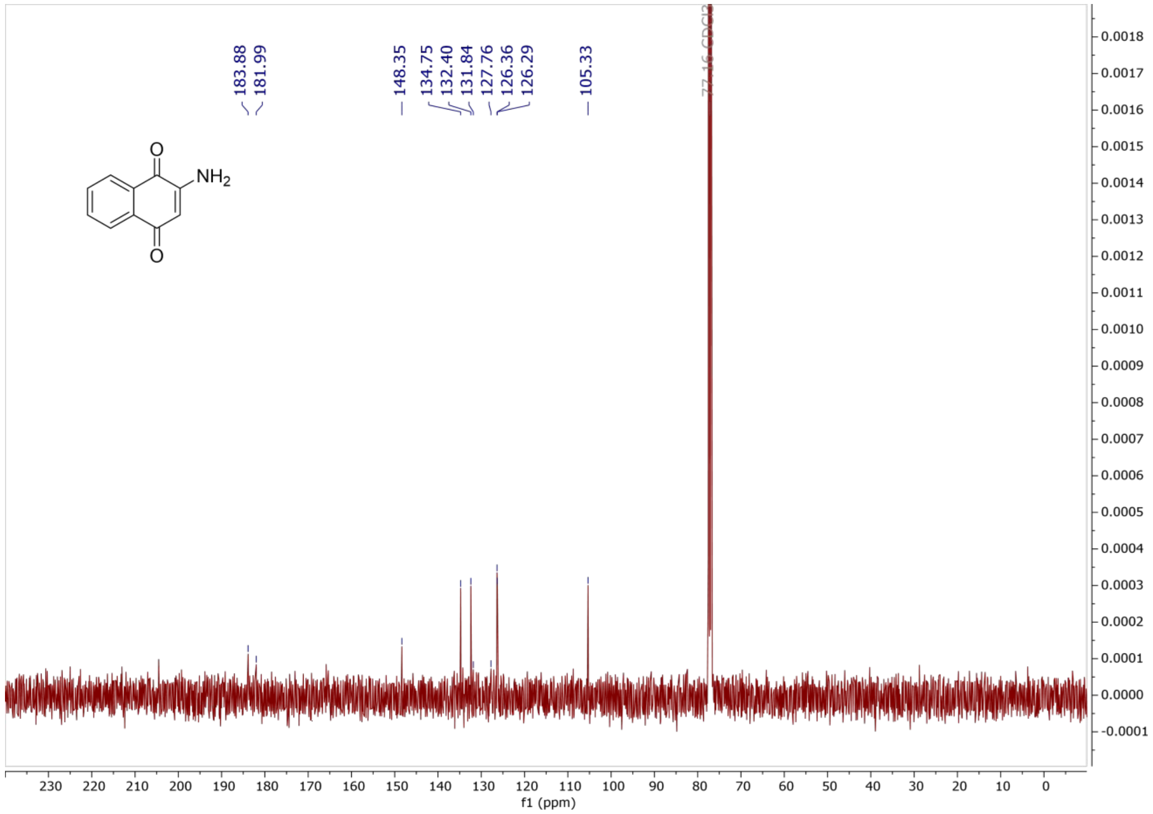

Figure S38: ^{13}C NMR Spectra (125 MHz) for 2-amine-naphthoquinone (**2a**) in $CDCl_3$

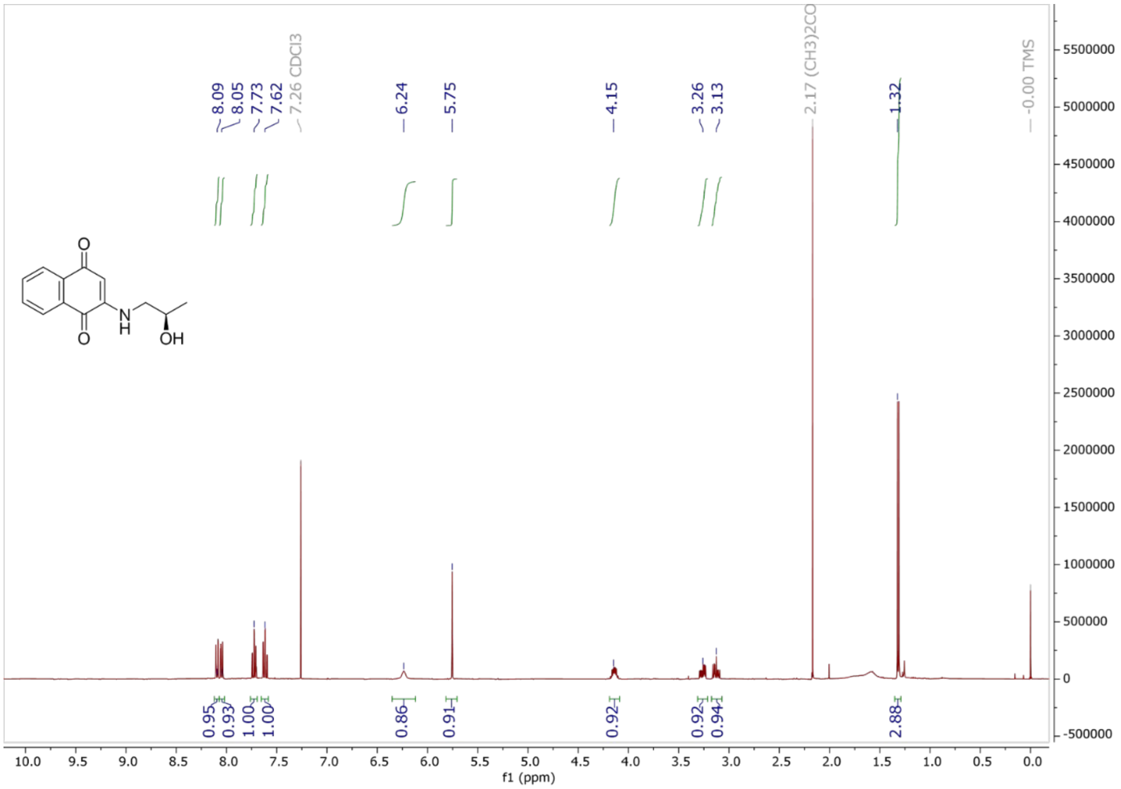

Figure S39: ^{1}H NMR Spectra (500 MHz) for 2-[(*R*)-amino-2-propanol]-naphthoquinone (**2c**) in $CDCl_3$

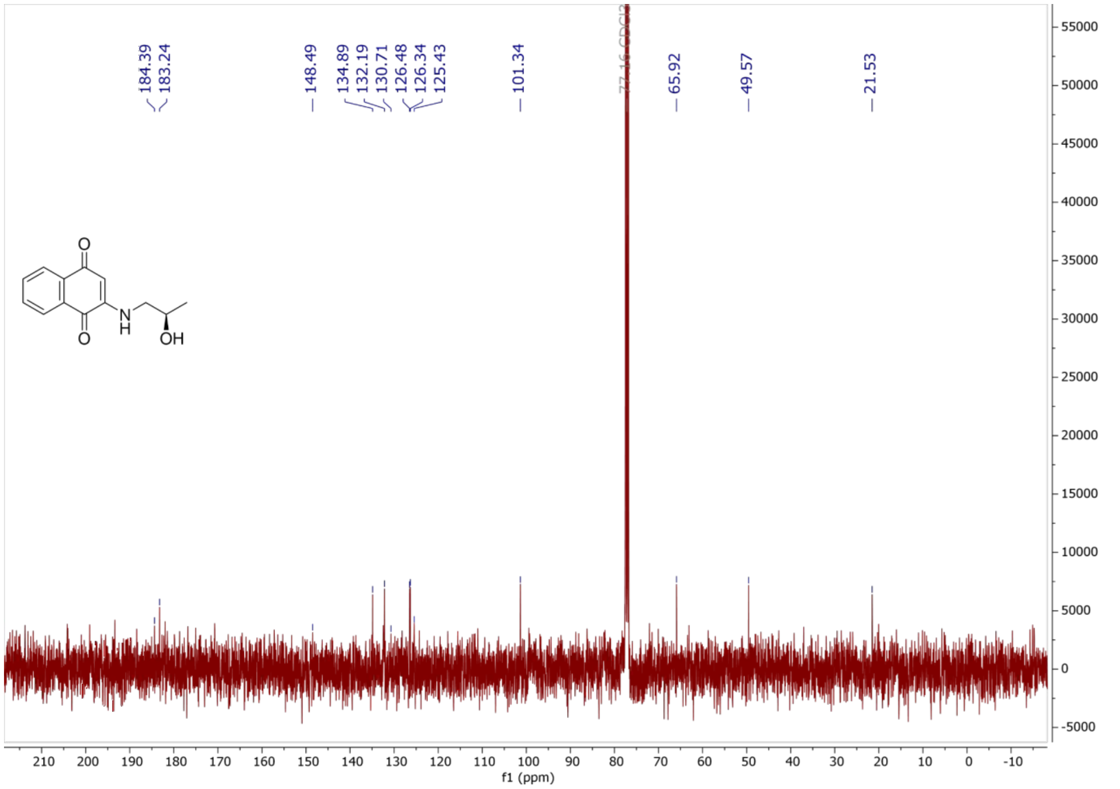

Figure S40: ^{13}C NMR Spectra (125 MHz) for 2-[(*R*)-amino-2-propanol]-naphthoquinone (**2c**) in $CDCl_3$

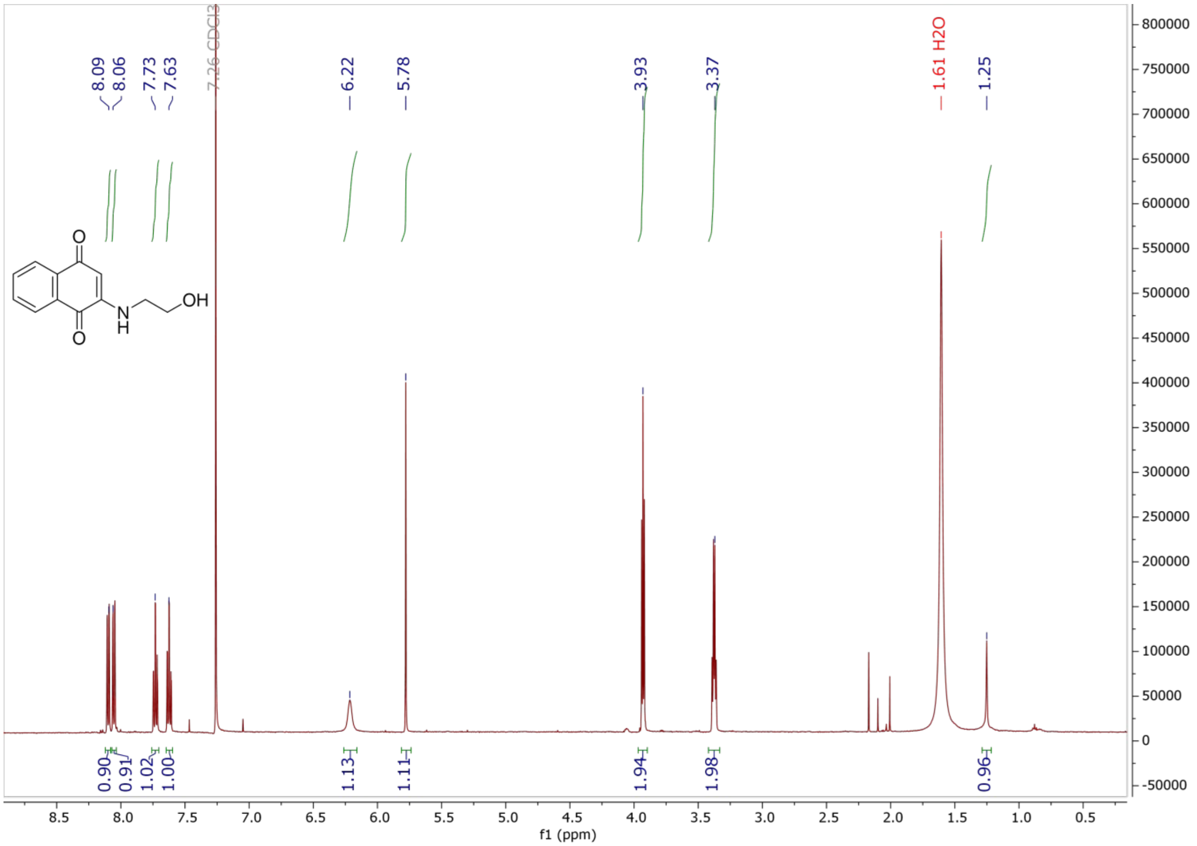

Figure S41: ^{1}H NMR Spectra (500 MHz) for 2-Aminoethanol-naphthoquinone (**2d**) in $CDCl_3$

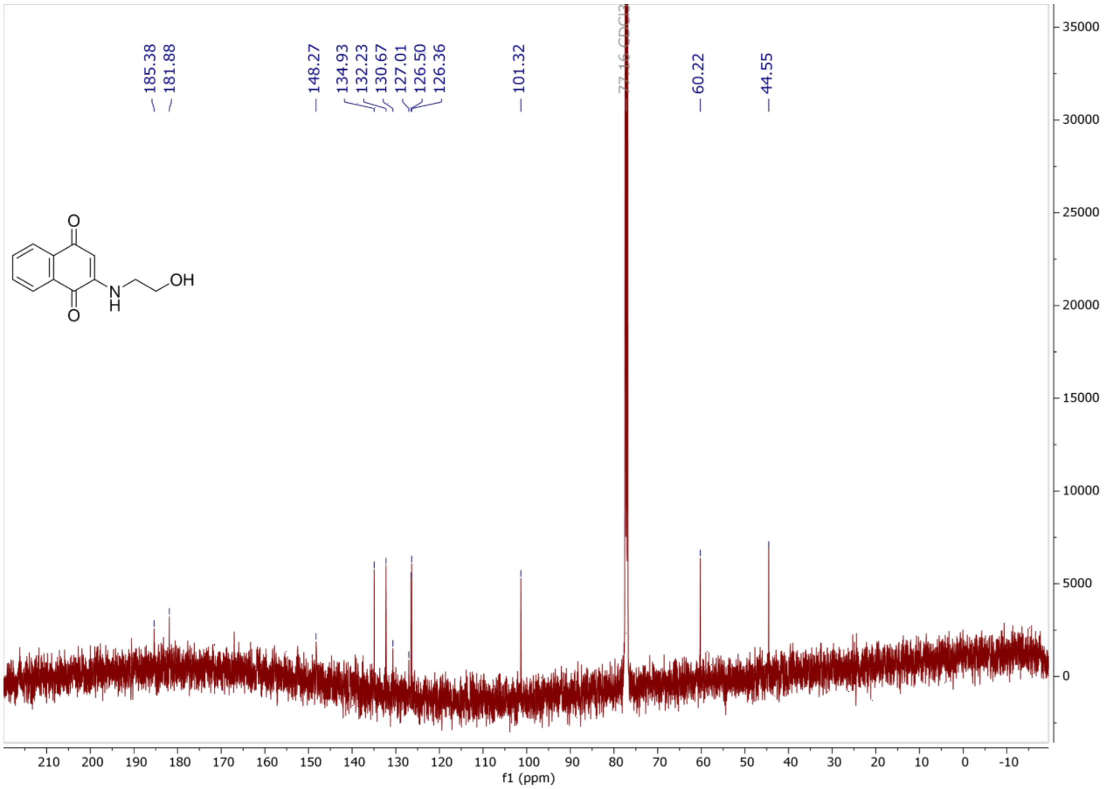

Figure S42: ^{13}C NMR Spectra (125 MHz) for 2-Aminoethanol-naphthoquinone (**2d**) in $CDCl_3$

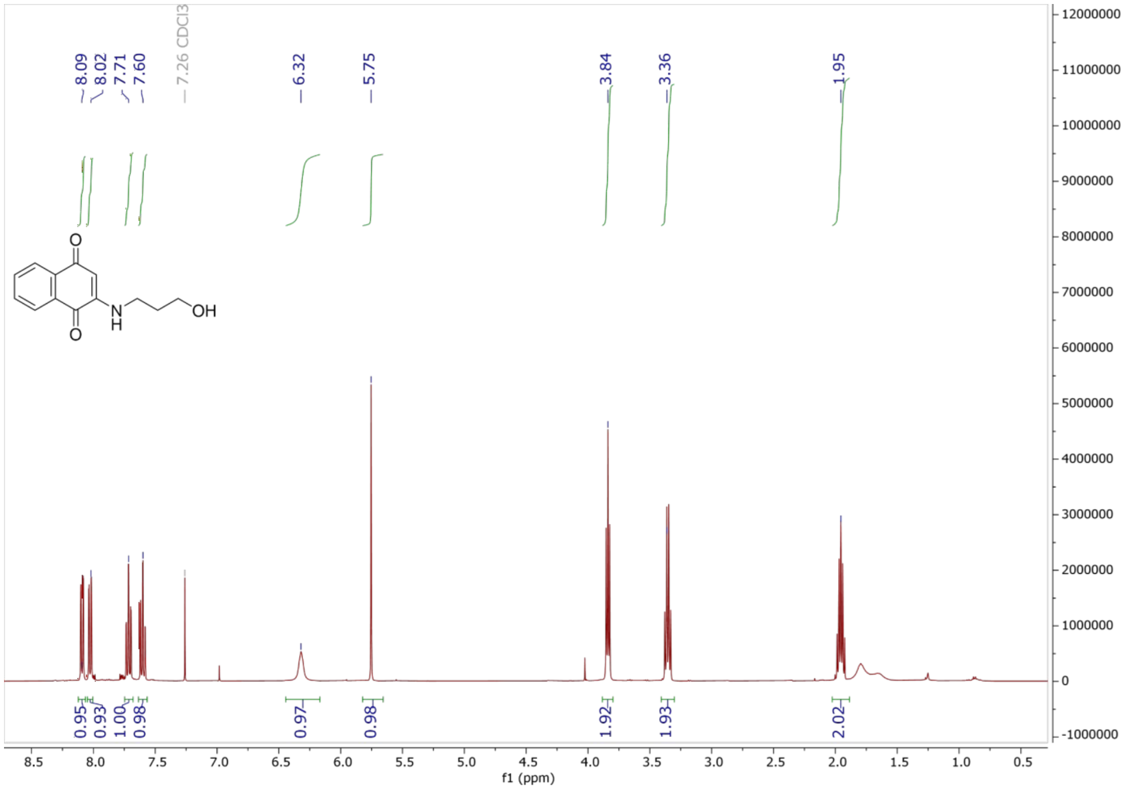

Figure S43: ^{1}H NMR Spectra (500 MHz) for 2-Aminopropanol-naphthoquinone (**2e**) in $CDCl_3$

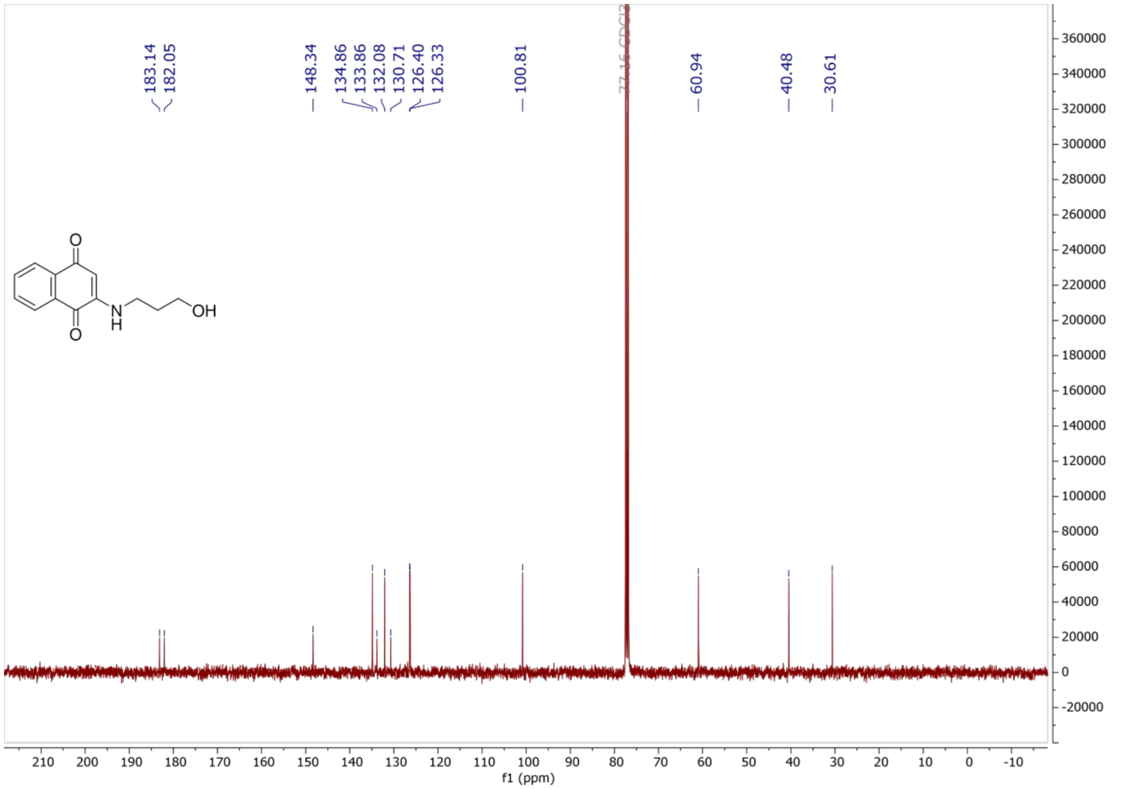

Figure S44: ^{13}C NMR Spectra (125 MHz) for 2-Aminopropanol-naphthoquinone (**2e**) in $CDCl_3$

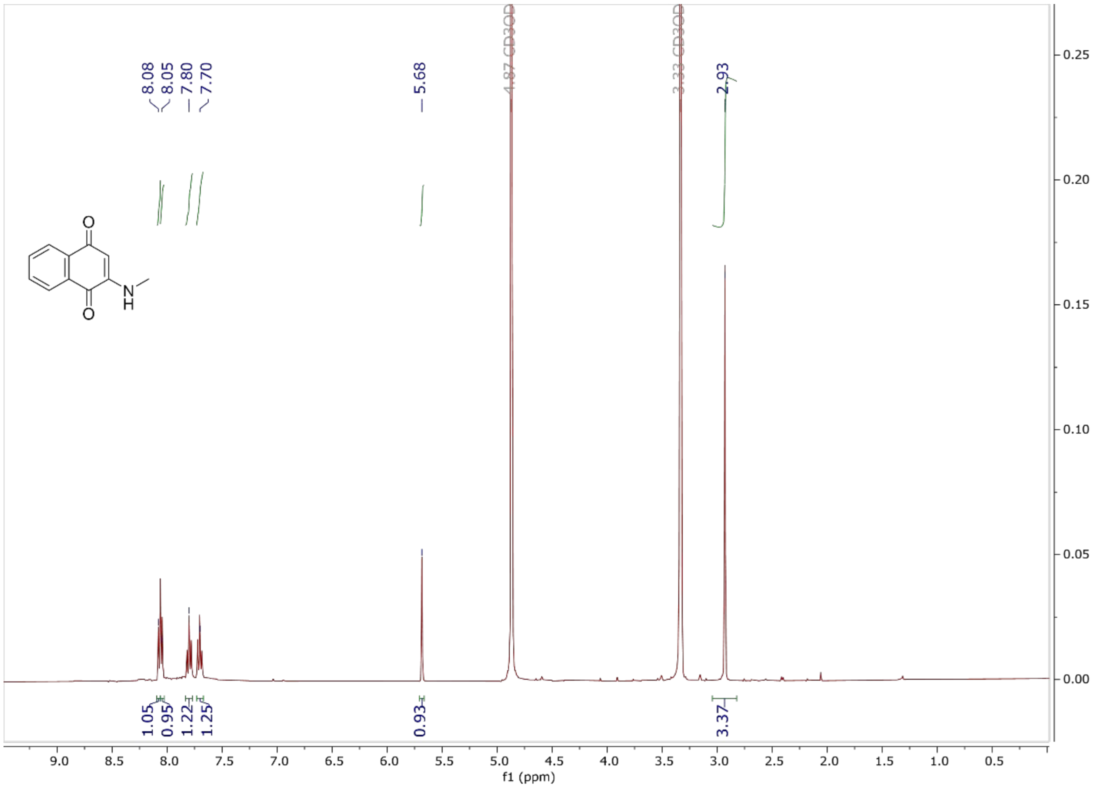

Figure S45: ^{1}H NMR Spectra (500 MHz) for 2-Methylamine-naphthoquinone (**2f**) in CD_3OD

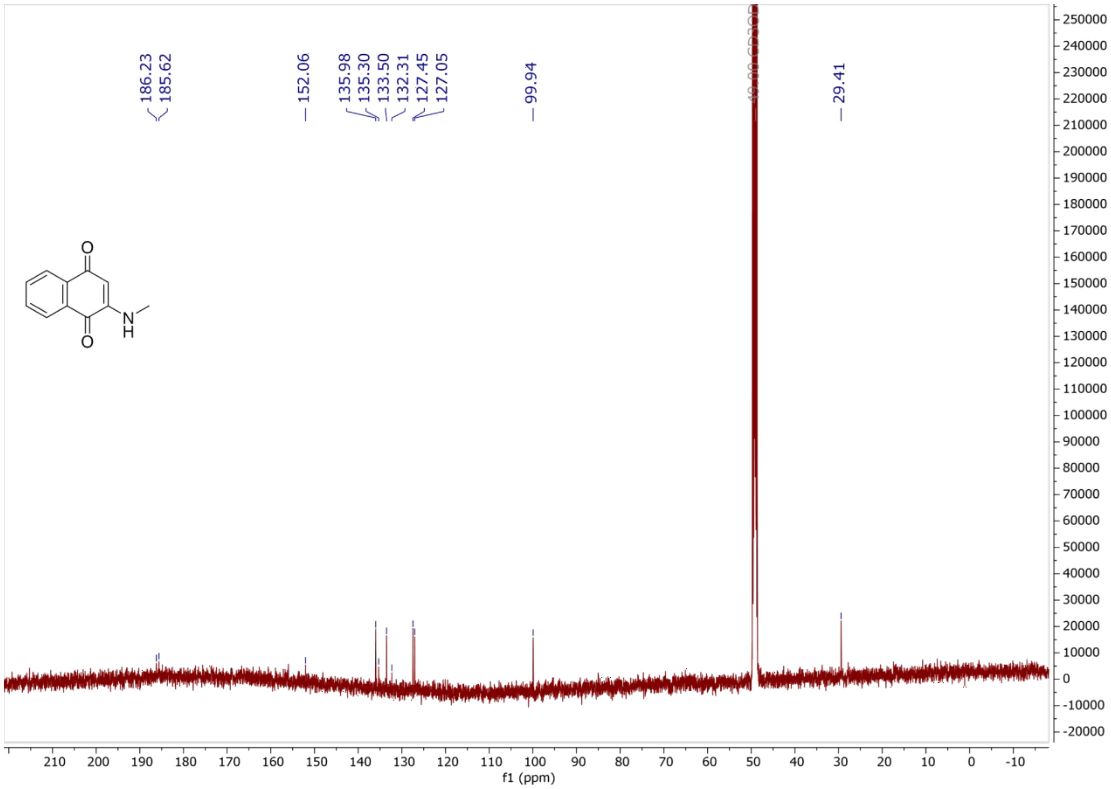

Figure S46: ^{13}C NMR Spectra (125 MHz) for 2-Methylamine-naphthoquinone (**2f**) in CD_3OD

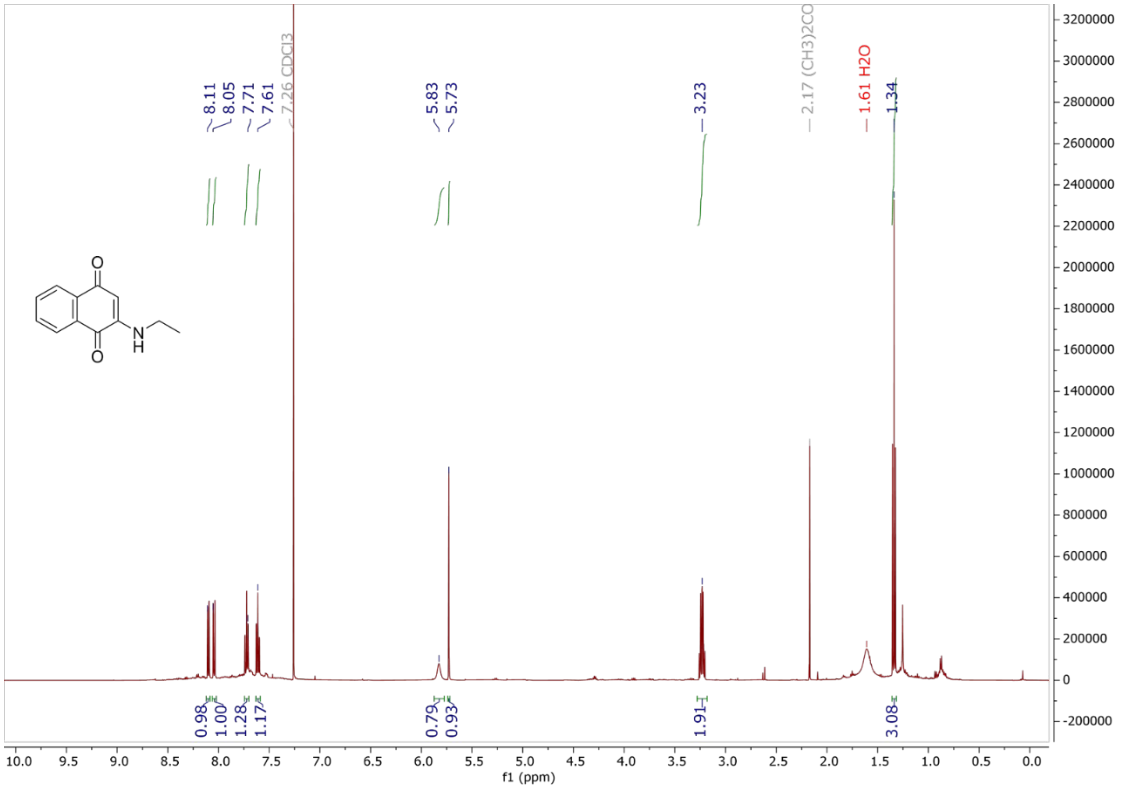

Figure S47: ^{1}H NMR Spectra (500 MHz) for 2-Ethylamine-naphthoquinone (**2g**) in $CDCl_3$

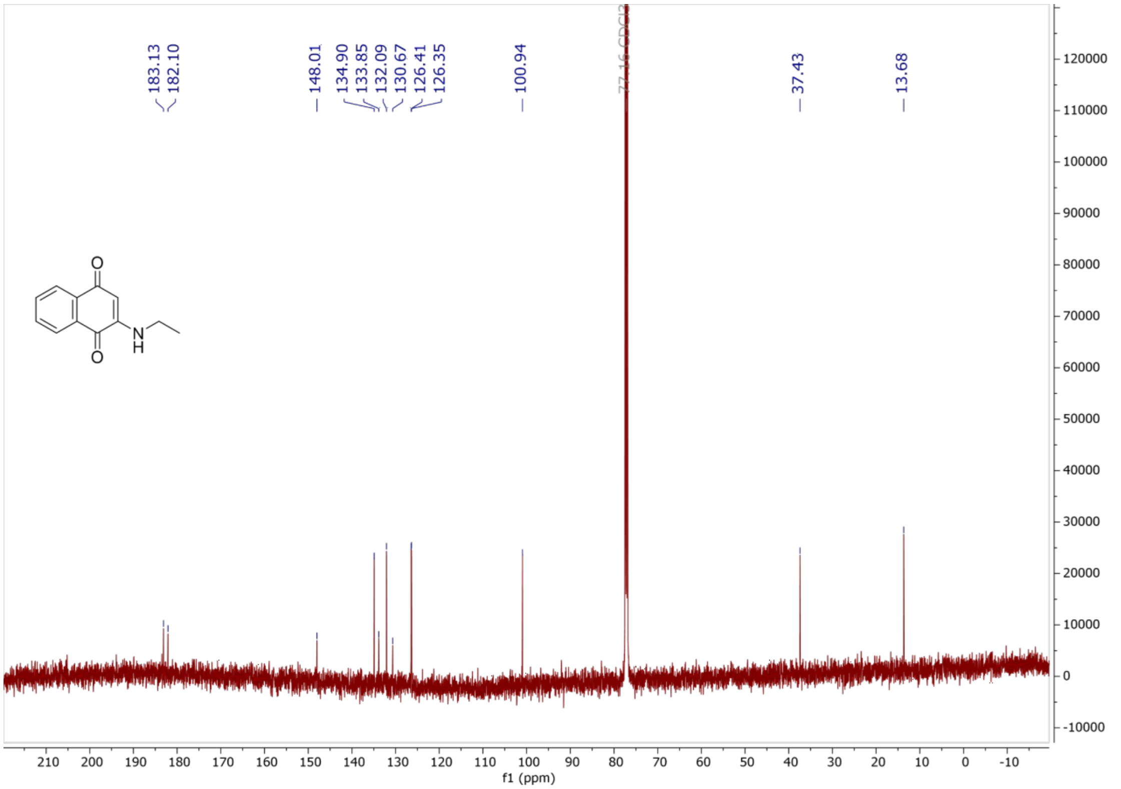

Figure S48: ^{13}C NMR Spectra (125 MHz) for 2-Ethylamine-naphthoquinone (**2g**) in $CDCl_3$

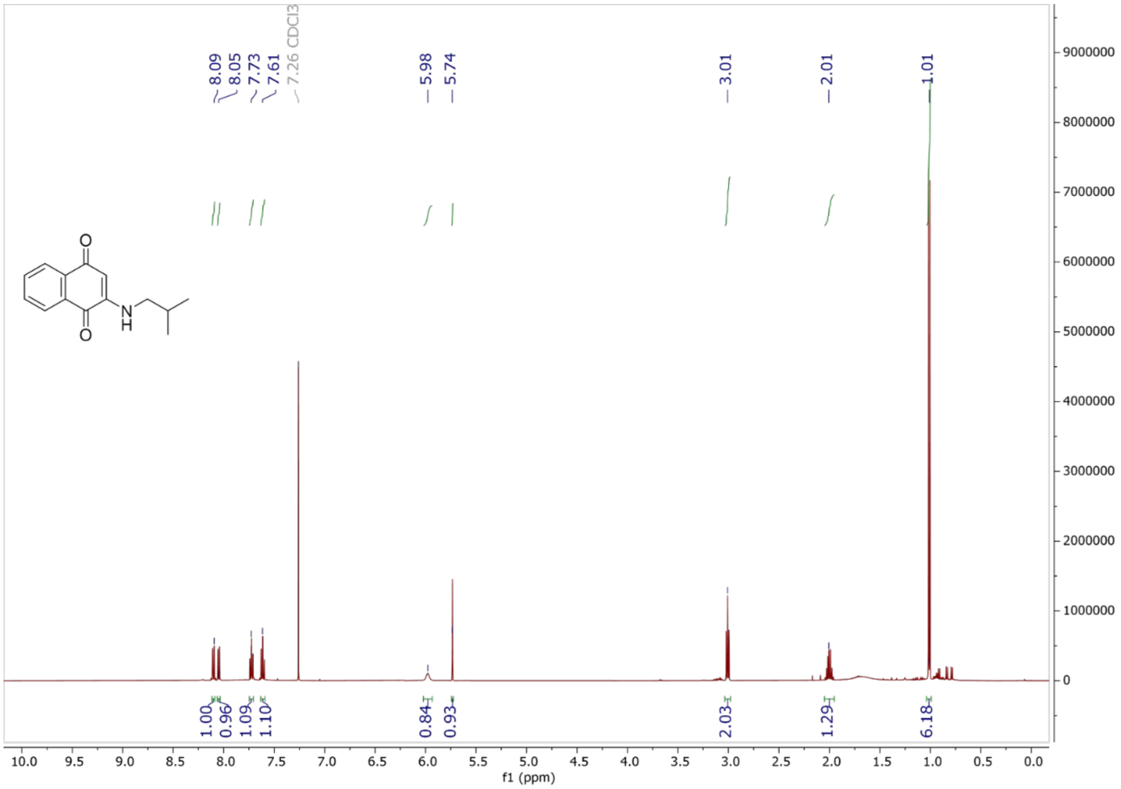

Figure S49: ^{1}H NMR Spectra (500 MHz) for 2-Isobutylamine-naphthoquinone (**2h**) in $CDCl_3$

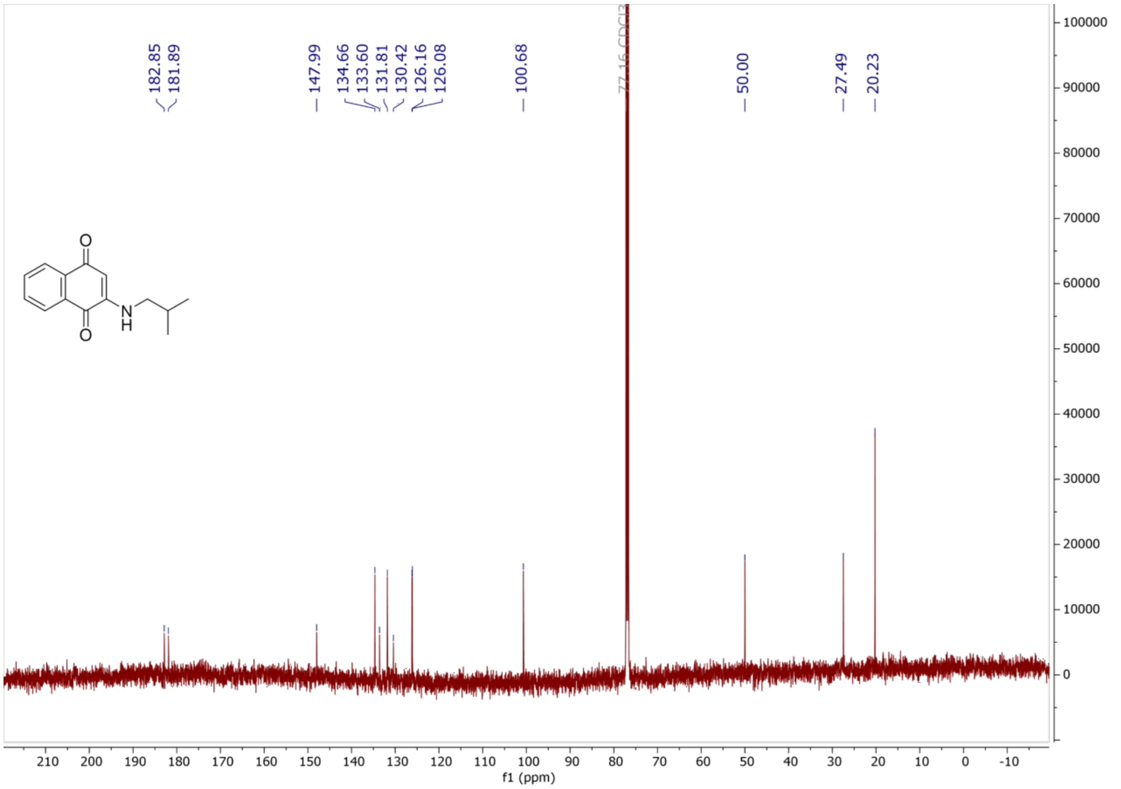

Figure S50: ^{13}C NMR Spectra (125 MHz) for 2-Isobutylamine-naphthoquinone (**2h**) in $CDCl_3$

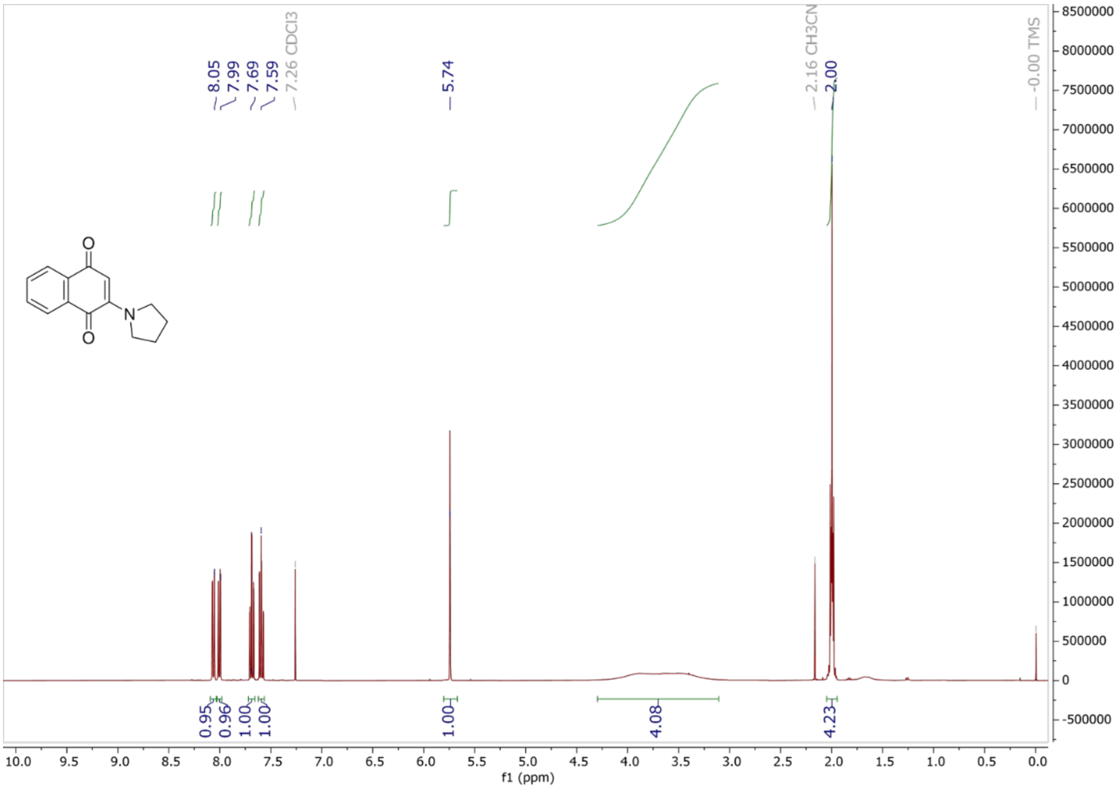

Figure S51: ^{1}H NMR Spectra (500 MHz) for 2-Pyrrolidine-naphthoquinone (**2i**) in $CDCl_3$

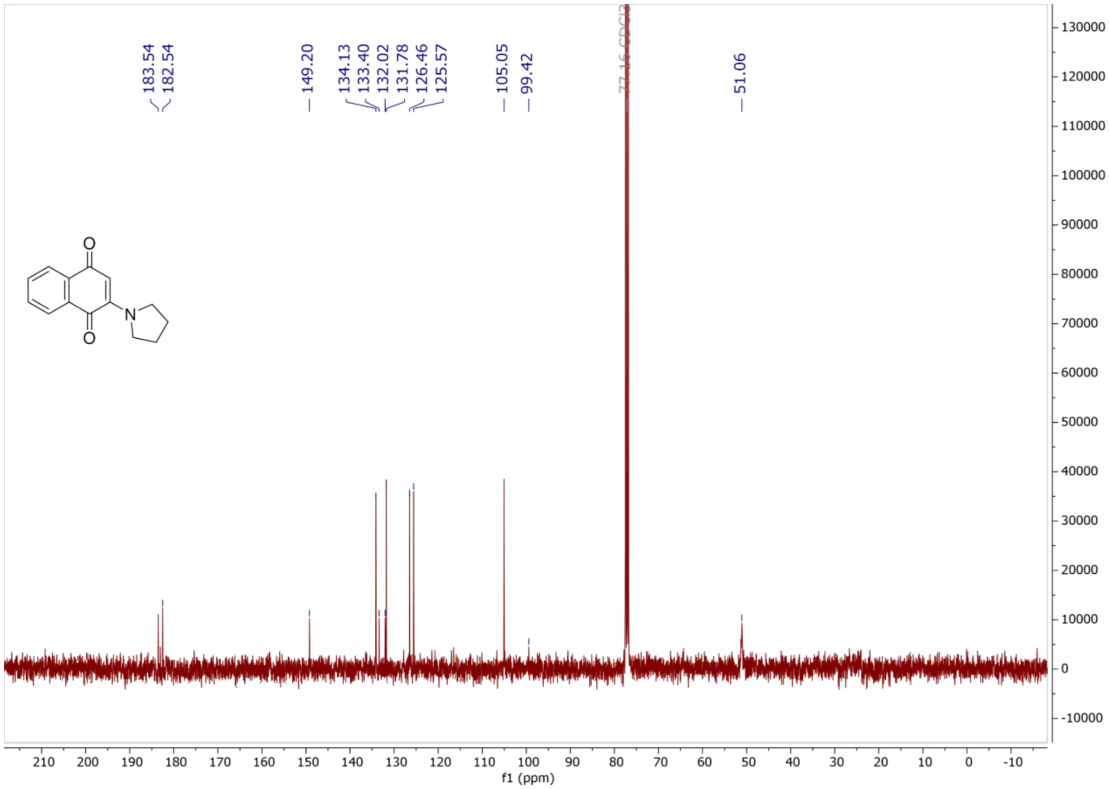

Figure S52: ^{13}C NMR Spectra (125 MHz) for 2-Pyrrolidine-naphthoquinone (**2i**) in $CDCl_3$

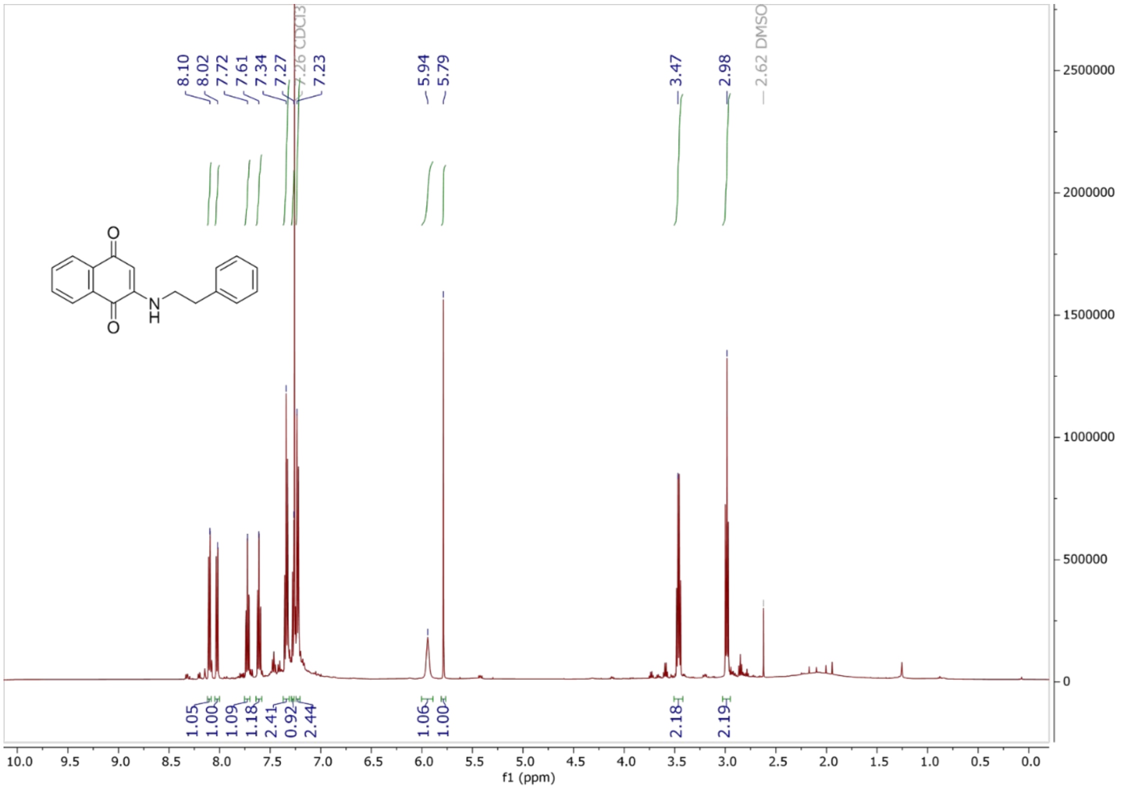

Figure S53: ^{1}H NMR Spectra (500 MHz) for 2-Phenethylamine-naphthoquinone (**2j**) in $CDCl_3$

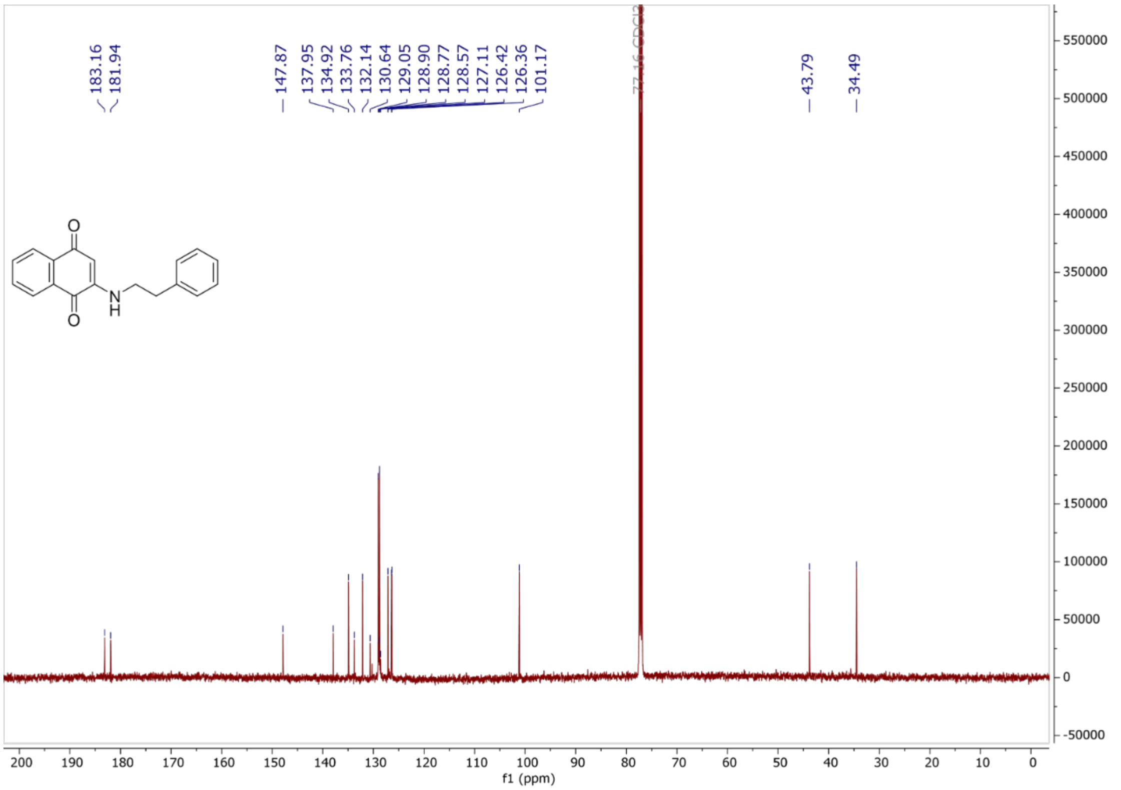

Figure S54: ^{13}C NMR Spectra (125 MHz) for 2-Phenethylamine-naphthoquinone (**2j**) in $CDCl_3$

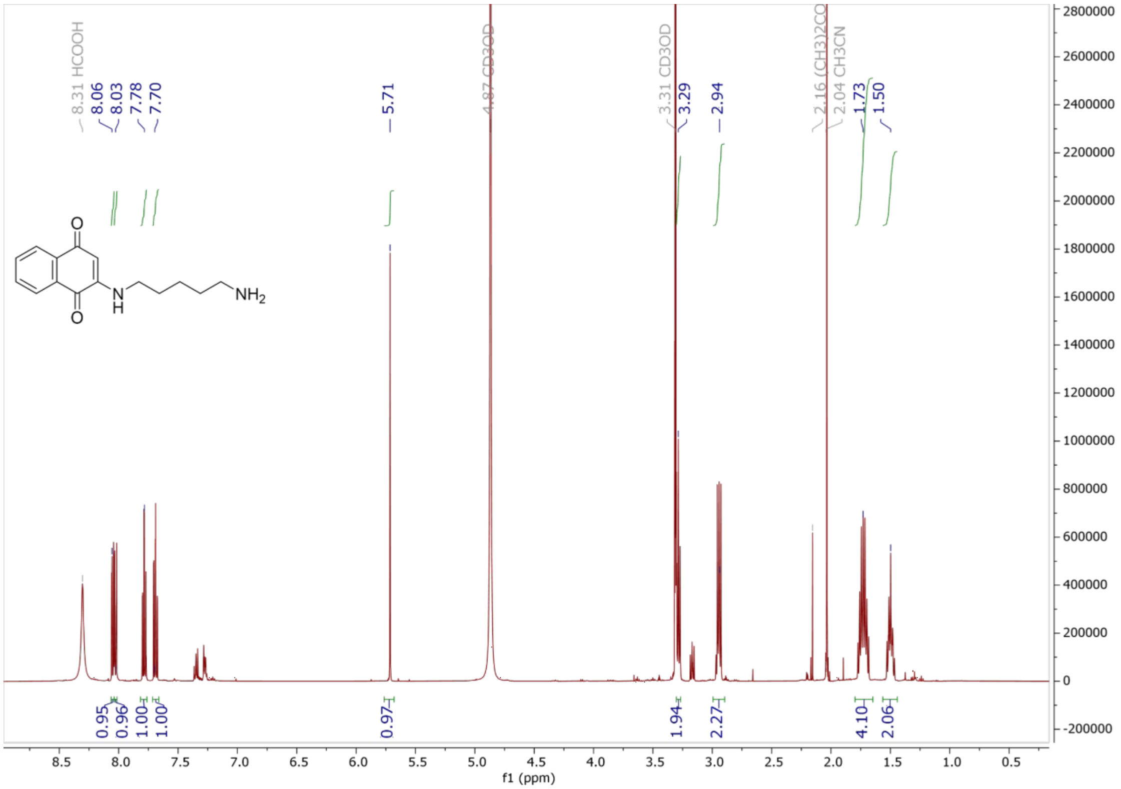

Figure S55: ^{1}H NMR Spectra (500 MHz) for 2-Cadaverine-naphthoquinone (**2k**) in CD_3OD

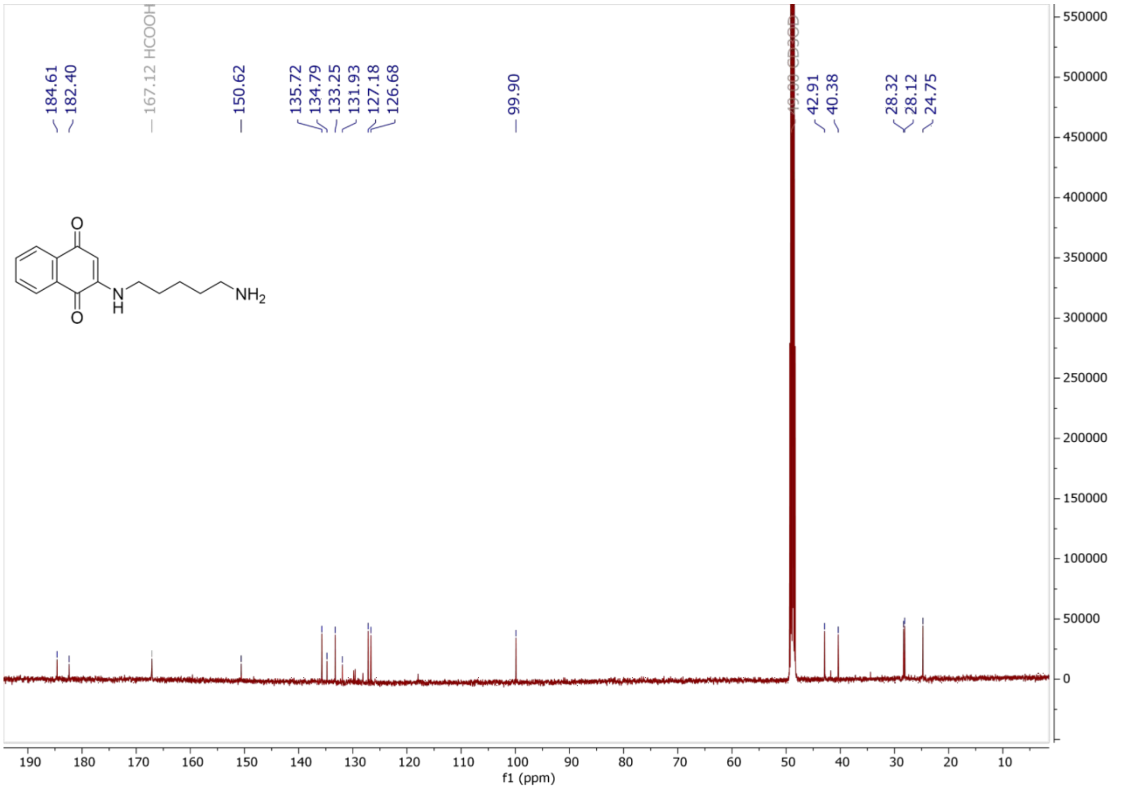

Figure S56: ^{13}C NMR Spectra (125 MHz) for 2-Cadaverine-naphthoquinone (**2k**) in CD_3OD

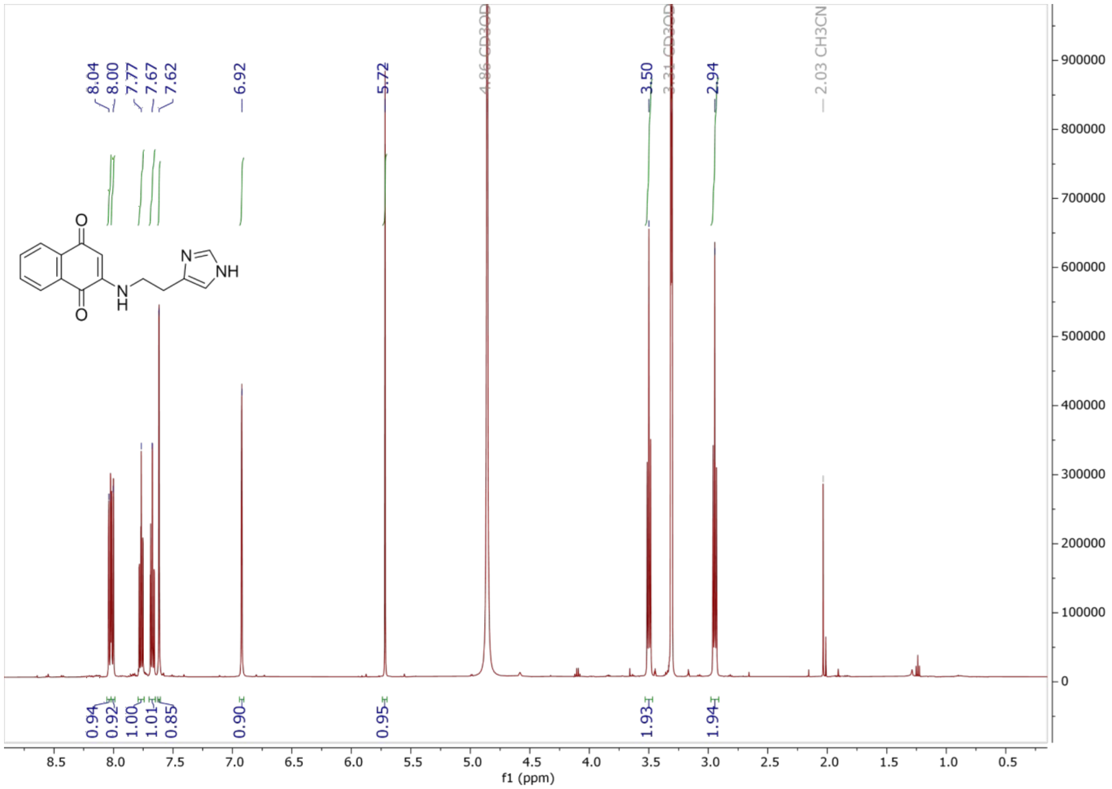

Figure S57: ^{1}H NMR Spectra (500 MHz) for 2-Histamine-naphthoquinone (**2l**) in CD_3OD

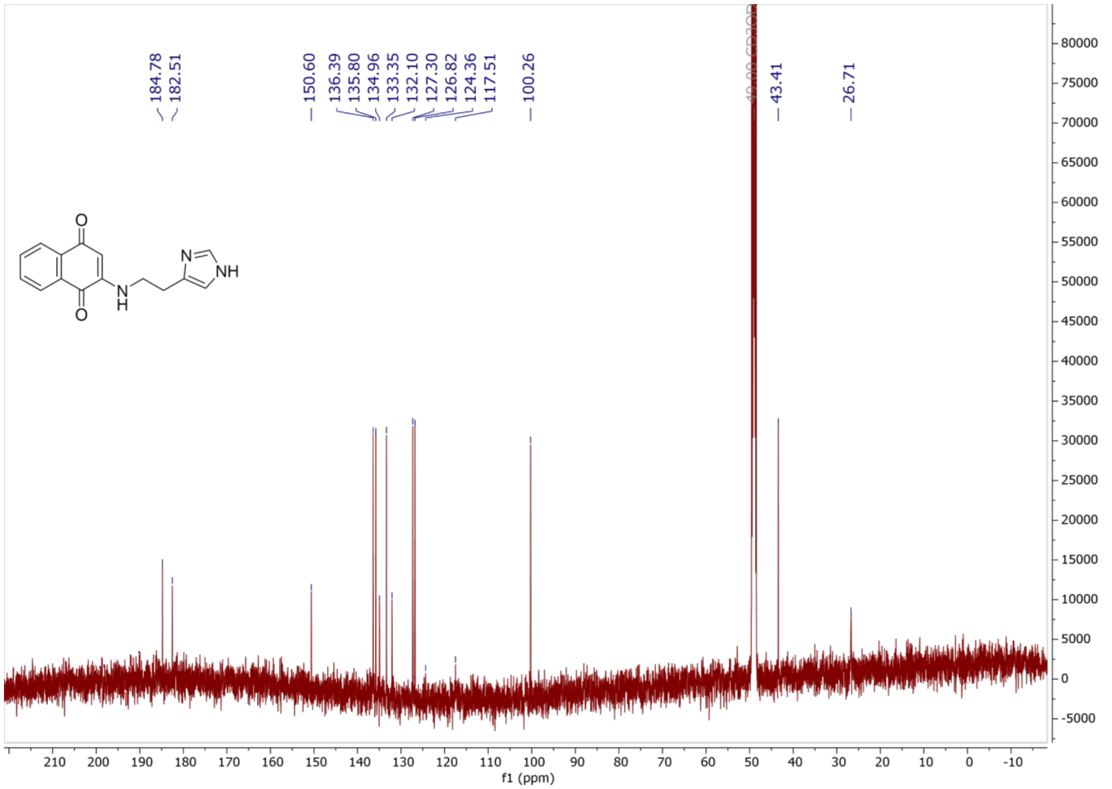

Figure S58: ^{13}C NMR Spectra (125 MHz) for 2-Histamine-naphthoquinone (**2l**) in CD_3OD

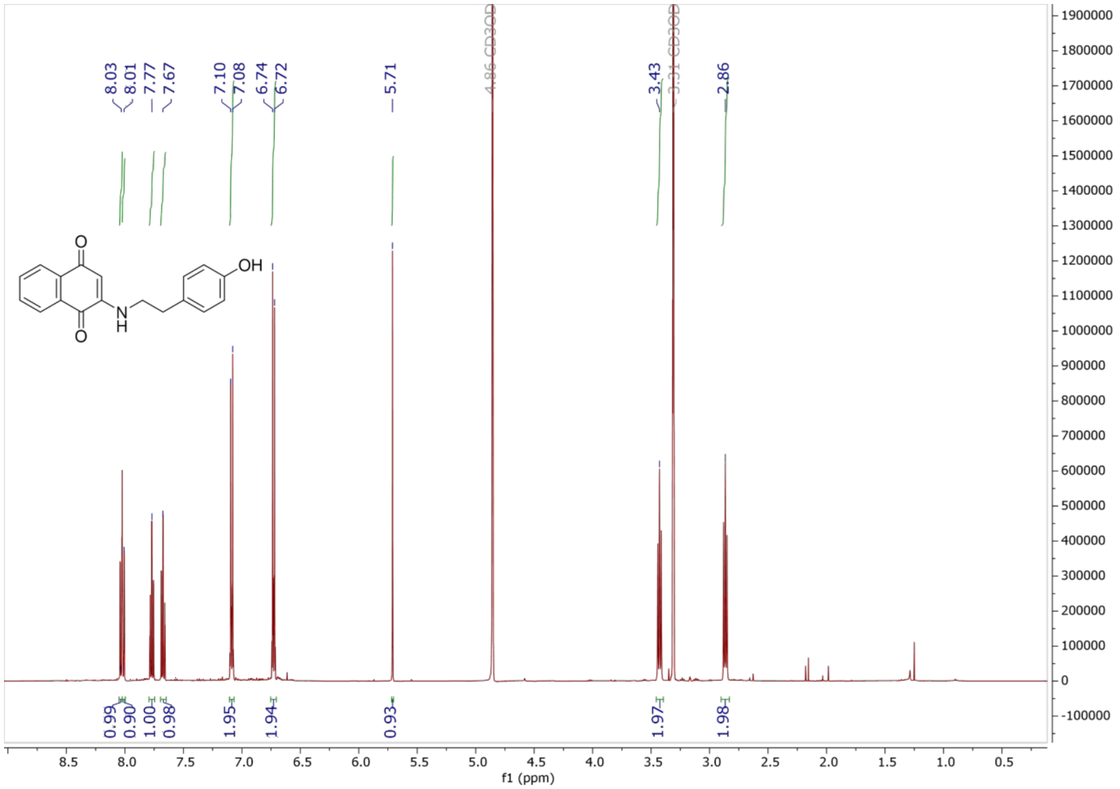

Figure S59: ^{1}H NMR Spectra (500 MHz) for 2-Tyramine-naphthoquinone (**2m**) in CD_3OD

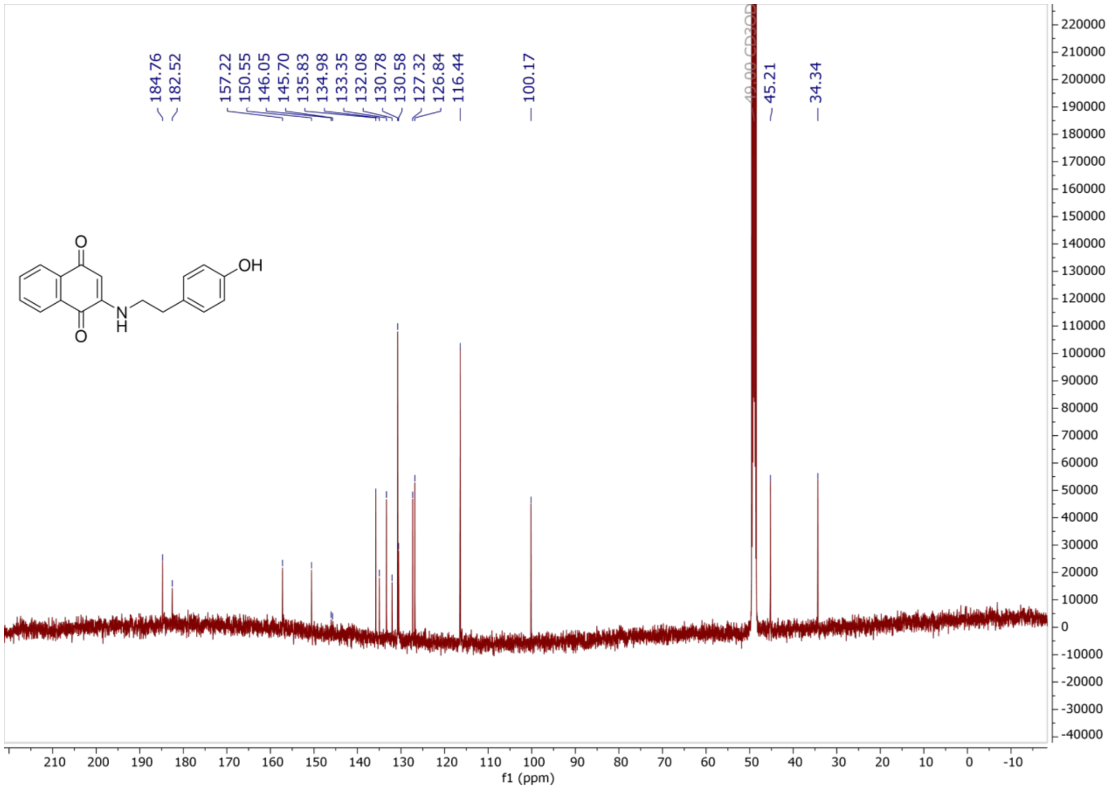

Figure S60: ^{13}C NMR Spectra (125 MHz) for 2-Tyramine-naphthoquinone (**2m**) in CD_3OD

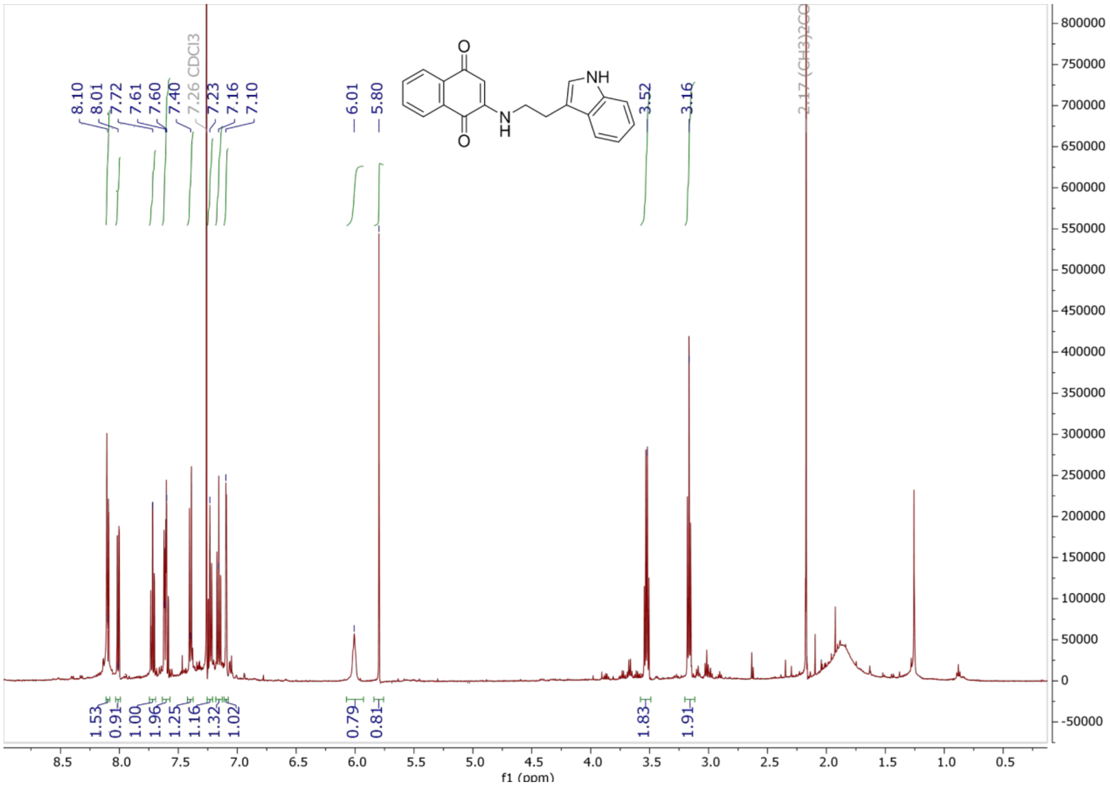

Figure S61: ^{1}H NMR Spectra (500 MHz) for 2-Tryptamine-naphthoquinone (**2n**) in $CDCl_3$

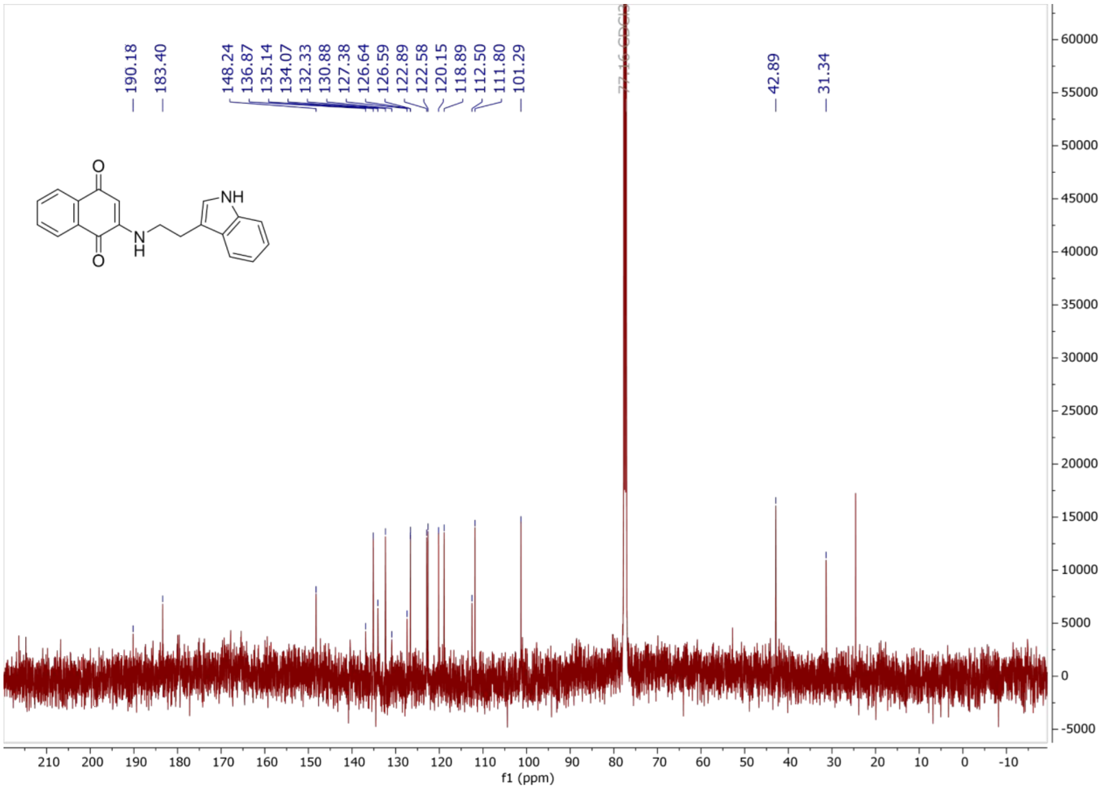

Figure S62: ^{13}C NMR Spectra (125 MHz) for 2-Tryptamine-naphthoquinone (**2n**) in $CDCl_3$

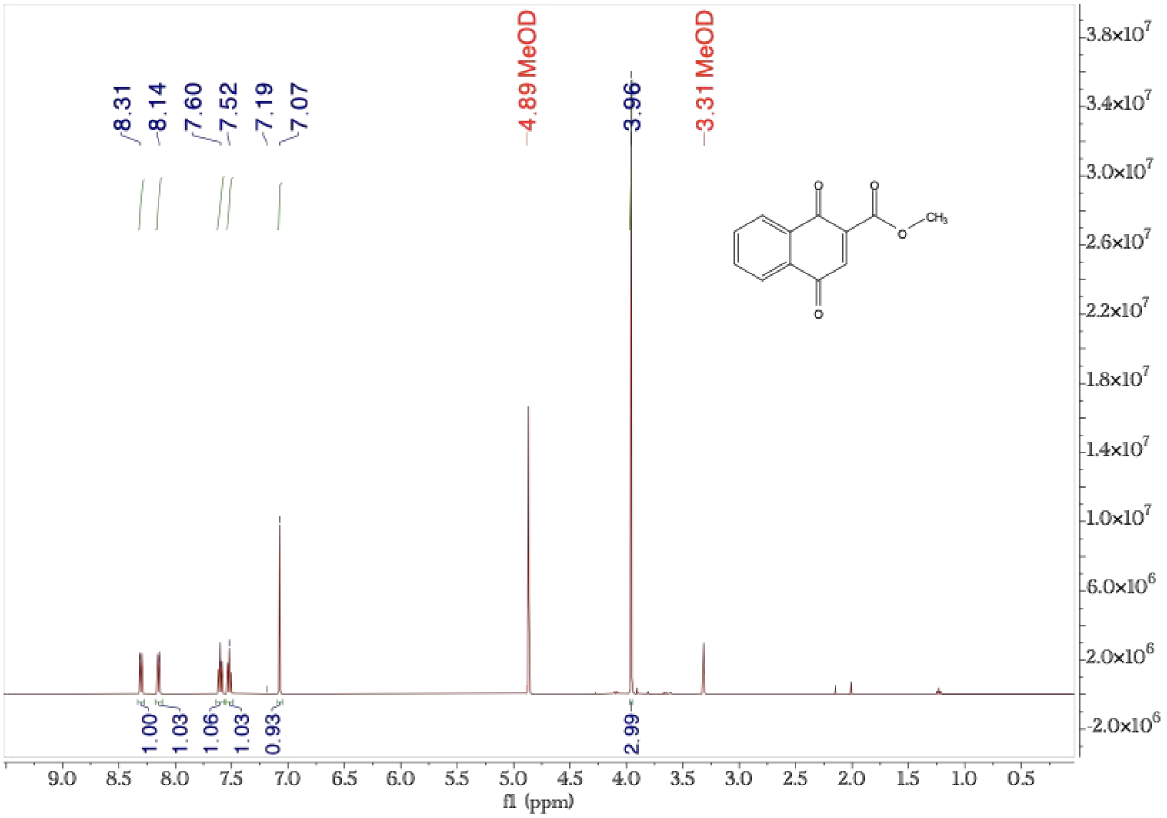

Figure S63: ^{1}H NMR Spectra (400 MHz) for Methyl DHNA (**4**) in CD_3OD

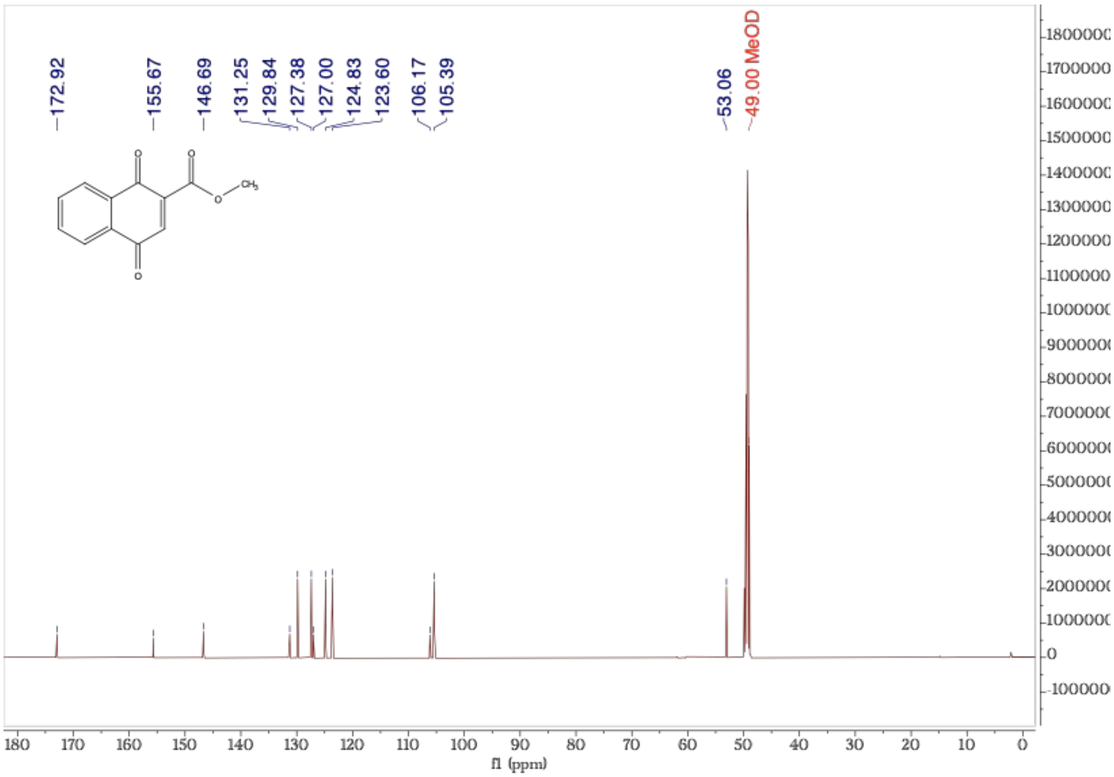

Figure S64: ^{13}C NMR Spectra (125 MHz) for Methyl DHNA (**4**) in CD_3OD

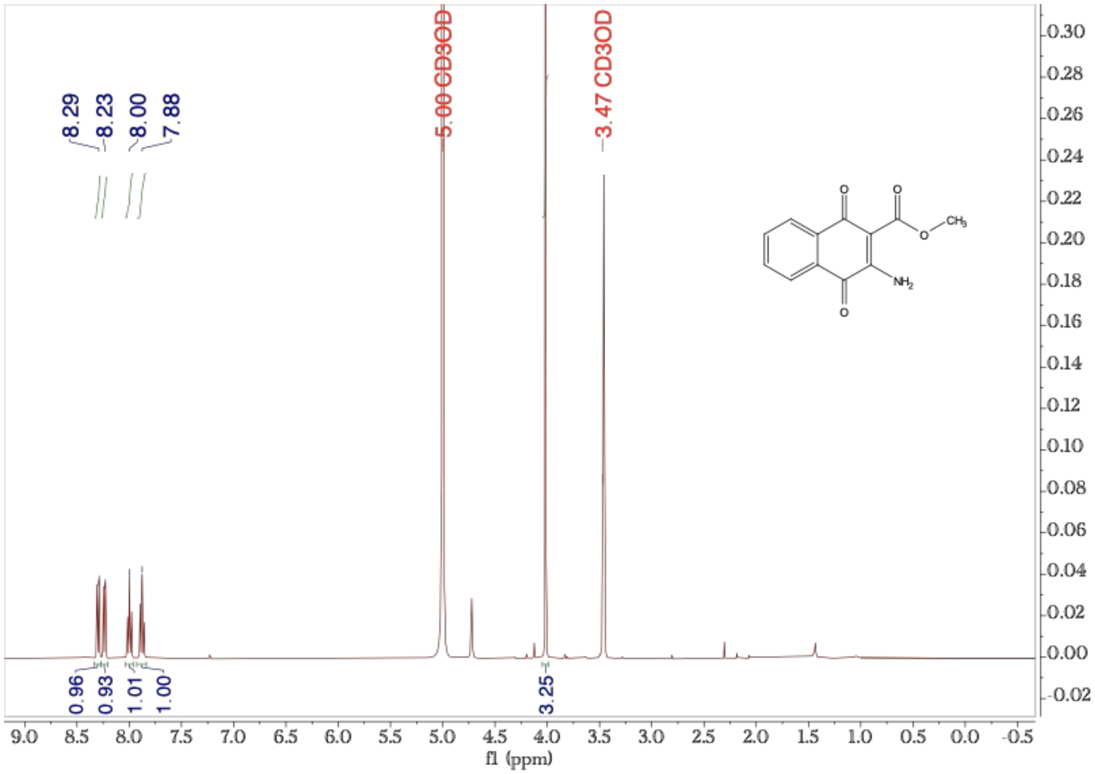

Figure S65: ^{1}H NMR Spectra (500 MHz) for 3-amine-DHNA methyl ester (**4a**) in CD_3OD

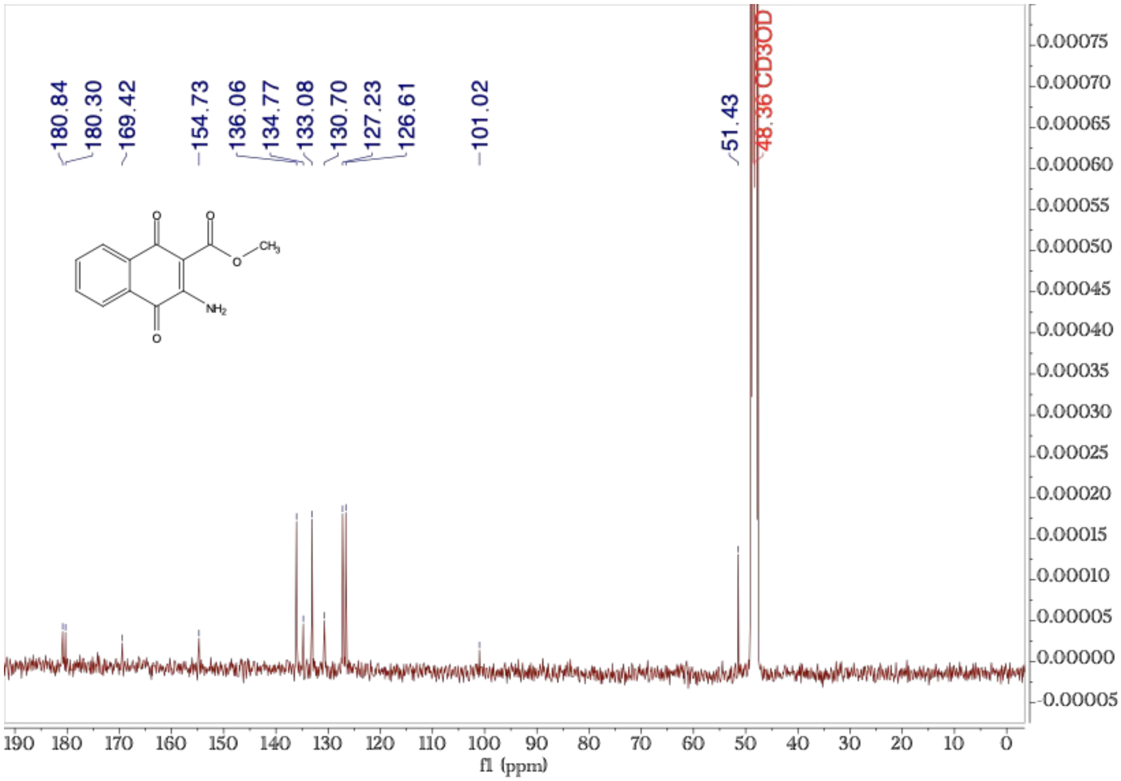

Figure S66: ^{13}C NMR Spectra (125 MHz) for 3-amine-DHNA methyl ester (**4a**) in CD_3OD

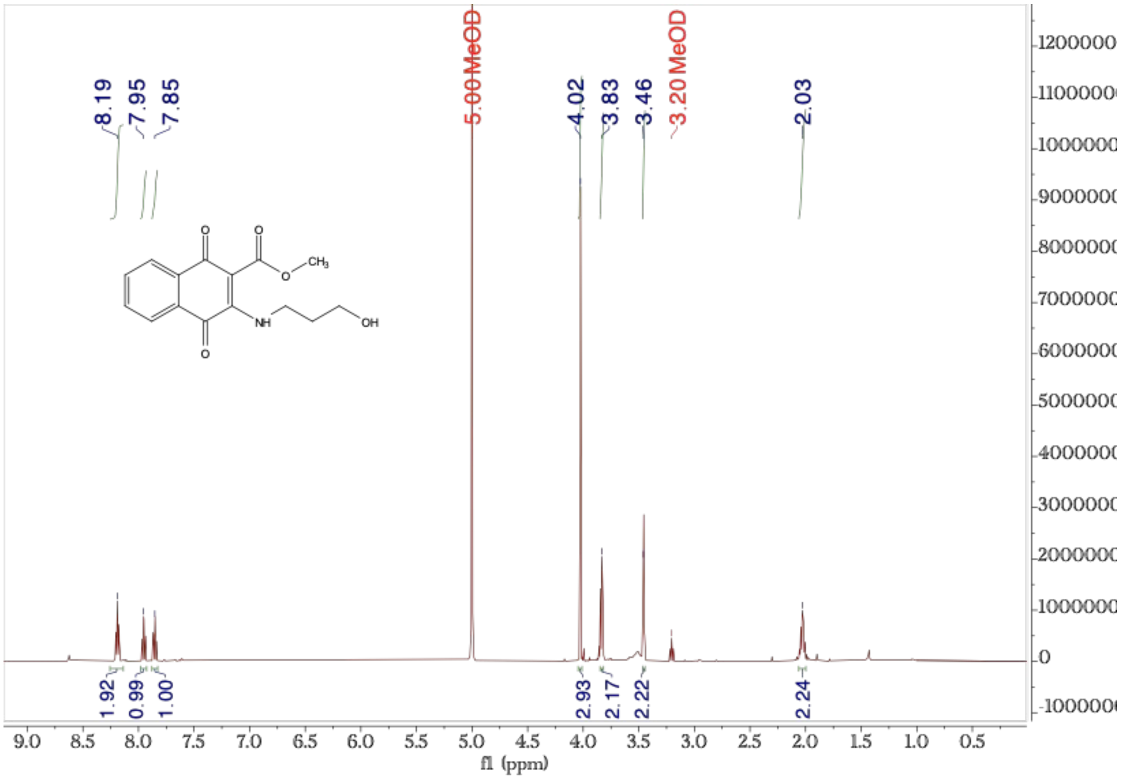

Figure S67: ^{1}H NMR Spectra (500 MHz) for 3-Aminopropanol-DHNA methyl ester (**4e**) in CD_3OD

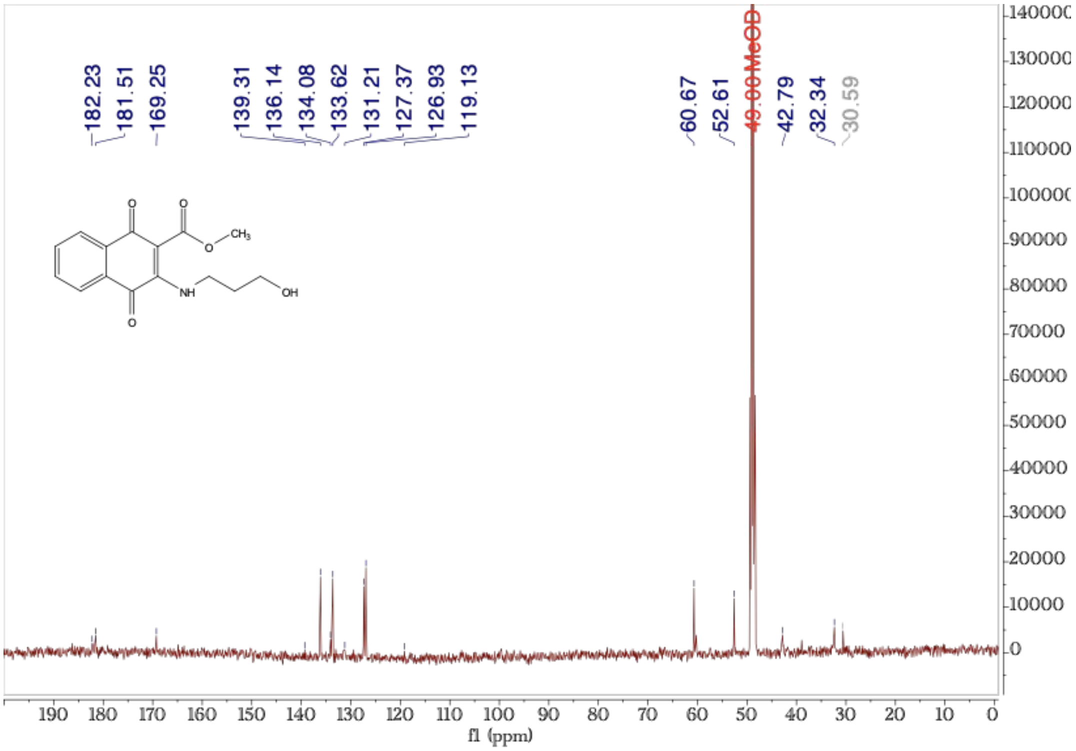

Figure S68: ^{13}C NMR Spectra (125 MHz) for 3-Aminopropanol-DHNA methyl ester (**4e**) in CD_3OD

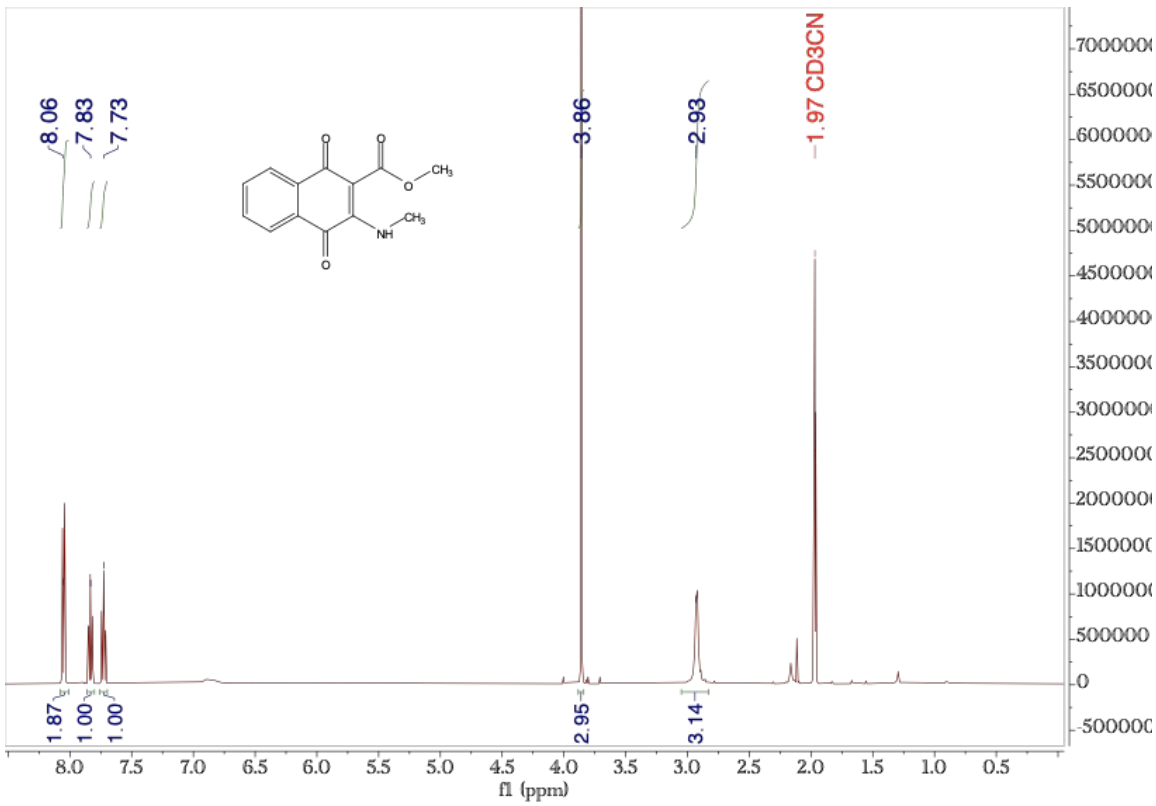

Figure S69: ^{1}H NMR Spectra (500 MHz) for 3-Methylamine-DHNA methyl ester (**4f**) in CD_3CN

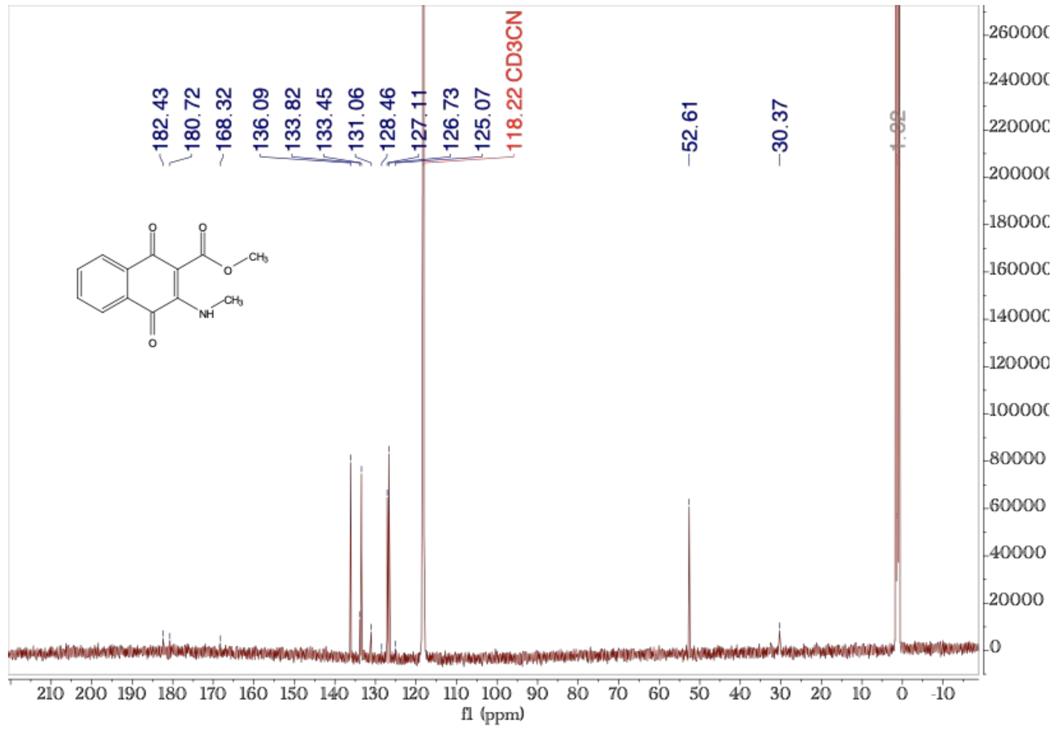

Figure S70: ^{13}C NMR Spectra (125 MHz) for 3-Methylamine-DHNA methyl ester (**4f**) in CD_3CN

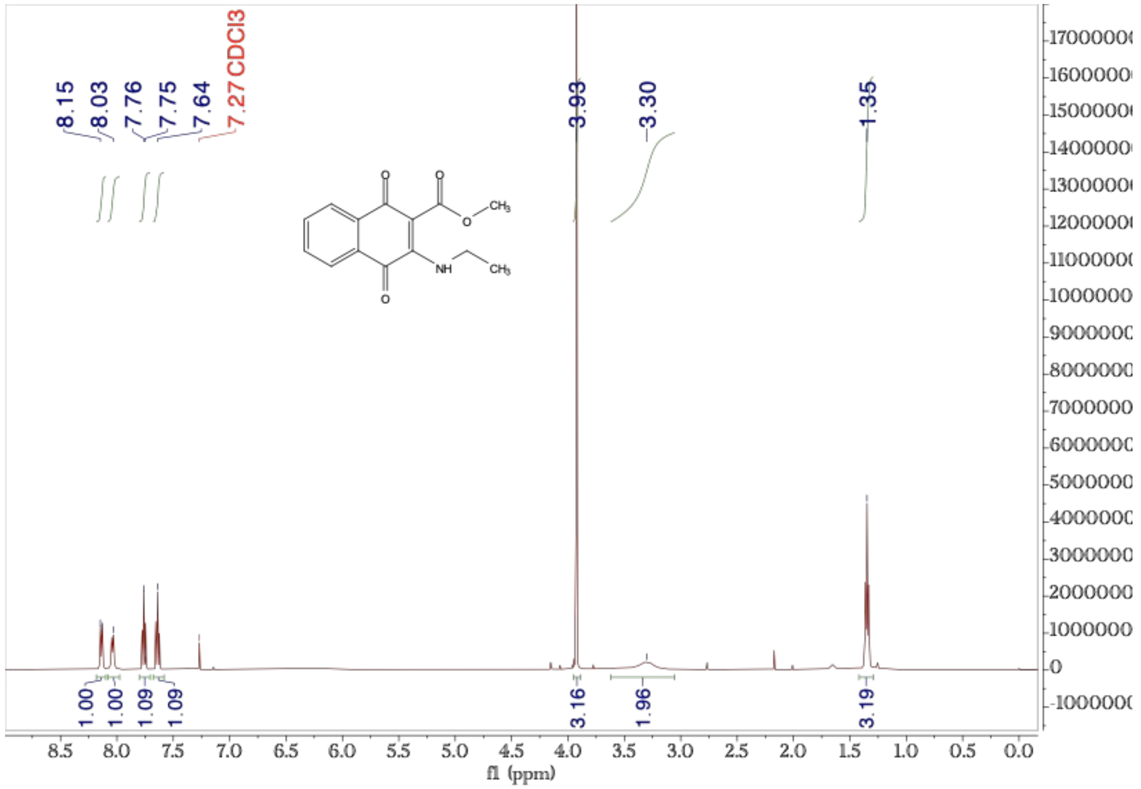

Figure S71: ^{1}H NMR Spectra (500 MHz) for 3-Ethylamine-DHNA methyl ester (**4g**) in $CDCl_3$

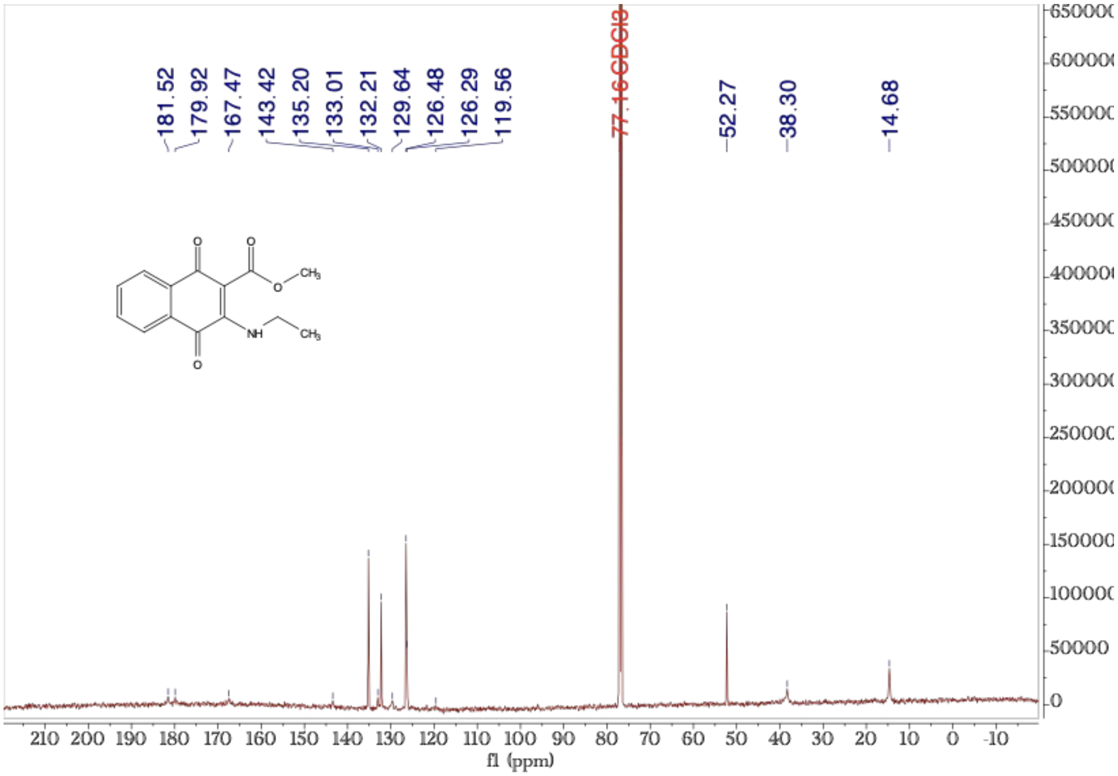

Figure S72: ^{13}C NMR Spectra (125 MHz) for 3-Ethylamine-DHNA methyl ester (**4g**) in $CDCl_3$

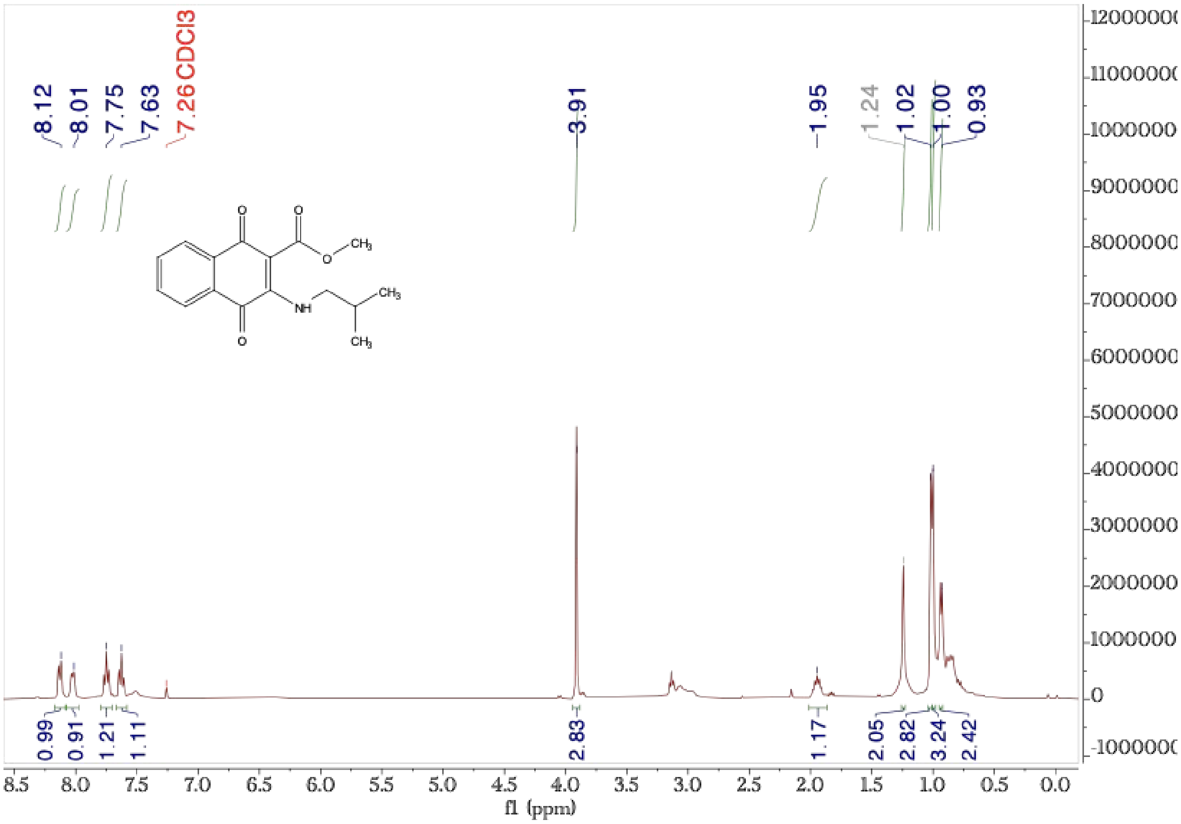

Figure S73: ^{1}H NMR Spectra (500 MHz) for 3-Isobutylamine-DHNA methyl ester (**4h**) in $CDCl_3$

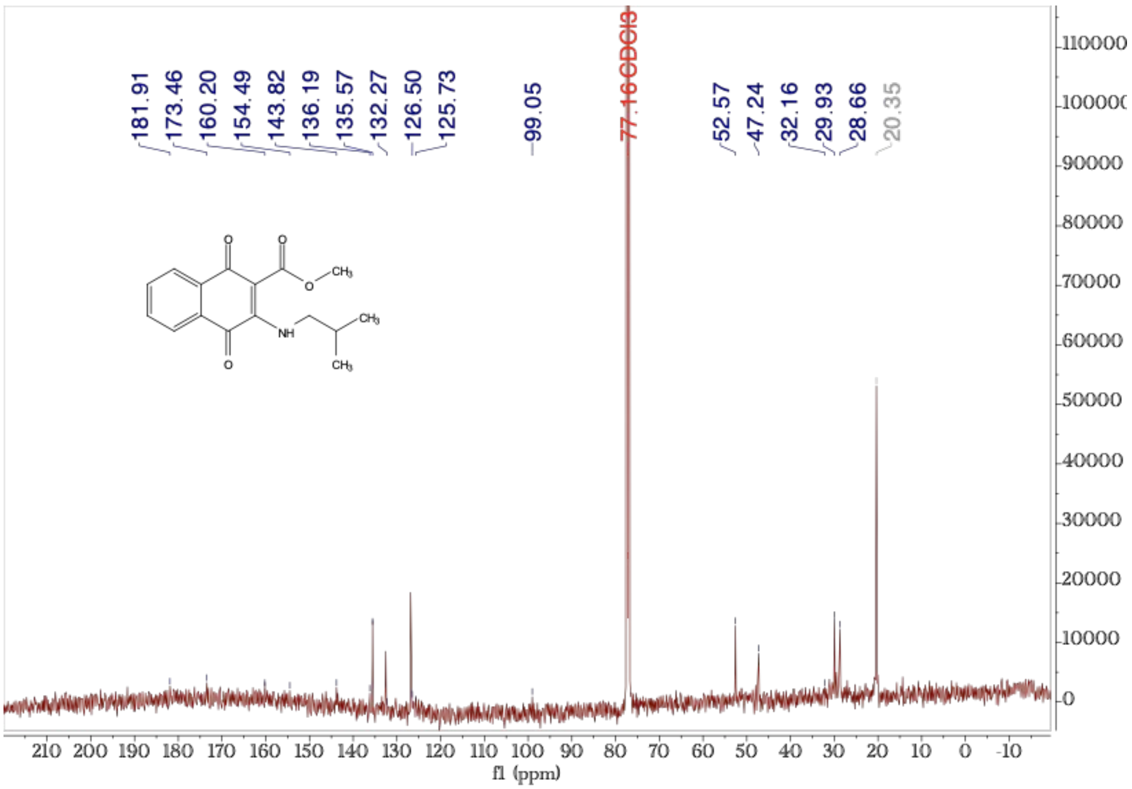

Figure S74: ^{13}C NMR Spectra (125 MHz) for 3-Isobutylamine-DHNA methyl ester (**4h**) in $CDCl_3$

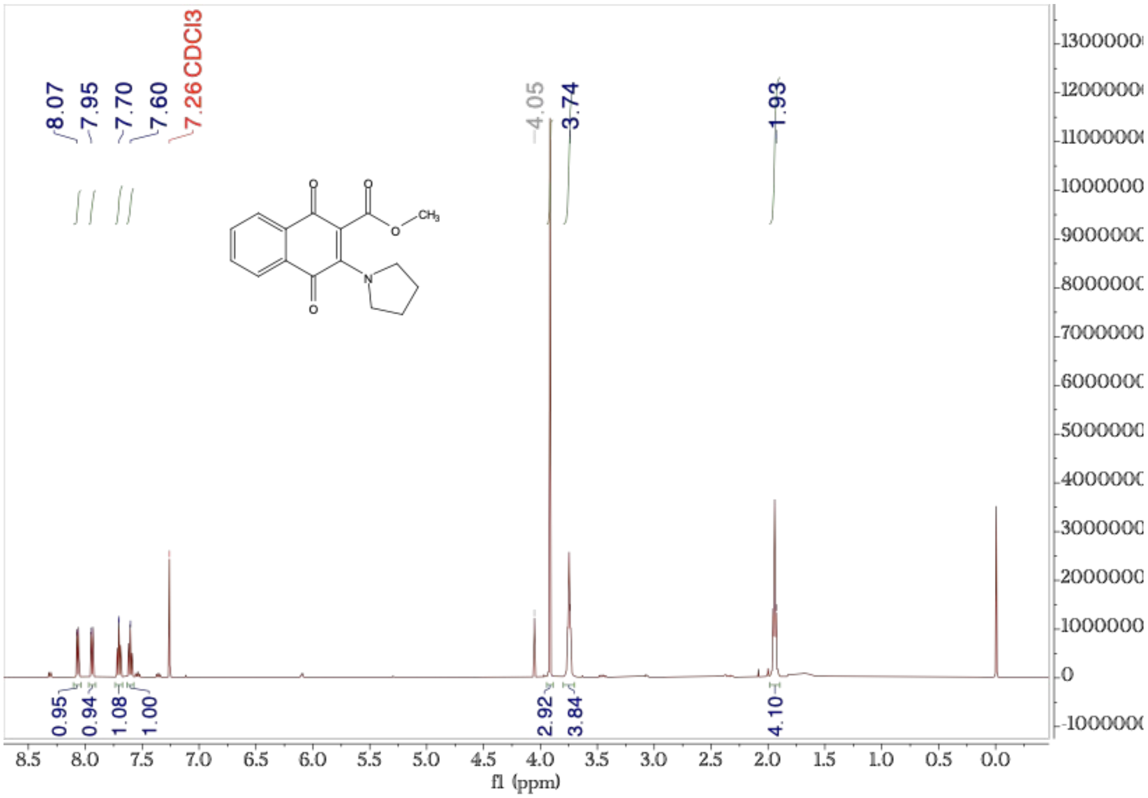

Figure S75: ^{1}H NMR Spectra (500 MHz) for 3-Pyrrolidine-DHNA methyl ester (**4i**) in $CDCl_3$

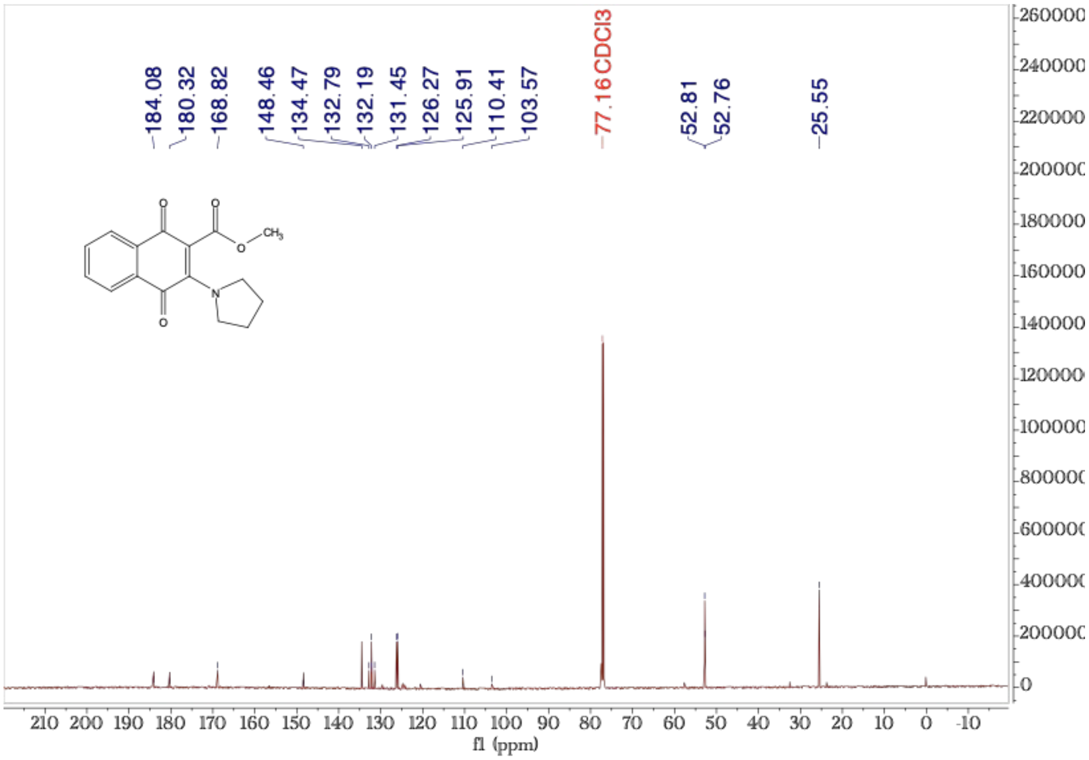

Figure S76: ^{13}C NMR Spectra (125 MHz) for 3-Pyrrolidine-DHNA methyl ester (**4i**) in $CDCl_3$

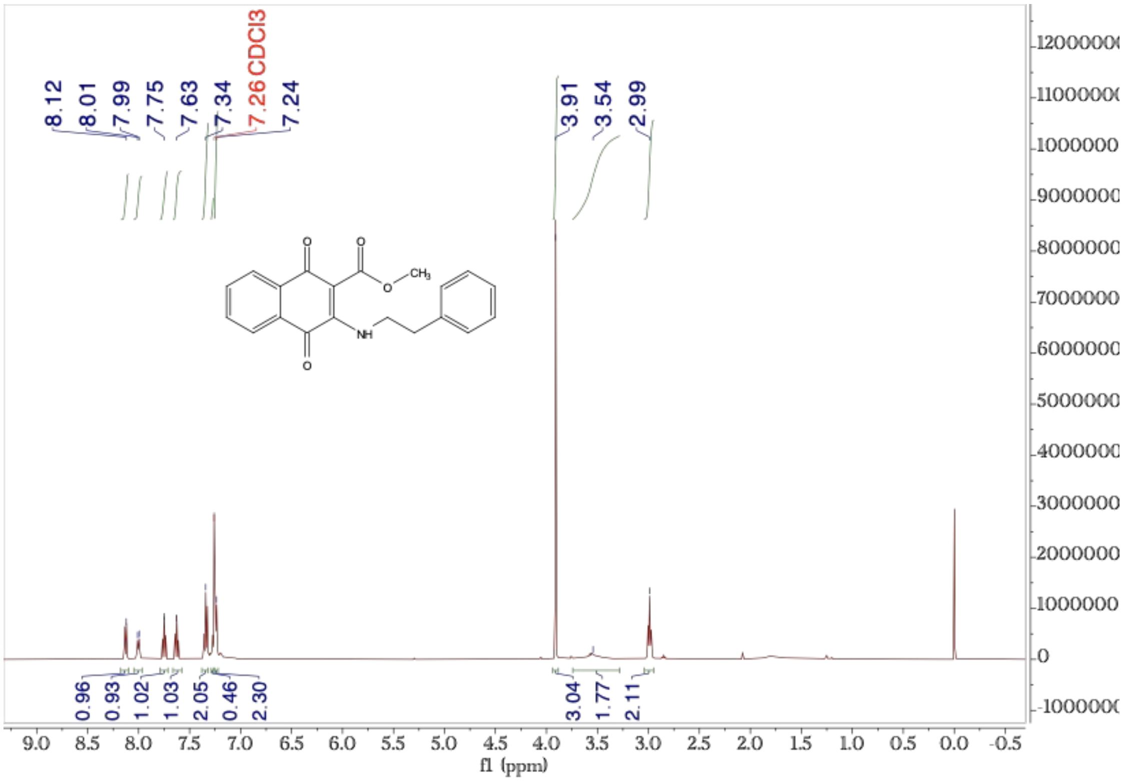

Figure S77: ^{1}H NMR Spectra (500 MHz) for 3-Phenethylamine-DHNA methyl ester (**4j**) in $CDCl_3$

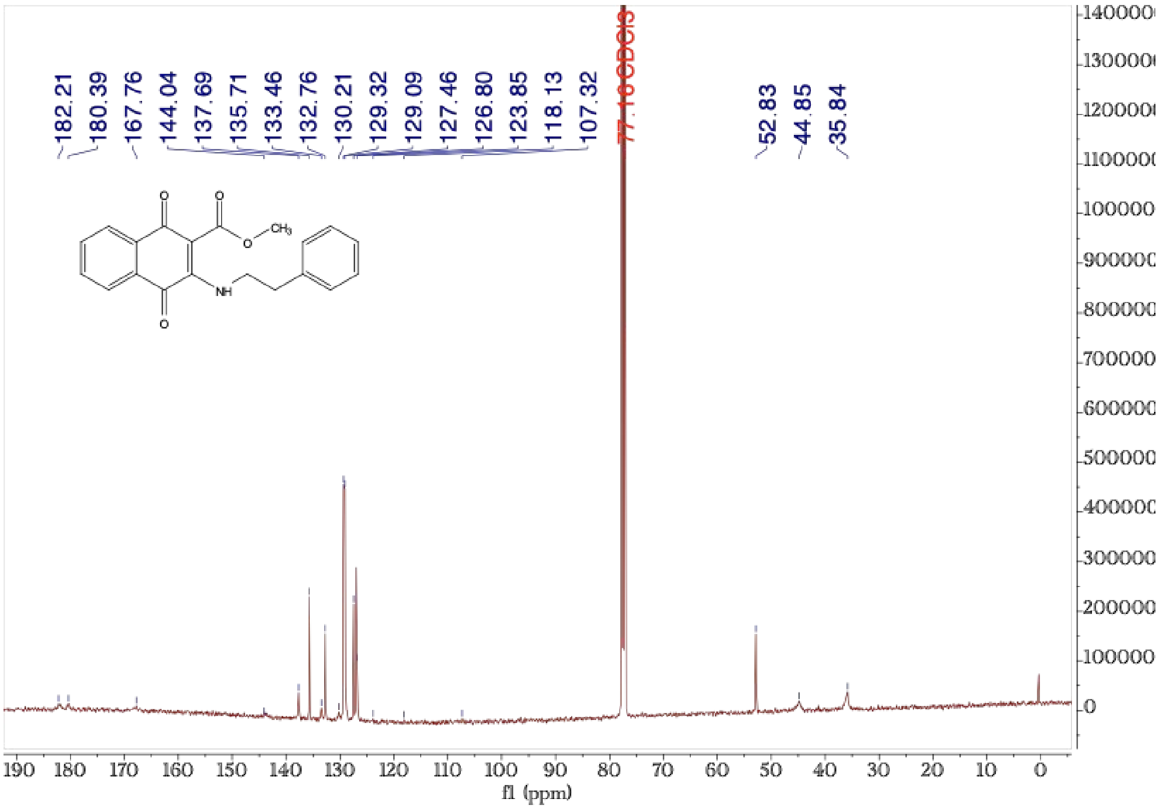

Figure S78: ^{13}C NMR Spectra (125 MHz) for 3-Phenethylamine-DHNA methyl ester (**4j**) in $CDCl_3$

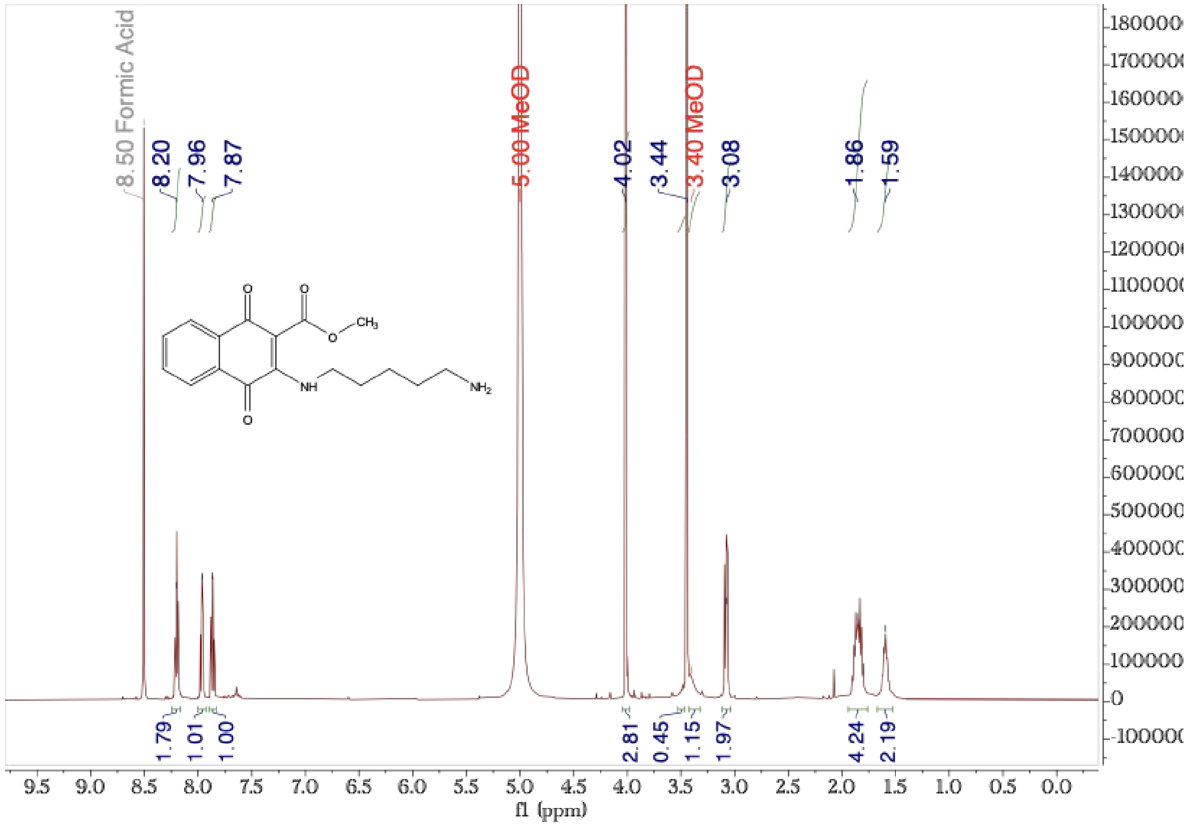

Figure S79: ^{1}H NMR Spectra (500 MHz) for 3-Cadaverine-DHNA methyl ester (**4k**) in CD_3OD

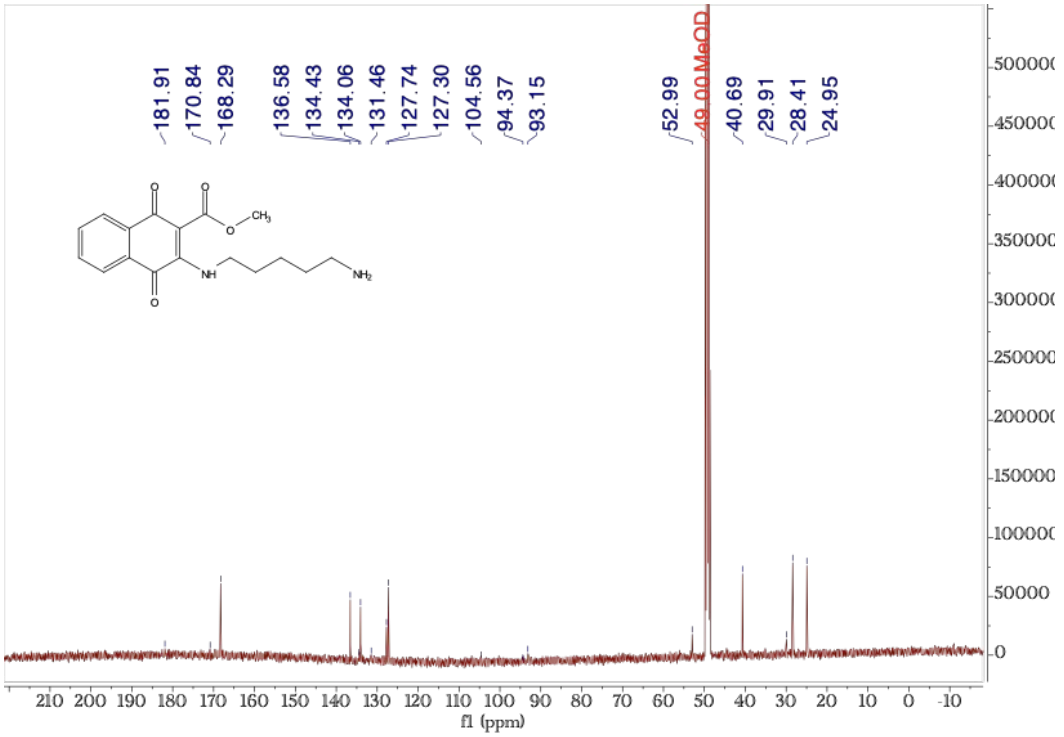

Figure S80: ^{13}C NMR Spectra (125 MHz) for 3-Cadaverine-DHNA methyl ester (**4k**) in CD_3OD

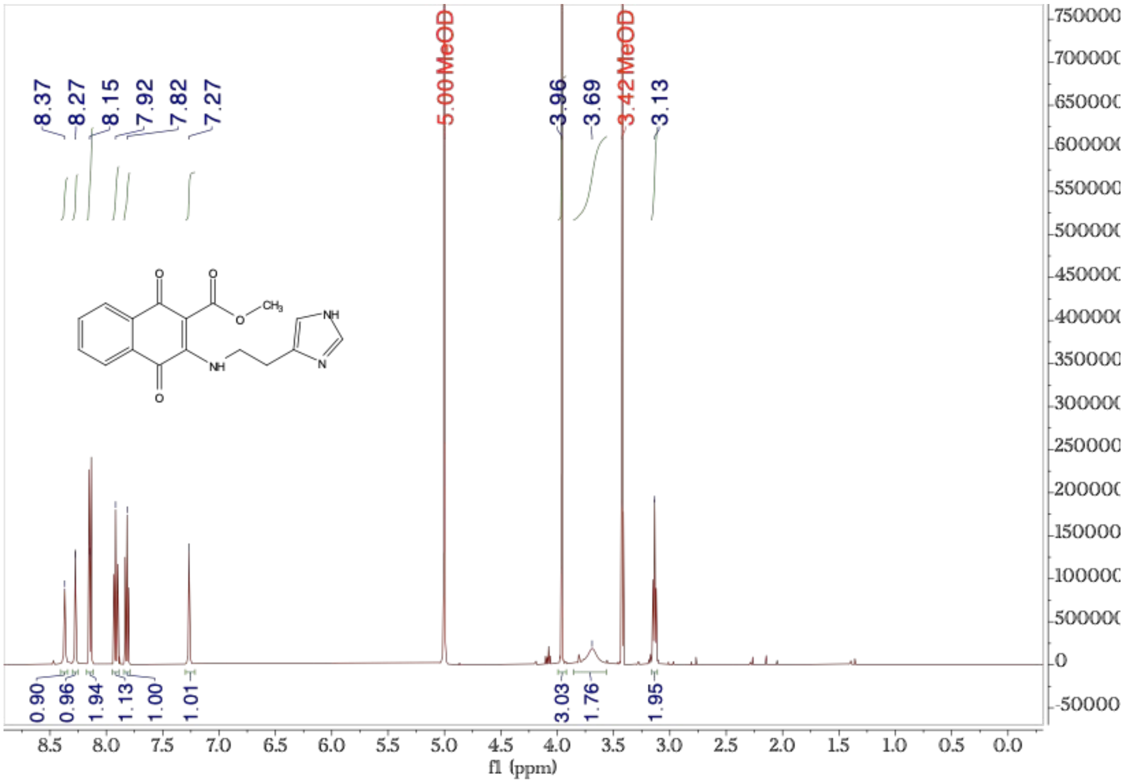

Figure S81: ^{1}H NMR Spectra (500 MHz) for 3-Histamine-DHNA methyl ester (**4l**) in CD_3OD

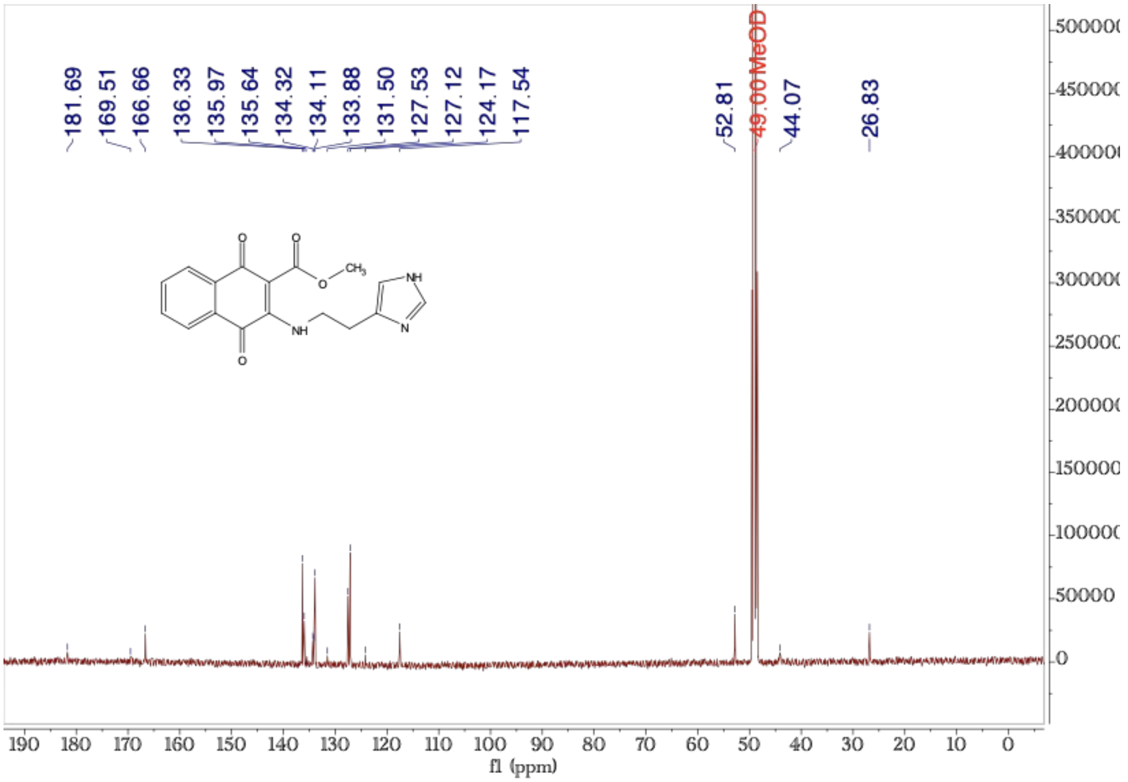

Figure S82: ^{13}C NMR Spectra (125 MHz) for 3-Histamine-DHNA methyl ester (**4l**) in CD_3OD

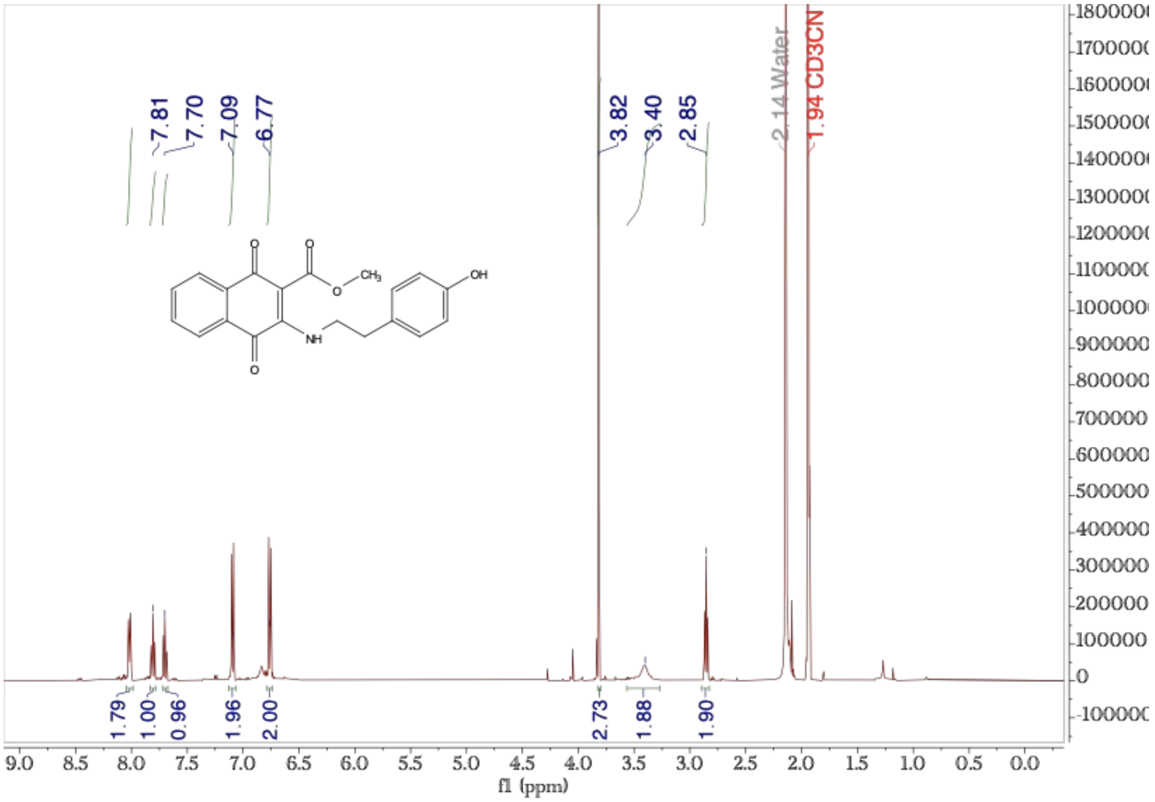

Figure S83: ^{1}H NMR Spectra (400 MHz) for 3-Tyramine-DHNA methyl ester (**4m**) in CD_3CN

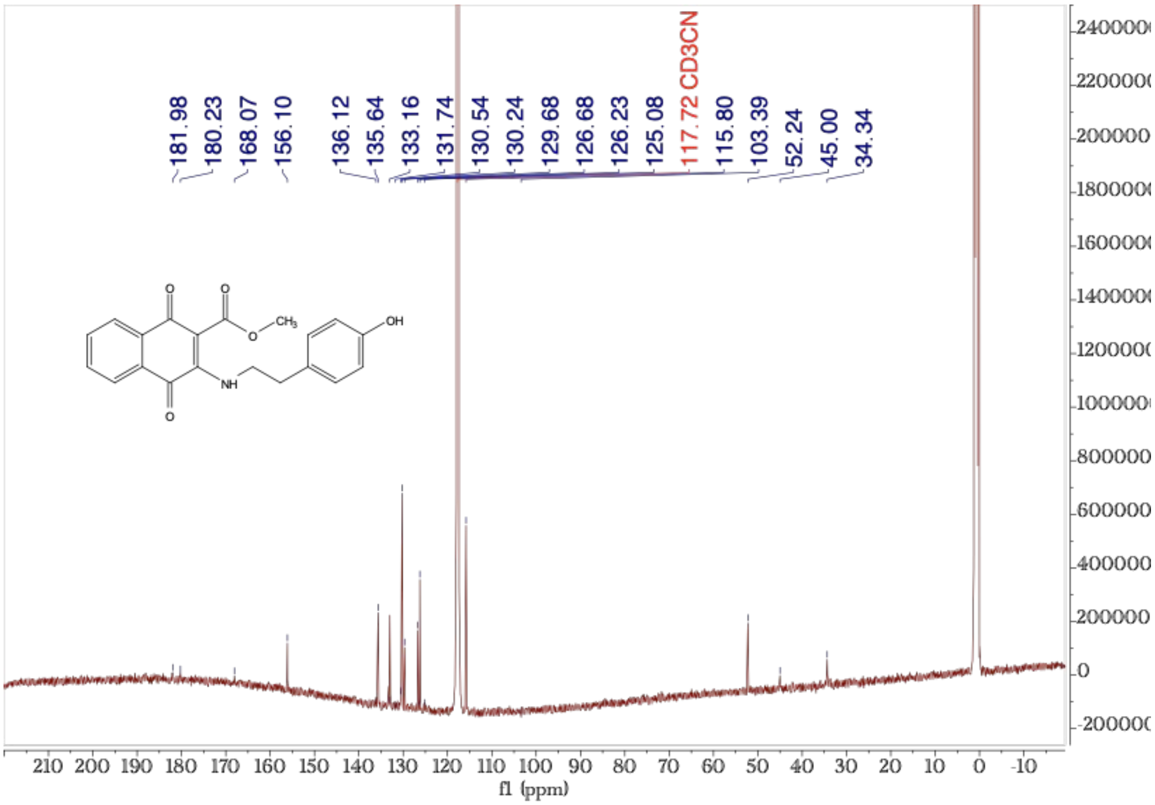

Figure S84: ^{13}C NMR Spectra (125 MHz) for 3-Tyramine-DHNA methyl ester (**4m**) in CD_3CN

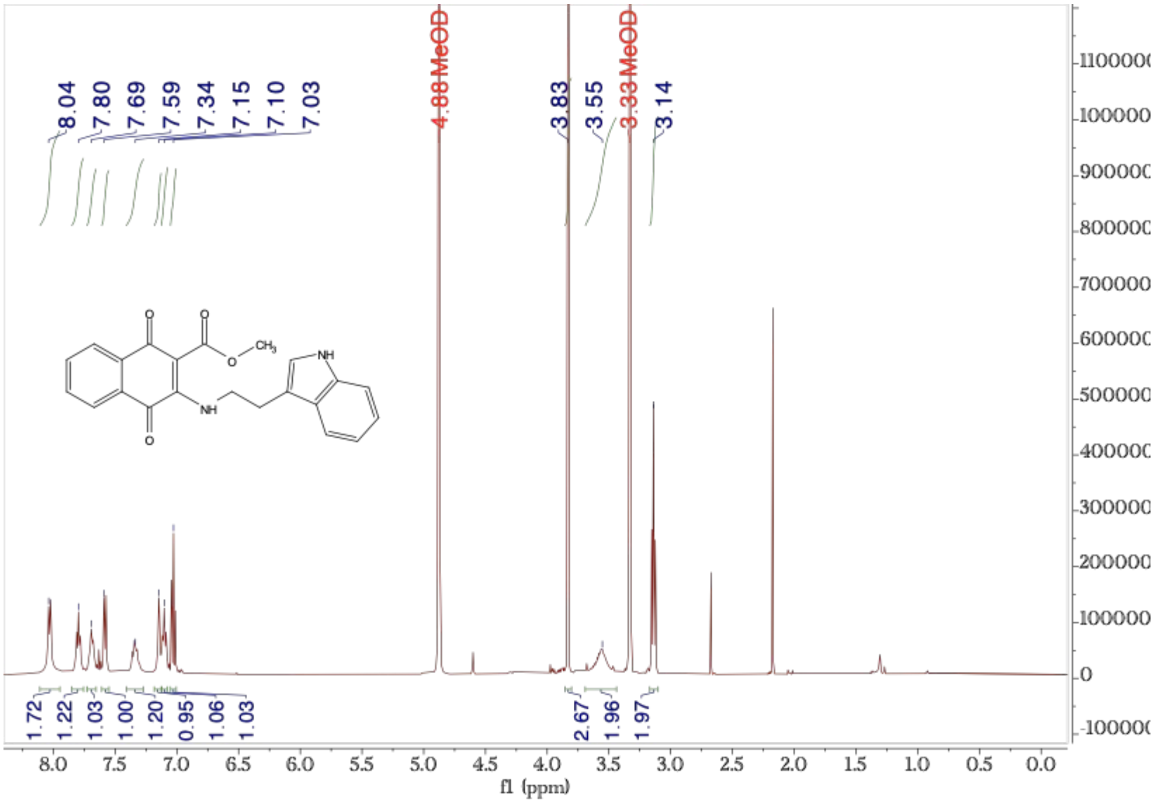

Figure S85: ^{1}H NMR Spectra (400 MHz) for 3-Tryptamine-DHNA methyl ester (**4n**) in CD_3OD

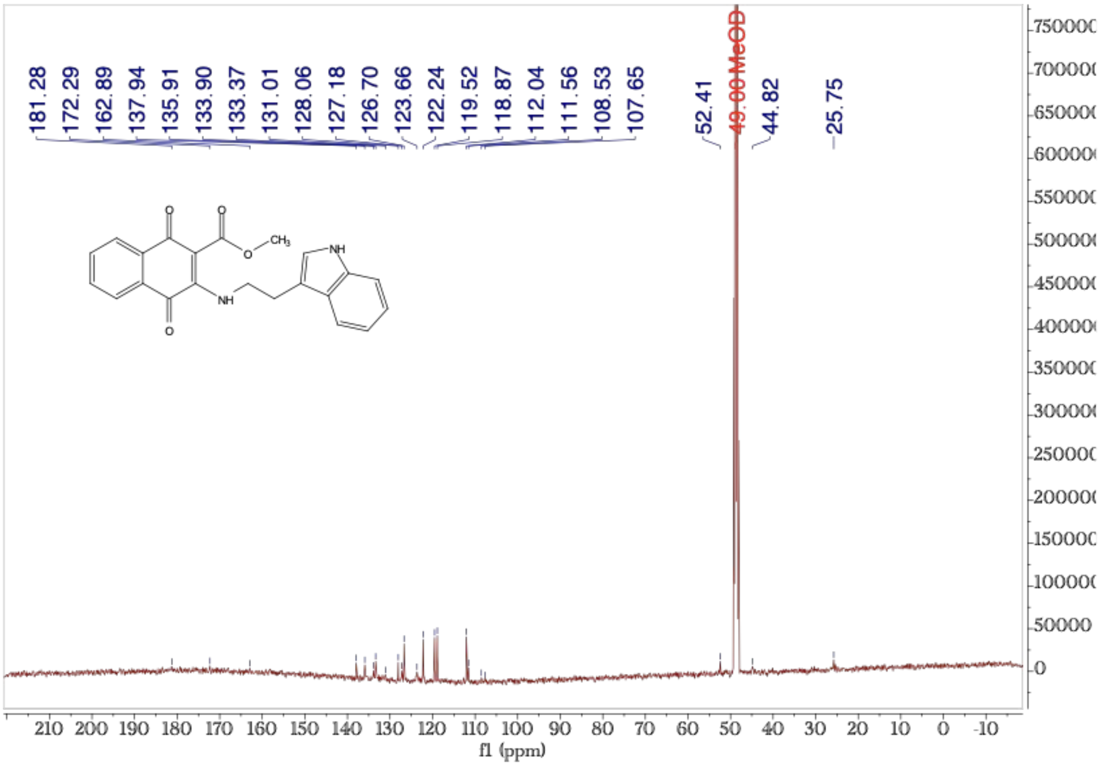

Figure S86: ^{13}C NMR Spectra (125 MHz) for 3-Tryptamine-DHNA methyl ester (**4n**) in CD_3OD

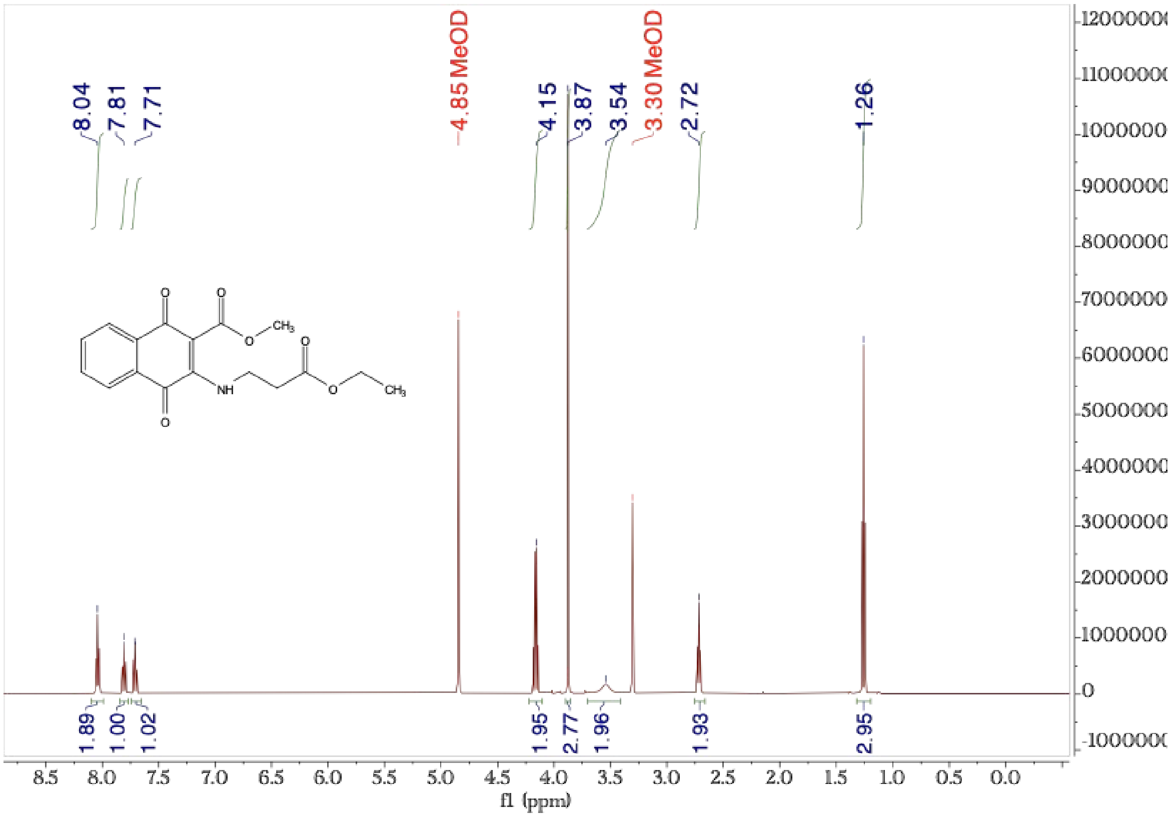

Figure S87: ^{1}H NMR Spectra (400 MHz) for 3-β-alanine-ethyl ester-DHNA methyl ester (**4o**)in CD_3OD

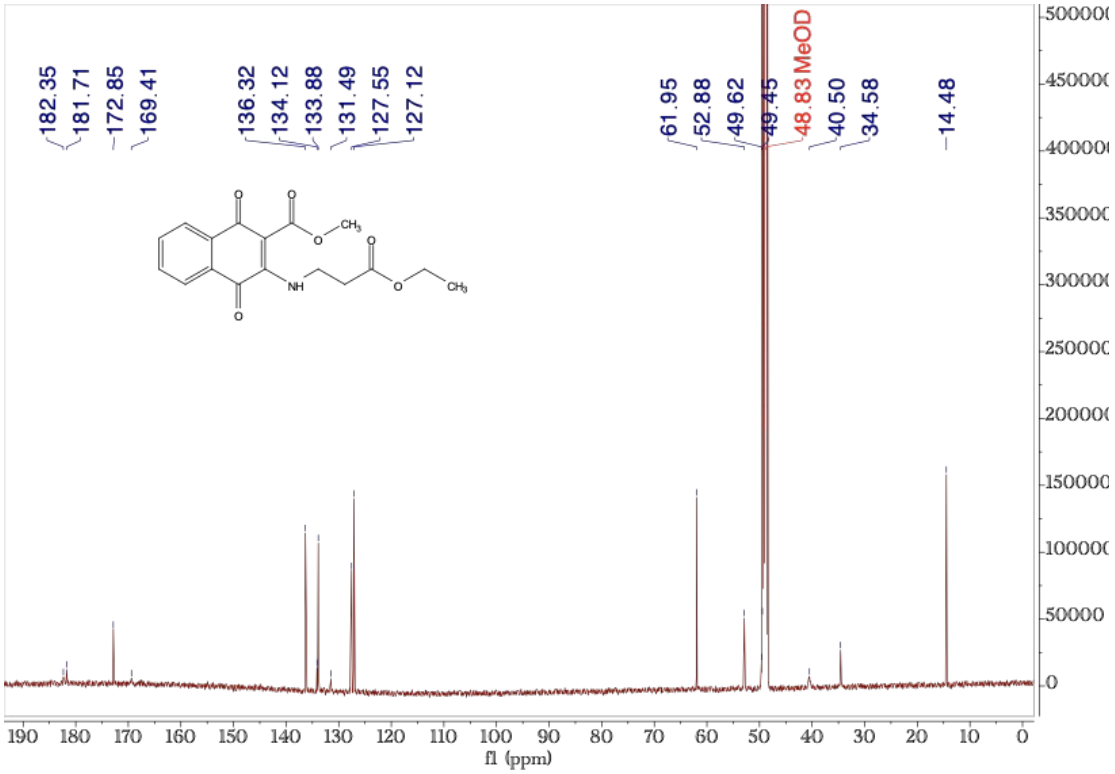

Figure S88: ^{13}C NMR Spectra (125 MHz) for 3-β-alanine-ethyl ester-DHNA methyl ester (**4o**) in CD_3OD

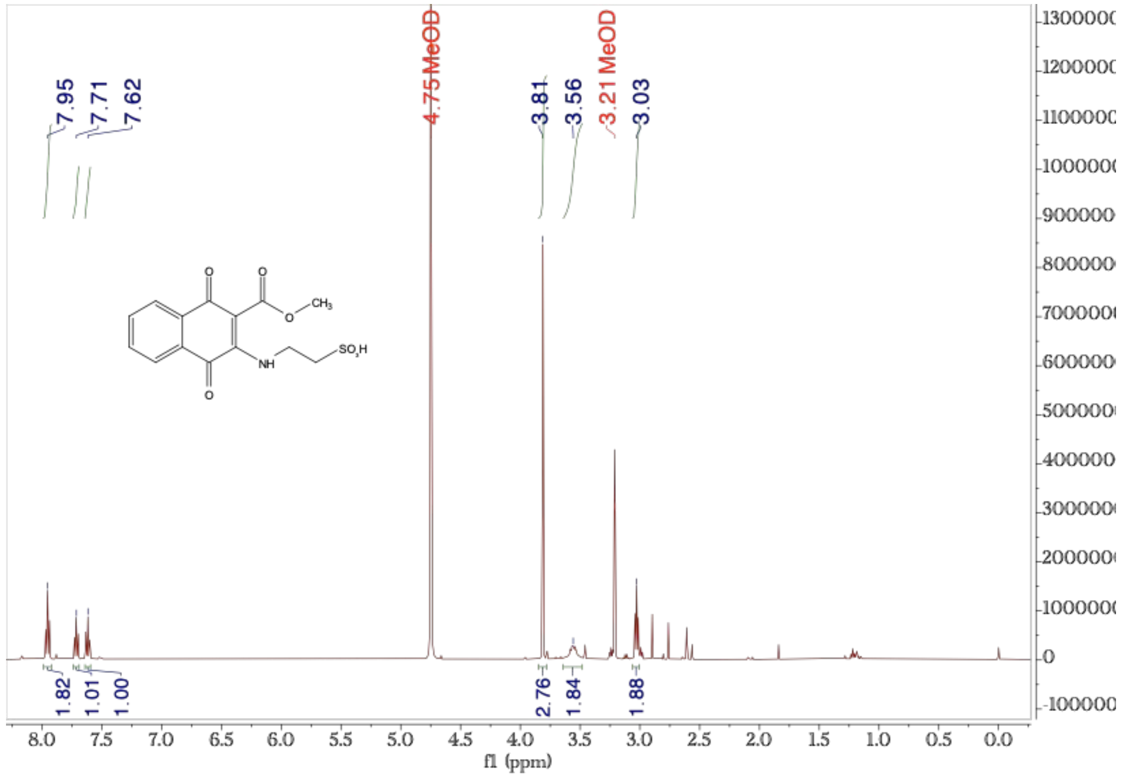

Figure S89: ^{1}H NMR Spectra (400 MHz) for 3-Taurine-DHNA methyl ester (**4p**) in CD_3OD

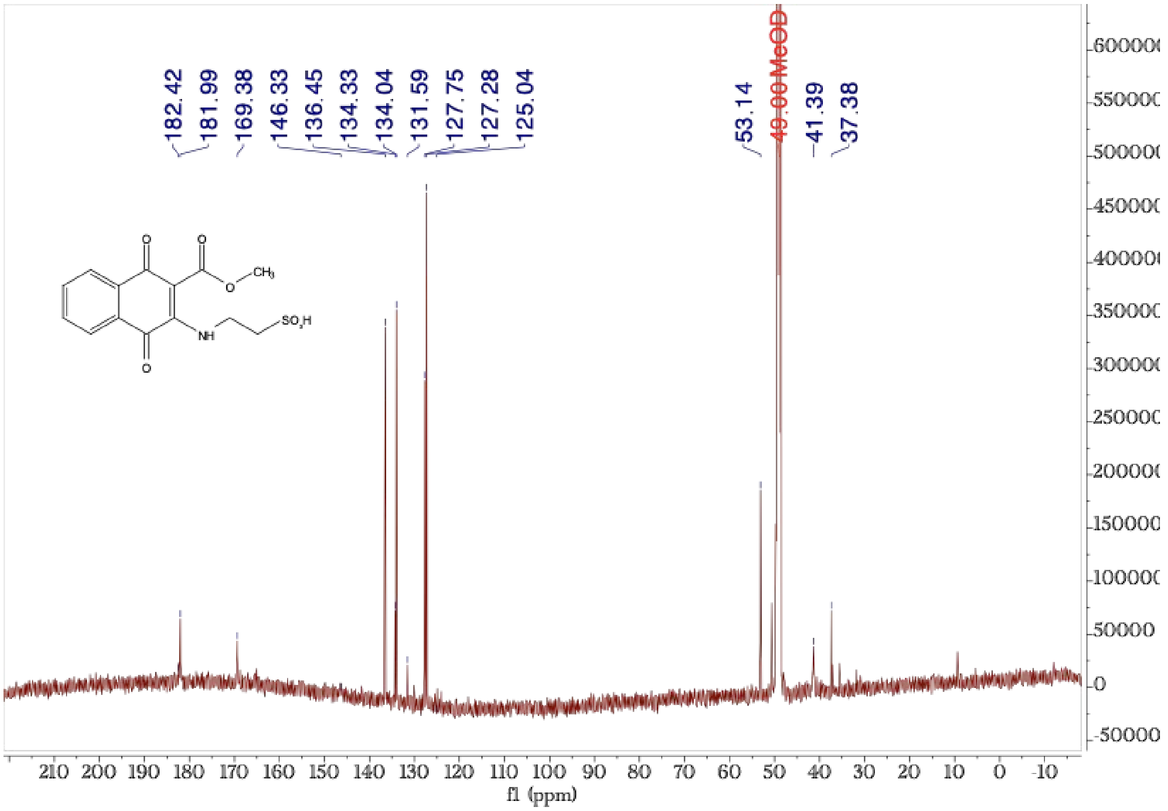

Figure S90: ^{13}C NMR Spectra (100 MHz) for 3-Taurine-DHNA methyl ester (**4p**) in CD_3OD

g. XYZ Coordinates

Menadione ground (**1**)

C 0.00000000 0.00000000 0.00000000
C -1.31879500 -0.74172500 0.00000000
C -1.34937300 -2.08301000 -0.00000000
C -2.49713700 -2.80239300 0.00000000
O -2.44723600 -4.01320100 -0.00000000
C -3.65597800 -2.09520600 -0.00000000
C -4.82548500 -2.76190600 -0.00000000
C -5.98542600 -2.08715300 0.00000000
C -5.98000600 -0.74644800 -0.00000000
C -4.81550700 -0.07915300 0.00000000
C -3.64793600 -0.75036200 0.00000000
C -2.48494700 -0.04617000 -0.00000000
O -2.45456000 1.16531600 0.00000000
H -4.86008800 1.02398900 -0.00000000
H -6.93559400 -0.19332900 -0.00000000
H -6.94482000 -2.63361800 -0.00000000
H -4.87294100 -3.86492600 -0.00000000
H -0.39481600 -2.63704700 -0.00000000
H 0.88016800 -0.68243700 -0.00000000
H 0.08542200 0.64382900 -0.90518100
H 0.08542200 0.64382900 0.90518100

Menadione radical anion (**1**)

C 0.00000000 0.00000000 0.00000000
C 1.46259300 0.38755500 0.00000000
C 2.41953900 -0.55278200 0.00000000
C 3.74393400 -0.26843600 -0.00000000
O 4.55222300 -1.17132800 -0.00000000
C 4.08179400 1.04643200 -0.00000000
C 5.38505200 1.38370700 -0.00000000
C 5.74631100 2.67608700 -0.00000000
C 4.80780600 3.63354600 -0.00000000
C 3.50772400 3.30018900 -0.00000000
C 3.13852400 2.00503000 -0.00000000
C 1.81380300 1.69917900 0.00000000
O 0.94747800 2.54658800 0.00000000
H 2.77067500 4.12218000 -0.00000000
H 5.10734300 4.69626300 -0.00000000
H 6.81510800 2.95309500 -0.00000000
H 6.18800300 0.62596300 -0.00000000
H 2.12138100 -1.61543800 0.00000000
H -0.15531500 -1.10285700 0.00000000
H -0.51006400 0.40205300 0.90518100
H -0.51006400 0.40205300 -0.90518100

3-Amine-menadione ground **(1a)**

C 0.00000000 0.00000000 0.00000000
C -1.31629500 0.72221500 -0.00041500

C	-1.35893500	2.09242300	-0.00095500
C	-2.65330300	2.85254500	-0.00057100
C	-3.90957100	2.07965700	-0.00056200
C	-5.13708700	2.74202000	-0.00041600
C	-6.31944900	2.01235600	-0.00056700
C	-6.27459800	0.62023700	-0.00089000
C	-5.05052600	-0.04292300	-0.00099800
C	-3.86124100	0.67882500	-0.00082100
C	-2.54770900	-0.04688900	-0.00073400
O	-2.54347100	-1.28194700	-0.00083200
H	-5.00549000	-1.12365600	-0.00120000
H	-7.19519900	0.04944100	-0.00105700
H	-7.27273600	2.52601000	-0.00043600
H	-5.15228600	3.82402000	-0.00020100
O	-2.62716400	4.07539400	-0.00027700
N	-0.26996500	2.88308600	-0.00164300
H	0.66437700	2.51150100	-0.00158000
H	-0.40249900	3.88301000	-0.00132000
H	0.59780900	0.25732800	0.88120400
H	-0.16022000	-1.07554800	0.00102300
H	0.59756400	0.25555300	-0.88190900

3-Amine-menadione radical anion **(1a)**

C	0.00000000	0.00000000	0.00000000
C	1.32681700	0.71046400	0.01222100
C	1.36822300	2.09985900	0.00568700
C	2.60288600	2.85533700	0.01883100
C	3.83909400	2.09390500	0.01477500
C	5.08514000	2.75354500	0.01266000
C	6.26826800	2.04167100	0.01204600
C	6.24185300	0.63659000	0.01463100
C	5.02871500	-0.02774100	0.01878900
C	3.81168900	0.67388000	0.01897200
C	2.53572500	-0.06172300	0.02622900
O	2.53050000	-1.33105900	0.04112100
H	4.99178600	-1.10974900	0.02236800
H	7.16979500	0.07606700	0.01375700
H	7.21752900	2.56572500	0.00936700
H	5.09159000	3.83647500	0.01099500
O	2.57364000	4.12893800	0.04014800
N	0.21175000	2.86444800	-0.05798400
H	-0.62061000	2.47325200	0.35780400
H	0.38092000	3.83298100	0.18544700
H	-0.61736800	0.31013500	-0.85049700

H 0.15551300 -1.07542100 -0.06015600
H -0.58573500 0.20303900 0.90552100

3-[(*S*)-amino-2-propanol]-menadione ground (**1b**)
C 0.00000000 0.00000000 0.00000000
C -0.95258900 -1.17844000 0.10353800
C -0.62940500 -2.41038600 -0.34401400
C -1.54417100 -3.41146200 -0.29103000
C -2.76195800 -3.16580300 0.25106600
C -3.66524400 -4.16152400 0.31818800
C -4.87371100 -3.93675400 0.85687400
C -5.18108900 -2.71985200 1.32872000
C -4.28139300 -1.72608700 1.26168500
C -3.06759000 -1.94809900 0.72266500
C -2.17093200 -0.93159200 0.65480200
O -2.43572000 0.17772700 1.06515000
H -4.57822300 -0.73996300 1.65941700
H -6.17619700 -2.53725100 1.77083300
H -5.61591600 -4.75226400 0.91295900
H -3.45053800 -5.17734800 -0.05719900
O -1.22500500 -4.49528800 -0.72677400
N 0.60781500 -2.72911800 -0.88967000
C 1.71106200 -2.71912000 0.04807900
C 1.66381600 -3.93489900 0.98691300
C 2.91552700 -4.03183300 1.86921600
H 2.86247600 -4.91779600 2.54324900
H 3.83740500 -4.12876500 1.25104600
H 3.02330300 -3.12540900 2.50827700
H 0.75381800 -3.88661200 1.63523100
O 1.55570500 -5.10238400 0.20523000
H 0.69365400 -5.09657400 -0.17127400
H 2.65078400 -2.76015500 -0.55070400
H 1.75417800 -1.78594100 0.65354900
H 0.58093200 -3.59867100 -1.42373200
H 0.39726700 0.27188300 1.00479300
H 0.85959000 -0.17399900 -0.68435900
H -0.51445100 0.89193600 -0.42547400

3-[(*S*)-amino-2-propanol]-menadione Radical Anion (**1b**)
C 0.00000000 0.00000000 0.00000000
C 0.66994300 -1.35938300 -0.10059400
C 0.05118300 -2.49941200 0.27345300
C 0.72065900 -3.67906200 0.22889300
C 1.99530700 -3.69849100 -0.23166900
C 2.65573800 -4.86991300 -0.29075100
C 3.91643300 -4.90768200 -0.74935700
C 4.51804900 -3.77801000 -1.14919400
C 3.86067700 -2.60930000 -1.09008100
C 2.59538700 -2.56765200 -0.63132500
C 1.94599600 -1.37750700 -0.57021700

O 2.47814000 -0.34481800 -0.91585200
H 4.39563800 -1.70417900 -1.42670100
H 5.55551200 -3.81157400 -1.52547800
H 4.45974300 -5.86760200 -0.79871700
H 2.19547200 -5.82182000 0.02696000
O 0.13966600 -4.67541300 0.59787600
N -1.25892100 -2.54685800 0.73325900
C -2.26834800 -2.26015600 -0.26448100
C -2.43263300 -3.42665100 -1.25142500
C -3.61395800 -3.21222500 -2.20700200
H -3.71622700 -4.06677800 -2.91505900
H -4.57257200 -3.11702300 -1.64719300
H -3.47452300 -2.28619900 -2.81086700
H -1.49447000 -3.56564000 -1.84411000
O -2.63938200 -4.61121700 -0.51671700
H -1.82431500 -4.81134100 -0.09182000
H -3.23056200 -2.10591900 0.27742100
H -2.06224700 -1.32367200 -0.82998700
H -1.46192800 -3.41559100 1.22940200
H -0.25995300 0.38439200 -1.01292500
H -0.91957800 0.00412600 0.62600100
H 0.67176400 0.73972000 0.49273300

3-[(*R*)-amino-2-propanol]-menadione ground (**1c**)
C 0.00000000 0.00000000 0.00000000
C -1.32357600 -0.71518800 -0.20634700
C -1.40266600 -2.05617100 -0.33035900
C -2.60934600 -2.66165000 -0.46450000
C -3.72688500 -1.89479300 -0.49670800
C -4.92709300 -2.48761300 -0.63785200
C -6.04361600 -1.74395200 -0.67527100
C -5.96326700 -0.40942100 -0.57298400
C -4.76715300 0.18297400 -0.43200300
C -3.64452600 -0.55965600 -0.39388500
C -2.44332000 0.05480400 -0.24469000
O -2.35200300 1.25891300 -0.14067100
H -4.74870400 1.28368500 -0.34943600
H -6.88323300 0.20025200 -0.60386500
H -7.02887300 -2.22865900 -0.79077200
H -5.03519300 -3.58272300 -0.72690600
O -2.63199200 -3.86998900 -0.55518200
N -0.29375300 -2.89081600 -0.32567600
C 0.48767200 -2.82944000 -1.54182800
C 1.76837700 -3.66853800 -1.40149700
C 2.71801200 -3.11497100 -0.32963100
H 3.00917200 -2.06333200 -0.55460700
H 2.24461900 -3.13167200 0.67870300
H 3.65091600 -3.72198100 -0.27281900
O 2.43880800 -3.68720000 -2.64232700
H 3.16936500 -4.27640400 -2.56132800
H 1.50478900 -4.72785800 -1.15727100

H 0.76141500 -1.78442200 -1.81477800
H -0.12983400 -3.24008300 -2.37587400
H -0.55198800 -3.85241700 -0.10117800
H 0.84750200 -0.67697600 0.24569900
H 0.27537500 0.58094600 -0.90987000
H -0.05999600 0.70307300 0.86214300

3-[(*R*)-amino-2-propanol]-menadione Radical Anion (**1c**)
C 0.00000000 0.00000000 0.00000000
C 0.82222800 1.26467900 0.17539000
C 0.27938900 2.49488400 0.06583800
C 1.05836700 3.59972500 0.18147900
C 2.38176900 3.44441900 0.43235600
C 3.15973300 4.53591700 0.55660700
C 4.47095800 4.40161300 0.80886200
C 5.00639300 3.17903800 0.93772600
C 4.23214200 2.08963400 0.81367900
C 2.91598900 2.22047800 0.56084900
C 2.14784200 1.10871800 0.43297700
O 2.61532200 -0.00465000 0.53749700
H 4.71576700 1.10349500 0.92556300
H 6.08537700 3.07090900 1.14519900
H 5.11034900 5.29598700 0.91109300
H 2.75724000 5.55934500 0.45897200
O 0.53322700 4.68528700 0.06061000
N -1.07001500 2.71430100 -0.17131200
C -1.91819200 2.45076900 0.97043000
C -3.38408700 2.66598600 0.56498200
C -4.34685600 2.48553700 1.74593900
H -5.40143300 2.65957400 1.43019600
H -4.28135500 1.45245500 2.15827400
H -4.11477100 3.19946100 2.56924900
O -3.51686800 3.97082100 0.04718600
H -4.37803800 4.04331800 -0.32567400
H -3.65816100 1.95679400 -0.25571600
H -1.78714800 1.41716200 1.36511200
H -1.65267600 3.16336600 1.78688900
H -1.24585900 3.64895600 -0.54195700
H -1.01334000 0.17232300 -0.42538300
H 0.49250200 -0.69709700 -0.71607700
H -0.11952700 -0.52443300 0.97567100

3-Aminoethanol-menadione ground (**1d**)
C 0.00000000 0.00000000 0.00000000
C -1.11151500 -0.98146000 0.32743900
C -0.91289800 -2.31581700 0.33185000
C -1.94445800 -3.16090400 0.58194300
C -3.16695100 -2.64040000 0.85129700
C -4.19346900 -3.47221500 1.10859500
C -5.40946300 -2.97431600 1.38162400
C -5.60156600 -1.64742400 1.39916600

C -4.57898400 -0.81695400 1.14219400
C -3.35748700 -1.31246900 0.86744300
C -2.33563000 -0.45868200 0.60383500
O -2.49279200 0.74314100 0.60479400
H -4.78326500 0.26766100 1.16543200
H -6.60278500 -1.24013800 1.62434400
H -6.25255900 -3.65513000 1.59288500
H -4.07587400 -4.56994800 1.10566600
O -1.72201300 -4.35205000 0.55831500
N 0.31857800 -2.90671600 0.08527900
C 1.26081500 -2.76590600 1.17436600
C 2.62419400 -3.33292500 0.75889400
O 3.52207200 -3.23290300 1.83938500
H 4.34964200 -3.58084900 1.55379800
H 2.54826900 -4.41009700 0.47457100
H 3.05417100 -2.76224500 -0.09922500
H 1.38426600 -1.70385000 1.48904700
H 0.87715100 -3.33612400 2.05371600
H 0.21378700 -3.88470500 -0.18752500
H 0.91254600 -0.47253000 -0.42603300
H -0.33110300 0.72825700 -0.77542300
H 0.29871200 0.56772000 0.91085300

3-Aminoethanol-menadione Radical Anion (**1d**)
C 0.00000000 0.00000000 0.00000000
C -0.93242200 -1.18784500 0.15986700
C -0.50928000 -2.45909400 0.00222000
C -1.38508200 -3.49016900 0.10560500
C -2.68211800 -3.22129300 0.39434200
C -3.55531800 -4.23950100 0.50673500
C -4.84214800 -3.99210900 0.79648300
C -5.25791200 -2.72994300 0.97518000
C -4.38836500 -1.71375100 0.86279700
C -3.09649500 -1.95766700 0.57184400
C -2.23154400 -0.91794100 0.45581300
O -2.59132100 0.23002900 0.60347700
H -4.77489800 -0.69111300 1.01597600
H -6.31700500 -2.52890700 1.21378200
H -5.55972500 -4.82605600 0.88903200
H -3.25224400 -5.29211600 0.36888500
O -0.96248300 -4.61381700 -0.06081200
N 0.80828700 -2.79824200 -0.27253400
C 1.69717600 -2.64588400 0.85901900
C 3.14302400 -2.91749600 0.42464500
O 3.99528500 -2.81128500 1.54089700
H 4.87402900 -2.97545400 1.24365100
H 3.25591600 -3.94548400 0.00345800
H 3.48152500 -2.17543500 -0.33803900
H 1.63325600 -1.62870000 1.31010300
H 1.40392500 -3.38253300 1.64448400
H 0.87566400 -3.73565000 -0.67077000

H 0.98570200 -0.25328300 -0.44918700
H -0.43939700 0.75792900 -0.68828900
H 0.18459000 0.48527800 0.98570800

3-Aminopropanol-menadione ground (**1e**)
C 0.00000000 0.00000000 0.00000000
C -0.88214900 -1.23501300 0.04941700
C -0.53135200 -2.39491300 -0.54419200
C -1.37972300 -3.45423200 -0.52651200
C -2.56283400 -3.33731200 0.12497300
C -3.40234600 -4.38906100 0.15737900
C -4.57547700 -4.29271300 0.80191200
C -4.91136100 -3.14808900 1.41418800
C -4.07545000 -2.09841800 1.38216400
C -2.89711700 -2.19158900 0.73725900
C -2.06577500 -1.11903100 0.70832400
O -2.35774600 -0.07286500 1.24623800
H -4.39395500 -1.17552000 1.89749800
H -5.87732300 -3.07116700 1.94339100
H -5.26482000 -5.15475100 0.82977000
H -3.16199100 -5.34886400 -0.33245500
O -1.03670700 -4.46883900 -1.09378700
N 0.67426500 -2.58795700 -1.20550600
C 1.83551400 -2.64264800 -0.34186800
C 1.86915700 -3.92782400 0.50111800
C 3.15387300 -4.02256600 1.33566100
O 3.12824800 -5.20345800 2.10321400
H 3.93316300 -5.24236900 2.59139400
H 4.06043500 -4.05778300 0.68511800
H 3.24739900 -3.16051000 2.03894300
H 0.98982700 -3.95367100 1.18789100
H 1.79725500 -4.81724300 -0.16969900
H 2.73486300 -2.62085200 -1.00093700
H 1.90554100 -1.75605300 0.32845200
H 0.63527500 -3.40042900 -1.82200600
H 0.81684300 -0.05956100 -0.75282500
H -0.58820400 0.89781000 -0.29869600
H 0.45512700 0.19546600 0.99801500

3-Aminopropanol-menadione Radical Anion (**1e**)
C 0.00000000 0.00000000 0.00000000
C 0.63014300 -1.37957500 0.07468200
C 0.05313700 -2.39971300 0.74339700
C 0.68319500 -3.59838500 0.83512500
C 1.87835500 -3.76116800 0.21629700
C 2.50143400 -4.95195500 0.29247600
C 3.68273700 -5.13241100 -0.31818000
C 4.24252100 -4.12591800 -1.00482300
C 3.62258500 -2.93787700 -1.08110400
C 2.43677900 -2.75329700 -0.47049300
C 1.82576300 -1.54397800 -0.55145300

O 2.32226400 -0.61944800 -1.15791200
H 4.12111800 -2.13715000 -1.65473800
H 5.21472300 -4.27674300 -1.50595200
H 4.19468700 -6.10875200 -0.25699300
H 2.07298700 -5.80567400 0.84612500
O 0.14211400 -4.47997300 1.46672800
N -1.17886000 -2.30635500 1.37710200
C -2.31333900 -2.21035100 0.48205900
C -2.57719500 -3.53026700 -0.26066300
C -3.84102800 -3.44769700 -1.12775000
O -4.02811800 -4.67064500 -1.80131900
H -4.81666000 -4.59607600 -2.31157700
H -4.74914200 -3.25742100 -0.50692300
H -3.75509600 -2.64379200 -1.89771500
H -1.70684200 -3.77809100 -0.91382100
H -2.68900100 -4.35949800 0.47832800
H -3.20361300 -1.96495800 1.10725500
H -2.20024200 -1.38419800 -0.25615700
H -1.30725400 -3.05848700 2.05497700
H -0.82657300 0.15780300 0.72759900
H 0.74372200 0.79146800 0.24844200
H -0.39122000 0.19609500 -1.02463400

3-Methylamine-menadione ground (**1f**)
C 0.00000000 0.00000000 0.00000000
C -1.17009900 -0.95046000 -0.18229500
C -1.00989600 -2.28967300 -0.21412300
C -2.08803800 -3.10518300 -0.33106100
C -3.32058700 -2.55071900 -0.43884200
C -4.39396500 -3.35343400 -0.56362500
C -5.62107600 -2.82176200 -0.67580600
C -5.77776700 -1.49016000 -0.66370800
C -4.70843300 -0.68863600 -0.53901400
C -3.47553700 -1.21821300 -0.42687600
C -2.40510000 -0.39368900 -0.29679800
O -2.52813800 0.81190300 -0.27648700
H -4.88449400 0.40121300 -0.53186000
H -6.78835500 -1.05507800 -0.75562100
H -6.50252100 -3.47873200 -0.77847800
H -4.30685100 -4.45388000 -0.57917900
O -1.89809600 -4.30209400 -0.34047500
N 0.22661400 -2.91495600 -0.12892100
C 1.02022400 -2.79413000 -1.32963500
H 2.01669000 -3.25681200 -1.14764400
H 0.52976000 -3.34752700 -2.16331900
H 1.17961700 -1.73872100 -1.64494600
H 0.13157900 -3.89134900 0.15299700
H 0.94509000 -0.49893400 0.30850500
H 0.19688300 0.56109900 -0.94224300
H -0.20990100 0.73547700 0.81009100

3-Methylamine-menadione Radical Anion (**1f**)
C 0.00000000 0.00000000 0.00000000
C 1.13216800 1.00784500 -0.09045000
C 0.91647200 2.33826800 -0.03030800
C 1.96130600 3.20319300 -0.06703900
C 3.21773000 2.70843200 -0.18882600
C 4.25847800 3.56097500 -0.23438700
C 5.50859000 3.08902300 -0.35905300
C 5.72080100 1.76745800 -0.43840200
C 4.68404500 0.91627200 -0.39276300
C 3.42824200 1.38590500 -0.26840700
C 2.39125600 0.51136600 -0.21905400
O 2.56441700 -0.68657500 -0.28194600
H 4.90554500 -0.16322100 -0.45947300
H 6.75020700 1.38151500 -0.54040100
H 6.36333500 3.78693300 -0.39682500
H 4.12553400 4.65524600 -0.17349000
O 1.72148500 4.38898300 0.00474100
N -0.34648900 2.90506900 0.07391200
C -1.11331200 2.83865900 -1.14825900
H -2.13133700 3.24621200 -0.95418000
H -0.63207500 3.46997300 -1.93036200
H -1.22275400 1.80302200 -1.54093400
H -0.29744100 3.86174300 0.42636200
H -0.97042500 0.43668100 0.32386800
H -0.15673100 -0.49950500 -0.98353800
H 0.22645800 -0.78262800 0.75989600

3-Ethylamine-menadione ground (**1g**)
C 0.00000000 0.00000000 0.00000000
C -1.14667100 -0.98490300 0.14460400
C -0.99773300 -2.30422400 -0.09605100
C -2.06507800 -3.13835200 -0.01116600
C -3.26861800 -2.62978000 0.35062300
C -4.32824400 -3.45408300 0.44914100
C -5.52586100 -2.96868400 0.81127900
C -5.66679400 -1.66168700 1.07543100
C -4.61131000 -0.83840100 0.97690800
C -3.40793900 -1.32199200 0.61462000
C -2.35187400 -0.47528800 0.51376100
O -2.46313000 0.71050700 0.73839400
H -4.77386500 0.23029500 1.20119500
H -6.65308700 -1.26458400 1.37293600
H -6.39583600 -3.64363300 0.89299100
H -4.25324900 -4.53594400 0.24204800
O -1.89250100 -4.31247000 -0.25697200
N 0.21152300 -2.88968800 -0.44610700
C 1.18232700 -2.95044600 0.62652800
C 0.78359700 -3.96959600 1.70283700
H 1.58268200 -4.06804600 2.47378500
H -0.15031600 -3.66452800 2.22890800

H 0.61559300 -4.97809200 1.25890600
H 2.15282000 -3.26667900 0.17611200
H 1.35492700 -1.95677800 1.10008700
H 0.07294300 -3.80778100 -0.86981100
H 0.88483400 -0.41204000 -0.53354300
H -0.30779300 0.88098700 -0.60856900
H 0.33169300 0.36275500 0.99989000

3-Ethylamine-menadione Radical Anion (**1g**)
C 0.00000000 0.00000000 0.00000000
C 0.96897100 -1.16623100 -0.08245700
C 0.58055400 -2.44157800 0.12569300
C 1.49312500 -3.44594300 0.09989900
C 2.78811400 -3.14957600 -0.17068700
C 3.69572800 -4.14287600 -0.21044500
C 4.98096400 -3.86832200 -0.48216500
C 5.36105200 -2.60363500 -0.71450200
C 4.45733500 -1.61204800 -0.67447500
C 3.16674200 -1.88383500 -0.40296700
C 2.26628700 -0.86908800 -0.36026200
O 2.59375600 0.28109800 -0.55719800
H 4.81520800 -0.58602200 -0.86950700
H 6.41900100 -2.38013700 -0.93758900
H 5.72650300 -4.68204300 -0.51560100
H 3.42286700 -5.19680300 -0.02682200
O 1.10590200 -4.57420900 0.31397500
N -0.73235800 -2.81266300 0.38291700
C -1.62191200 -2.70114800 -0.75416500
C -1.32877900 -3.76860200 -1.81751300
H -2.07707800 -3.72504900 -2.64258300
H -0.32204900 -3.62574300 -2.27368900
H -1.36683900 -4.79300200 -1.37988300
H -2.66121200 -2.84838700 -0.37613300
H -1.58882900 -1.69043200 -1.22190800
H -0.78249200 -3.74286200 0.79985900
H -0.97727600 -0.25669500 0.46520400
H 0.41037000 0.81236400 0.64256600
H -0.19419700 0.41884800 -1.01402400

3-Isobutylamine-menadione ground (**1h**)
C 0.00000000 0.00000000 0.00000000
C -1.16990100 -0.71403100 0.65374000
C -1.07521500 -1.98099200 1.10758300
C -2.15802300 -2.59983400 1.64182500
C -3.32524600 -1.91586900 1.73144700
C -4.40216200 -2.52069200 2.26658400
C -5.56481200 -1.85784300 2.36615400
C -5.65346700 -0.59208200 1.93269400
C -4.58044700 0.01218600 1.39885600
C -3.41200800 -0.64937800 1.29755100
C -2.33806000 -0.02536300 0.74995000

O -2.40119600 1.11566800 0.34623000
H -4.69971700 1.05381200 1.05310700
H -6.61126300 -0.04901300 2.01494400
H -6.44923700 -2.35254500 2.80447200
H -4.36989400 -3.56067300 2.63565300
O -2.02949400 -3.74308700 2.02279100
N 0.09569500 -2.72442900 1.05828000
C 1.09471800 -2.31044200 2.02016900
C 2.40752400 -3.08970400 1.81363700
C 3.00785500 -2.81498100 0.42448700
H 3.95393800 -3.38444800 0.27569900
H 2.31177700 -3.11607300 -0.39145200
H 3.23831600 -1.73299900 0.29379300
C 3.42504300 -2.73298200 2.91048400
H 4.36754400 -3.31536000 2.79094200
H 3.68708900 -1.65062000 2.87993400
H 3.02166700 -2.95800000 3.92457400
H 2.18430700 -4.18320200 1.88881200
H 1.30321800 -1.21762000 1.96303200
H 0.69953000 -2.51961100 3.04252900
H -0.09249800 -3.72494800 1.12969100
H 0.85094100 -0.66764000 -0.25893700
H -0.30592600 0.45679900 -0.96895200
H 0.38438600 0.80764700 0.66424900

3-Isobutylamine-menadione Radical Anion (**1h**)
C 0.00000000 0.00000000 0.00000000
C 0.96984400 -1.16419400 -0.09969800
C 0.57626900 -2.44338400 0.07029800
C 1.48321200 -3.45129300 0.02104200
C 2.78258600 -3.15162400 -0.22406200
C 3.68722800 -4.14675100 -0.28128000
C 4.97716700 -3.86891400 -0.52602700
C 5.36486000 -2.59919100 -0.71416400
C 4.46387900 -1.60596400 -0.65734200
C 3.16861400 -1.88067600 -0.41238900
C 2.27135100 -0.86383200 -0.35263700
O 2.60439600 0.29042600 -0.51294300
H 4.82798600 -0.57590500 -0.81594900
H 6.42679500 -2.37296800 -0.91461300
H 5.72058500 -4.68389300 -0.57291500
H 3.40821100 -5.20456000 -0.13294800
O 1.08590600 -4.58305000 0.19412100
N -0.74060200 -2.81230000 0.30675300
C -1.59693000 -2.70845900 -0.85539200
C -3.06214100 -3.00375700 -0.48213600
C -3.59549200 -1.98359100 0.53786600
H -4.65332000 -2.20316500 0.81042800
H -3.00376400 -1.99743900 1.48158400
H -3.55809300 -0.94869700 0.12741000
C -3.94858900 -3.00921100 -1.73905600

H -5.00570300 -3.25447500 -1.48584300
H -3.94046000 -2.01506200 -2.24176000
H -3.59900100 -3.76761500 -2.47684700
H -3.11006800 -4.02058600 -0.01848000
H -1.53466000 -1.70384200 -1.33274400
H -1.25133300 -3.45554800 -1.60876100
H -0.79998400 -3.74058800 0.72664700
H -0.99077100 -0.27086100 0.42649600
H 0.39527800 0.78778500 0.68137700
H -0.16836600 0.45427800 -1.00327400

3-Pyrrolidine-menadione ground (**1i**)
C 0.00000000 0.00000000 0.00000000
C -1.33494800 -0.61011900 0.40443500
C -1.44764000 -1.88755500 0.83656400
C -2.67446100 -2.35682600 1.19601600
C -3.75607600 -1.54352200 1.08282400
C -4.97315000 -2.00945700 1.42430800
C -6.05481300 -1.22214200 1.31803700
C -5.92690500 0.03542600 0.87168900
C -4.71645000 0.50493600 0.53274000
C -3.63117700 -0.28507000 0.63765500
C -2.41559500 0.20686100 0.29419600
O -2.27787100 1.34386300 -0.10292800
H -4.65534400 1.54656200 0.17202800
H -6.81747800 0.68232900 0.78497300
H -7.05114700 -1.60763900 1.59710500
H -5.13049500 -3.03693000 1.79619000
O -2.80285100 -3.47944700 1.63049600
N -0.29616400 -2.67201500 0.92912700
C -0.30121800 -4.03712600 0.45132600
C 1.13329800 -4.47616900 0.78211300
C 1.40149800 -3.79029800 2.13530300
C 0.33462800 -2.68177600 2.23153900
H -0.41282200 -2.93950000 3.02036300
H 0.77821000 -1.68646300 2.47343500
H 1.28260100 -4.51680500 2.97455500
H 2.43779800 -3.37774300 2.17698800
H 1.25152100 -5.58522700 0.82377300
H 1.83110200 -4.07593200 0.00719400
H -0.99678700 -4.69676000 1.01798800
H -0.52945100 -4.09300700 -0.63981500
H 0.87922000 -0.67696200 0.07069800
H -0.03424900 0.34015200 -1.06044700
H 0.23537200 0.87608100 0.64683000

3-Pyrrolidine-menadione Radical Anion (**1i**)
C 0.00000000 0.00000000 0.00000000
C -0.79844300 -1.29541000 0.04798600
C -0.20730800 -2.51270000 0.04157000
C -0.98888400 -3.62620200 0.10291600

C -2.33946300 -3.48870700 0.13752600
C -3.11576100 -4.58878700 0.18400700
C -4.45253400 -4.47431200 0.21696000
C -5.02153400 -3.26057100 0.20532400
C -4.25253900 -2.16181400 0.16165700
C -2.91140100 -2.27616800 0.12696100
C -2.14985800 -1.15544200 0.08664100
O -2.64494200 -0.04894500 0.08452600
H -4.76150600 -1.18214600 0.15574900
H -6.12141200 -3.16762900 0.23277500
H -5.08598600 -5.37796500 0.25327700
H -2.69551900 -5.60958900 0.19470900
O -0.49038800 -4.72856300 0.14578300
N 1.18667700 -2.58504100 0.00398300
C 1.84654000 -3.52337100 -0.87690600
C 3.32333100 -3.25329700 -0.55107700
C 3.30252900 -3.00719800 0.96960600
C 1.82888600 -2.69417900 1.29624000
H 1.38101600 -3.53442900 1.87996800
H 1.71599200 -1.75242000 1.88468700
H 3.63638800 -3.91931000 1.51995300
H 3.98035600 -2.16542800 1.24858800
H 3.99629900 -4.09262700 -0.84833300
H 3.65670400 -2.32890400 -1.08209400
H 1.63247000 -4.58644100 -0.62373400
H 1.59812300 -3.33078500 -1.94788700
H 1.10438100 -0.11752800 -0.05322500
H -0.28330500 0.59738800 -0.89682200
H -0.19942700 0.60989200 0.91080600

3-Phenethylamine-menadione ground (**1j**)
C 0.00000000 0.00000000 0.00000000
C -0.80495400 -1.09855700 0.67195000
C -0.33331700 -2.35555300 0.80459600
C -1.10280900 -3.31977400 1.36929900
C -2.33939200 -2.99142700 1.81793300
C -3.10566500 -3.94035900 2.38742800
C -4.33059200 -3.63220800 2.84091900
C -4.79121100 -2.37808500 2.72737700
C -4.02848300 -1.43102700 2.15919500
C -2.79885500 -1.73617100 1.70307900
C -2.04255700 -0.76749100 1.12659400
O -2.44537400 0.36942000 1.00700800
H -4.44865900 -0.41283500 2.08488800
H -5.79922900 -2.12690600 3.10147900
H -4.96028800 -4.40933400 3.30853300
H -2.76244400 -4.98349200 2.50109100
O -0.64242200 -4.43768600 1.45314100
N 0.93069300 -2.74507200 0.38363600
C 2.00322200 -2.26381800 1.23004500
C 3.36137100 -2.61041000 0.59909300

C 4.59160200 -2.33794100 1.44518100
C 4.60655200 -1.43977100 2.44769300
C 5.71451300 -1.22638900 3.17524300
C 6.83683000 -1.91116000 2.91099900
C 6.84167600 -2.80777500 1.91397200
C 5.72883100 -3.01369300 1.19273700
H 5.75716900 -3.75390800 0.37489300
H 7.76159800 -3.37424300 1.68678400
H 7.74936100 -1.73648100 3.50713800
H 5.70464200 -0.48413800 3.99252800
H 3.70834000 -0.85248000 2.69490700
H 3.34466500 -3.69508100 0.33731000
H 3.47313100 -2.04508600 -0.35620100
H 1.92376200 -1.16461500 1.39145000
H 1.91727100 -2.76833200 2.22199000
H 0.98632700 -3.75620600 0.25632500
H 0.32649800 0.75699000 0.74944600
H 0.90375800 -0.36236500 -0.53809700
H -0.60758000 0.51508900 -0.77887900

3-Phenethylamine-menadione Radical Anion (**1j**)
C 0.00000000 0.00000000 0.00000000
C -0.80055800 -1.24416400 0.34272200
C -0.33018200 -2.48713400 0.11067300
C -1.09605800 -3.57213400 0.38805300
C -2.32752900 -3.38470900 0.92337700
C -3.09010800 -4.45639000 1.20963500
C -4.30991800 -4.28970200 1.74380500
C -4.76909500 -3.05486800 1.99326100
C -4.01005200 -1.98535900 1.70776900
C -2.78556100 -2.14841800 1.17188300
C -2.03298500 -1.05582700 0.88458800
O -2.43465900 0.06830800 1.09460500
H -4.42888100 -0.98790800 1.92790400
H -5.77289800 -2.92026200 2.43304000
H -4.93657600 -5.16770000 1.97943500
H -2.74789600 -5.48893400 1.02098500
O -0.63712800 -4.66791100 0.14853300
N 0.92886200 -2.74103100 -0.41563200
C 2.01058600 -2.51722500 0.52144700
C 3.36179800 -2.67061900 -0.19513600
C 4.60079100 -2.64689600 0.68116500
C 4.62735600 -2.06801900 1.89613800
C 5.74280700 -2.06764300 2.64327000
C 6.86110300 -2.64912700 2.18542600
C 6.85439000 -3.22804300 0.97595700
C 5.73413200 -3.22304400 0.23703700
H 5.75297800 -3.70244400 -0.75664800
H 7.77090100 -3.70665100 0.58904900
H 7.77977000 -2.64897000 2.79751800
H 5.74242600 -1.58611600 3.63681000

H 3.73279000 -1.57506600 2.30791600
H 3.34034500 -3.63739300 -0.75210000
H 3.46535000 -1.85866500 -0.95321000
H 1.93492500 -1.50827100 0.98717200
H 1.93332300 -3.28115700 1.33157900
H 0.98121000 -3.67513200 -0.82356700
H 0.33533700 0.51508200 0.92927100
H 0.89772000 -0.19509700 -0.62743200
H -0.61412800 0.71339700 -0.59584600

3-Cadaverine-menadione ground (**1k**)
C 0.00000000 0.00000000 0.00000000
C -0.97444400 -1.05892700 0.48480200
C -0.64683600 -2.36662600 0.53542800
C -1.56252100 -3.28719200 0.92914300
C -2.79930800 -2.86702800 1.29231900
C -3.71076100 -3.77343600 1.69194400
C -4.93859800 -3.37493600 2.05903500
C -5.25748900 -2.07289900 2.02812700
C -4.34986000 -1.16808100 1.62941900
C -3.11673500 -1.56386700 1.26067800
C -2.21234800 -0.63636200 0.85519000
O -2.48521100 0.54382000 0.81322200
H -4.65657800 -0.10760600 1.61777500
H -6.26831200 -1.74741600 2.33041100
H -5.68736500 -4.11697500 2.38730400
H -3.48703700 -4.85380500 1.73267300
O -1.22791100 -4.45193100 0.94457400
N 0.60773700 -2.85390100 0.19622900
C 1.63094900 -2.56702100 1.17914000
C 2.99642800 -3.05824800 0.67344700
C 4.11606300 -2.79927700 1.69440500
C 5.47861400 -3.30367100 1.19249800
C 6.59714900 -3.03899300 2.21050000
N 7.86375100 -3.52895800 1.72709400
H 8.59155300 -3.41210600 2.43242600
H 7.81950400 -4.53068400 1.53930600
H 6.69283600 -1.94664300 2.41474500
H 6.37274200 -3.55405500 3.17341500
H 5.41793700 -4.39930300 0.98778900
H 5.73062100 -2.79877700 0.22950100
H 4.18047000 -1.70490900 1.90327800
H 3.86524500 -3.30806600 2.65541000
H 2.93743000 -4.15135100 0.45510000
H 3.23978900 -2.54049600 -0.28508900
H 1.69170500 -1.47895400 1.41200700
H 1.37496600 -3.10032700 2.12539300
H 0.57510200 -3.85043500 -0.02154000
H 0.33099600 0.64215000 0.84816200
H 0.90730400 -0.41060900 -0.49579700
H -0.47406700 0.65079800 -0.77005400

3-Cadaverine-menadione Radical Anion (**1k**)

C	0.00000000	0.00000000	0.00000000
C	-0.97444400	-1.05892700	0.48480200
C	-0.64683600	-2.36662600	0.53542800
C	-1.56252100	-3.28719200	0.92914300
C	-2.79930800	-2.86702800	1.29231900
C	-3.71076100	-3.77343600	1.69194400
C	-4.93859800	-3.37493600	2.05903500
C	-5.25748900	-2.07289900	2.02812700
C	-4.34986000	-1.16808100	1.62941900
C	-3.11673500	-1.56386700	1.26067800
C	-2.21234800	-0.63636200	0.85519000
O	-2.48521100	0.54382000	0.81322200
H	-4.65657800	-0.10760600	1.61777500
H	-6.26831200	-1.74741600	2.33041100
H	-5.68736500	-4.11697500	2.38730400
H	-3.48703700	-4.85380500	1.73267300
O	-1.22791100	-4.45193100	0.94457400
N	0.60773700	-2.85390100	0.19622900
C	1.63094900	-2.56702100	1.17914000
C	2.99642800	-3.05824800	0.67344700
C	4.11606300	-2.79927700	1.69440500
C	5.47861400	-3.30367100	1.19249800
C	6.59714900	-3.03899300	2.21050000
N	7.86375100	-3.52895800	1.72709400
H	8.59155300	-3.41210600	2.43242600
H	7.81950400	-4.53068400	1.53930600
H	6.69283600	-1.94664300	2.41474500
H	6.37274200	-3.55405500	3.17341500
H	5.41793700	-4.39930300	0.98778900
H	5.73062100	-2.79877700	0.22950100
H	4.18047000	-1.70490900	1.90327800
H	3.86524500	-3.30806600	2.65541000
H	2.93743000	-4.15135100	0.45510000
H	3.23978900	-2.54049600	-0.28508900
H	1.69170500	-1.47895400	1.41200700
H	1.37496600	-3.10032700	2.12539300
H	0.57510200	-3.85043500	-0.02154000
H	0.33099600	0.64215000	0.84816200
H	0.90730400	-0.41060900	-0.49579700
H	-0.47406700	0.65079800	-0.77005400

3-Histamine-menadione ground (**1l**)

C	0.00000000	0.00000000	0.00000000
C	-1.15804700	-0.90846500	0.37110200
C	-1.03666400	-2.25164300	0.39784100
C	-2.11069900	-3.03121200	0.68162500
C	-3.29536700	-2.43600400	0.96511200
C	-4.36293200	-3.20217700	1.25697800
C	-5.54219500	-2.62982900	1.54468000
C	-5.65678100	-1.29390400	1.54198500

C -4.59318500 -0.52882900 1.25071000
C -3.40811200 -1.09924700 0.96184400
C -2.34394400 -0.31112600 0.66328100
O -2.43210100 0.89760800 0.64721500
H -4.73409100 0.56607500 1.25802900
H -6.62815400 -0.82535800 1.77882000
H -6.41877500 -3.25674600 1.78467300
H -4.30924400 -4.30474900 1.27158400
O -1.95946200 -4.23391400 0.67347100
N 0.14892100 -2.92610900 0.14336800
C 1.14562900 -2.79337100 1.18464600
C 2.30808000 -3.75447200 0.89387300
C 1.79563900 -5.16210800 0.81735400
C 1.41673200 -5.83484000 -0.27701500
N 0.96195300 -7.07933600 0.07323700
C 1.08039100 -7.01751200 1.44526700
N 1.56208900 -5.90436700 1.81681700
H 0.44715200 -7.62510700 2.11385600
H 0.14327700 -7.48395100 -0.37973100
H 1.42779100 -5.46347500 -1.31510200
H 2.79287000 -3.47988100 -0.07253100
H 3.08747800 -3.68462900 1.68901300
H 1.53263100 -1.75184400 1.26520900
H 0.69076900 -3.06382100 2.16773700
H -0.02253300 -3.90982600 -0.06989900
H 0.39765000 0.51303600 0.90547800
H 0.83921900 -0.52749200 -0.50475100
H -0.31819300 0.77666700 -0.73258500

3-Histamine-menadione Radical Anion (**1l**)
C 0.00000000 0.00000000 0.00000000
C -0.29011800 -1.48523500 0.11835000
C 0.68257300 -2.41577200 0.03145700
C 0.37458000 -3.73629400 0.08778300
C -0.91984600 -4.10005400 0.26160500
C -1.23209800 -5.40771700 0.32808500
C -2.50760900 -5.78577900 0.50459500
C -3.47139700 -4.86021900 0.61542400
C -3.16143600 -3.55602900 0.54887300
C -1.88277200 -3.17259400 0.37220300
C -1.58722400 -1.84961300 0.30077700
O -2.44701300 -0.99995900 0.38975900
H -3.98710900 -2.82925600 0.64262400
H -4.52013700 -5.17378000 0.76038900
H -2.76545200 -6.85792200 0.55973000
H -0.46794200 -6.19994100 0.24318800
O 1.27888200 -4.53688200 -0.01579900
N 2.02857600 -2.11542200 -0.12130900
C 2.64931800 -1.54476300 1.05526800
C 4.16768800 -1.46769000 0.83579700
C 4.70976600 -2.83750200 0.55356000

C	4.91103600	-3.40560600	-0.64248800
N	5.37196700	-4.68604100	-0.48031600
C	5.36463200	-4.78771600	0.89442900
N	4.98085900	-3.70607800	1.43475400
H	5.25983500	-5.75880500	1.40743800
H	5.03990000	-5.44150000	-1.07876100
H	4.71767600	-2.95123900	-1.62829800
H	4.39162500	-0.79248400	-0.02345100
H	4.67617600	-1.04371700	1.73372400
H	2.25315600	-0.53008800	1.28878300
H	2.44451400	-2.20033500	1.93548900
H	2.55443700	-2.92646100	-0.45034300
H	-0.07455400	0.49161900	0.99698200
H	0.99888700	0.22819500	-0.43296900
H	-0.71993800	0.49558800	-0.69081200

3-Tyramine-menadione ground (**1m**)

C	0.00000000	0.00000000	0.00000000
C	-1.16482000	-0.93150200	0.28621100
C	-0.99723600	-2.26056200	0.44584400
C	-2.05524200	-3.05358000	0.74919700
C	-3.28320400	-2.49035800	0.86360400
C	-4.33973700	-3.27139300	1.15617400
C	-5.56330600	-2.73173800	1.26890100
C	-5.73343900	-1.41387100	1.08952000
C	-4.68068900	-0.63380500	0.79832800
C	-3.45145500	-1.17143100	0.68402400
C	-2.39686100	-0.36729900	0.39421000
O	-2.52950700	0.82718500	0.23721300
H	-4.86677600	0.44568400	0.66098200
H	-6.74102200	-0.97212700	1.18293000
H	-6.43108200	-3.37092900	1.50872200
H	-4.24157200	-4.36028700	1.30975400
O	-1.85226600	-4.23893400	0.89898500
N	0.23171900	-2.89248600	0.32232600
C	0.69884600	-3.00676500	-1.04273500
C	2.14924200	-3.51358000	-1.06207600
C	3.06152700	-2.52898900	-0.36232300
C	3.54965900	-1.46007500	-1.01480700
C	4.34449300	-0.57703800	-0.39156500
C	4.67991200	-0.72853100	0.90115700
C	4.18822000	-1.79877700	1.54869800
C	3.39286600	-2.68512200	0.93091100
H	3.00266900	-3.54647500	1.49935300
H	4.44159600	-1.94872100	2.61220700
O	5.48291700	0.16666400	1.53671800
H	5.58654600	-0.13048900	2.45551900
H	4.72860100	0.29455900	-0.94875100
H	3.28883700	-1.29821600	-2.07452500
H	2.20797500	-4.51565500	-0.57514200
H	2.48923800	-3.66084400	-2.11462700

H 0.04456800 -3.73400100 -1.58030500
H 0.63521400 -2.03844000 -1.59153700
H 0.24050400 -3.79795100 0.79279000
H 0.99903600 -0.46588100 0.14605600
H -0.05239200 0.38079400 -1.04560100
H -0.00871900 0.87176600 0.69348200

3-Tyramine-menadione Radical Anion (**1m**)
C 0.00000000 0.00000000 0.00000000
C 1.23562700 0.83371700 0.29079500
C 1.16165500 2.15138200 0.57053700
C 2.28305500 2.85210400 0.87340200
C 3.47742500 2.21053800 0.86496000
C 4.59599400 2.90036500 1.15619200
C 5.78738400 2.28263900 1.14831100
C 5.86349400 0.97767400 0.84970300
C 4.74883300 0.28847000 0.55969000
C 3.55158200 0.90464500 0.56618100
C 2.43364000 0.19149500 0.27564700
O 2.48145700 -0.99052600 0.01187800
H 4.85827600 -0.78400500 0.32176500
H 6.84477800 0.47157500 0.84401200
H 6.70594000 2.84697200 1.38684400
H 4.57566500 3.97566500 1.40565400
O 2.16417700 4.02976600 1.13355500
N -0.02987200 2.86212200 0.57569900
C -0.56476100 3.12154200 -0.74386600
C -1.97899700 3.71170200 -0.63192000
C -2.91097800 2.72484200 0.03804600
C -3.50160000 1.74688400 -0.67001300
C -4.31442600 0.86082800 -0.07489400
C -4.56646300 0.91873200 1.24408700
C -3.97248700 1.89823700 1.94712200
C -3.15862800 2.78707800 1.35767300
H -2.68343500 3.57161500 1.97086300
H -4.15574600 1.96973200 3.03295700
O -5.38750800 0.01999100 1.85094700
H -5.42037400 0.24173900 2.79592400
H -4.78356700 0.06477100 -0.67811500
H -3.31146300 1.66277600 -1.75352700
H -1.94680400 4.66940700 -0.06054200
H -2.36757400 3.96938000 -1.64570400
H 0.10297100 3.85339200 -1.25812500
H -0.59341300 2.20249100 -1.37427100
H 0.04505500 3.72226900 1.11958600
H -0.95779200 0.50880600 0.24535700
H -0.03059700 -0.29093400 -1.07492000
H -0.00749400 -0.92773100 0.61662600

3-Tryptamine-menadione ground (**1n**)
C 0.00000000 0.00000000 0.00000000

C -1.24240700 0.33154900 -0.81262700
C -1.81664400 -0.55720200 -1.65327000
C -2.95032700 -0.21487700 -2.32070500
C -3.45094500 1.03723800 -2.16782700
C -4.55771700 1.39234700 -2.84780200
C -5.06537200 2.62743900 -2.71399600
C -4.47071400 3.51206300 -1.90091500
C -3.36889300 3.16096000 -1.22007800
C -2.85781700 1.92252900 -1.35359100
C -1.74752800 1.58306700 -0.65268300
O -1.20621900 2.36328000 0.10053700
H -2.91263300 3.91939100 -0.56029300
H -4.88973300 4.52782700 -1.79234900
H -5.97198500 2.91777000 -3.27329300
H -5.07891900 0.69852300 -3.53019100
O -3.50126000 -1.03129000 -3.02557700
N -1.27128100 -1.82748100 -1.80655000
C -0.94844600 -2.25972500 -3.15058400
C -0.32137400 -1.19273400 -4.06330200
C 0.86476700 -0.51013600 -3.44601600
C 1.03671600 0.79833700 -3.19957700
C 2.24444200 0.93074700 -2.63305400
N 2.76719700 -0.22109400 -2.54747000
C 1.98749300 -1.10659100 -3.01528900
H 2.26324400 -2.17330600 -3.02596500
H 3.71506900 -0.40998400 -2.13961700
C 2.71713900 2.12316200 -2.25431000
C 1.93528300 3.19769700 -2.45649900
C 0.72173400 3.07072200 -3.02379900
C 0.25816700 1.86813300 -3.40641800
H -0.73967600 1.77642700 -3.86630700
H 0.09424600 3.96614700 -3.17559800
H 2.29304700 4.19571400 -2.14960300
H 3.71169600 2.21644800 -1.78604800
H 0.01815100 -1.67060300 -5.01226800
H -1.09030100 -0.44516200 -4.36180000
H -0.23609300 -3.11215300 -3.04739100
H -1.85234300 -2.69690000 -3.63494600
H -1.83442100 -2.52919700 -1.32700900
H 0.80441100 0.74898100 -0.17909800
H -0.24498800 0.00663400 1.08668700
H 0.45607200 -0.99147400 -0.21335700

3-Tryptamine-menadione Radical Anion (**1n**)
C 0.00000000 0.00000000 0.00000000
C 1.17881900 0.18822800 -0.94277000
C 1.41798100 1.35607800 -1.57935100
C 2.51207700 1.47438100 -2.37738100
C 3.31294200 0.39407700 -2.55997500
C 4.38267200 0.49603900 -3.37157400
C 5.18394500 -0.56228800 -3.56899800

C 4.92081100 -1.72562700 -2.95688500
C 3.85691500 -1.83025100 -2.14571300
C 3.05158100 -0.76973900 -1.94699600
C 1.98472800 -0.89186600 -1.11865000
O 1.74076600 -1.93010200 -0.54277200
H 3.67844500 -2.80558200 -1.66034300
H 5.58107400 -2.59530100 -3.12077500
H 6.05980600 -0.47774700 -4.23591000
H 4.63648200 1.43243400 -3.89830100
O 2.76243600 2.53873000 -2.89832500
N 0.56960600 2.44187400 -1.39005800
C -0.00643600 3.05488900 -2.56898600
C -0.46430200 2.08474900 -3.67077100
C -1.36920700 0.99937500 -3.16379600
C -1.19152700 -0.32979400 -3.22814400
C -2.25524200 -0.89222400 -2.63715800
N -3.02475600 0.03639400 -2.24619500
C -2.54210500 1.17420300 -2.53487500
H -3.06480900 2.10804800 -2.27267900
H -3.93422800 -0.12160200 -1.74781300
C -2.37923600 -2.21934800 -2.52711400
C -1.39264900 -2.98236800 -3.02836300
C -0.32211400 -2.42336300 -3.62176000
C -0.20982100 -1.08821300 -3.73256200
H 0.67426300 -0.64193900 -4.21715800
H 0.47886400 -3.06908500 -4.02181900
H -1.46115900 -4.08106000 -2.94836100
H -3.25877000 -2.66806200 -2.03498400
H -1.01992400 2.65089000 -4.45495300
H 0.41986700 1.64677900 -4.18617400
H -0.88469800 3.64851500 -2.22114300
H 0.70136900 3.80693400 -2.98874600
H 1.00145000 3.14536100 -0.79163600
H -0.61686800 -0.87592500 -0.30390000
H 0.36809400 -0.17728500 1.03635400
H -0.70271800 0.85981000 0.05879800

3-β-alanine-ethyl ester-menadione ground (**1o**)
C 0.00000000 0.00000000 0.00000000
C -1.09805300 -0.98300500 -0.36623600
C -0.87698400 -2.31202900 -0.43308200
C -1.87139800 -3.15459100 -0.80924100
C -3.09294200 -2.63941900 -1.09281200
C -4.08722500 -3.46956700 -1.45893500
C -5.30376900 -2.97757400 -1.74018600
C -5.52878600 -1.65840500 -1.65583700
C -4.53814000 -0.82952600 -1.29099000
C -3.31622200 -1.31919700 -1.00765800
C -2.32415400 -0.46638500 -0.64513500
O -2.50608400 0.72966500 -0.57150900
H -4.76791200 0.24890100 -1.23667200

H -6.53034200 -1.25593500 -1.88801200
H -6.12052000 -3.65698600 -2.04067400
H -3.94248900 -4.56111900 -1.53885200
O -1.61924300 -4.33846200 -0.86855400
N 0.34663600 -2.89580100 -0.13831400
C 0.65516600 -2.93372100 1.27497400
C 2.11426600 -3.36444500 1.48699400
C 3.07836200 -2.34787400 0.89842300
O 3.49517500 -1.37949300 1.49291000
O 3.37585300 -2.65570500 -0.39636200
C 4.18807300 -1.73020800 -1.09254800
C 4.32411700 -2.19455300 -2.54803400
H 4.96074500 -1.49077900 -3.13265300
H 4.79136900 -3.20487800 -2.60394900
H 3.32808700 -2.24796300 -3.04561100
H 3.70836200 -0.72056600 -1.06219700
H 5.19411700 -1.68535900 -0.60687400
H 2.32563000 -3.45174700 2.57898300
H 2.29370900 -4.37184700 1.04186300
H 0.48771900 -1.94740300 1.76738300
H -0.02555500 -3.66971200 1.76565100
H 0.43319000 -3.82007500 -0.56232900
H 1.02064500 -0.44165000 0.00945800
H 0.05513700 0.82727500 -0.74408700
H -0.19195300 0.44123400 1.00481000

3-β-alanine-ethyl ester-menadione Radical Anion (**1o**)
C 0.00000000 0.00000000 0.00000000
C 1.10258400 1.03646300 -0.12728100
C 0.84239300 2.35979300 -0.15456000
C 1.84994300 3.25362700 -0.31632700
C 3.12067400 2.79408300 -0.42608200
C 4.12697900 3.67490900 -0.57868000
C 5.39148500 3.23819500 -0.68566200
C 5.65236900 1.92373600 -0.63994800
C 4.64980500 1.04436400 -0.48794500
C 3.37975800 1.47847100 -0.37991600
C 2.37711700 0.57532400 -0.23177500
O 2.59232800 -0.61694700 -0.19672300
H 4.91061700 -0.02792500 -0.45754900
H 6.69340900 1.56680900 -0.72849800
H 6.21816900 3.95922600 -0.81089500
H 3.95361900 4.76444200 -0.61974200
O 1.56366200 4.43074300 -0.34937100
N -0.43491100 2.88702000 -0.03026600
C -0.96739700 2.82826800 1.31398700
C -2.45758000 3.20080500 1.30992600
C -3.27356400 2.19385600 0.51649200
O -3.74146800 1.17846800 0.98013200
O -3.37153100 2.57191800 -0.79010700
C -4.02409000 1.66705900 -1.65977300

C -3.94328200 2.21606400 -3.08967800
H -4.44938100 1.53082000 -3.80848100
H -4.43534200 3.21333700 -3.16405000
H -2.88326200 2.33015900 -3.41502600
H -3.51566900 0.67242300 -1.60852300
H -5.09241700 1.56193800 -1.34729900
H -2.84466500 3.21409200 2.35621600
H -2.60324300 4.22788800 0.89872800
H -0.84198700 1.81893800 1.77099200
H -0.40402500 3.55278500 1.94921600
H -0.48904100 3.83267400 -0.41022900
H -1.02570200 0.40918900 -0.13175900
H 0.09788900 -0.78104100 -0.78834200
H 0.04570000 -0.49643300 0.99631400

Naphthoquinone ground (**2**)
C 0.00000000 0.00000000 0.00000000
C 0.03794000 -1.34016300 0.00000000
C 1.21858800 -1.97816000 -0.00000000
C 2.36814000 -1.27712800 -0.00000000
C 2.33003400 0.06889800 0.00000000
C 1.14266200 0.70377100 0.00000000
H 1.06584000 1.80511800 0.00000000
C 3.46805100 0.81257600 -0.00000000
C 4.63864000 0.13031000 0.00000000
C 4.67652200 -1.20783600 0.00000000
C 3.54640600 -1.95523200 0.00000000
O 3.62960300 -3.16417100 0.00000000
H 5.65961400 -1.71152300 -0.00000000
H 5.59166300 0.68880600 0.00000000
O 3.48272100 2.02428400 0.00000000
H 1.20419800 -3.08208800 0.00000000
H -0.90362300 -1.91683400 0.00000000
H -0.97268100 0.52247700 0.00000000

Naphthoquinone (**2**)
C 0.00000000 0.00000000 0.00000000
C -0.00000300 -1.34070000 0.00000000
C 1.16211600 -2.01185500 0.00000000
C 2.33104700 -1.34363700 0.00000000
C 2.33105100 0.00292800 0.00000000
C 1.16212200 0.67115000 0.00000000
H 1.11650000 1.77423000 0.00000000
C 3.48965900 0.71410100 0.00000000
C 4.64047000 -0.00102100 0.00000000
C 4.64046600 -1.33970300 0.00000000
C 3.48965000 -2.05481600 0.00000000
O 3.53860000 -3.26562500 0.00000000
H 5.60890900 -1.87101000 0.00000000
H 5.60891700 0.53028000 0.00000000
O 3.53861500 1.92490800 0.00000000

H 1.11649000 -3.11493400 0.00000000
H -0.95750900 -1.89049300 0.00000000
H -0.95750400 0.54979500 0.00000000

2-[(*R*)-amino-2-propanol]-naphthoquinone ground (**2c**)
C 0.00000000 0.00000000 0.00000000
N -1.20024600 -0.64903400 -0.47602700
C -2.39875300 0.04566100 -0.37685900
C -3.53175500 -0.68850600 -0.23537100
C -4.72319000 -0.04261900 -0.14230200
C -5.84925700 -0.76758900 -0.00282600
C -7.03954100 -0.15468900 0.09192600
C -7.11090800 1.18355200 0.04844700
C -5.99158900 1.91092200 -0.08997500
C -4.79668200 1.29855700 -0.18564700
C -3.67704700 2.05289800 -0.32451600
C -2.50173700 1.38473200 -0.41633600
H -1.59687000 2.00296200 -0.53655300
O -3.68788100 3.26397800 -0.36734100
H -6.09275100 3.00982500 -0.12106000
H -8.09141700 1.68497100 0.12691200
H -7.96190200 -0.75073000 0.20611500
H -5.83699400 -1.87078400 0.03788500
O -3.42826900 -1.89516600 -0.18587000
H -1.28489600 -1.63120100 -0.21257900
C 1.14120700 -1.02512600 0.11290300
C 2.46437900 -0.37164600 0.53481600
H 3.27721600 -1.13023900 0.61152000
H 2.78634400 0.39982500 -0.20177100
H 2.36610400 0.12263400 1.52860800
H 0.86652100 -1.81983400 0.85096200
O 1.31379600 -1.66273200 -1.13253500
H 1.97214500 -2.32723300 -1.02382500
H 0.31003800 0.80942600 -0.70270300
H -0.18266400 0.43601000 1.01102000

2-[(*R*)-amino-2-propanol]-naphthoquinone Radical Anion (**2c**)
C 0.00000000 0.00000000 0.00000000
N -1.00126100 0.91932500 0.49066900
C -2.33326900 0.53441500 0.41034200
C -3.25753800 1.52034600 0.28235900
C -4.57082000 1.18109300 0.20788100
C -5.49055000 2.15642500 0.08184200
C -6.79478000 1.84890600 0.00556300
C -7.18632400 0.56742200 0.05417800
C -6.27373500 -0.40861000 0.17926500
C -4.96513600 -0.10272700 0.25640100
C -4.05874300 -1.10500900 0.38201400
C -2.75575000 -0.74024400 0.45539900
H -2.02517900 -1.55860400 0.56492800
O -4.36087500 -2.27769800 0.42873900

H -6.63661600 -1.45063500 0.21516000
H -8.25986600 0.31746200 -0.00908000
H -7.54759400 2.64995300 -0.09762700
H -5.21303400 3.22407100 0.03754500
O -2.86666300 2.66638900 0.22768400
H -0.85016200 1.89298500 0.22538200
C 1.35311700 0.71947700 -0.13194900
C 2.47335600 -0.23384500 -0.57020600
H 3.44404300 0.30620500 -0.66056500
H 2.61011800 -1.06047000 0.16421700
H 2.24456400 -0.68945400 -1.56099800
H 1.26781700 1.55724400 -0.86857300
O 1.69219000 1.29615500 1.10898500
H 2.48980900 1.78218500 0.98906500
H 0.11554700 -0.86056800 0.70082600
H -0.29686500 -0.37869000 -1.00702400

2-Aminoethanol-naphthoquinone ground (**2d**)
C 0.00000000 0.00000000 0.00000000
N -1.21004600 -0.66918300 -0.41901100
C -2.40422500 0.03817700 -0.35237500
C -3.53892800 -0.68321700 -0.16438500
C -4.72863600 -0.03066000 -0.09972400
C -5.85530000 -0.74431100 0.08535700
C -7.04376000 -0.12419000 0.15202200
C -7.11274300 1.20975300 0.03478600
C -5.99263600 1.92578400 -0.14870600
C -4.79950600 1.30617400 -0.21613300
C -3.67880000 2.04870300 -0.40193700
C -2.50535500 1.37346500 -0.46333000
H -1.60220200 1.98506800 -0.62128200
O -3.68797500 3.25563300 -0.51209700
H -6.09172100 3.02153300 -0.24006200
H -8.09192400 1.71698100 0.08976100
H -7.96683900 -0.71080400 0.30320200
H -5.84492800 -1.84369800 0.18655800
O -3.43650300 -1.88573800 -0.05182200
H -1.30580000 -1.62345300 -0.06980800
C 1.16013500 -1.00521700 0.03373100
O 2.33058600 -0.35603700 0.47293700
H 3.02894700 -0.98825900 0.45989300
H 0.95075600 -1.84591600 0.73837600
H 1.36153100 -1.42792800 -0.98008500
H 0.26792800 0.82018700 -0.70782600
H -0.14406600 0.42177100 1.02339800

2-Aminoethanol-naphthoquinone Radical Anion (**2d**)
C 0.00000000 0.00000000 0.00000000
N -1.06262200 -0.85301300 -0.48040600
C -2.37156900 -0.40878700 -0.33834100
C -3.33565800 -1.35743200 -0.22041700

C -4.62938800 -0.96554100 -0.08588200
C -5.58720600 -1.90474400 0.02990400
C -6.87322500 -1.54442100 0.16314400
C -7.20847400 -0.24628100 0.18181500
C -6.25751600 0.69377400 0.06787400
C -4.96704000 0.33495800 -0.06608900
C -4.02081100 1.30119300 -0.17964900
C -2.73843400 0.88361800 -0.31317300
H -1.97870700 1.67597700 -0.41152500
O -4.27207200 2.48677600 -0.16700700
H -6.57434700 1.75114400 0.08827900
H -8.26730000 0.04698500 0.29126800
H -7.65759900 -2.31573700 0.25735800
H -5.35634000 -2.98436800 0.01923300
O -2.99385000 -2.52030900 -0.23054700
H -0.95869400 -1.83682300 -0.22928800
C 1.33711000 -0.75128500 -0.07225400
O 2.36538700 0.07232100 0.42635500
H 3.17487700 -0.40307400 0.34693800
H 1.31555500 -1.68211700 0.54437100
H 1.59441900 -1.02321500 -1.12435400
H 0.08291900 0.92285600 -0.62206900
H -0.20072400 0.28153200 1.06133800

2-Aminopropanol-naphthoquinone ground (**2e**)
C 0.00000000 0.00000000 0.00000000
N -1.19392600 -0.71380700 -0.39274200
C -2.41272900 -0.05569000 -0.28324500
C -3.51224800 -0.82175400 -0.06507900
C -4.72335900 -0.21593900 0.04254100
C -5.81523500 -0.97344200 0.25929100
C -7.02366400 -0.39995900 0.37028600
C -7.14743000 0.93101100 0.26523600
C -6.06238200 1.69069100 0.04937500
C -4.84936200 1.11785300 -0.06176200
C -3.76467900 1.90407000 -0.27838500
C -2.56869600 1.27525800 -0.38185000
H -1.69489700 1.92178000 -0.56439700
O -3.82258800 3.11040900 -0.37931200
H -6.20616700 2.78241000 -0.03055600
H -8.14280400 1.39990500 0.35724700
H -7.91797300 -1.02253400 0.54860400
H -5.75953900 -2.07212400 0.35234800
O -3.36102000 -2.02020900 0.03533300
H -1.23871800 -1.67605000 -0.05570200
C 1.20078700 -0.96004100 0.01338100
C 2.49562200 -0.24519700 0.42329300
O 3.55974100 -1.16831300 0.42548100
H 4.34102500 -0.70093400 0.66817500
H 2.75415800 0.57216700 -0.29197300
H 2.41521900 0.18547600 1.45013700

H 1.00015900 -1.79771600 0.72373100
H 1.33017800 -1.40815800 -1.00097400
H 0.21913900 0.82780900 -0.71532700
H -0.13848800 0.42043200 1.02458200

2-Aminopropanol-naphthoquinone Radical Anion (**2e**)
C 0.00000000 0.00000000 0.00000000
N -1.06089900 -0.86402400 -0.46602600
C -2.37208500 -0.43796800 -0.29323600
C -3.32193400 -1.39844000 -0.15658700
C -4.61667500 -1.02214900 0.00904700
C -5.56047000 -1.97278300 0.14517500
C -6.84702000 -1.62806500 0.31089100
C -7.19686900 -0.33403100 0.34116200
C -6.26015800 0.61748200 0.20612300
C -4.96919600 0.27419400 0.04034200
C -4.03737000 1.25173200 -0.09276300
C -2.75356200 0.84990100 -0.25663300
H -2.00525900 1.65109400 -0.37052200
O -4.30107300 2.43443000 -0.07088000
H -6.58867200 1.67106400 0.23705400
H -8.25599000 -0.05355800 0.47789000
H -7.61966700 -2.40886200 0.42295400
H -5.31737200 -3.04954400 0.12638300
O -2.96763000 -2.55743000 -0.17795700
H -0.93636400 -1.84869700 -0.22849000
C 1.34661400 -0.73741300 -0.08123100
C 2.50702100 0.14312800 0.40222400
O 3.71252700 -0.58029500 0.31327600
H 4.40649900 -0.01318500 0.60402800
H 2.61206800 1.06025100 -0.22577300
H 2.36993100 0.44844700 1.46727800
H 1.30200500 -1.66406700 0.53995400
H 1.53512600 -1.05272300 -1.13551300
H 0.06445100 0.92064300 -0.62709300
H -0.19261600 0.28559400 1.06162400

2-Methylamine-naphthoquinone ground (**2f**)
C 0.00000000 0.00000000 0.00000000
N -1.15647300 0.69905300 0.50325200
C -2.36424000 0.01286700 0.53871600
C -2.47618400 -1.32055000 0.66083800
C -3.66082500 -1.97756800 0.70002300
C -4.78171100 -1.21809500 0.60752000
C -5.98559000 -1.81930200 0.64180900
C -7.10606500 -1.08626000 0.55064400
C -7.02675100 0.24634600 0.42496200
C -5.82752800 0.84804100 0.39086900
C -4.70024100 0.11737000 0.48240900
C -3.49950700 0.75169400 0.44521000
O -3.38956200 1.95241400 0.32131500

H -5.80893400 1.94692800 0.28662600
H -7.95003900 0.84717200 0.34951100
H -8.09404500 -1.57841900 0.57836800
H -6.09345800 -2.91331100 0.74366600
O -3.67819700 -3.18410300 0.81257200
H -1.57159500 -1.94504700 0.74284300
H -1.26068800 1.65033500 0.14835400
H 0.83853700 0.72764900 -0.08697600
H 0.33727600 -0.80417400 0.69390300
H -0.20068000 -0.41332700 -1.01537400

2-Methylamine-naphthoquinone Radical Anion (**2f**)
C 0.00000000 0.00000000 0.00000000
N 1.03048300 0.85244300 -0.53927000
C 2.34337800 0.40166200 -0.47671400
C 2.70137000 -0.89338900 -0.46529200
C 3.98639800 -1.32025600 -0.40987500
C 4.94523400 -0.36098600 -0.36323100
C 6.23878800 -0.72923400 -0.30789300
C 7.20209000 0.20397700 -0.26001300
C 6.87614500 1.50460600 -0.26697700
C 5.58708500 1.87417600 -0.32243800
C 4.61667600 0.94198900 -0.37109800
C 3.32005700 1.34348700 -0.42668900
O 2.98811000 2.50923500 -0.42335300
H 5.36426300 2.95542500 -0.32638300
H 7.67049100 2.27047300 -0.22688200
H 8.26342100 -0.09699000 -0.21430600
H 6.54831600 -1.78894700 -0.29976600
O 4.22797600 -2.50781600 -0.40372600
H 1.92998600 -1.67954900 -0.50912100
H 0.94436600 1.83487800 -0.27629700
H -0.95995500 0.56447600 -0.00590000
H -0.15499300 -0.91259600 -0.62056300
H 0.22860300 -0.27466700 1.05564900

2-Ethylamine-naphthoquinone ground (**2g**)
C 0.00000000 0.00000000 0.00000000
N -1.17418600 -0.70538300 -0.46036000
C -2.39595700 -0.04568000 -0.40855800
C -3.50732800 -0.81313000 -0.26961100
C -4.72194000 -0.20663600 -0.22181600
C -4.83984500 1.12925000 -0.30650700
C -6.05663500 1.70262500 -0.25554000
C -7.15358400 0.94132100 -0.12077900
C -7.03791500 -0.39184400 -0.03645400
C -5.82569200 -0.96579300 -0.08630800
H -5.77680400 -2.06628300 -0.01306400
H -7.94213900 -1.01572900 0.07415500
H -8.15216500 1.41046600 -0.07920800
H -6.19416600 2.79604500 -0.32154900

C -3.74326600 1.91698600 -0.44345500
C -2.54412700 1.28731100 -0.48989700
H -1.66015500 1.93490400 -0.60863400
O -3.79412200 3.12519300 -0.52375300
O -3.36368300 -2.01360400 -0.18341300
H -1.23890500 -1.67112700 -0.13691400
C 1.19223900 -0.96493000 0.06159300
H 2.11239700 -0.44423500 0.41529900
H 0.98969500 -1.80942800 0.76056500
H 1.40947900 -1.39759900 -0.94252200
H 0.26010600 0.83527600 -0.69345300
H -0.18546000 0.40963100 1.02186700

2-Ethylamine-naphthoquinone Radical Anion (**2g**)
C 0.00000000 0.00000000 0.00000000
N -1.00943000 0.89064900 0.52536100
C -2.33964200 0.50066900 0.43014100
C -3.26970400 1.48751700 0.36270000
C -4.58208100 1.14766100 0.27572400
C -4.97092700 -0.13839700 0.25294700
C -6.27864800 -0.44524100 0.16550800
C -7.19588300 0.53232800 0.10162600
C -6.80978500 1.81615800 0.12456500
C -5.50640700 2.12458500 0.21097800
H -5.23327300 3.19415700 0.22666400
H -7.56662100 2.61836300 0.07208400
H -8.26880400 0.28186100 0.03023600
H -6.63705000 -1.48923400 0.14389500
C -4.05946900 -1.14185400 0.31687300
C -2.75749500 -0.77611500 0.40450100
H -2.02539400 -1.59797200 0.46332600
O -4.35657900 -2.31668300 0.29951400
O -2.88328900 2.63633500 0.37280700
H -0.87283700 1.87219300 0.28167900
C 1.36459400 0.70295700 0.00714400
H 2.16212700 0.03572300 -0.39492600
H 1.34478300 1.62472400 -0.61952700
H 1.65684400 0.99950500 1.04121000
H 0.07808800 -0.92407500 0.62145400
H -0.25628400 -0.27912400 -1.05014900

2-Isobutylamine-naphthoquinone ground (**2h**)
C 0.00000000 0.00000000 0.00000000
N -1.22587300 -0.74047100 -0.19249800
C -2.41594600 -0.02819200 -0.27409500
C -3.54688100 -0.66775400 0.12007900
C -4.73218900 -0.00786000 0.05005600
C -5.85539700 -0.63995300 0.43966400
C -7.03939100 -0.01055300 0.38113300
C -7.10735600 1.25161900 -0.06622000
C -5.99093400 1.88636200 -0.45572900

C -4.80235900 1.25725000 -0.39763400
C -3.68531200 1.91739400 -0.79524100
C -2.51619300 1.23671100 -0.71615900
H -1.61597200 1.77942500 -1.04766600
O -3.69312500 3.05768300 -1.20577300
H -6.08903100 2.92477400 -0.81761300
H -8.08270400 1.76699100 -0.11316300
H -7.95941300 -0.53046200 0.70121400
H -5.84577700 -1.67840300 0.81429700
O -3.44535800 -1.80400500 0.52984300
H -1.30959800 -1.57446600 0.38980900
C 1.17406500 -0.95901600 0.28093700
C 1.39335500 -1.93705900 -0.88577000
H 2.24321200 -2.62701300 -0.67756500
H 0.49405200 -2.56931500 -1.06646100
H 1.62148300 -1.39120200 -1.82963700
C 2.46235200 -0.16572400 0.55911900
H 3.31128300 -0.84625300 0.79994300
H 2.75676100 0.44644400 -0.32386600
H 2.33329300 0.52418900 1.42468700
H 0.93070800 -1.55554900 1.19552800
H 0.24652500 0.59942600 -0.90771200
H -0.12217000 0.68616900 0.87168600

2-Isobutylamine-naphthoquinone Radical Anion (**2h**)
C 0.00000000 0.00000000 0.00000000
N -1.01489300 -0.91318100 -0.47351000
C -2.34209500 -0.50682500 -0.41283900
C -3.28109500 -1.47993600 -0.29134600
C -4.59094700 -1.12440800 -0.23471900
C -5.52430000 -2.08744300 -0.11493400
C -6.82540400 -1.76326400 -0.05621200
C -7.20027600 -0.47732900 -0.11675000
C -6.27414400 0.48654700 -0.23606500
C -4.96866800 0.16383200 -0.29484800
C -4.04813400 1.15378200 -0.41439900
C -2.74888700 0.77253700 -0.46944000
H -2.00975400 1.58355200 -0.57321500
O -4.33487400 2.32992700 -0.47200500
H -6.62342000 1.53284300 -0.28249000
H -8.27133200 -0.21354000 -0.06807500
H -7.58938400 -2.55437200 0.04217900
H -5.26072700 -3.15819300 -0.06159500
O -2.90437000 -2.63029000 -0.22774000
H -0.88817100 -1.87856800 -0.16755600
C 1.37356500 -0.69801700 0.05618800
C 1.80528700 -1.20315200 -1.33100900
H 2.79860300 -1.70594500 -1.28447000
H 1.08159000 -1.94366100 -1.74216800
H 1.88297300 -0.36266000 -2.05793800
C 2.43753000 0.25296000 0.63064700

H 3.42812700 -0.25108100 0.71102000
H 2.56446800 1.15204700 -0.01481100
H 2.15844600 0.60252200 1.65131400
H 1.29285900 -1.58019700 0.73941300
H 0.08318000 0.88353800 -0.67562500
H -0.27141500 0.34587300 1.02581500

2-Pyrrolidine-naphthoquinone ground (**2i**)
C 0.00000000 0.00000000 0.00000000
N -0.05418100 1.26254000 -0.70594400
C -1.25505100 1.96530400 -0.60960100
C -2.42320700 1.54943600 -1.17050200
C -3.53495300 2.31712200 -1.01072000
C -4.69431400 1.91273000 -1.56513400
C -5.80805400 2.64858200 -1.42466000
C -5.77312500 3.79427400 -0.72946700
C -4.62250900 4.20398500 -0.17408200
C -3.50538800 3.46633700 -0.31455000
C -2.35527400 3.90076500 0.25783200
C -1.26409900 3.11841100 0.08378100
H -0.32227800 3.46391400 0.54670800
O -2.26821400 4.92639600 0.89747100
H -4.63352300 5.15222500 0.39126700
H -6.69040000 4.39801800 -0.61487700
H -6.75564400 2.31238900 -1.88079800
H -4.77592700 0.97790700 -2.14664600
O -2.46368200 0.51154400 -1.79357300
C 0.46534900 1.02466700 -2.03419100
C 1.70194500 0.16216200 -1.74342600
C 1.26641900 -0.69327700 -0.53897900
H 2.07327600 -0.73807600 0.23102000
H 1.03537500 -1.73770200 -0.85818100
H 2.55351800 0.82325900 -1.45148900
H 2.01756800 -0.45000000 -2.62160500
H 0.72065300 1.97966800 -2.55295800
H -0.24452300 0.44165100 -2.66566400
H 0.04321300 0.16660300 1.10292600
H -0.89247900 -0.63030600 -0.22521500

2-Pyrrolidine-naphthoquinone Radical Anion (**2i**)
C 0.00000000 0.00000000 0.00000000
N -0.65228900 0.46171900 -1.20691200
C -2.04661200 0.42950200 -1.19837100
C -2.77774800 -0.71831000 -1.18781600
C -4.13523200 -0.62876700 -1.17941200
C -4.86540100 -1.76093800 -1.17121100
C -6.20644800 -1.70486100 -1.16257500
C -6.82927600 -0.51776000 -1.16212100
C -6.10938600 0.61446200 -1.17013500
C -4.76453000 0.55875300 -1.17865500
C -4.05929600 1.71708000 -1.18469900

C -2.71051000 1.59985500 -1.19291800
H -2.12719500 2.53816400 -1.19758900
O -4.57082100 2.81570400 -1.18280800
H -6.65799500 1.57254700 -1.16886500
H -7.93247200 -0.47376800 -1.15478100
H -6.80004900 -2.63577600 -1.15554300
H -4.40119900 -2.76255100 -1.17116300
O -2.22153500 -1.79425200 -1.18057400
C 0.01485200 -0.17997300 -2.31771700
C 1.49340800 -0.03013200 -1.93165000
C 1.47889900 -0.17139300 -0.39803200
H 2.12827600 0.60056100 0.07979700
H 1.85349300 -1.17669900 -0.08959100
H 1.84814100 0.99014900 -2.21562900
H 2.14876200 -0.78190400 -2.43255900
H -0.22820600 0.31827300 -3.28673800
H -0.23443500 -1.26410800 -2.38907600
H -0.13491800 0.73685900 0.82759000
H -0.41067100 -0.98206100 0.33381200

2-Phenehtylamine-naphthoquinone ground (**2j**)
C 0.00000000 0.00000000 0.00000000
N -1.24927700 0.69094300 0.22102700
C -2.39362100 -0.04806200 0.49898000
C -2.40207700 -1.29231200 1.00647600
C -3.53105100 -1.99211000 1.27521700
C -4.70876800 -1.37510900 1.00394300
C -5.86150900 -2.02393500 1.25310100
C -7.03664400 -1.43171400 0.98961900
C -7.06260800 -0.19294200 0.47708300
C -5.91437400 0.45598100 0.22835200
C -4.73259600 -0.13323200 0.49153800
C -3.58526500 0.54573600 0.23060100
O -3.57267100 1.66120600 -0.24368400
H -5.98295800 1.47416600 -0.19297800
H -8.03065600 0.29178000 0.26043500
H -7.98294200 -1.96281900 1.19361300
H -5.88226300 -3.04442200 1.67375700
O -3.45248200 -3.11133300 1.73354800
H -1.45498400 -1.80624600 1.23404400
H -1.42687200 1.46292200 -0.42250400
C 0.82967100 -0.14939200 1.28623400
C 2.11576500 -0.89788200 1.00709000
C 2.14048900 -2.24277400 1.02597600
C 3.27475300 -2.91551200 0.77679000
C 4.40488700 -2.24646500 0.50301300
C 4.39303400 -0.90516200 0.47923300
C 3.25504400 -0.23888200 0.72925700
H 3.26047500 0.86401500 0.70174700
H 5.31973400 -0.35000500 0.25191200
H 5.33923800 -2.79673600 0.29630300

H 3.27813900 -4.01928400 0.79510500
H 1.21708800 -2.80546300 1.24578600
H 1.05272000 0.85987900 1.70631700
H 0.25955000 -0.67769000 2.08396500
H -0.16440800 -0.97105700 -0.52387000
H 0.58957900 0.62536200 -0.71106100

2-Phenehtylamine-naphthoquinone Radical Anion (**2j**)
C 0.00000000 0.00000000 0.00000000
N 1.30110600 -0.48848600 0.39427500
C 2.38226500 0.38558600 0.39604000
C 2.29062100 1.72319500 0.48651100
C 3.35961800 2.55610100 0.49225300
C 4.58310500 1.97688900 0.39796600
C 5.67989200 2.75722100 0.40051900
C 6.89881100 2.20370800 0.30590800
C 7.02431700 0.87232100 0.20803600
C 5.93203100 0.09237600 0.20580200
C 4.70673300 0.64244300 0.30124900
C 3.61776300 -0.16970200 0.29684800
O 3.69491300 -1.37534000 0.19754100
H 6.08227800 -0.99834300 0.12406800
H 8.02818100 0.41944300 0.12949500
H 7.79929800 2.84267900 0.30796700
H 5.61850500 3.85672800 0.47856100
O 3.19129000 3.75329700 0.57761600
H 1.30529400 2.20874800 0.56569100
H 1.53999300 -1.40803900 0.02147600
C -0.83817000 0.48345100 1.19533100
C -2.18065500 1.00523800 0.72878200
C -2.31381600 2.28147200 0.32480500
C -3.49883300 2.75264400 -0.09347100
C -4.57147200 1.94714900 -0.11362300
C -4.45143300 0.67178500 0.28491700
C -3.26322300 0.20727000 0.70186200
H -3.17965300 -0.84507600 1.02241000
H -5.33046100 0.00420500 0.26776600
H -5.54731700 2.33113600 -0.45832300
H -3.59126800 3.80224600 -0.42279000
H -1.43868900 2.95355600 0.33254800
H -0.97876800 -0.35584300 1.91690300
H -0.31201400 1.27822000 1.77153700
H 0.08516300 0.76594800 -0.80653100
H -0.53766100 -0.86074600 -0.46295200

2-Cadaverine-naphthoquinone ground (**2k**)
C 0.00000000 0.00000000 0.00000000
N -1.21435600 -0.72877500 -0.28822300
C -2.42357400 -0.05425200 -0.17292100
C -3.51889600 -0.79692500 0.13048700
C -4.72126800 -0.17547800 0.24620500

C	-5.80921600	-0.91008000	0.54551800
C	-7.00918800	-0.32105900	0.66595300
C	-7.12828100	1.00268100	0.48856700
C	-6.04700800	1.73954800	0.19042100
C	-4.84252800	1.15103300	0.06893400
C	-3.76160800	1.91419500	-0.23251100
C	-2.57448500	1.27001200	-0.34328200
H	-1.70404200	1.89756100	-0.59482000
O	-3.81572600	3.11314700	-0.40092800
H	-6.18681000	2.82599900	0.05231400
H	-8.11663600	1.48448300	0.58849000
H	-7.90031000	-0.92500600	0.91130400
H	-5.75698900	-2.00207400	0.69959100
O	-3.37138500	-1.98854000	0.29628900
H	-1.25019100	-1.66505500	0.11621000
C	1.19434800	-0.96835900	0.03250000
C	2.51431500	-0.24082100	0.33533000
C	3.70932600	-1.20680500	0.36998200
C	5.02589100	-0.47501100	0.66809200
N	6.13438600	-1.39595600	0.70118400
H	6.99600700	-0.91760300	0.96484600
H	5.98985000	-2.11497500	1.41024300
H	5.23012900	0.29933900	-0.10817000
H	4.96946000	0.03304500	1.65889200
H	3.53553300	-1.98688500	1.14913300
H	3.79340800	-1.73060700	-0.61206800
H	2.69328700	0.53946600	-0.44210700
H	2.43212200	0.28076800	1.31843200
H	1.01507500	-1.75074400	0.80835800
H	1.27339300	-1.48953900	-0.95151100
H	0.19174500	0.77114800	-0.78346700
H	-0.09172800	0.49560900	0.99586500

2-Cadaverine-naphthoquinone Radical Anion (**2k**)

C	0.00000000	0.00000000	0.00000000
N	1.09114000	-0.85902400	0.40042000
C	2.38830300	-0.41145800	0.18239200
C	3.34573600	-1.35675900	0.00051600
C	4.62778500	-0.95992000	-0.20961400
C	5.57930400	-1.89554400	-0.38872000
C	6.85377300	-1.53067100	-0.59843100
C	7.18368600	-0.23141600	-0.63051900
C	6.23908500	0.70517400	-0.45291300
C	4.96027000	0.34167500	-0.24224800
C	4.02023100	1.30435900	-0.06550200
C	2.74954500	0.88216100	0.14332400
H	1.99480100	1.67141800	0.29230000
O	4.26616200	2.49088100	-0.08697700
H	6.55120400	1.76368300	-0.48658400
H	8.23285700	0.06573700	-0.80344100
H	7.63279100	-2.29924500	-0.74496800

H 5.35239700 -2.97585600 -0.36983700
O 3.00881600 -2.52087200 0.02330400
H 0.96973300 -1.84029300 0.14774900
C -1.33218100 -0.75933100 0.11811700
C -2.52812800 0.11301700 -0.29667100
C -3.86064100 -0.64422600 -0.18105200
C -5.05244900 0.23246200 -0.59184100
N -6.29242800 -0.49438100 -0.48162900
H -7.07411400 0.06819000 -0.81809800
H -6.27956600 -1.32828200 -1.06906900
H -5.11396300 1.14021700 0.05322400
H -4.94030100 0.56538300 -1.64994200
H -3.83015400 -1.55317500 -0.82811300
H -4.00277100 -0.98945900 0.87083800
H -2.56373600 1.02272600 0.34865700
H -2.38823600 0.45622900 -1.34925600
H -1.29656800 -1.67088900 -0.52532200
H -1.46887900 -1.10250400 1.17154000
H -0.04951300 0.90376400 0.65270000
H 0.14491900 0.31438800 -1.06110700

2-Histamine-naphthoquinone ground (**2l**)
C 0.00000000 0.00000000 0.00000000
N -1.06085200 -0.95501500 -0.22968300
C -2.37273500 -0.50224500 -0.15948500
C -3.31842100 -1.40226100 0.21360000
O -2.95924500 -2.52989900 0.47547600
C -4.61380100 -1.00017000 0.28868100
C -5.55353200 -1.89096100 0.65800700
C -6.84062200 -1.51948200 0.74041600
C -7.19513100 -0.25832300 0.45472700
C -6.26245800 0.63362700 0.08650000
C -4.97091600 0.26352100 0.00348900
C -4.04330300 1.18118300 -0.36952200
O -4.31141900 2.33267400 -0.63584400
C -2.75879200 0.75449500 -0.43702800
H -2.01440600 1.50578000 -0.74722300
H -6.59462200 1.66187400 -0.13996700
H -8.25467600 0.04454100 0.52332200
H -7.60997100 -2.25122800 1.04346400
H -5.30657600 -2.93905900 0.90194400
H -0.93187400 -1.84955500 0.24442600
C 1.34816300 -0.73340600 0.08107700
C 2.53185200 0.16049700 0.31565200
C 2.56750700 1.48614000 0.50872300
N 3.86191000 1.90035900 0.68899800
C 4.52873500 0.69651800 0.62031800
N 3.72358700 -0.26060700 0.40998100
H 5.50229100 0.53756400 1.11449300
H 4.09433200 2.60914500 1.38389000
H 1.71019300 2.17839500 0.53795800

H 1.31284100 -1.48400500 0.90563600
H 1.52026100 -1.29012100 -0.87028500
H 0.04875600 0.73836600 -0.83551800
H -0.18832600 0.52911300 0.96472900

2-Histamine-naphthoquinone Radical Anion (**2l**)
C 0.00000000 0.00000000 0.00000000
N -1.17067100 -0.78139100 -0.33041300
C -2.41837500 -0.20146300 -0.13577200
C -3.45392100 -1.03767300 0.13244200
O -3.22331200 -2.22565500 0.20078300
C -4.69106600 -0.51008100 0.32334200
C -5.71970900 -1.33731200 0.58902700
C -6.95203800 -0.84184100 0.78206000
C -7.16292500 0.48037600 0.71074700
C -6.14112800 1.30935900 0.44662300
C -4.90423200 0.81483300 0.25302200
C -3.88455700 1.67036400 -0.01162100
O -4.02207000 2.87225000 -0.08447000
C -2.66090700 1.11865900 -0.19840700
H -1.84115700 1.82152900 -0.41918700
H -6.35573000 2.39119200 0.39666700
H -8.17771700 0.88524700 0.86994200
H -7.79445000 -1.52161300 0.99986800
H -5.59161400 -2.43184900 0.65672100
H -1.13622800 -1.74869300 -0.00675200
C 1.25441600 -0.88332600 -0.09042200
C 2.53999300 -0.17642000 0.23017600
C 2.73541600 1.08919500 0.62502400
N 4.07337700 1.32181500 0.81291700
C 4.59262800 0.07719100 0.53026000
N 3.67603700 -0.73799300 0.20788000
H 5.55230800 -0.26639100 0.95246000
H 4.40376300 1.88050300 1.59903700
H 1.96612200 1.85962200 0.79732900
H 1.15095200 -1.74676700 0.60836900
H 1.33737500 -1.29737900 -1.12304800
H 0.11510600 0.85167900 -0.71207000
H -0.10198900 0.38667000 1.04221600

2-Tyramine-naphthoquinone ground (**2m**)
C 0.00000000 0.00000000 0.00000000
N -0.79420700 0.76289400 -0.93542800
C -2.10944900 1.05445700 -0.59849300
C -2.98439100 1.23094000 -1.62135300
C -4.27857600 1.52608500 -1.33296500
C -5.14861700 1.70305500 -2.34517200
C -6.43302400 1.99555600 -2.08797700
C -6.85415400 2.11265600 -0.82038500
C -5.99114000 1.93687600 0.19224200
C -4.70253300 1.64321500 -0.06311000

C -3.84636400 1.46865500 0.97531400
C -2.56107400 1.17588900 0.66100900
H -1.87006700 1.04308400 1.50956200
O -4.17675500 1.56180100 2.13761100
H -6.37721100 2.04167700 1.22124900
H -7.91132300 2.35357000 -0.61214200
H -7.14516600 2.14054000 -2.91914500
H -4.84485200 1.61643900 -3.40306000
O -2.56946200 1.10606500 -2.75339600
H -0.68606800 0.47858600 -1.90963500
C 1.31728200 -0.42368800 -0.67039300
C 2.06683000 0.78450300 -1.18947400
C 1.97524600 1.15961000 -2.47712600
C 2.63472700 2.23781200 -2.92747100
C 3.40612600 2.97786400 -2.11259900
C 3.49289300 2.59850400 -0.82608500
C 2.83583200 1.52149000 -0.36987400
H 2.92673700 1.24295400 0.69386300
H 4.11575900 3.18699100 -0.13073300
O 4.07360400 4.07268000 -2.56649400
H 4.55699600 4.45574600 -1.81628400
H 2.54146400 2.52637700 -3.98856100
H 1.34840100 0.58181700 -3.17750000
H 1.95511400 -0.98291200 0.05474300
H 1.10402000 -1.13403500 -1.50403700
H 0.23658900 0.61015200 0.90430000
H -0.55799900 -0.91569800 0.31097100

2-Tyramine-naphthoquinone Radical Anion (**2m**)
C 0.00000000 0.00000000 0.00000000
N -0.61105400 -1.25723100 -0.36575900
C -1.94227000 -1.25230900 -0.76102400
C -2.65354300 -2.38904900 -0.54930700
C -3.95692200 -2.42687300 -0.93005200
C -4.66482000 -3.55325600 -0.72270300
C -5.95429600 -3.61731600 -1.08952100
C -6.54221800 -2.55825100 -1.66448300
C -5.84125700 -1.43297700 -1.87309500
C -4.54795400 -1.36616600 -1.50587500
C -3.85731800 -0.21865500 -1.72510500
C -2.55895700 -0.20686600 -1.33704600
H -2.00256800 0.72595000 -1.52534200
O -4.34208000 0.76603700 -2.23906500
H -6.36167500 -0.58347200 -2.34890600
H -7.60306500 -2.61420800 -1.96528700
H -6.53334100 -4.54177200 -0.91894700
H -4.22068900 -4.44836400 -0.25320300
O -2.09123700 -3.32161700 -0.01694400
H -0.38308500 -2.03316000 0.25679100
C 1.37273300 -0.26315300 0.64072100
C 2.25399700 -1.06600500 -0.29190700

C 2.36536800 -2.39988500 -0.16741400
C 3.14069000 -3.10593600 -1.00444000
C 3.82995600 -2.50908300 -1.99207800
C 3.71374700 -1.17543200 -2.11219900
C 2.94005800 -0.46405400 -1.27843400
H 2.86487400 0.62879000 -1.41042300
H 4.26453400 -0.65584200 -2.91497700
O 4.61460900 -3.22322400 -2.84324900
H 5.00836000 -2.59965300 -3.47518300
H 3.21373100 -4.20034700 -0.88275100
H 1.81194900 -2.92858000 0.62726400
H 1.87012500 0.70310800 0.89434500
H 1.23948200 -0.80365900 1.60774300
H 0.14079200 0.64539400 -0.89981300
H -0.64635600 0.53422300 0.73715600

2-Tryptamine-naphthoquinone ground (**2n**)
C 0.00000000 0.00000000 0.00000000
N -1.41825400 0.05645000 -0.27183400
C -2.17544200 1.00810800 0.39969400
C -3.44680000 0.66611600 0.73054500
C -4.22082100 1.57094000 1.38415200
C -5.48167000 1.23300900 1.71422700
C -6.26765100 2.10837000 2.36023500
C -5.79886400 3.32333800 2.67975400
C -4.54246800 3.66465500 2.35410000
C -3.75226700 2.78857300 1.70604300
C -2.48508700 3.15338800 1.38517400
C -1.73472000 2.23308800 0.73226800
H -0.71056100 2.54156400 0.46718500
O -2.01253500 4.23705200 1.65186200
H -4.19402400 4.67393400 2.63488000
H -6.44831600 4.04127100 3.21067700
H -7.30158600 1.83006500 2.62954500
H -5.90805300 0.24411100 1.47074600
O -3.83144100 -0.44450100 0.43452500
H -1.86989000 -0.85867900 -0.28425000
C 0.29687000 -0.35668900 1.46431100
C 1.77405400 -0.49712700 1.69486300
C 2.67533100 0.48216600 1.86427900
C 3.86541000 -0.10972300 2.03587600
N 3.69162800 -1.36430900 1.97479800
C 2.46925100 -1.64263200 1.77430000
H 2.11815700 -2.68380500 1.68996000
H 4.45976000 -2.07158500 2.07669600
C 4.98080000 0.60228600 2.23053600
C 4.87045500 1.94203500 2.24839700
C 3.67681800 2.53972900 2.07597200
C 2.56047700 1.81575900 1.88164000
H 1.58374300 2.30840000 1.74054400
H 3.61172000 3.64149900 2.09404400

H 5.77223100 2.55873100 2.40636100
H 5.95501700 0.10378800 2.37060300
H -0.10017500 0.42098300 2.15669000
H -0.21298900 -1.31261800 1.72958000
H 0.41977500 -0.80253800 -0.65138900
H 0.51489800 0.93827100 -0.31234100

2-Tryptamine-naphthoquinone Radical Anion (**2n**)
C 0.00000000 0.00000000 0.00000000
N 1.40281000 -0.31859800 0.13835800
C 2.33209800 0.61708600 -0.29851300
C 3.48430000 0.14057900 -0.83530100
C 4.42009400 1.02380300 -1.27029700
C 5.56298700 0.55297700 -1.80415600
C 6.50409700 1.40403700 -2.24160300
C 6.30850100 2.72733300 -2.14870800
C 5.17056500 3.20174700 -1.61850800
C 4.22524700 2.35025300 -1.17891100
C 3.08189800 2.85018600 -0.64542000
C 2.16567600 1.94795700 -0.21785700
H 1.24481700 2.36294700 0.22274300
O 2.85497600 4.03598700 -0.53988600
H 5.04753600 4.29734000 -1.55991400
H 7.08502500 3.42468600 -2.50899200
H 7.44108000 1.01642300 -2.67843300
H 5.76534800 -0.52817500 -1.89988400
O 3.62046200 -1.06133200 -0.91222800
H 1.63909800 -1.26604200 -0.15847100
C -0.41401800 0.17317900 -1.46906900
C -1.89170500 0.41305500 -1.58675900
C -2.55661800 1.55939700 -1.37746100
C -3.85345600 1.30274900 -1.59748000
N -3.96210500 0.08110100 -1.91948700
C -2.82701800 -0.48757200 -1.92712800
H -2.71458600 -1.55372300 -2.18227600
H -4.87142000 -0.38997000 -2.14727100
C -4.78738400 2.25154400 -1.46898200
C -4.38157500 3.48152100 -1.10856900
C -3.07995000 3.74259700 -0.88642300
C -2.14777400 2.78257500 -1.01879000
H -1.08188100 2.99951900 -0.83597800
H -2.77131700 4.76034000 -0.59077700
H -5.12750400 4.28699100 -0.99334700
H -5.85209900 2.02832300 -1.65275300
H 0.12492300 1.02653400 -1.94144200
H -0.13867700 -0.73974300 -2.04776200
H -0.56789900 -0.85942400 0.42833000
H -0.28273500 0.88321400 0.61888800

1,4-dihydroxy-2-naphthoic acid methyl ester ground (**4**)
C 0.00000000 0.00000000 0.00000000

O -1.21487900 -0.72025800 0.04326400
C -2.38779700 -0.00342800 -0.02414300
C -3.61298500 -0.61743600 -0.00406400
C -3.64429500 -1.95779700 0.08645100
C -4.79219200 -2.67790000 0.11345900
C -5.95055800 -1.97499700 0.04355200
C -7.11677900 -2.64732000 0.06586700
C -8.27993000 -1.98233300 -0.00204300
C -8.27882500 -0.64508200 -0.09170500
C -7.11665100 0.02586900 -0.11364500
C -5.94297000 -0.63317000 -0.04669600
C -4.78283000 0.07980500 -0.07277500
O -4.81401700 1.28894200 -0.15496100
H -7.17379800 1.12576900 -0.18834500
H -9.23631000 -0.09799500 -0.14756000
H -9.23671000 -2.53314000 0.01553500
H -7.16022400 -3.74809200 0.13911300
O -4.74270100 -3.88628900 0.19641900
H -2.71490100 -2.55066900 0.14425900
O -2.30941300 1.20428100 -0.10634400
H 0.83564800 -0.73633200 0.05668300
H 0.08151900 0.55942900 -0.96240500
H 0.07028800 0.68992400 0.87469600

1,4-dihydroxy-2-naphthoic acid methyl ester Radical Anion (**4**)
C 0.00000000 0.00000000 0.00000000
O 1.36648800 -0.35922200 -0.01558300
C 2.29607900 0.65565900 -0.01727000
C 3.64312900 0.40277000 -0.01709300
C 4.04307200 -0.88011200 -0.01724200
C 5.34510300 -1.25648700 -0.01545300
C 6.26454200 -0.25879100 -0.01345000
C 7.57099900 -0.58398100 -0.01053100
C 8.50549900 0.37853800 -0.00803900
C 8.13544900 1.66669200 -0.00905700
C 6.83326100 1.99165500 -0.01232800
C 5.88699700 1.03200800 -0.01408100
C 4.57516600 1.39811300 -0.01610100
O 4.27150300 2.57179500 -0.01647100
H 6.58469300 3.06721900 -0.01322700
H 8.90480000 2.45871200 -0.00703100
H 9.57711600 0.11254100 -0.00496900
H 7.91649500 -1.63259100 -0.00987500
O 5.63096700 -2.43454400 -0.01558600
H 3.31335300 -1.70843600 -0.01860700
O 1.88748900 1.79781200 -0.01562000
H -0.60004500 -0.93999500 0.00783100
H -0.23450200 0.58117300 0.92362100
H -0.25616000 0.58108300 -0.91801500

3-Aminopropanol-DHNA methyl ester ground (**4e**)
C 0.00000000 0.00000000 0.00000000
C -0.86800500 -1.26016200 -0.01220400
C -0.51421000 -2.44280600 -0.55914200
C -1.39039500 -3.48042200 -0.57524600
C -2.60455800 -3.32752800 0.00784300
C -3.47235300 -4.35647100 0.00769500
C -4.67377800 -4.22174100 0.59058300
C -5.00920300 -3.06252600 1.17529900
C -4.14485700 -2.03574800 1.17662000
C -2.93950400 -2.16824200 0.59190400
C -2.07903100 -1.12017500 0.59199400
O -2.35036300 -0.06291700 1.11846400
H -4.46224200 -1.09830900 1.66582200
H -5.99814400 -2.95432000 1.65416000
H -5.38681000 -5.06475700 0.59155600
H -3.23312900 -5.32800500 -0.45878600
O -1.04625100 -4.51350100 -1.10792800
N 0.72132600 -2.69351300 -1.13668100
C 1.79397000 -2.86440700 -0.17751500
C 1.75033200 -4.24363100 0.50003100
C 2.94456200 -4.44791800 1.44307300
O 2.85847900 -5.71899100 2.04439800
H 2.94213000 -6.35963900 1.35859200
H 3.91104700 -4.39873400 0.88650500
H 2.94977600 -3.68559300 2.25878900
H 0.80659700 -4.34741700 1.08659300
H 1.75344400 -5.04383100 -0.27830900
H 2.75476100 -2.76675900 -0.73534600
H 1.78422100 -2.07467700 0.60857700
H 0.68126100 -3.48060900 -1.78520400
O 1.17856200 -0.04185300 -0.79386800
C 1.76176300 1.23274600 -1.00931300
H 2.68059900 1.08707700 -1.62448200
H 1.05914300 1.89199300 -1.57195300
H 2.05409200 1.70135100 -0.04062900
O 0.41520100 0.25196700 1.32260300
H -0.37532000 0.40430200 1.81233700
H -0.61641200 0.86484700 -0.35223300

3-Aminopropanol-DHNA methyl ester Radical Anion (**4e**)
C 0.00000000 0.00000000 0.00000000
C -0.93144600 1.19865300 0.19294700
C -0.61636800 2.32857500 0.86149800
C -1.54414500 3.30561800 1.03244800
C -2.77310700 3.15187000 0.48147300
C -3.69276100 4.12263200 0.63525300
C -4.90995800 3.98760200 0.08613500
C -5.20959900 2.88632300 -0.61784000
C -4.29355000 1.91764600 -0.77240100
C -3.07223700 2.05047200 -0.22187900

C -2.15888300 1.06032000 -0.37757600
O -2.39735900 0.05709200 -1.01416800
H -4.58260800 1.02630800 -1.35618700
H -6.21162200 2.77773900 -1.06859500
H -5.66552200 4.78296000 0.21111900
H -3.48446700 5.04593500 1.20344000
O -1.23148800 4.28831300 1.66962400
N 0.62855000 2.58118300 1.41766800
C 1.64753200 2.92262200 0.44562600
C 1.50194000 4.36604100 -0.06287900
C 2.64118300 4.74568400 -1.01956200
O 2.46226500 6.07063200 -1.46355400
H 2.54233300 6.63160700 -0.71079100
H 3.63246700 4.68793800 -0.50936300
H 2.65043100 4.08411100 -1.91889200
H 0.52917400 4.48269400 -0.59727600
H 1.49737100 5.06996100 0.80350300
H 2.63572800 2.81634500 0.95159300
H 1.64459000 2.22959900 -0.42696800
H 0.57588000 3.28461800 2.15530700
O 1.20853400 0.01727800 0.74842000
C 1.86661400 -1.23823400 0.79038700
H 2.80273100 -1.11223200 1.38344700
H 1.22492600 -1.99706500 1.29759200
H 2.14018900 -1.57447400 -0.23698600
O 0.36924000 -0.07355000 -1.35772200
H -0.43299500 -0.21330000 -1.83191400
H -0.55416900 -0.93351800 0.27098700

3-Methylamine-DHNA methyl ester ground (**4f**)
C 0.00000000 0.00000000 0.00000000
O -1.15011700 -0.79291200 -0.21672600
C -2.37732700 -0.17242400 -0.22252300
C -3.58516800 -0.83325400 -0.28189600
C -3.63147700 -2.19052700 -0.33911500
C -4.84817400 -2.80937600 -0.36127000
C -5.98325500 -2.06526600 -0.34415300
C -7.18208800 -2.68117300 -0.37227300
C -8.31673900 -1.96511400 -0.35905700
C -8.25996700 -0.62712800 -0.31750500
C -7.06837700 -0.01115100 -0.28904200
C -5.92708400 -0.72821800 -0.30263400
C -4.73152800 -0.08686000 -0.27012800
O -4.73849400 1.12529100 -0.23607700
H -7.07825600 1.09224700 -0.25397400
H -9.19268400 -0.03615700 -0.30626700
H -9.29639400 -2.47390900 -0.38280100
H -7.28656400 -3.77960300 -0.40788100
O -4.95833300 -4.01463000 -0.39890200
N -2.44124500 -2.92257100 -0.35441500
C -2.40809600 -4.33253500 -0.65336800

H -1.33994000 -4.64427400 -0.70111500
H -2.87016800 -4.94696100 0.15359300
H -2.84960600 -4.54231500 -1.65481400
H -1.68291300 -2.43463500 -0.82379600
O -2.34976800 1.03822700 -0.14253200
H 0.88479500 -0.67874000 0.01348600
H 0.12579000 0.73323400 -0.83212500
H -0.06830400 0.51641500 0.98719500

3-Methylamine-DHNA methyl ester Radical Anion (**4f**)
C 0.00000000 0.00000000 0.00000000
O 1.25060800 -0.64196700 0.14934500
C 2.38338300 0.13719900 0.17774300
C 3.66993400 -0.35665000 0.17773400
C 3.89904300 -1.69612200 0.14717500
C 5.18790400 -2.14496400 0.11259600
C 6.21231700 -1.25459300 0.12734300
C 7.48321900 -1.70330900 0.09915400
C 8.51105400 -0.84097500 0.11600100
C 8.27451600 0.47704300 0.16110300
C 7.01077300 0.92682900 0.18885300
C 5.97650100 0.06257700 0.17240900
C 4.70533600 0.53717500 0.19780700
O 4.54898200 1.73891000 0.24143600
H 6.87186000 2.02147500 0.22445500
H 9.11910400 1.18825300 0.17472900
H 9.55035500 -1.21313300 0.09337700
H 7.73477100 -2.77761400 0.06281000
O 5.45945200 -3.32426400 0.07134400
N 2.81833300 -2.58190600 0.13216000
C 2.98122300 -3.99912600 0.34054800
H 1.96584400 -4.45476500 0.38314600
H 3.50321700 -4.49452000 -0.51052400
H 3.46928600 -4.20860700 1.32021300
H 2.01219000 -2.23084600 0.64241400
O 2.19199200 1.33561400 0.17591800
H -0.78590200 -0.79000100 -0.04459300
H -0.20419300 0.65680000 0.87909400
H -0.02376500 0.58076300 -0.95291900

3-Ethylamine-DHNA methyl ester ground (**4g**)
C 0.00000000 0.00000000 0.00000000
O -1.01786000 -0.98011600 0.05800600
C -2.27133700 -0.58930600 0.46824600
C -3.37230400 -1.41470200 0.48600000
C -3.25773300 -2.71695600 0.13302600
C -4.36248700 -3.50253200 0.03923000
C -5.57245100 -2.98561400 0.35875200
C -6.66238900 -3.77217400 0.28264500
C -7.86901300 -3.28227100 0.60516200
C -7.98570100 -2.00814500 1.00468300

C	-6.89815300	-1.22500400	1.07806200
C	-5.68264200	-1.71021400	0.75571400
C	-4.59347300	-0.89910300	0.82326500
O	-4.75480400	0.25012300	1.17506800
H	-7.05313500	-0.18311200	1.40816600
H	-8.97878200	-1.60499200	1.27037800
H	-8.76440700	-3.92526500	0.54426400
H	-6.60731600	-4.82725100	-0.03786500
O	-4.22080000	-4.65065500	-0.32352300
N	-2.06800200	-3.36622100	-0.17091700
C	-1.28038700	-3.72534600	0.99262400
C	-1.80367100	-4.99432700	1.67929200
H	-1.12550700	-5.30747300	2.50675400
H	-2.81274600	-4.83172700	2.12312900
H	-1.87189000	-5.84316500	0.96021500
H	-0.23859200	-3.91555100	0.64120100
H	-1.23283600	-2.90210000	1.74403800
H	-2.22146300	-4.17809100	-0.76970500
O	-2.38392100	0.57093500	0.80422500
H	0.92887700	-0.49522400	-0.36867500
H	-0.28251900	0.80809100	-0.71623100
H	0.19698600	0.41245300	1.01836500

3-Ethylamine-DHNA methyl ester Radical Anion (**4g**)

C	0.00000000	0.00000000	0.00000000
O	1.20783000	-0.73358500	-0.05505200
C	2.36268800	-0.05771100	-0.37400600
C	3.61630200	-0.62531200	-0.37597100
C	3.77239100	-1.94196900	-0.10095800
C	5.01637700	-2.47717800	0.01066100
C	6.09762400	-1.69358800	-0.21339800
C	7.32811600	-2.23178100	-0.12028200
C	8.41209500	-1.47504300	-0.34897600
C	8.26612900	-0.18233100	-0.67180600
C	7.03859500	0.35286500	-0.76225500
C	5.94493700	-0.40119100	-0.53396400
C	4.70950800	0.16054100	-0.61717600
O	4.63218500	1.33798400	-0.89675000
H	6.97746600	1.42292900	-1.02636400
H	9.15829100	0.44045200	-0.86028300
H	9.42233600	-1.91397600	-0.27392800
H	7.48994700	-3.29268200	0.13903500
O	5.11215800	-3.65005000	0.30229100
N	2.74025200	-2.84921000	0.10150400
C	2.09886300	-3.29246400	-1.12131300
C	2.91341100	-4.37274600	-1.84598900
H	2.35469100	-4.76953100	-2.72522300
H	3.88085100	-3.96945000	-2.22424200
H	3.13400700	-5.23206600	-1.17133700
H	1.10938000	-3.72368100	-0.83849300
H	1.90533400	-2.45156600	-1.82845800

H	3.04100400	-3.64645300	0.66291400
O	2.23477100	1.11900500	-0.63988400
H	-0.81388300	-0.70544700	0.28968600
H	0.07011000	0.80168600	0.77343400
H	-0.23891600	0.42582100	-1.00378400

3-Isobutylamine-DHNA methyl ester ground (**4h**)

C	0.00000000	0.00000000	0.00000000
O	-0.72766400	-1.20868700	-0.09704200
C	-1.94310200	-1.28598400	0.54217700
C	-2.81522000	-2.34370400	0.42227800
C	-2.47837000	-3.43208700	-0.30939100
C	-3.39518900	-4.40578900	-0.55104000
C	-4.62270900	-4.31577800	0.01306300
C	-5.51767400	-5.29753500	-0.20493900
C	-6.73468100	-5.23429500	0.35608900
C	-7.05554900	-4.19196700	1.13538900
C	-6.16192300	-3.21397800	1.35112600
C	-4.93714600	-3.27115900	0.79135600
C	-4.04921000	-2.26436500	1.00719900
O	-4.39476100	-1.33520500	1.70549000
H	-6.48132300	-2.37734600	1.99661500
H	-8.05738200	-4.13973800	1.59679600
H	-7.47049500	-6.03835400	0.17978100
H	-5.28949300	-6.17456500	-0.83549000
O	-3.07047700	-5.33653000	-1.25703700
N	-1.24038900	-3.66564200	-0.89279800
C	-0.17377400	-4.03072100	0.02163800
C	-0.15022500	-5.52392900	0.41192000
C	1.27354500	-5.94066500	0.82908100
H	1.31491200	-7.01762700	1.11228600
H	2.00130200	-5.79512200	-0.00226300
H	1.62751800	-5.34410100	1.70099800
C	-1.11208900	-5.84648300	1.56991300
H	-1.07878200	-6.92955800	1.82913100
H	-0.84605300	-5.27177700	2.48634700
H	-2.17003000	-5.61053800	1.32809100
H	-0.43698800	-6.13479600	-0.47933200
H	0.77199600	-3.79374400	-0.51928800
H	-0.17214000	-3.42015400	0.95340900
H	-1.30234700	-4.33285100	-1.66223100
O	-2.25135100	-0.32574000	1.21635700
H	0.93170900	-0.11395700	-0.60240600
H	-0.59650200	0.84453900	-0.42068600
H	0.28441600	0.19496800	1.06165700

3-Isobutylamine-DHNA methyl ester Radical Anion (**4h**)

C	0.00000000	0.00000000	0.00000000
O	0.91152500	1.08107000	-0.01570700
C	2.16151600	0.88208600	0.52261700
C	3.18846600	1.79556400	0.45427800

C 2.99159600 3.00912300 -0.11308500
C 4.04062600 3.84911600 -0.31556100
C 5.26970400 3.49420100 0.12774500
C 6.30010600 4.34245400 -0.04853200
C 7.52311500 4.01580300 0.39577200
C 7.71503000 2.84366200 1.01703400
C 6.68642900 1.99918000 1.19139900
C 5.45484500 2.32096200 0.74828700
C 4.42703700 1.44737200 0.91888700
O 4.65690700 0.39264100 1.47120500
H 6.90261500 1.04600600 1.70457300
H 8.72174800 2.57465300 1.38233800
H 8.36995000 4.70978400 0.25337200
H 6.18137700 5.31885200 -0.54992300
O 3.83102500 4.90311000 -0.87708800
N 1.77495600 3.50871200 -0.55772600
C 0.84563800 3.91552600 0.48054800
C 1.09710100 5.33028400 1.04426200
C -0.20709200 5.90855500 1.62692400
H -0.04960400 6.93255700 2.03726800
H -1.00109700 5.98878500 0.84906500
H -0.59866600 5.26715800 2.44954500
C 2.17209600 5.34400200 2.14624600
H 2.33698500 6.37583200 2.53289000
H 1.87356400 4.70435600 3.00801400
H 3.15790000 4.97762300 1.78939300
H 1.42369100 5.99879000 0.20990000
H -0.15936800 3.90291300 -0.00219900
H 0.80223400 3.19709400 1.33082100
H 1.89765600 4.25200000 -1.24584000
O 2.34793700 -0.19406100 1.05059300
H -0.93652400 0.33668800 -0.50351100
H 0.41861400 -0.86615700 -0.56619100
H -0.24380300 -0.28387500 1.05166300

3-Pyrrolidine-DHNA methyl ester ground (**4i**)
C 0.00000000 0.00000000 0.00000000
O -1.14227600 -0.72524900 -0.41037300
C -2.36489200 -0.09258200 -0.37837000
C -3.56232200 -0.67759700 -0.73227800
C -3.60394500 -1.96826300 -1.15472700
C -4.80122800 -2.52777800 -1.48973400
C -5.93372700 -1.78770900 -1.40391200
C -7.11324200 -2.34556600 -1.73897200
C -8.24621900 -1.63100000 -1.66291900
C -8.20329300 -0.35639900 -1.25128000
C -7.02865300 0.19966700 -0.91692900
C -5.88768100 -0.51406400 -0.99195100
C -4.70802800 0.06539000 -0.64843200
O -4.72806900 1.22109600 -0.28152600
H -7.05121600 1.25206000 -0.58425400

H -9.13503600 0.23269900 -1.18826500
H -9.21258800 -2.08875900 -1.93811600
H -7.19795800 -3.39098900 -2.08342900
O -4.85377100 -3.67973000 -1.86249700
N -2.45261100 -2.75112600 -1.25401400
C -2.32080000 -3.86341200 -0.33973800
C -1.01096300 -4.50423900 -0.81778900
C -1.15309000 -4.41537800 -2.34601200
C -2.03469300 -3.17233800 -2.57685600
H -1.45713600 -2.34732000 -3.05864800
H -2.88863500 -3.42405100 -3.24759500
H -0.16327000 -4.34721100 -2.85694600
H -1.67747900 -5.32684100 -2.72168800
H -0.14439200 -3.88600200 -0.47939800
H -0.87292500 -5.54874100 -0.44977900
H -2.26452500 -3.51798900 0.72035800
H -3.15288100 -4.59771100 -0.44417500
O -2.34780100 1.06079000 -0.00218500
H 0.88216100 -0.67668100 -0.08946500
H 0.15745900 0.88143700 -0.66643200
H -0.10308500 0.31183400 1.06684400

3-Pyrrolidine-DHNA methyl ester Radical Anion (**4i**)
C 0.00000000 0.00000000 0.00000000
O -0.91561400 1.07724400 0.01261200
C -2.26244900 0.79059400 0.01629800
C -3.26737900 1.73473300 0.01145600
C -2.97122200 3.06078700 0.00636500
C -3.98224600 3.97536400 -0.00884400
C -5.26953000 3.55074500 -0.01099700
C -6.26383300 4.45965900 -0.02335200
C -7.54501500 4.06146800 -0.02394300
C -7.83607400 2.75342500 -0.01224800
C -6.84593200 1.84767600 -0.00072400
C -5.55713800 2.24256600 -0.00003800
C -4.56831300 1.31128300 0.01048300
O -4.88893200 0.14169700 0.01820200
H -7.14245400 0.78437700 0.00816300
H -8.89021200 2.42473100 -0.01247100
H -8.35944200 4.80689100 -0.03366700
H -6.07278700 5.54689200 -0.03260600
O -3.73259100 5.16117900 -0.01964600
N -1.65455000 3.52401300 0.01163500
C -1.18006500 4.20384800 -1.17275800
C 0.23588100 4.61417700 -0.74633100
C 0.01359800 5.06745900 0.70600100
C -1.19673700 4.24193200 1.18487400
H -0.90472000 3.50781800 1.97364600
H -1.97355300 4.91406900 1.61756500
H 0.92095000 4.91345700 1.33717600
H -0.24016300 6.15480600 0.71938600

H 0.90799600 3.72221500 -0.76800900
H 0.67554800 5.41097600 -1.39222100
H -1.17918700 3.52767400 -2.06112300
H -1.77768600 5.11563500 -1.40492600
O -2.54664200 -0.38887100 0.02004100
H 1.02942200 0.42912800 -0.01029500
H -0.11944500 -0.61866000 0.92131200
H -0.13942400 -0.61435700 -0.92157700

3-Phenehtylamine-DHNA methyl ester ground (**4j**)
C 0.00000000 0.00000000 0.00000000
O -0.55771800 -1.26660600 0.29070200
C -1.64894100 -1.31741400 1.12651300
C -2.34199900 -2.46790900 1.42625000
C -1.93673500 -3.66102600 0.93034200
C -2.68393600 -4.77779400 1.13247000
C -3.80752300 -4.69666100 1.88361700
C -4.53436200 -5.80936100 2.09794300
C -5.64624700 -5.75230500 2.84655300
C -6.02970400 -4.58467900 3.38168000
C -5.30393400 -3.47632100 3.16647800
C -4.18585900 -3.52602200 2.41538800
C -3.46977400 -2.39116300 2.19647000
O -3.86740800 -1.35768400 2.69069700
H -5.66851100 -2.54179500 3.62733200
H -6.94510400 -4.53744800 3.99730100
H -6.24457500 -6.66321700 3.02344900
H -4.24982600 -6.78985700 1.67795700
O -2.30781600 -5.81680300 0.63369200
N -0.78276000 -3.87370100 0.18697300
C 0.42940000 -3.91971700 0.98199600
C 0.62044700 -5.27547500 1.68070700
C 1.95029300 -5.32453000 2.40402000
C 2.07235600 -4.82446200 3.64692600
C 3.24769600 -4.86239900 4.29372400
C 4.32230400 -5.40406200 3.70083100
C 4.21448300 -5.90440900 2.46076500
C 3.03598400 -5.86265300 1.81982700
H 2.96471200 -6.27476200 0.79893800
H 5.09631600 -6.34905500 1.96748700
H 5.29059200 -5.43625700 4.22990300
H 3.33127000 -4.44693700 5.31308400
H 1.19754200 -4.37304200 4.14533400
H -0.20036900 -5.45397700 2.41429900
H 0.55730200 -6.10082800 0.93293700
H 1.28445200 -3.75477100 0.28453700
H 0.46128900 -3.10819900 1.74634400
H -0.86020300 -4.70098100 -0.40517800
O -2.01268100 -0.25790900 1.59214300
H 0.85457800 -0.15649400 -0.69930700
H -0.75683800 0.64955900 -0.50121700

H 0.38638100 0.47477200 0.93345400

3-Phenehtylamine-DHNA methyl ester Radical Anion (**4j**)
C 0.00000000 0.00000000 0.00000000
O -0.26663900 -1.37971200 -0.15857900
C -1.28042300 -1.93346100 0.58810200
C -1.70298900 -3.23811100 0.47348700
C -1.08338600 -4.08667200 -0.38075400
C -1.57071600 -5.33972800 -0.57746600
C -2.64779400 -5.75113300 0.13239300
C -3.11547400 -7.00012100 -0.05100500
C -4.17623500 -7.43035500 0.64874200
C -4.76727900 -6.61349900 1.53201100
C -4.29978300 -5.36844500 1.71311800
C -3.23463000 -4.92931100 1.01361200
C -2.78229600 -3.66071300 1.19978500
O -3.36129400 -2.95686300 1.99966500
H -4.82754900 -4.73764000 2.44945300
H -5.64067800 -6.96723800 2.10752800
H -4.56225400 -8.45401500 0.49988500
H -2.65429100 -7.70369500 -0.76585800
O -1.01180800 -6.05174900 -1.38396100
N 0.05114800 -3.79239400 -1.12621800
C 1.28433500 -3.83806300 -0.36419700
C 1.78796000 -5.27455100 -0.15056800
C 3.13317100 -5.27439600 0.54548200
C 3.21011200 -5.19683900 1.88632000
C 4.39827800 -5.19418800 2.51039200
C 5.53126100 -5.26897800 1.79592800
C 5.46843100 -5.34469500 0.45799900
C 4.27667600 -5.34643000 -0.15935100
H 4.24207900 -5.40522100 -1.26047500
H 6.39786400 -5.40399100 -0.13470800
H 6.51012300 -5.26641700 2.30618100
H 4.44411700 -5.12919000 3.61146500
H 2.28638100 -5.13150100 2.48627200
H 1.05981600 -5.85464100 0.46348500
H 1.86091900 -5.80163000 -1.13098600
H 2.05054100 -3.27503700 -0.94789200
H 1.18454900 -3.33581600 0.62655900
H 0.11828200 -4.37544100 -1.96083200
O -1.83315400 -1.18905000 1.37041200
H 0.83257100 0.26776400 -0.69206300
H -0.89945700 0.60134600 -0.27442400
H 0.32500800 0.21225900 1.04658900

3-Cadaverine-DHNA methyl ester ground (**4k**)
C 0.00000000 0.00000000 0.00000000
O -0.36037600 -1.35997200 -0.12721700
C -1.43653300 -1.82080800 0.59271500
C -1.83761400 -3.13680300 0.60920300

C -1.14439400 -4.07075600 -0.08531100
C -1.58214000 -5.36172700 -0.11348700
C -2.68471400 -5.70170100 0.59908300
C -3.10768800 -6.98073400 0.58796700
C -4.19286400 -7.34147200 1.28968800
C -4.85868700 -6.42460900 2.00521500
C -4.43952400 -5.14987600 2.01532900
C -3.34973400 -4.78268500 1.31227000
C -2.94707100 -3.48534400 1.32827800
O -3.59179100 -2.68918300 1.97684700
H -5.02660000 -4.43534400 2.61815300
H -5.75192700 -6.72058000 2.58300200
H -4.53821100 -8.39014900 1.28026200
H -2.59081600 -7.77096800 0.01599900
O -1.00245200 -6.19806100 -0.77366800
N 0.00593900 -3.76781200 -0.80686100
C 1.24391300 -3.93600000 -0.07023300
C 1.63685400 -5.38144400 0.27271100
C 3.02142400 -5.45196300 0.94012600
C 3.42636000 -6.89662300 1.27563300
C 4.80331200 -6.96095300 1.95232500
N 5.16883300 -8.32163500 2.25752000
H 6.04688600 -8.35423000 2.77595100
H 4.47433800 -8.75850000 2.86364700
H 5.58765000 -6.52190600 1.29192900
H 4.79083000 -6.38449600 2.90656100
H 2.66217300 -7.35156500 1.95007500
H 3.44715400 -7.50383100 0.33933700
H 3.78567400 -5.00513300 0.26062100
H 3.01213600 -4.84390600 1.87589800
H 0.89054200 -5.82358600 0.97353700
H 1.64947200 -5.99341200 -0.66016600
H 2.04819700 -3.49599200 -0.70518900
H 1.21218000 -3.32976100 0.86562200
H -0.03524700 -2.84486000 -1.23416600
O -2.03473200 -0.99057700 1.24501700
H 0.90273400 0.17338300 -0.63154500
H -0.82591700 0.65386400 -0.36892800
H 0.25773500 0.23202000 1.06087300

3-Cadaverine-DHNA methyl ester radical anion (**4k**)
C 0.00000000 0.00000000 0.00000000
O 0.18908000 -1.34862500 -0.37554900
C -0.60531400 -2.30669900 0.20739000
C -0.46674100 -3.65639100 -0.02088400
C 0.51361800 -4.11400200 -0.83615000
C 0.60748100 -5.44726900 -1.10561300
C -0.25812800 -6.30468700 -0.51016000
C -0.15477700 -7.62445300 -0.76014300
C -0.99663100 -8.49208800 -0.17821300
C -1.94454600 -8.04312000 0.65594600

C -2.04977900 -6.72854000 0.90403700
C -1.20619900 -5.85304800 0.32210200
C -1.33520800 -4.52536300 0.57919700
O -2.21894100 -4.16588600 1.32750800
H -2.85012500 -6.41282000 1.59568600
H -2.63786700 -8.75645500 1.13529400
H -0.91056700 -9.57355000 -0.38341900
H 0.61091300 -8.04119900 -1.43750200
O 1.44643000 -5.86612200 -1.87506600
N 1.43730700 -3.27019200 -1.44480300
C 2.66425300 -3.07593200 -0.69623500
C 3.59403700 -4.29491300 -0.59179100
C 4.91653200 -3.94359600 0.11179400
C 5.85686700 -5.15594600 0.20956700
C 7.17020700 -4.80358100 0.92302600
N 8.04094000 -5.94966300 1.00380200
H 8.87759700 -5.73261000 1.54574700
H 7.58605100 -6.71921200 1.49516100
H 7.70558700 -3.98795900 0.38247000
H 6.96276500 -4.45375100 1.96110700
H 5.34659500 -5.98069000 0.76205700
H 6.08434300 -5.53359800 -0.81582000
H 5.42985300 -3.12528800 -0.44707700
H 4.69932400 -3.56065700 1.13730200
H 3.09604100 -5.10582100 -0.01036500
H 3.81590600 -4.68062500 -1.61498400
H 3.21836200 -2.25624000 -1.21074100
H 2.42705300 -2.70328800 0.32808400
H 1.03119400 -2.37455900 -1.70755500
O -1.45919700 -1.89911100 0.96724500
H 0.74789500 0.61535000 -0.55302600
H -1.02421600 0.33970100 -0.28525200
H 0.17763600 0.12289600 1.09501300

3-Histamine-DHNA methyl ester ground (**4l**)
C 0.00000000 0.00000000 0.00000000
O -0.51380600 -1.29459300 -0.24462100
C -1.57059800 -1.72825200 0.52158600
C -2.20706900 -2.93614800 0.34944400
C -1.77537900 -3.80975500 -0.59067600
C -2.46885600 -4.95190200 -0.83698200
C -3.56324700 -5.23607300 -0.09212500
C -4.23507000 -6.37933200 -0.32433500
C -5.31577700 -6.68490100 0.40953900
C -5.72388400 -5.84861400 1.37429300
C -5.05379200 -4.70834500 1.60305100
C -3.96642500 -4.39545600 0.87077200
C -3.30608600 -3.23087000 1.10839300
O -3.72652800 -2.50054000 1.98041600
H -5.43665000 -4.05505200 2.40631200
H -6.61355300 -6.09969800 1.97805100

H -5.86798700 -7.62246200 0.22222900
H -3.92847800 -7.09513500 -1.10697800
O -2.07282700 -5.68378300 -1.71863500
N -0.64371600 -3.64813800 -1.37969700
C 0.59791300 -3.94742100 -0.69218200
C 0.87632800 -5.45705300 -0.62142500
C 2.21423400 -5.73196800 -0.00148600
C 3.32977000 -6.15504000 -0.60990000
N 4.34441400 -6.27468400 0.30399300
C 3.70853400 -5.84702400 1.45006000
N 2.49647400 -5.55172900 1.22021300
H 4.26195700 -5.40533900 2.29624200
H 5.29496900 -5.98776900 0.07299900
H 3.45372800 -6.36780300 -1.68473900
H 0.09990700 -5.97399300 -0.01079800
H 0.84467700 -5.89565800 -1.64640300
H 1.41925800 -3.46119900 -1.26991300
H 0.61828800 -3.52360400 0.33951000
H -0.71187000 -4.17310300 -2.25200800
O -1.95528000 -0.96261100 1.38033600
H 0.83031700 0.17875500 -0.72297500
H -0.79153700 0.76859800 -0.16868400
H 0.41065200 0.06205600 1.03612300

3-Histamine-DHNA methyl ester radical anion (**4l**)
C 0.00000000 0.00000000 0.00000000
O -0.32772100 -1.36768400 -0.14780500
C -1.38570000 -1.86309400 0.57827400
C -1.85162800 -3.15453800 0.48434400
C -1.23597800 -4.04733800 -0.32636800
C -1.75990600 -5.28819200 -0.50485300
C -2.87156600 -5.64293300 0.18189800
C -3.37517300 -6.88075000 0.01911800
C -4.46972300 -7.25583900 0.69818900
C -5.05961200 -6.39451700 1.53892300
C -4.55705000 -5.16019700 1.69845700
C -3.45695200 -4.77686400 1.02071100
C -2.96778800 -3.51899600 1.18581400
O -3.54881200 -2.77245000 1.94453400
H -5.08548800 -4.49113600 2.39967700
H -5.96070200 -6.70287400 2.09752000
H -4.88423900 -8.27082200 0.56769800
H -2.91624300 -7.61958100 -0.66089300
O -1.20199200 -6.04035300 -1.27478000
N -0.06858400 -3.81277900 -1.04156600
C 1.13604100 -3.88228900 -0.23662100
C 1.59058600 -5.32949300 0.00934700
C 2.89326200 -5.36860800 0.75190900
C 4.10086300 -5.69660400 0.27473400
N 5.03937900 -5.59754500 1.26891300
C 4.25877100 -5.15259700 2.31456000

N	3.04416300	-5.04084600	1.96619700
H	4.67823600	-4.56602000	3.14964400
H	5.96392200	-5.21139700	1.08091300
H	4.34279500	-5.98978000	-0.76036500
H	0.83351600	-5.88483200	0.61048500
H	1.70209300	-5.86133400	-0.96464900
H	1.93844300	-3.34835600	-0.79863600
H	1.01462000	-3.36589900	0.74471600
H	0.00419900	-4.42052400	-1.85796500
O	-1.93455600	-1.07824300	1.32285800
H	0.86164000	0.21817900	-0.67383000
H	-0.86231100	0.63958600	-0.30508600
H	0.30846900	0.21073800	1.05189000

3-Tyramine-DHNA methyl ester ground (**4m**)

C	0.00000000	0.00000000	0.00000000
O	-0.93676400	-1.01863200	0.29793100
C	-2.26195400	-0.65407800	0.37825100
C	-3.31414400	-1.53745900	0.45171700
C	-3.09040000	-2.87029200	0.51977500
C	-4.13619000	-3.73953700	0.53200300
C	-5.40419300	-3.25858300	0.51854200
C	-6.44069300	-4.11832900	0.55059000
C	-7.70131500	-3.65614300	0.55458100
C	-7.92833000	-2.33511900	0.52876000
C	-6.89459200	-1.48061000	0.48655100
C	-5.62631800	-1.93786000	0.48009200
C	-4.59407000	-1.05403200	0.43349700
O	-4.86049800	0.12631400	0.36358300
H	-7.13667600	-0.40426000	0.46555900
H	-8.96528600	-1.95354500	0.54293600
H	-8.55429000	-4.35696100	0.58532800
H	-6.30009500	-5.21312900	0.58050400
O	-3.89129700	-4.92578700	0.57126700
N	-1.83872000	-3.47565200	0.60193500
C	-1.35333600	-3.56312600	1.96502700
C	0.03548400	-4.21940500	2.03836200
C	1.13458800	-3.34014200	1.48158600
C	1.61795700	-2.31964300	2.21044100
C	2.59978900	-1.53698600	1.73809000
C	3.13228300	-1.74362500	0.52147300
C	2.64381000	-2.76531300	-0.20324200
C	1.66652000	-3.55551500	0.26645600
H	1.29899300	-4.38712500	-0.35958000
H	3.05774900	-2.95417400	-1.20910300
O	4.11567300	-0.94332900	0.02836200
H	4.32577800	-1.25691900	-0.86662000
H	2.97134700	-0.70415000	2.35850900
H	1.20001900	-2.11947300	3.21112800
H	0.28147100	-4.43468200	3.10556600
H	0.01659000	-5.21076200	1.52608500

H -1.33843500 -2.56409100 2.46047000
H -2.05832400 -4.20379400 2.54787900
H -1.83927900 -4.39893600 0.16574100
O -2.48964600 0.53742300 0.35027800
H 0.93962000 -0.48504600 -0.34741200
H -0.36466600 0.64795600 -0.83191600
H 0.21335300 0.60149500 0.91489800

3-Tyramine-DHNA methyl ester radical anion (**4m**)
C 0.00000000 0.00000000 0.00000000
O 0.99342800 -0.99833600 -0.14257400
C 2.28868300 -0.57642600 -0.34204000
C 3.39465100 -1.39335100 -0.29375900
C 3.25799300 -2.72645900 -0.10591600
C 4.35838300 -3.51848300 0.00015500
C 5.59154600 -2.96836400 -0.12636400
C 6.68147600 -3.75532700 -0.04113400
C 7.90802600 -3.22731000 -0.18103600
C 8.04752800 -1.91346400 -0.40831300
C 6.96099000 -1.12957600 -0.48271100
C 5.72655000 -1.65275100 -0.34098700
C 4.63967700 -0.83920500 -0.41654200
O 4.82907900 0.34727300 -0.57617600
H 7.13161900 -0.05566100 -0.67024000
H 9.05605500 -1.47979800 -0.53434600
H 8.80440200 -3.86917800 -0.11642600
H 6.61303800 -4.84291800 0.13694500
O 4.19172400 -4.70374500 0.19011000
N 2.04740900 -3.41040800 -0.02458800
C 1.53029100 -3.77949900 -1.32752000
C 0.18655600 -4.51995200 -1.22261500
C -0.95217200 -3.61973400 -0.79361600
C -1.52274300 -2.78390800 -1.67800100
C -2.54049800 -1.98714400 -1.31883000
C -3.02325600 -1.99477300 -0.06452000
C -2.44763300 -2.83223200 0.81581200
C -1.43383200 -3.63617200 0.46082000
H -0.99426100 -4.31288600 1.21408400
H -2.81932400 -2.85463200 1.85509700
O -4.04314400 -1.17693400 0.31185000
H -4.20641400 -1.33011400 1.25702400
H -2.98401100 -1.30842100 -2.06664200
H -1.14766600 -2.74921300 -2.71445100
H -0.07488600 -4.94484000 -2.22106200
H 0.28576500 -5.39542400 -0.53757500
H 1.43503500 -2.89311900 -1.99760400
H 2.25935800 -4.47422200 -1.81022400
H 2.12164800 -4.23441500 0.57392600
O 2.43751600 0.61084800 -0.54335800
H -0.89509200 -0.46715000 0.46843400
H 0.34440300 0.81241100 0.68285200

H -0.27875300 0.40642000 -1.00073700

3-Tryptamine-DHNA methyl ester ground (**4n**)
C 0.00000000 0.00000000 0.00000000
O -1.15661700 -0.78325800 -0.22057700
C -2.31618700 -0.14051200 -0.58735400
C -3.52184100 -0.77386700 -0.78469700
C -3.61504700 -2.11815700 -0.65630300
C -4.81565800 -2.74187800 -0.77534000
C -5.91632300 -2.00624900 -1.05948700
C -7.10362700 -2.62724200 -1.18938600
C -8.20540500 -1.91800200 -1.47762700
C -8.11974600 -0.58980200 -1.63634200
C -6.93516400 0.02770700 -1.50585900
C -5.82386000 -0.67817300 -1.21692500
C -4.63359900 -0.03470600 -1.08108100
O -4.61050700 1.16921400 -1.22446600
H -6.92346300 1.12282100 -1.64449600
H -9.02639100 -0.00586800 -1.87344300
H -9.18044700 -2.42475100 -1.58530400
H -7.21429100 -3.71906200 -1.06873200
O -4.85112900 -3.94465200 -0.62712300
N -2.55114000 -2.97250300 -0.40329500
C -1.80165700 -3.31859600 -1.59114900
C -0.51153800 -3.98338900 -1.20779700
C 0.74698900 -3.57537800 -1.45350700
C 1.58175500 -4.45985900 -0.88113400
N 0.89851400 -5.48814800 -0.27823700
C -0.40795300 -5.10963800 -0.48026100
H -1.25658200 -5.67506200 -0.06041500
H 1.22532100 -5.94809600 0.57048200
C 2.90803900 -4.27477000 -0.92450300
C 3.38567000 -3.19744300 -1.56962800
C 2.54931700 -2.32240200 -2.15516600
C 1.21859700 -2.50202500 -2.10169500
H 0.54333300 -1.76897800 -2.57302400
H 2.95943200 -1.44085800 -2.67783500
H 4.47560000 -3.02996400 -1.61824400
H 3.58865300 -4.99688500 -0.44253400
H -2.39786400 -4.03883200 -2.19835200
H -1.61824300 -2.43140200 -2.23875600
H -2.84305200 -3.81791000 0.08696600
O -2.24004400 1.06264900 -0.72201800
H 0.82072800 -0.68569000 0.31683200
H 0.30551400 0.50917700 -0.94514700
H -0.18180500 0.73818400 0.81713100

3-Tryptamine-DHNA methyl ester radical anion (**4n**)
C 0.00000000 0.00000000 0.00000000
O 0.86451500 -1.09657500 0.22370800
C 2.16857000 -0.83465400 0.57454600

C	3.12530000	-1.80371700	0.77271200
C	2.80244600	-3.11357400	0.66168900
C	3.75652400	-4.07276900	0.78146100
C	5.03200600	-3.70531100	1.04881000
C	5.97435500	-4.65772800	1.17949000
C	7.24288200	-4.31569300	1.45127700
C	7.56831700	-3.02313400	1.59278600
C	6.62749200	-2.07487200	1.46155100
C	5.35091100	-2.41080600	1.18908600
C	4.41254600	-1.43615200	1.05217900
O	4.75947100	-0.28119900	1.17962300
H	6.95200500	-1.02705200	1.58562800
H	8.61227200	-2.74138700	1.81634300
H	8.01775600	-5.09475900	1.55955300
H	5.74526400	-5.73243400	1.07272500
O	3.42168800	-5.23046200	0.64909200
N	1.52604500	-3.60496000	0.42682900
C	0.71871200	-3.69285600	1.62390400
C	-0.71655200	-3.93608900	1.25761100
C	-1.78794100	-3.16067400	1.50579100
C	-2.85856700	-3.75416100	0.95055700
N	-2.52790800	-4.94839800	0.35714000
C	-1.16628700	-4.98470700	0.54584300
H	-0.53501600	-5.78673300	0.12823400
H	-2.98805000	-5.29572400	-0.48328400
C	-4.06455800	-3.17250300	0.99982200
C	-4.18403600	-1.99389600	1.63351000
C	-3.11464800	-1.40974100	2.20202400
C	-1.90286600	-1.98765700	2.14255700
H	-1.03136300	-1.49075300	2.59978700
H	-3.23082900	-0.43949000	2.71545700
H	-5.17028700	-1.50111500	1.68676400
H	-4.93792500	-3.65747900	0.53184100
H	1.07272500	-4.55414500	2.23689200
H	0.82138600	-2.78509700	2.26075500
H	1.54102700	-4.50436100	-0.05393600
O	2.46465900	0.33568000	0.69361500
H	-0.99400500	-0.40573000	-0.30246300
H	-0.12597200	0.58830700	0.94017600
H	0.39019100	0.63856700	-0.82804200

3-β-alanine-ethyl ester-DHNA methyl ester ground (**4o**)

C	0.00000000	0.00000000	0.00000000
O	-0.89202100	-1.03267300	0.37040200
C	-2.14481900	-0.67975600	0.81516400
C	-3.12386100	-1.58170100	1.16335700
C	-2.87524900	-2.91142900	1.11413900
C	-3.86046200	-3.80744600	1.38187400
C	-5.08764700	-3.35536200	1.73247700
C	-6.05946300	-4.24542200	2.00732200
C	-7.28094600	-3.81877000	2.36201200

C -7.52985100 -2.50411400 2.44229600
C -6.55962400 -1.61816500 2.16780000
C -5.32985100 -2.03929100 1.81137600
C -4.36127200 -1.12774900 1.52844200
O -4.63876100 0.05005700 1.60728900
H -6.82095400 -0.54846100 2.24663300
H -8.53544900 -2.15304400 2.73321000
H -8.08008200 -4.54638800 2.58787100
H -5.89285100 -5.33547200 1.95403900
O -3.59508100 -4.98751700 1.29878100
N -1.65193000 -3.48890400 0.80087200
C -0.74306400 -3.54135000 1.92812300
C 0.66431500 -3.93282400 1.45241300
C 1.67692300 -4.01398000 2.58274100
O 1.42198700 -3.86381500 3.75613000
O 2.91956300 -4.27918600 2.08536500
C 3.96785000 -4.38179500 3.03024600
C 5.27641500 -4.67130300 2.28402200
H 6.12985900 -4.75823200 2.99573600
H 5.20883700 -5.62635700 1.71330200
H 5.51495300 -3.85558200 1.56281600
H 4.05491100 -3.41871200 3.59151000
H 3.74258900 -5.21387700 3.74236200
H 0.62304100 -4.92568600 0.94512700
H 1.02298400 -3.18046100 0.71065500
H -0.70019100 -2.56650400 2.46919600
H -1.11774600 -4.30509100 2.65047200
H -1.76911300 -4.41488000 0.38829500
O -2.37304400 0.51007200 0.87695600
H 0.95014500 -0.47609100 -0.33841800
H -0.42447800 0.59147900 -0.84612400
H 0.22155000 0.65152400 0.87885800

3-β-alanine-ethyl ester-DHNA methyl ester radical anion (**4o**)
C 0.00000000 0.00000000 0.00000000
O -0.66567600 1.24747200 -0.00116700
C -1.93616600 1.30476400 -0.52503700
C -2.71248100 2.44059000 -0.54740800
C -2.22666800 3.60865700 -0.06573300
C -3.01446900 4.71404000 -0.01402300
C -4.28312800 4.64399600 -0.48187200
C -5.05849100 5.74360700 -0.43896600
C -6.31665800 5.69735500 -0.90236000
C -6.79831200 4.55339400 -1.40840400
C -6.02373400 3.45800800 -1.45027100
C -4.75854000 3.49699200 -0.98714000
C -3.99073900 2.37547600 -1.02944600
O -4.47536700 1.36327100 -1.48881500
H -6.47262900 2.54283300 -1.87420800
H -7.83413700 4.51512600 -1.78881300
H -6.95504500 6.59755700 -0.86834900

H -4.69744100 6.70404900 -0.03153300
O -2.54153700 5.72928000 0.45022000
N -0.93673800 3.80770800 0.40816700
C 0.02571600 4.04852900 -0.64784900
C 1.45334300 3.98732100 -0.08357700
C 2.52306200 4.23588400 -1.13406300
O 2.30851200 4.52670700 -2.28895400
O 3.76462000 4.08220600 -0.58928000
C 4.86330100 4.28357700 -1.45794800
C 6.16061200 4.05896600 -0.67083900
H 7.05260400 4.20736000 -1.32259700
H 6.23909100 4.77111200 0.18303800
H 6.20587900 3.02353000 -0.26064100
H 4.80195800 3.55719600 -2.30572900
H 4.83358200 5.32867500 -1.85430700
H 1.56801000 4.75005600 0.72258900
H 1.62705500 2.98090700 0.36570400
H -0.08224700 3.31314900 -1.48000400
H -0.16266700 5.06224100 -1.07490200
H -0.90376400 4.55377900 1.10363400
O -2.37557100 0.26645800 -0.97274100
H 1.00182100 0.14924600 0.46695200
H -0.57097800 -0.74145300 0.60842900
H 0.14505700 -0.36038300 -1.04644200

3-Taurine-DHNA methyl ester ground (**4p**)
C 0.00000000 0.00000000 0.00000000
O -1.21258900 -0.70223300 -0.19387300
C -1.21561000 -2.06095400 0.03372000
C -2.34390600 -2.83884500 -0.08613400
C -2.30359400 -4.17436100 0.13644200
C -3.44156100 -4.91453200 0.08075900
C -4.61128100 -4.30758500 -0.23223400
C -5.73693100 -5.04319500 -0.29560100
C -6.90393100 -4.46038300 -0.60989200
C -6.94612100 -3.14440300 -0.86194200
C -5.82338100 -2.41160100 -0.79814800
C -4.64870500 -2.99131200 -0.48261900
C -3.52288400 -2.23368600 -0.41379800
O -3.59142400 -1.04566500 -0.64069100
H -5.91289200 -1.33215600 -1.01142300
H -7.90673500 -2.66540400 -1.12050900
H -7.82862700 -5.06144700 -0.66251300
H -5.74299400 -6.12916700 -0.09698300
O -3.36155200 -6.10248900 0.30965500
N -1.16309300 -4.90885100 0.43753300
C -0.31777400 -5.16979900 -0.71058600
C 1.05969000 -5.64891900 -0.22799900
S 2.11724100 -5.98309700 -1.62806700
O 3.45043800 -6.31282700 -1.16240400
O 1.48932500 -6.96064600 -2.49574900

O 2.26143300 -4.79166900 -2.24788300
H 3.15698500 -4.67892100 -2.50775600
H 1.54432600 -4.87829400 0.41612300
H 0.95956500 -6.58779300 0.36493800
H -0.81335000 -5.95923600 -1.32470100
H -0.21620700 -4.26692700 -1.35781300
H -1.39152200 -5.77649500 0.92435600
O -0.16038300 -2.54658100 0.38243500
H 0.33400200 -0.09147300 1.06121900
H 0.78068400 -0.37767200 -0.70287100
H -0.18972400 1.07635200 -0.22225900

3-Taurine-DHNA methyl ester radical anion (**4p**)
C 0.00000000 0.00000000 0.00000000
O -1.21258900 -0.70223300 -0.19387300
C -1.21561000 -2.06095400 0.03372000
C -2.34390600 -2.83884500 -0.08613400
C -2.30359400 -4.17436100 0.13644200
C -3.44156100 -4.91453200 0.08075900
C -4.61128100 -4.30758500 -0.23223400
C -5.73693100 -5.04319500 -0.29560100
C -6.90393100 -4.46038300 -0.60989200
C -6.94612100 -3.14440300 -0.86194200
C -5.82338100 -2.41160100 -0.79814800
C -4.64870500 -2.99131200 -0.48261900
C -3.52288400 -2.23368600 -0.41379800
O -3.59142400 -1.04566500 -0.64069100
H -5.91289200 -1.33215600 -1.01142300
H -7.90673500 -2.66540400 -1.12050900
H -7.82862700 -5.06144700 -0.66251300
H -5.74299400 -6.12916700 -0.09698300
O -3.36155200 -6.10248900 0.30965500
N -1.16309300 -4.90885100 0.43753300
C -0.31777400 -5.16979900 -0.71058600
C 1.05969000 -5.64891900 -0.22799900
S 2.11724100 -5.98309700 -1.62806700
O 3.45043800 -6.31282700 -1.16240400
O 1.48932500 -6.96064600 -2.49574900
O 2.26143300 -4.79166900 -2.24788300
H 3.15698500 -4.67892100 -2.50775600
H 1.54432600 -4.87829400 0.41612300
H 0.95956500 -6.58779300 0.36493800
H -0.81335000 -5.95923600 -1.32470100
H -0.21620700 -4.26692700 -1.35781300
H -1.39152200 -5.77649500 0.92435600
O -0.16038300 -2.54658100 0.38243500
H 0.33400200 -0.09147300 1.06121900
H 0.78068400 -0.37767200 -0.70287100
H -0.18972400 1.07635200 -0.22225900
